# Direct access to spirocycles by Pd/WingPhos-catalyzed enantioselective cycloaddition of 1,3-enynes

**DOI:** 10.1038/s41467-021-25981-x

**Published:** 2021-09-27

**Authors:** Long Li, Shan Wang, Pengfei Luo, Ran Wang, Zheng Wang, Xiaoguang Li, Yuhua Deng, Fangzhi Peng, Zhihui Shao

**Affiliations:** 1 Key Laboratory of Medicinal Chemistry for Natural Resource, Ministry of Education, School of Chemical Science and Technology, Yunnan Provincial Center for Research & Development of Natural Products, and State Key Laboratory for Conservation and Utilization of Bio-Resources in Yunnan, Yunnan University, Kunming, 650091 China; 2 Kunming Institute of Physics, Kunming, 650223 China; 3 Institute for Advanced Study, Shenzhen University, Shenzhen, 518060 China

**Keywords:** Asymmetric catalysis, Synthetic chemistry methodology

## Abstract

Spirocycles play an important role in drug discovery and development. The direct, catalytic, and enantioselective synthesis of spirocycles from readily available starting materials and in an atom economic manner remains a highly sought-after task in organic synthesis. Herein, an enantioselective Pd-hydride-catalyzed cycloaddition method for the synthesis of spirocyclic compounds directly from two classes of commonly available starting materials, 1,3-enynes and cyclic carbon−hydrogen (C−H) bonds, is reported. The reactions employ a chiral Pd/WingPhos catalyst to both suppress the formation of bis-allenyl by-products and control the stereoselectivity. 1,3-Enynes are used as dielectrophilic four-carbon units in the cycloaddition reactions, which also enables an enyne substrate-directed enantioselectivity switch with good levels of stereocontrol. The present spirocycle synthesis tolerates a broad range of functional groups of 1,3-enyne substrates, including alcohols, esters, nitriles, halides, and olefins. A variety of diverse cyclic nucleophiles, including pharmaceutically important heterocycles and carbocycles, can be flexibly incorporated with spiro scaffolds.

## Abstract (web summary)

Spirocycles are traditionally difficult structures to synthesize due to the congested nature of the central atom. Here the authors show a method to synthesize quaternary carbon spirocycles in one step from 1,3-enynes and pyrazolidine-type heterocycles, both relatively unactivated structures, proceeding via palladium catalysis.

## Introduction

Spirocyclic scaffolds are widely present in numerous natural products and biologically active compounds^1–7^. Moreover, the uniquely rigid structures of spirocyclic scaffolds can reduce the conformational entropy penalty upon binding to a protein target^8,9^. As a result, spiro scaffolds have been increasingly utilized in drug discovery and development programs^8,9^. The development of efficient asymmetric approaches for constructing spirocyclic compounds has attracted much attention^10–18^. Despite considerable progress in the asymmetric synthesis of spirocycles, the methods that are direct, catalytic, enantioselective, and atom economic^19^ and that rely on the use of commonly available starting materials are in high need.

The direct addition of enols/enolates to unactivated unsaturated hydrocarbons (hydroalkylation) with catalysis by transition-metal hydrides (MH) has been attracting increasing attention as an atom-economical strategy for the C–C bond formation. Elegant progress on asymmetric variants of these reactions has recently been made^20–39^. However, the potential of this technique in the direct asymmetric synthesis of spirocyclic compounds has remained elusive. Moreover, the reported studies focused on asymmetric mono-hydroalkylation. In contrast, transition-metal-hydride-catalyzed asymmetric annulative double hydroalkylation sequences of unactivated unsaturated hydrocarbons with enols/enolates are scarce. In addition, effective chiral catalyst systems that are applicable to the establishment of asymmetric addition of enols/enolates to unsaturated hydrocarbons are comparatively limited. As our continuous interest in asymmetric cycloadditions^40–46^, we explored the possibility of transition-metal hydride-based cycloaddition strategy for the direct catalytic asymmetric spirocycle synthesis.

**Fig. 1:**
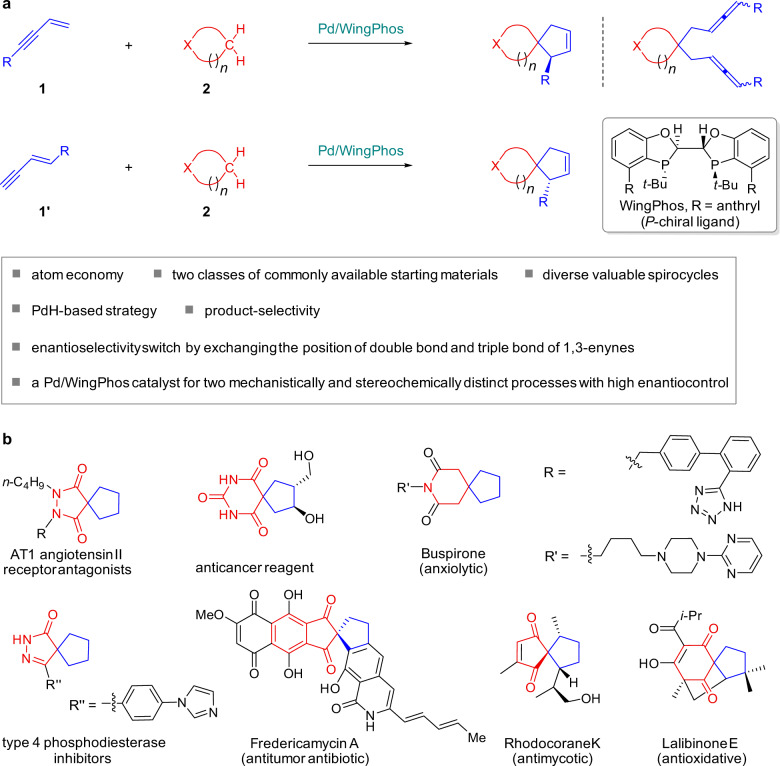
Transition-metal hydride-based cycloaddition strategy for the direct catalytic asymmetric spirocycle synthesis. **a** Pd/WingPhos-catalyzed enantioselective cycloaddition reactions of 1,3-enynes as dielectrophilic C4 synthons. **b** Selected bioactive spirocyclic molecules relevant to this study.

Here, we report the successful development of Pd-hydride catalyzed cycloaddition of 1,3-enynes employing P-chiral WingPhos as the ligand that enables the direct, atom-economical, and enantioselective synthesis of spirocycles from two classes of commonly available starting materials (Fig. 1a). The challenging product selectivity issue of cycloaddition products versus double intermolecular hydroalkylation products has been addressed. A chiral Pd/WingPhos catalyst is able to affect both 1,3-enynes **1** having a terminal double bond and 1,3-enynes **1′** having a terminal triple bond to engage in the asymmetric cycloaddition reactions with high levels of enantioselectivity switch. Mechanistic studies suggest that two cycloaddition reactions involve mechanistically and stereochemically distinct processes. For the cycloaddition reaction with 1,3-enynes **1**, the previously unreported enantioselective intermolecular hydroalkylation step of 1,3-enynes **1** with cyclic enols/enolates forms allene intermediates with axial chirality which serves as a chiral relay during cyclization processes involving a very high efficiency of axial-to central chirality transfer, whereas for the cycloaddition with 1,3-enynes **1′**, the marked stereocenter of the spirocyclic products is directly introduced in the enantioselective intermolecular hydroalkylation step of 1,3-enynes **1′** with enols/enolates to form allene intermediates with central chirality, which has not been previously realized.

## Results

### Optimization of reaction conditions

We began the research by examining the cycloaddition reaction of 1,3-enynes having a terminal double bond with pyrazolidine-3,5-diones, a class of important heterocyclic scaffolds, which are widely present in biologically active molecules and pharmaceutical compounds^47–50^, for the asymmetric synthesis of spiro-pyrazolidine-3,5-diones. Spiro-pyrazolidine-3,5-diones have been shown to possess valuable biological properties, as exemplified by their use as AT1 angiotensin II receptor antagonists (Fig. 1b). However, there are no reports of catalytic asymmetric synthesis of spiro-pyrazolidine-3,5-diones, thus limiting their potential applications in discovering chiral bioactive molecules. The model reaction of 1,3-enyne **1a** with pyrazolidine-3,5-dione **2a** was initially investigated at CH_3_CN in the presence of various chiral palladium catalysts. Most axially chiral ligands tested were either unreactive or gave double intermolecular hydroalkylation product rather than cyclization product. Selected results are shown in Table 1 (for the details, see Supplementary Tables 1–3 in the Supplementary Information). When planar-chiral Xylyl-PhanePhos (**L6**) was used as the ligand, spirocyclic product **3aa** was obtained in 44% yield but with only 21% ee, together with double intermolecular hydroalkylation product **4aa** in 16% yield (Table 1, entry 6). Obviously, achieving the enantioselective cycloaddition of 1,3-enyne **1a** with pyrazolidine-3,5-dione **2a** with high enantiocontrol has posed a unique challenge, and it requires an efficient chiral catalytic system which has multifunctional roles (reactive, product-selective/pathway-selective, and enantioselective). Such a chiral catalyst should not only activate 1,3-enye **1a** to generate the terminal Pd-butadienyl complex by PdH-mediated migratory insertion and catalyze the selective formation of cycloaddition product rather than double intermolecular hydroalkylation product, but also provide high levels of enantiocontrol. To our knowledge, the catalytic asymmetric cycloaddition which involves the terminal metal-butadienyl intermediates has not been previously reported.

**Table 1:** Chiral ligand effects on product-selectivity and enantioselectivity^a^.


Entry	Ligand	*T* (^o^C)	Yield (%)^b^ of 3aa	Yield (%)^b^ of 4aa	ee (%)^c^ of 3aa
1	**L1**	50	0	0	–
2	**L2**	50	0	30	–
3	**L3**	50	0	79	–
4	**L4**	50	0	31	–
5	**L5**	50	10	16	–
6	**L6**	50	44	16	21
7	**L7**	50	37	7	90
8	**L7**	30	22	0	96
9	**L8**	30	Trace	0	–
10	**L9**	30	16	0	69
11	**L10**	30	45	0	44

^a^Reaction conditions: **1a** (0.12 mmol), **2a** (0.1 mmol), [Pd(allyl)Cl]_2_ (2.5 mol%), chiral ligand (5 mol%), Et_3_N (2 equiv), CH_3_CN (0.5 mL), 22 h.

^b^Isolated yields.

^c^Determined by chiral HPLC.

Tang and co-workers recently introduced P-chiral BIBOP‐type ligands for various asymmetric catalytic reactions^51–54^. To our knowledge, this class of chiral ligands have not been successfully applied in the catalytic asymmetric hydroalkylation process of unactivated unsaturated hydrocarbons with enols/enolates. We tested this type of chiral ligands in the reaction of 1,3-enyne **1a** and pyrazolidine-3,5-dione **2a**, and found that WingPhos afforded promising results, with the formation of the desired spirocyclic product **3aa** as the major product with high enantiocontrol (Table 1, entry 7). To further suppress the second intermolecular hydroalkylation to form the undesired non-annulative double hydroalkylation product **4aa**, the temperature was decreased. The non-annulative double hydroalkylation product **4aa** was fully suppressed, unfortunately, the reaction conversion decreased (Table 1, entry 8). We found that bases had a crucial effect on the reaction conversion (Table 2). BnN(Me)_2_, which has rarely been used as the base in organic synthesis, provided the desired spirocyclic product **3aa** in 71% yield with 90% ee (Table 2, entry 5 versus entries 1–4).

**Table 2:** Base effects^a^.


Entry	Base	Yield (%)^b^ of 3aa	Yield (%)^b^ of 4aa	ee (%)^c^ of 3aa
1	K_2_CO_3_	Trace	0	–
2	Cs_2_CO_3_	Trace	0	–
3	DIPEA	Trace	0	–
4	Et_3_N	22	0	96
5	BnN(Me)_2_	71	0	90

^a^Reaction conditions: **1a** (0.12 mmol), **2a** (0.1 mmol), [Pd(allyl)Cl]_2_ (2.5 mol%), **L7** (5 mol%), base (2 equiv), CH_3_CN (0.5 mL), 30 °C, 22 h.

^b^Isolated yields.

^c^Determined by chiral HPLC.

*DIPEA N,N*-diisopropylethylamine, *PMP* 4-methoxyphenyl.

### Substrate scope

**Fig. 2:**
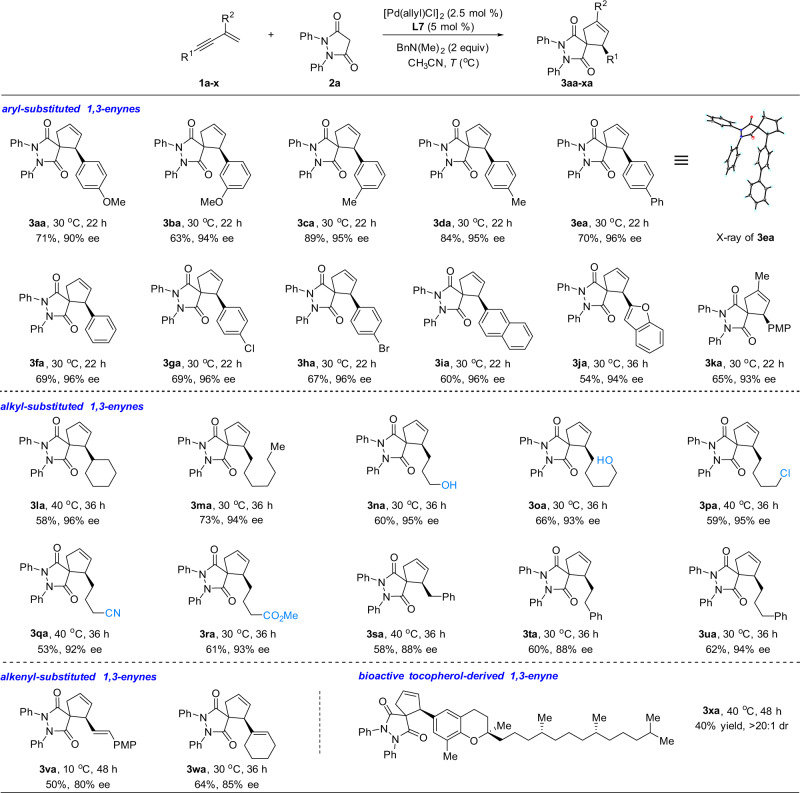
Pd/WingPhos catalyzed cycloaddition of 1,3-enynes having a double bond with pyrazolidine-3,5-dione. The reactions were carried out on a 0.1-mmol scale. Isolated yields are reported.

With optimized chiral catalyst system and reaction conditions, a range of 1,3-enynes **1** have been examined for the cycloaddition reaction with **2a** (Fig. 2).

(Hetero)aryl-substituted 1,3-enynes afforded spirocyclized products **3aa**–**3ja** in good yields with high enantioselectivities. A 1,3-disubstituted enyne **1k** also led to the spirocyclized product **3ka** in 65% yield with 93% ee. Notably, alkyl-substituted 1,3-enynes were also suitable substrates for this transformation (**3la**–**3ua**). Several functional groups were well tolerated, including esters, nitriles, halides, and free alcohols. Furthermore, alkenyl-substituted 1,3-enynes also underwent the cycloaddition to afford the corresponding spirocyclized products (**3va**–**3wa**). It is worth noting that such substrates have rarely been used in the reactions by transition-metal hydride catalysis, as could potentially generate multiple regioisomers of the butadienyl palladium intermediates and could lead to side products. 1,4-Disubstituted enynes did not work due to steric bulkiness. The absolute configuration of **3ea** was determined by X-ray crystal analysis (for the details, see Supplementary Table 4 in the Supplementary Information).

**Fig. 3:**
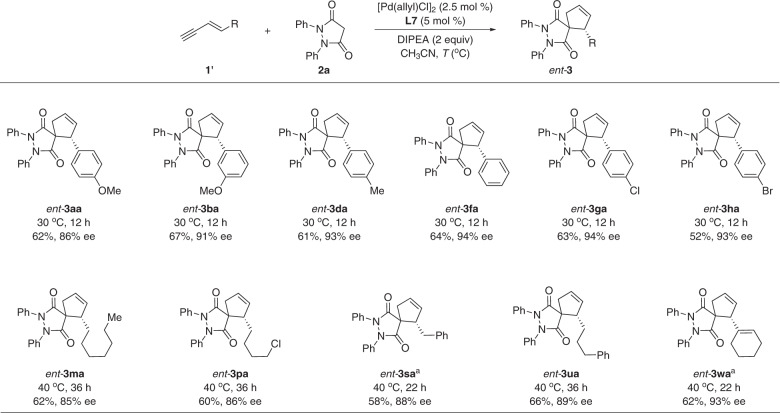
Pd/WingPhos catalyzed cycloaddition of 1,3-enynes having a triple bond with enantioselectivity switch. The reactions were carried out on a 0.1-mmol scale. Isolated yields are reported. ^a^**L8** was used as ligand. DIPEA *N,N*-diisopropylethylamine.

Notably, Pd/WingPhos catalyst also permitted the asymmetric cycloaddition reaction of 1,3-enynes having a terminal triple bond (Fig. 3). More interestingly, a switch of enantioselectivity was observed as compared with 1,3-enyne substrates having a terminal double bond.

**Fig. 4:**
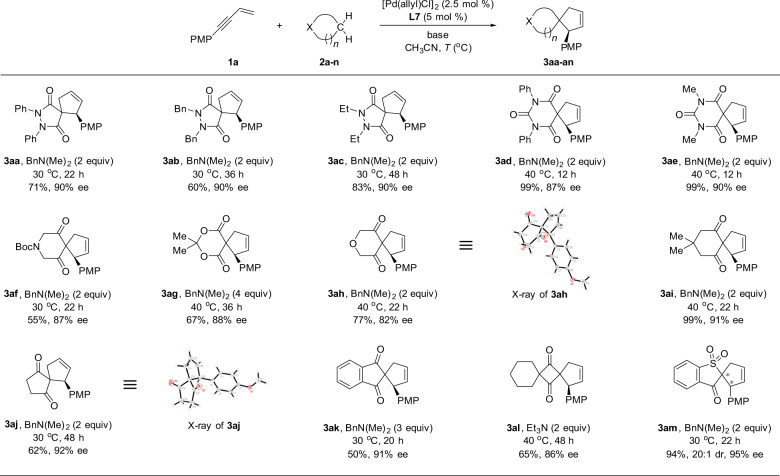
Pd/WingPhos catalyzed enyne cycloaddition with various cyclic pronucleophiles. The reactions were carried out on a 0.1-mmol scale. Isolated yields are reported.

A variety of diverse pronucleophiles, including biologically active structural cores, can be flexibly incorporated into spirocyclic scaffolds by Pd/WingPhos catalyzed enyne cycloaddition. As illustrated in Fig. 4, pyrazolidine-3,5-diones **2a-2c** reacted smoothly with 1,3-enyne **1a**, delivering spiro-pyrazolidine-3,5-diones **3aa-3ac**. Barbiturates **2d-2e** also took part in the enyne cycloaddition to produce spiro-barbiturates **3ad-3ae** in good yields with good enantioselectivityies. Piperidine-3,5-dione **2f** also participated, delivering spiro-piperidine-3,5-dione **3af** in 55% yield with 87% ee. O-heterocycles, such as meldrum’s acid **2g** and 2*H*-pyran-3,5(4*H*,6*H*)-dione **2h**, were also suitable reaction partners for the spiroannulation. Six-, five-, and four-membered carbocycles **2i-2l** all took part in the enyne cycloaddition reactions to deliver spirocyclized products **3ai-3al**. Prochiral pronucleophiles also underwent smoothly the enyne cycloaddition reactions. For example, benzo[b]thiophen-3(2*H*)-one 1,1-dioxide **2m** afforded the spirocyclic product **3am** in good yield (94%) with both high diastereoselectivity (20:1 dr) and enantioselectivity (95% ee). Interestingly, acyclic 1,3-dicarbonyl compounds did not work under the present chiral catalyst system. The absolute configuration of **3ah** and **3aj** was determined by X-ray crystal analysis, respectively (for the details, see Supplementary Tables 5–6 in Supplementary Information).

### Synthetic applications

**Fig. 5:**
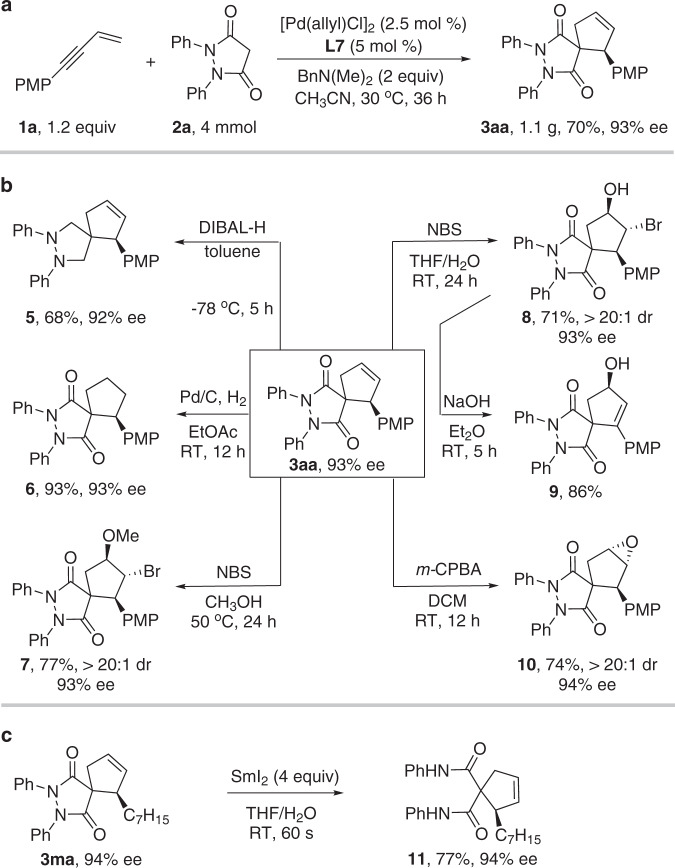
Synthetic applications. **a** Gram-scale experiment. **b** Synthetic transformations of product **3aa** (hydrogenation, alkoxybromination, hydroxybromination, and epoxidation of C=C double bond, reduction of amide group). **c** Synthetic transformation of product **3ma** (reductive cleavage of the N–N bond). DIBAL-H diisobutylaluminum hydride, NBS *N*-Bromosuccinimide, *m*-CPBA *m*-Chloroperbenzoic acid, THF tetrahydrofuran, DCM dichloromethane.

To demonstrate the practicability of our method, a gram-scale synthesis of the spirocyclic compound **3aa** was conducted without loss of the yield and enantioselectivity (Fig. 5a). The olefin group provided a versatile handle for rapid diversification to afford highly functionalized spirocyclic compounds **6**−**10** with up to three contiguous stereocenters (Fig. 5b). The amide group could be reduced by DIBAL-H to deliver valuable spiro-pyrazolidine **5**. The N–N bond of **3ma** could be cleaved with SmI_2_ to afford functionalized cyclopentene **11** without loss of the enantioselectivity^55^.

### Mechanistic studies

**Fig. 6:**
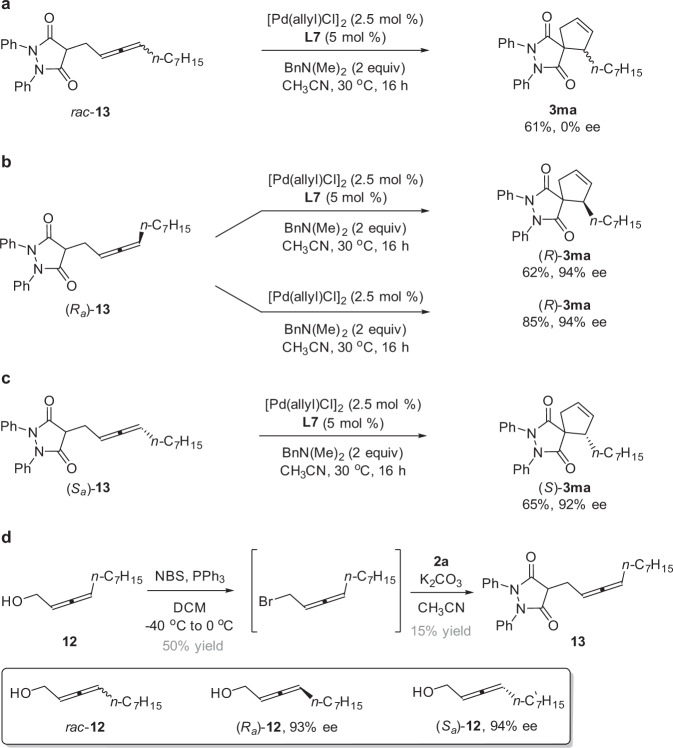
Mechanistic experiments. **a** The cyclization of *rac-***13** led to the spirocyclic product **3ma** with 0% ee. **b** The cyclization of (*R*_*a*_)-**13** afforded (*R*)-**3ma** in 62% yield with 94% ee. **c** The cyclization of (*S*_*a*_)-**13** afforded (*S*)-**3ma** in 65% yield with 92% ee. **d** The preparation of the allene intermediate **13** from allenyl alcohol **12**. NBS *N*-Bromosuccinimide.

In order to gain insight into the reaction mechanism and understand the origin of the high enantioselectivity we observed in the Pd/WingPhos-catalyzed enantioselective cycloaddition reaction of 1,3-enynes **1**, a series of experiments have been conducted (Fig. 6). We prepared the allene intermediates *rac-***13**, (*R*_*a*_)-**13**, and (*S*_*a*_)-**13** from pre-functionalized allenylic partners, *rac-***12**, (*R*_*a*_)-**12** (93% ee), and (*S*_*a*_)-**12** (94% ee), respectively (We could not isolate the corresponding allene intermediate during the Pd/WingPhos-catalyzed cycloaddition reaction of **1a** and **2a**), and subjected them to the conditions of the enantioselective catalytic reaction. The cyclization of *rac*-**13** led to the spirocyclic product **3ma** with 0% ee (Fig. 6a), whereas the cyclization of (*R*_*a*_)-**13** afforded (*R*)-**3ma** in 62% yield with 94% ee (Fig. 6b) and the cyclization of (*S*_*a*_)-**13** afforded (*S*)-**3ma** in 65% yield and 92% ee (Fig. 6c). Taken together, these results suggest that axial chirality of the allene intermediates in situ generated via the Pd/WingPhos-catalyzed enantioselective intermolecular hydroalkylation of 1,3-enynes **1** with cyclic enols/enolates likely serves as a chiral relay during the cyclization process involving a very high efficiency of axial-to central chirality transfer. In addition, the axially chiral allenes in situ generated must be stable to racemization under the reaction conditions; otherwise no chirality transfer could be obtained. We also found that the allene intermediates (*R*_*a*_)-**13** in the absence of ligand provided (*R*)-**3ma** in much higher yield (85% yield) with the same enantioselectivity (94% ee) (Fig. 6b).

**Fig. 7:**
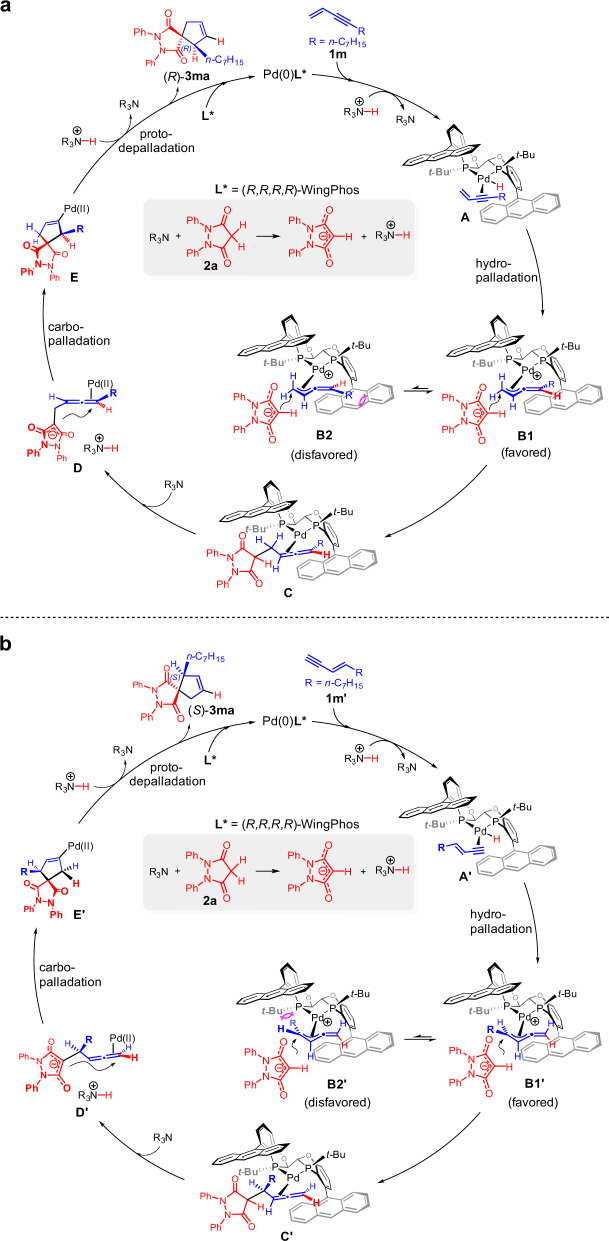
Proposed catalytic cycle. **a** The cycloaddition reaction with 1,3-enyne **1m** having a terminal double bond. **b** The cycloaddition reaction with 1,3-enyne **1m′** having a terminal triple bond.

On the basis of the above mechanistic studies and previous reports^36^, a plausible catalytic cycle for the cycloaddition reaction of 1,3-enynes is proposed in Fig. 7. The cycloaddition reaction of 1,3-enyne **1m** having a terminal double bond involves the terminal Pd-butadienyl intermediate (Fig. 7a). First, the PdH species likely coordinates to 1,3-enyne **1m** to form the complex **A** which undergoes migratory alkyne insertion^36^ to produce the terminal butadienyl−Pd **B1** or **B2**. Due to severe steric repulsion in **B2**, **B1** is favored to undergo the intermolecular nucleophilic attack to afford chiral 1,3-disubstituted allene intermediate **C** with axial chirality. Subsequently, the intramolecular carbopalladation^56^ of the chiral allene intermediate **D** forms **E** through a very high efficiency of axial-to central chirality transfer. The protodepalladation of **E** produces the spirocyclic product (*R*)-**3ma**. For the cycloaddition reaction of 1,3-enyne **1m′** having a terminal triple bond which involves the internal Pd-butadienyl intermediate (Fig. 7b), the intermolecular hydroalkylation of 1,3-enyne **1m′** serves as the enantiodetermining step in which mono-substituted allene intermediate **C′** with central chirality is formed.

**Fig. 8:**
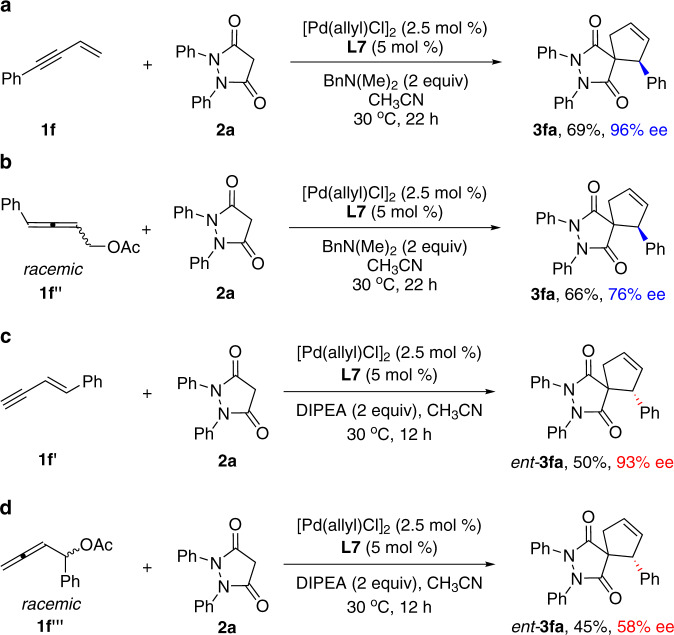
Comparison experiments. **a** The cycloaddition of enyne **1f** via the terminal Pd-butadienyl intermediate generated by PdH catalysis. **b** The cycloaddition of 2,3-allenyl acetate **1f″** via the terminal Pd-butadienyl intermediate generated by oxidative addition. **c** The cycloaddition of enyne **1f′** via the internal Pd-butadienyl intermediate generated by PdH catalysis. **d** The cycloaddition of 2,3-allenyl acetate **1f‴** via the internal Pd-butadienyl intermediate generated by oxidative addition. DIPEA *N,N*-diisopropylethylamine.

Finally, we were interested in seeing whether the Pd-butadienyl intermediates generated by PdH insertion of 1,3-enynes differ with the corresponding intermediates generated by oxidative addition of allenol derivatives in reactivity or selectivity. We made the following comparison experiments (Fig. 8). Interestingly, the achiral enyne **1f** as the precursor provided the cycloaddition product **3fa** in 69% yield with 96% ee (Fig. 8a) whereas the racemic 2,3-allenyl acetate **1f″** as the precursor afforded the product **3fa** in 66% yield with only 76% ee (Fig. 8b) under the identical reaction conditions. On the other hand, the achiral enyne **1f′** as the precursor provided the cycloaddition product *ent-***3fa** in 50% yield with 93% ee (Fig. 8c) whereas the racemic 2,3-allenyl acetate **1f‴** as the precursor afforded the product *ent-***3fa** in 45% yield with only 58% ee (Fig. 8d) under the identical reaction conditions. These results show the advantage and unique of the use of nonpolarized 1,3-enynes in the PdH-catalyzed asymmetric cycloaddition reactions in terms of not only atom economy but also enantioselectivity.

## Discussion

We have developed a PdH-based cycloaddition strategy for the enantioselective synthesis of a series of spirocyclic compounds directly from two classes of commonly available starting materials, 1,3-enynes and activated cyclic carbon–hydrogen (C–H) bonds. In the present atom economic cycloaddition, nonpolarized 1,3-enynes are utilized as dielectrophilic four-carbon units. By employing P-chiral WingPhos as the chiral ligand, the challenging product selectivity issue of cycloaddition products versus double intermolecular hydroalkylation products has been addressed. Notably, a chiral Pd/WingPhos catalyst affects the enantioswitchable enyne cycloaddition reactions with high levels of stereocontrol, thus providing a protocol for the enantioselectivity switch by exchanging the position of double bond and triple bond of 1,3-enyne substrates while maintaining the same absolute configuration of the chiral catalyst^57–59^. A variety of diverse cyclic nucleophiles including pharmaceutically important heterocycles and carbocycles can be flexibly and directly incorporated with spiro scaffolds. A broad range of functional groups of 1,3-enyne substrates, including alcohols, esters, nitriles, halides, and olefins, are tolerated. We believe this methodology may find considerable use and enable the discovery of chiral spirocyclic molecules with interesting biological activities.

## Methods

### Representative procedure for the cycloaddition

[Pd(allyl)Cl]_2_ (0.91 mg, 2.5 mol%), and **L7** (3.7 mg, 5 mol%) were dissolved in CH_3_CN (0.5 mL) and stirred for 15 min at 30 °C under Ar atmosphere. Subsequently, 1,3-enyne **1a** (0.12 mmol, 1.2 equiv), pyrazolidine-3,5-dione **2a** (0.1 mmol, 1 equiv), and BnN(Me)_2_ (0.2 mmol, 2 equiv) were added. The reaction mixture was stirred until the reaction completed. The solution was concentrated in vacuum and the crude product was purified by column chromatography on silica gel (*n-*hexane/EtOAc = 95:5) to afford the spiro-pyrazolidine-3,5-dione **3aa**.

## Supplementary information

Supplementary Information

## Data Availability

The authors declare that the data supporting the findings of this study are available within the article and the Supplementary Information as well as from the authors upon reasonable request. The X-ray crystallographic coordinates for structures (*S*)-**3ah**, (*S*)-**3aj**, and (*S*)-**3ea** reported in this study have been deposited at the Cambridge Crystallographic Data Centre (CCDC), under CCDC 2086798, CCDC 2086799, and CCDC 2086800, respectively. These data can be obtained free of charge from The Cambridge Crystallographic Data Centre via www.ccdc.cam.ac.uk/data_request/cif.
