# Supplementary material for: Direct access to spirocycles by Pd/WingPhos-catalyzed enantioselective cycloaddition of 1,3-enynes
Source: Nat Commun. 2021 Sep 27;12:5667. doi: 10.1038/s41467-021-25981-x (PMC8476582; doi:10.1038/s41467-021-25981-x)
Supplement: Supplementary file 1 — Supplementary Information [file 41467_2021_25981_MOESM1_ESM.pdf]

# Supplementary Information

# Direct access to spirocycles by Pd/WingPhos-catalyzed enantioselective cycloaddition of 1,3-enynes

Long Li[1,4], Shan Wang[1,4], Pengfei Luo[1], Ran Wang[1], Zheng Wang[2], Xiaoguang Li[3], Yuhua Deng[1], Fangzhi Peng[1] & Zhihui Shao[1]*

[1]Key Laboratory of Medicinal Chemistry for Natural Resource, Ministry of Education, School of Chemical Science and Technology, Yunnan Provincial Center for Research & Development of Natural Products, and State Key Laboratory for Conservation and Utilization of Bio-Resources in Yunnan, Yunnan University, Kunming 650091, China

[2]Kunming Institute of Physics, Kunming 650223, China

[3]Institute for Advanced Study, Shenzhen University, Shenzhen 518060, China

[4]These authors contributed equally: Long Li, Shan Wang

email: zhihui_shao@hotmail.com

# Table of Contents


1. Supplementary Methods ............................................................................... 3

 1.1 General Information .............................................................................. 3

 1.2 Synthesis of Starting Materials ............................................................. 3

 1.3 Reaction Optimization........................................................................... 5

 1.4 General Procedures for Asymmetric [4+1] Cycloaddition with Enynes............. 7

 1.5 Synthesis of The Double Hydroalkylation Product **4aa**.................................... 64

 1.6 Gram-scale Reaction ............................................................................ 64

 1.7 Synthetic Transformations .................................................................... 65

2. Supplementary Discussion........................................................................... 75

 2.1 Control Experiments ............................................................................. 75

 2.2 Proposed Reaction Mechanism ............................................................. 81

3. Supplementary Data.................................................................................... 81

 3.1 X-Ray Diffraction Data.......................................................................... 81

 3.2 $^{1}$H and $^{13}$C NMR Spectra for New Compounds ................................................. 86

4. Supplementary References......................................................................... 131

# 1. Supplementary Methods

## 1.1 General Information

Unless otherwise noted, all reactions in standard conditions were carried out under an argon atmosphere. Solvents were dried by standard methods under argon atmosphere. $^{1}$H NMR, $^{13}$C NMR, $^{19}$F NMR and $^{31}$P NMR spectra were recorded on 400 MHz, 500 MHz, 600 MHz instruments using $CDCl_3$ as solvent. Chemical shifts of $^{1}$H NMR were recorded in parts per million (ppm, $\delta$) relative to tetramethylsilane ($\delta$ = 0.00 ppm) with the solvent resonance as an internal standard ($CDCl_3$: $\delta$ = 7.26 ppm). NMR multiplicities are abbreviated as follows: s = singlet, d = doublet, t = triplet, sept = septet, m = multiple, br = broad signal. High-resolution mass spectral analysis (HRMS) data were measured on a spectrometer by means of the ESI technique. The enantiomeric excess was determined by chiral HPLC with *n*-hexane and *i*-propanol as eluents. Optical rotations were measured on a polarimeter. Column chromatography was performed on silica gel (200–300 mesh).

## 1.2 Synthesis of Starting Materials

### 1.2.1 Synthesis of the conjugated enynes 1′ [1]

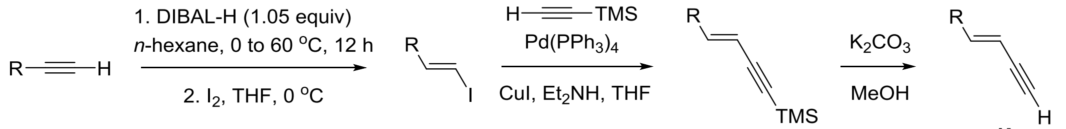

**1'**

Terminal alkyne (40 mmol) was stirred in hexane (40 mL) at room temperature in 200 mL two-necked flask under the atmosphere of argon. Diisobutylaluminum hydride (DIBAL-H, 1.0 M in *n*-hexane, 42 mmol, 42 mL) was added to the solution at 0 ℃. The mixture was stirred at 0 °C to room temperature for 2 h and then heated to 60 ℃ for 10 h. When the starting material was consumed completely with the monitoring of TLC (*n*-hexane), the mixture was added the solution of THF (30 mL) with $I_2$ (20 g) at 0 °C and stirred at room temperature for 6 h. The reaction mixture

was quenched with water at 0 °C. The aqueous mixture was extracted with *n*-hexane. The organic layer was dried over $Na_2SO_4$ and concentrated. The residue was purified with silica gel column chromatography (*n*-hexane).

$Pd(PPh_3)_4$ (2 mol %, 0.668 mmol, 771.9 mg) and CuI (2 mol %, 0.668 mmol, 127.2 mg) were placed in a 100 mL two-necked flask under the atmosphere of argon. To the flask was added $Et_2NH$ (30 mL) and then iodine compounds (33.4 mmol) in THF (30 mL) via syringe. Then trimethylsily acetylene (36.7 mmol, 5.2 mL) was added. After stirring for 17 h, the reaction mixture was monitored with TLC (*n*-hexane). The mixture was poured into water at 0 ℃. The aqueous mixture was extracted with $Et_2O$. The organic layer was washed with 2 M aq. HCl and then washed with saturated aq. $NaHCO_3$. The organic layer was dried over $Na_2SO_4$ and concentrated. The residue was purified with silica gel column (*n*-hexane) and then distilled.

Finally, to a solution of above enynes in methanol (20 mL), $K_2CO_3$ (3 equiv) was added. The reaction mixture was stirred for 5 h at room temperature and quenched with saturated aq. $NH_4Cl$. The aqueous phase was extracted with $Et_2O$ (3 x 20 mL), and the combined organic phases were washed with brine (50 mL), dried over $MgSO_4$, and concentrated under reduced pressure. The residue was purified with silica gel column (*n*-hexane) and then distilled to give enynes **1′**.

### 1.2.2 Synthesis of the conjugated enynes 1[2]

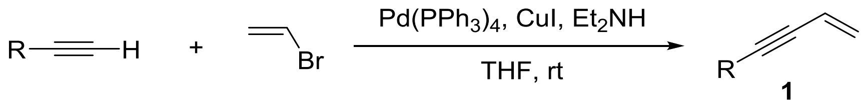


Copper (I) iodide (152.4 mg, 0.8 mmol) and $Pd(PPh_3)_4$ (231.1 mg, 0.2 mmol) were dissolved in diethylamine (20 mL, 0.5 mL/1.0 mmol alkyne) under an Ar atmosphere which was then cooled to 0 ℃. Terminal alkyne (40 mmol, 1 equiv) and vinyl bromide (52 mL, 52 mmol, 1.3 equiv, 1.0 M in THF) were added and the resulting mixture was left to stir and warmed up to room temperature until complete conversion of the starting material was observed from TLC. The reaction mixture was washed with water followed by extraction with *n*-pentane:diethyl ether (1:1). The combined organic layers were washed with 1 M HCl and dried over magnesium

sulfate. The crude product was afforded after evaporation of the solvent in vacuo and ready to be purified by column chromatography to afford enynes **1**.

## 1.3 Reaction Optimization

**Supplementary Table 1.** Chiral ligand screening and reaction optimization[a]

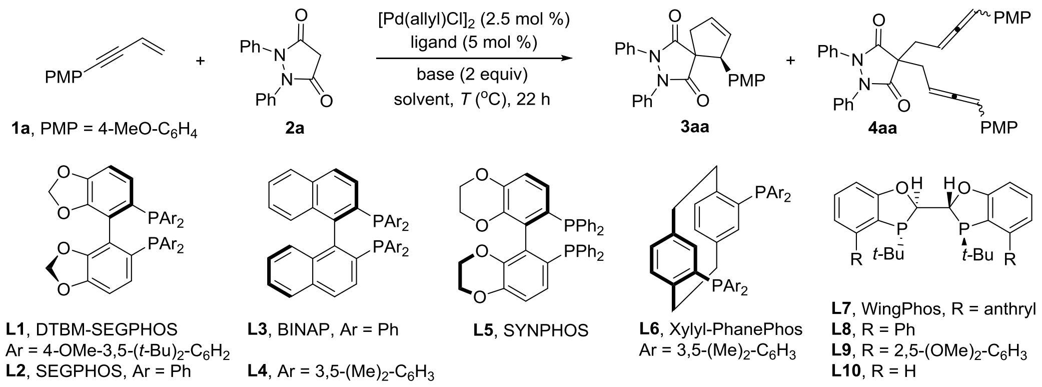


| entry | solvent | base | ligand | *T* (°C) | yield (%) | | ee (%) of **3aa** |
|---|---|---|---|---|---|---|---|
| | | | | | **3aa** | **4aa** | |
| 1 | $CH_3CN$ | $Et_3N$ | **L1** | 50 | 0 | 0 | - |
| 2 | $CH_3CN$ | $Et_3N$ | **L2** | 50 | 0 | 30 | - |
| 3 | $CH_3CN$ | $Et_3N$ | **L3** | 50 | 0 | 79 | - |
| 4 | $CH_3CN$ | $Et_3N$ | **L4** | 50 | 0 | 31 | - |
| 5 | $CH_3CN$ | $Et_3N$ | **L5** | 50 | 10 | 16 | - |
| 6 | $CH_3CN$ | $Et_3N$ | **L6** | 50 | 44 | 16 | 21 |
| 7 | $CH_3CN$ | $Et_3N$ | **L7** | 50 | 37 | 7 | 90 |
| 8 | $CH_3CN$ | $Et_3N$ | **L7** | 30 | 22 | 0 | 96 |
| 9 | $CH_3CN$ | $Et_3N$ | **L8** | 30 | trace | 0 | - |
| 10 | $CH_3CN$ | $Et_3N$ | **L9** | 30 | 16 | 0 | 69 |
| 11 | $CH_3CN$ | $Et_3N$ | **L10** | 30 | 45 | 0 | 44 |
| 12 | $CH_3CN$ | DIPEA | **L7** | 30 | trace | 0 | - |

| | | | | | | | |
|---|---|---|---|---|---|---|---|
| 13 | $CH_3CN$ | $K_2CO_3$ | **L7** | 30 | trace | 0 | - |
| 14 | $CH_3CN$ | $BnN(Me)_2$ | **L7** | 30 | 71 | 0 | 90 |
| 15 | $CH_3CN$ | $BnN(Et)_2$ | **L7** | 30 | 55 | 0 | 90 |
| 16 | $CH_3CN$ | BnN(Me)(Et) | **L7** | 30 | 60 | 0 | 90 |
| 17 | $CH_3CN$ | $Bn_2NMe$ | **L7** | 30 | 68 | 0 | 52 |
| 18 | toluene | $BnN(Me)_2$ | **L7** | 30 | trace | 0 | - |
| 19 | THF | $BnN(Me)_2$ | **L7** | 30 | trace | 0 | - |
| 20 | $CH_2Cl_2$ | $BnN(Me)_2$ | **L7** | 30 | 24 | 0 | 69 |
| 21 | MeOH | $BnN(Me)_2$ | **L7** | 30 | 12 | 0 | 65 |
| 22 | EtOAc | $BnN(Me)_2$ | **L7** | 30 | trace | 0 | - |

[a] Reactions were performed with **1a** (0.12 mmol), **2a** (0.1 mmol), $[Pd(allyl)Cl]_2$ (2.5 mol %), ligand (5 mol %), base (2 equiv) in $CH_3CN$ (0.5 mL) for 22 h. Isolated yields. Ee determined by chiral HPLC. *PMP* 4-MeO-$C_6H_4$, *DIPEA* *N*,*N*-diisopropylethylamine.

**Supplementary Table 2**. Effect of reaction concentration

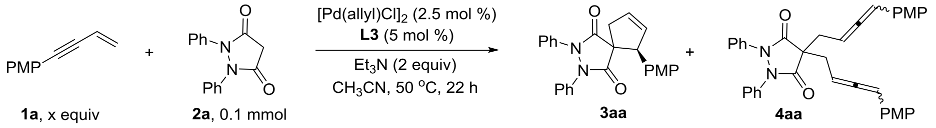


| entry | x | yield (%) of **3aa** | yield (%) of **4aa** |
|---|---|---|---|
| 1 | 0.5 | 0 | 21 |
| 2 | 1.2 | 0 | 79 |

**Supplementary Table 3.** Effect of reaction concentration under standard conditions

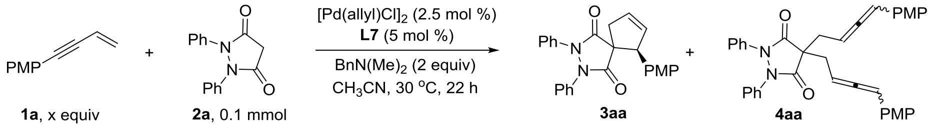


| entry | x | yield (%) of **3aa** | yield (%) of **4aa** |
|---|---|---|---|
| 1 | 1.2 | 71 | 0 |
| 2 | 3 | 66 | 5% |

In order to further explore the effect of reaction concentration on the selectivity between product **3aa** and **4aa**, a series of control experiments have been conducted. If the amount of **1a** was 0.5 equiv, the reaction under standard conditions conducive to the production of product **4aa** still afforded double hydroalkylation product **4aa**, albeit in a low yield (Supplementary Table 2, entry 1). If the amount of **1a** was 3 equiv, the reaction under standard conditions conducive to the production of product **3aa** still provided the desired spirocyclic product **3aa** in 66% yield, and double hydroalkylation product **4aa** was detected in 5% yield (Supplementary Table 3, entry 2).

## 1.4 General Procedures for Asymmetric [4+1] Cycloaddition with Enynes

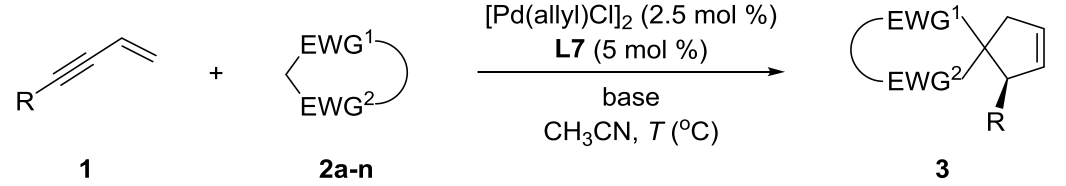


***General Procedures I:*** In an Ar-filled glovebox, dissolving the $[Pd(allyl)Cl]_2$ (0.91 mg, 2.5 mol %), and **L7** (3.7 mg, 5 mol %) in $CH_3CN$ (0.5 mL, 0.2 M) was stirred for 15 min at room temperature. Subsequently, conjugated enyne **1** (0.12 mmol, 1.2 equiv), **2a-m** (0.1 mmol, 1 equiv), and base were added. The reaction mixture was stirred outside the glove box. The solution was concentrated in vacuo and the crude product was purified by column chromatography on silica gel (*n*-hexane:EtOAc = 95:5) to afford the chiral spiro product **3**.

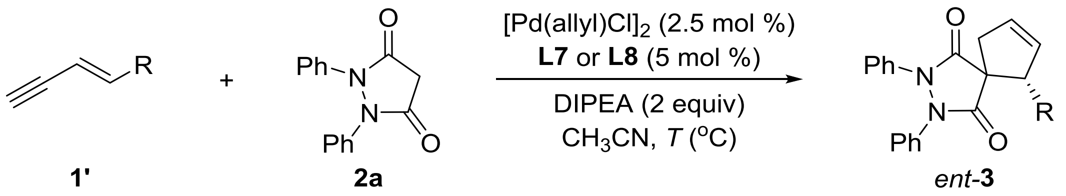

***General Procedures II***: Under an Ar atmosphere, dissolving the $[Pd(allyl)Cl]_2$ (0.91 mg, 2.5 mol %), and **L7** (3.7 mg, 5 mol %) or **L8** (2.7 mg, 5 mol %) in $CH_3CN$ (0.5 mL, 0.2 M) was stirred for 15 min at room temperature. Subsequently, conjugated enyne **1′** (0.12 mmol, 1.2 equiv), **2a** (0.1 mmol, 1 equiv), and DIPEA (2 equiv, 32 μL) were added. The reaction mixture was stirred. The solution was concentrated in vacuo and the crude product was purified by column chromatography on silica gel (*n*-hexane:EtOAc = 95:5) to afford the chiral spiro product *ent*-**3**.

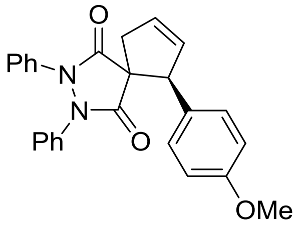


**3aa**

**(*S*)-6-(4-methoxyphenyl)-2,3-diphenyl-2,3-diazaspiro[4.4]non-7-ene-1,4-dione (3aa).** Following the *General Procedure I*: $BnN(Me)_2$ (2 equiv), 30 °C, 22 h. **3aa** was obtained as a yellow oil (29.1 mg, 71% yield, 90% ee). $[\alpha]_D^{20}$ = -39.6 (*c* 1.0, $CHCl_3$). **$^1$H NMR** (400 MHz, $CDCl_3$, ppm): $\delta$ 7.32-7.25 (m, 2H), 7.19-7.13 (m, 7H), 7.05 (t, *J* = 7.3 Hz, 1H), 6.87 (d, *J* = 7.8 Hz, 2H), 6.80 (d, *J* = 8.6 Hz, 2H), 6.08-5.96 (m, 1H), 5.83-5.71 (m, 1H), 4.76-4.65 (m, 1H), 3.78 (s, 3H), 3.19-2.96 (m, 2H). **$^{13}$C NMR** (100 MHz, $CDCl_3$, ppm): $\delta$ 174.0, 171.4, 159.5, 135.7, 135.4, 130.9, 130.2, 130.0, 129.3, 128.9, 128.7, 126.8, 126.5, 122.7, 122.6, 113.8, 63.1, 59.7, 55.4, 40.4. **HPLC analysis:** Daicel CHIRALPAK AD-H, *n*-hexane:*i*-PrOH = 80:20, flow rate = 1.0 mL/min, λ = 254 nm, retention time: $t_{major}$ = 10.7 min, $t_{minor}$ = 16.5 min. **HRMS** (ESI) calcd for $C_{26}H_{23}N_2O_3$ $[M + H]^+$: 411.1703, found 411.1705.

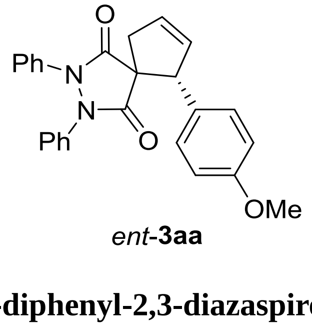


*ent*-**3aa**

**(*R*)-6-(4-methoxyphenyl)-2,3-diphenyl-2,3-diazaspiro[4.4]non-7-ene-1,4-dione**

**(*ent*-3aa).** Following the *General Procedure II*: DIPEA (2 equiv), 30 °C, 12 h. *ent*-**3aa** was obtained as a yellow oil (25.4 mg, 62% yield, 86% ee). **$[\alpha]_D^{20}$** = +34.5 (*c* 1.0, $CHCl_3$). **HPLC analysis:** Daicel CHIRALPAK AD-H, *n*-hexane:*i*-PrOH = 80:20, flow rate = 1.0 mL/min, λ = 254 nm, retention time: $t_{minor}$ = 10.7 min, $t_{major}$ = 16.4 min. **HRMS** (ESI) calcd for $C_{26}H_{23}N_2O_3$ $[M + H]^+$: 411.1703, found 411.1703.

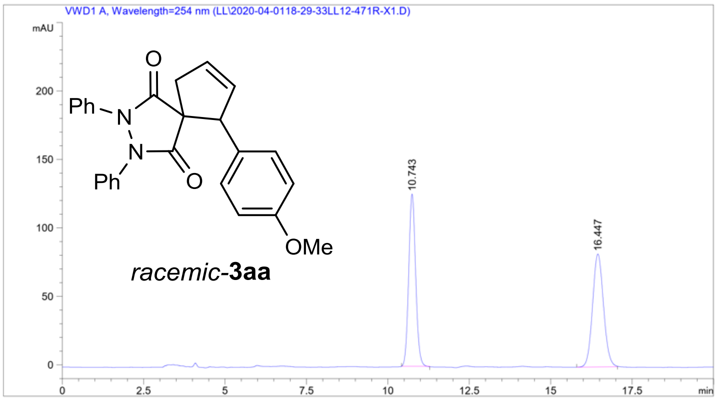


| Peak # | Ret. Time [min] | Type | Width [min] | Area [mAU*s] | Height [mAU] | Area % |
|---|---|---|---|---|---|---|
| 1 | 10.743 | HHAS | 0.2281 | 1855.06421 | 125.74533 | 49.0860 |
| 2 | 16.447 | HHA | 0.3624 | 1924.14746 | 82.40914 | 50.9140 |

**Supplementary Figure 1.** HPLC spectrum of *racemic* **3aa**

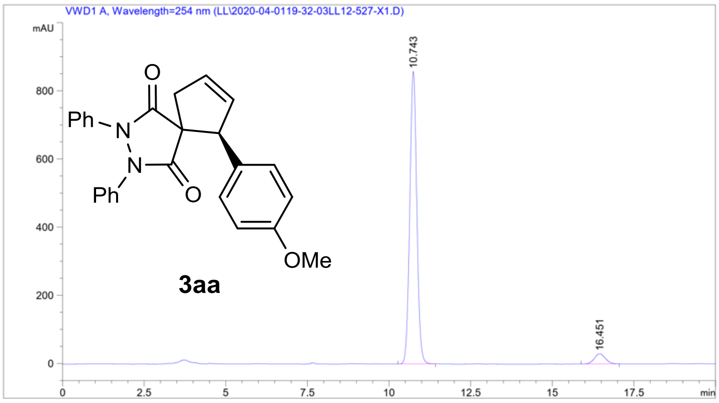


| Peak # | Ret. Time [min] | Type | Width [min] | Area [mAU*s] | Height [mAU] | Area % |
|---|---|---|---|---|---|---|
| 1 | 10.743 | BBA | 0.2311 | 1.28006e4 | 857.83228 | 94.8123 |
| 2 | 16.451 | BBA | 0.3649 | 700.38641 | 29.72833 | 5.1877 |

**Supplementary Figure 2.** HPLC spectrum of **3aa**

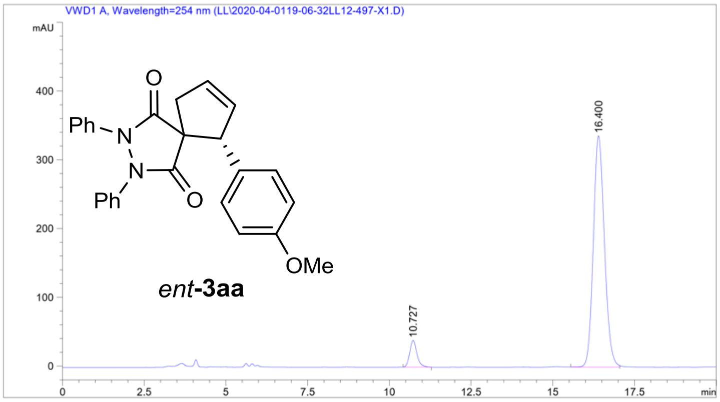


| Peak # | Ret. Time [min] | Type | Width [min] | Area [mAU*s] | Height [mAU] | Area % |
|---|---|---|---|---|---|---|
| 1 | 10.727 | HHAS | 0.2342 | 591.75360 | 38.74349 | 6.9322 |
| 2 | 16.400 | HHA | 0.3645 | 7944.49707 | 336.40921 | 93.0678 |

**Supplementary Figure 3.** HPLC spectrum of *ent*-**3aa**

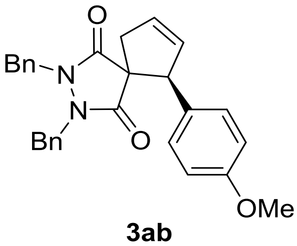


**3ab**

**(*S*)-2,3-dibenzyl-6-(4-methoxyphenyl)-2,3-diazaspiro[4.4]non-7-ene-1,4-dione (3ab).** Following the *General Procedure I*: $BnN(Me)_2$ (2 equiv), 30 $^{o}$C, 36 h. **3ab** was obtained as a yellow oil (26.3 mg, 60% yield, 90% ee). $[\alpha]_D^{20}$ = -18.5 (*c* 1.0, $CHCl_3$). **$^1$H NMR** (400 MHz, $CDCl_3$, ppm): $\delta$ 7.34-7.28 (m, 3H), 7.25-7.15 (m, 3H), 7.14-7.02 (m, 4H), 6.85-6.76 (m, 2H), 6.63 (d, *J* = 7.2 Hz, 2H), 6.01-5.92 (m, 1H), 5.85-5.74 (m, 1H), 4.76 (d, *J* = 16.1 Hz, 1H), 4.65-4.45 (m, 3H), 4.25 (d, *J* = 16.1 Hz, 1H), 3.81 (s, 3H), 3.07-2.87 (m, 2H). **$^{13}$C NMR** (100 MHz, $CDCl_3$, ppm): $\delta$ 174.0, 171.6, 159.3, 135.3, 135.1, 132.0, 129.4, 129.3, 129.0, 128.8, 128.1, 127.8, 127.3, 113.8, 60.5, 58.1, 55.3, 47.1, 46.5, 41.5. **HPLC analysis:** Daicel CHIRALPAK AD-H, *n*-hexane:*i*-PrOH = 80:20, flow rate = 1.0 mL/min, $\lambda$ = 254 nm, retention time: $t_{major}$ = 15.9 min, $t_{minor}$ = 27.9 min. **HRMS** (ESI) calcd for $C_{28}H_{26}N_2O_3Na$ [M + Na]$^+$: 461.1839, found 461.1836.

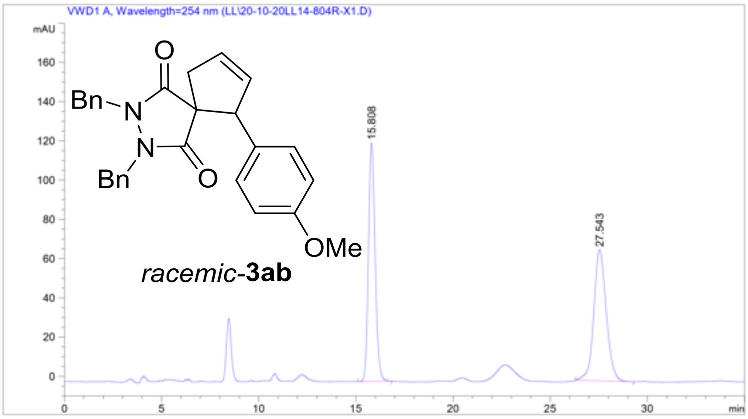


| Peak # | Ret. Time [min] | Type | Width [min] | Area [mAU*s] | Height [mAU] | Area % |
|---|---|---|---|---|---|---|
| 1 | 15.808 | BB | 0.3663 | 2881.22852 | 121.66881 | 49.2416 |
| 2 | 27.543 | HB | 0.6863 | 2969.98340 | 66.72820 | 50.7584 |

**Supplementary Figure 4.** HPLC spectrum of *racemic* **3ab**

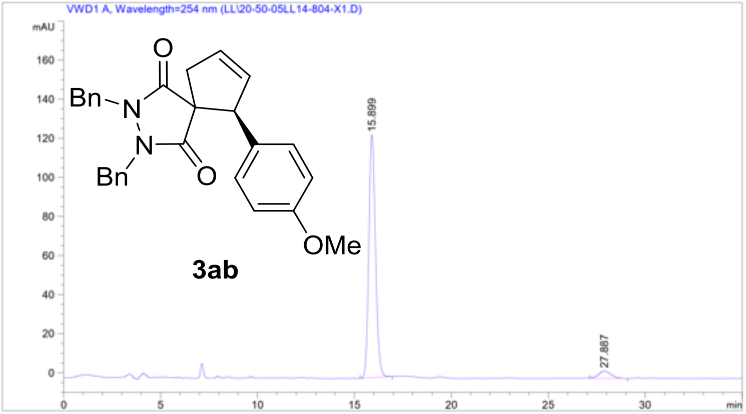


| Peak # | Ret. Time [min] | Type | Width [min] | Area [mAU*s] | Height [mAU] | Area % |
|---|---|---|---|---|---|---|
| 1 | 15.899 | BB | 0.3694 | 2965.02490 | 124.26405 | 94.7031 |
| 2 | 27.887 | HB | 0.6553 | 165.84015 | 3.74715 | 5.2969 |

**Supplementary Figure 5.** HPLC spectrum of **3ab**

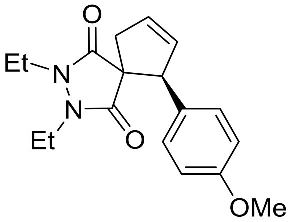

**3ac**

**(*S*)-2,3-diethyl-6-(4-methoxyphenyl)-2,3-diazaspiro[4.4]non-7-ene-1,4-dione (3ac).**

Following the *General Procedure I*: $BnN(Me)_2$ (2 equiv), 30 °C, 48 h. **3ac** was obtained as a yellow oil (26 mg, 83% yield, 90% ee). $[\alpha]_D^{20}$ = -16.0 (*c* 1.0, $CHCl_3$). **$^1$H NMR** (400 MHz, $CDCl_3$, ppm): $\delta$ 7.11 (d, *J* = 8.3 Hz, 2H), 6.78 (d, *J* = 8.5 Hz, 2H), 5.95-5.86 (m, 1H), 5.79-5.70 (m, 1H), 4.54 (s, 1H), 3.75 (s, 3H), 3.70-3.52 (m, 2H), 3.31 (q, *J* = 7.3 Hz, 2H), 2.86 (q, *J* = 7.3 Hz, 2H), 2.96-2.76 (m, 2H), 1.08 (t, *J* = 7.2 Hz, 3H), 0.70 (t, *J* = 7.3 Hz, 3H). **$^{13}$C NMR** (100 MHz, $CDCl_3$, ppm): $\delta$ 175.0, 172.8, 159.2, 131.9, 130.1, 129.8, 129.3, 113.6, 60.4, 58.1, 55.3, 41.1, 37.9, 37.5, 12.3, 11.6. **HPLC analysis:** Daicel CHIRALPAK AD-H, *n*-hexane:*i*-PrOH = 80:20, flow rate = 1.0 mL/min, λ = 254 nm, retention time: $t_{major}$ = 7.4 min, $t_{minor}$ = 8.3 min. **HRMS** (ESI) calcd for $C_{18}H_{23}N_2O_3$ $[M + H]^+$: 315.1702, found 315.1703.

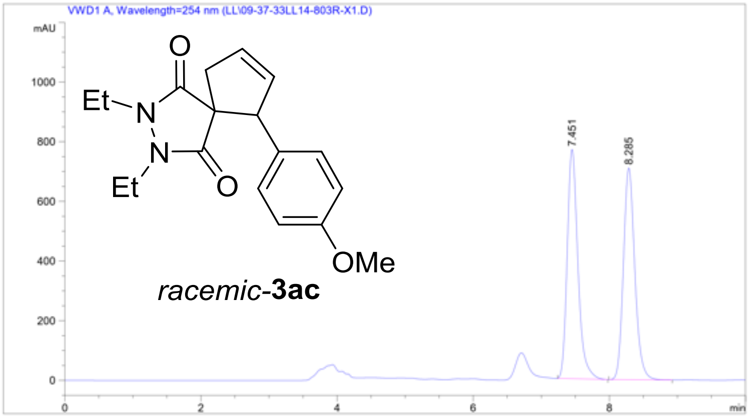


| Peak # | Ret. Time [min] | Type | Width [min] | Area [mAU*s] | Height [mAU] | Area % |
|---|---|---|---|---|---|---|
| 1 | 7.451 | HB | 0.1672 | 8376.25293 | 768.24365 | 50.3707 |
| 2 | 8.285 | HB | 0.1786 | 8252.96289 | 709.91333 | 49.6293 |

**Supplementary Figure 6.** HPLC spectrum of *racemic* **3ac**

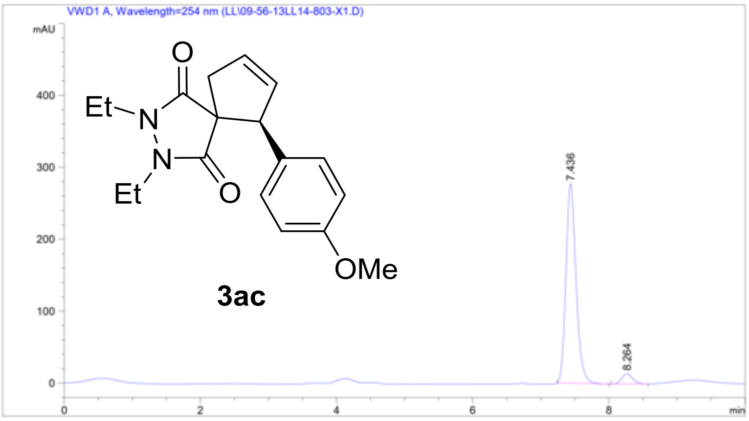


| Peak # | Ret. Time [min] | Type | Width [min] | Area [mAU*s] | Height [mAU] | Area % |
|---|---|---|---|---|---|---|
| 1 | 7.436 | HB | 0.1581 | 2854.13745 | 277.22601 | 94.8747 |
| 2 | 8.264 | BH | 0.1682 | 154.18614 | 14.14663 | 5.1253 |

**Supplementary Figure 7.** HPLC spectrum of **3ac**

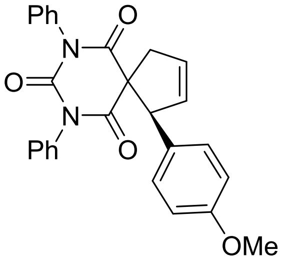


**3ad**

**(*S*)-1-(4-methoxyphenyl)-7,9-diphenyl-7,9-diazaspiro[4.5]dec-2-ene-6,8,10-trione (3ad)** Following the *General Procedure I*: $BnN(Me)_2$ (2 equiv), 40 $^oC$, 12 h, **3ad** was obtained as a yellow solid (43.5 mg, 99% yield, 87% ee). $[\alpha]_D^{20}$ = -19.8 (*c* 1.0, $CHCl_3$). **$^1$H NMR** (400 MHz, $CDCl_3$, ppm): $\delta$ 7.51 (t, *J* = 7.2 Hz, 2H), 7.44 (t, *J* = 7.2 Hz, 1H), 7.33 (d, *J* = 7.5 Hz, 2H), 7.29-7.17 (m, 5H), 6.98-6.89 (m, 2H), 6.75-6.09 (m, 2H), 6.08-6.00 (m, 1H), 5.56-5.48 (m,1H), 4.75-4.67 (m, 1H), 3.82 (s, 3H), 3.50-3.30 (m, 2H). **$^{13}$C NMR** (100 MHz, $CDCl_3$, ppm): $\delta$ 172.1, 168.6, 160.2, 150.6, 134.7, 134.0, 131.8, 130.6, 129.5, 129.3, 129.2, 128.8, 128.8, 128.4, 128.3, 128.1, 114.6, 67.1, 63.3, 55.6, 40.1. **HPLC analysis:** Daicel CHIRALPAK AD-H, *n*-hexane:*i*-PrOH = 80:20, flow rate = 1.0 mL/min, $\lambda$ = 254 nm, retention time: $t_{minor}$ = 10.4 min, $t_{major}$ = 12.2 min. **HRMS** (ESI) calcd for $C_{27}H_{23}N_2O_4$ $[M + H]^+$: 439.1652, found 439.1650.

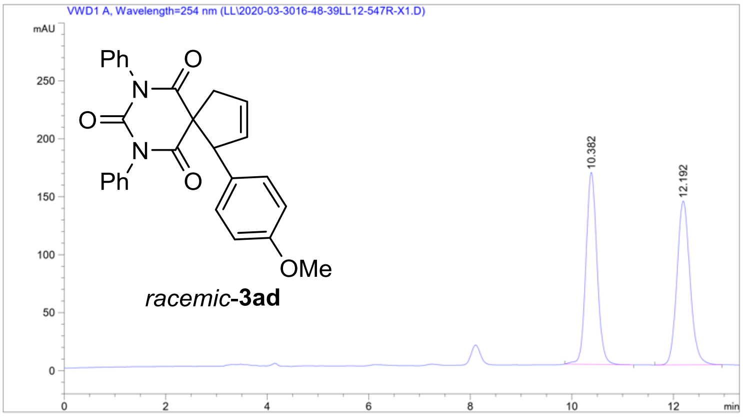


| Peak # | Ret. Time [min] | Type | Width [min] | Area [mAU*s] | Height [mAU] | Area % |
|---|---|---|---|---|---|---|
| 1 | 10.382 | HB | 0.2368 | 2506.95020 | 165.47351 | 49.8003 |
| 2 | 12.192 | BHA | 0.2768 | 2527.05884 | 141.22336 | 50.1997 |

**Supplementary Figure 8.** HPLC spectrum of *racemic* **3ad**

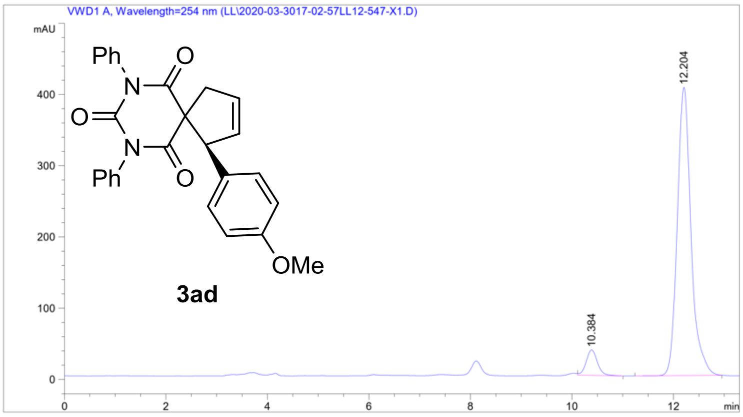


| Peak # | Ret. Time [min] | Type | Width [min] | Area [mAU*s] | Height [mAU] | Area % |
|---|---|---|---|---|---|---|
| 1 | 10.384 | HB | 0.2344 | 547.63336 | 35.81727 | 6.7201 |
| 2 | 12.204 | BHA | 0.2833 | 7601.56250 | 404.33301 | 93.2799 |

**Supplementary Figure 9.** HPLC spectrum of **3ad**

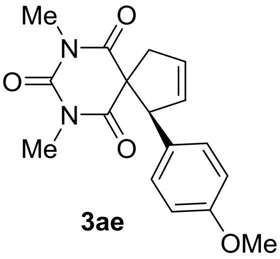


**3ae**

**(*S*)-1-(4-methoxyphenyl)-7,9-dimethyl-7,9-diazaspiro[4.5]dec-2-ene-6,8,10-trione (3ae).** Following the *General Procedure I*: BnN(Me)$_2$ (2 equiv), 40 $^o$C, 12 h, **3ea** was obtained as a yellow oil (31.0 mg, 99% yield, 90% ee). $[\alpha]_D^{20}$ = -13.9 (*c* 1.0, $CHCl_3$).

**¹H NMR** (400 MHz, $CDCl_3$, ppm): $\delta$ 6.92 (d, $J$ = 8.6 Hz, 2H), 6.79 (d, $J$ = 8.6 Hz, 2H), 6.11-5.99 (m, 1H), 5.52-5.43 (m, 1H), 4.39-4.30 (m, 1H), 3.77 (s, 3H), 3.37 (s, 3H), 3.34-3.15 (m, 2H), 2.57 (s, 3H). **¹³C NMR** (100 MHz, $CDCl_3$, ppm): $\delta$ 172.2, 169.3, 159.8, 151.3, 132.2, 129.6, 129.1, 128.0, 113.9, 66.4, 63.3, 55.4, 38.9, 29.2, 28.3. **HPLC analysis:** Daicel CHIRALPAK AD-H, *n*-hexane:*i*-PrOH = 80:20, flow rate = 1.0 mL/min, λ = 254 nm, retention time: $t_{major}$ = 8.3 min, $t_{minor}$ = 12.0 min. **HRMS** (ESI) calcd for $C_{17}H_{18}N_2O_4$ $[M + H]^+$: 315.1339, found 315.1340.

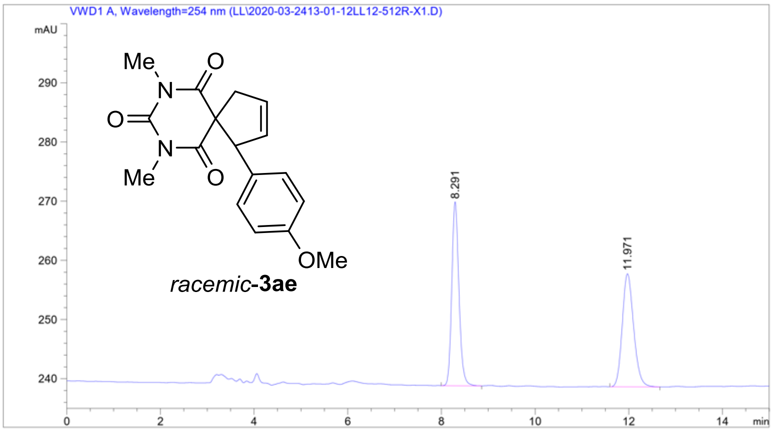


| Peak # | Ret. Time [min] | Type | Width [min] | Area [mAU*s] | Height [mAU] | Area % |
|---|---|---|---|---|---|---|
| 1 | 8.291 | BB | 0.1628 | 329.52402 | 31.06344 | 50.5684 |
| 2 | 11.971 | BB | 0.2588 | 322.11655 | 19.09355 | 49.4316 |

**Supplementary Figure 10.** HPLC spectrum of *racemic* **3ae**

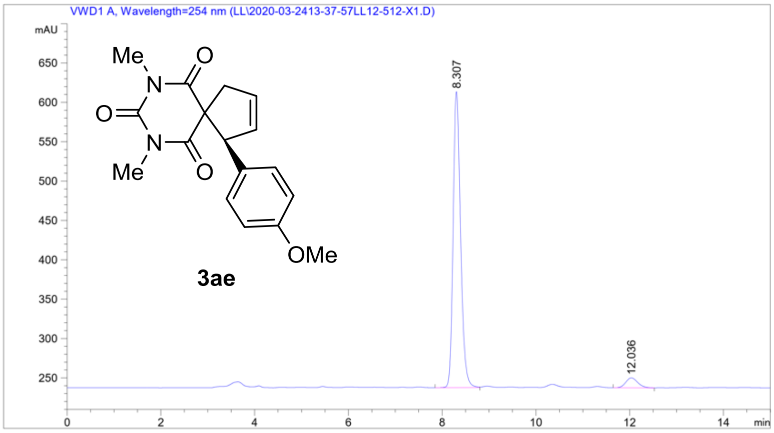


| Peak # | Ret. Time [min] | Type | Width [min] | Area [mAU*s] | Height [mAU] | Area % |
|---|---|---|---|---|---|---|
| 1 | 8.307 | BV | 0.1686 | 4145.61182 | 376.17639 | 94.9330 |
| 2 | 12.036 | BB | 0.2728 | 221.27162 | 12.60195 | 5.0670 |

**Supplementary Figure 11.** HPLC spectrum of **3ae**

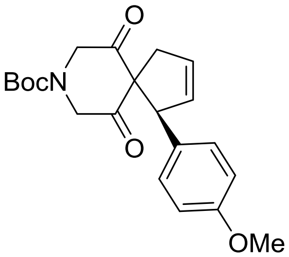


**3af**

***Tert*-butyl-(*S*)-1-(4-methoxyphenyl)-6,10-dioxo-8-azaspiro[4.5]dec-2-ene-8-carboxylate (3af).** Following the *General Procedure I*: $BnN(Me)_2$ (2 equiv), 30 $^oC$, 22 h. **3af** was obtained as a yellow oil (20.5 mg, 55%, 87% ee). **$[\alpha]_D^{20}$** = -32.7 (*c* 1.0, $CHCl_3$). **$^1$H NMR** (400 MHz, $CDCl_3$, ppm): $\delta$ 7.07-6.87 (m, 2H), 6.79 (d, $J$ = 8.7 Hz, 2H), 6.02-5.91 (m, 1H), 5.38-5.30 (m, 1H), 5.15 (s, 1H), 4.19 (s, 1H), 3.96-3.71 (m, 4H), 3.63 (d,1H), 3.36 (d, 1H), 3.32-3.23 (m, 1H), 2.91-2.78 (m, 1H), 1.62-1.44 (m, 9H). **$^{13}$C NMR** (100 MHz, $CDCl_3$, ppm): $\delta$ 203.0, 202.0, 159.5, 130.7, 130.0, 129.1, 114.1, 81.9, 77.5, 77.4, 76.8, 76.7, 59.8, 55.4, 31.8, 28.5. **HPLC analysis:** Daicel CHIRALPAK ID, *n*-hexane:*i*-PrOH = 90:10, flow rate = 1.0 mL/min, λ = 254 nm, retention time: $t_{major}$ = 15.1 min, $t_{minor}$ = 20.1 min. **HRMS** (ESI) calcd for $C_{21}H_{25}NO_5Na$ [M + Na]$^+$: 3394.1625, found394.1622.

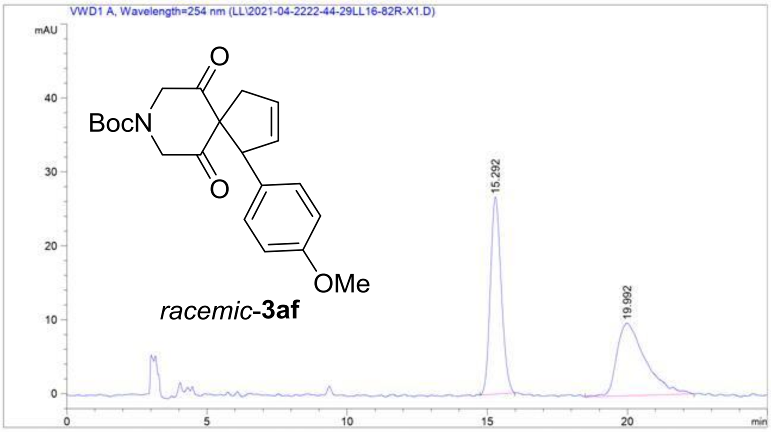


| Peak # | Ret. Time [min] | Type | Width [min] | Area [mAU*s] | Height [mAU] | Area % |
|---|---|---|---|---|---|---|
| 1 | 15.292 | BBA | 0.4164 | 718.07373 | 26.64608 | 50.7930 |
| 2 | 19.992 | BV R | 0.9669 | 695.65106 | 9.81361 | 49.2070 |

**Supplementary Figure 12.** HPLC spectrum of *racemic* **3af**

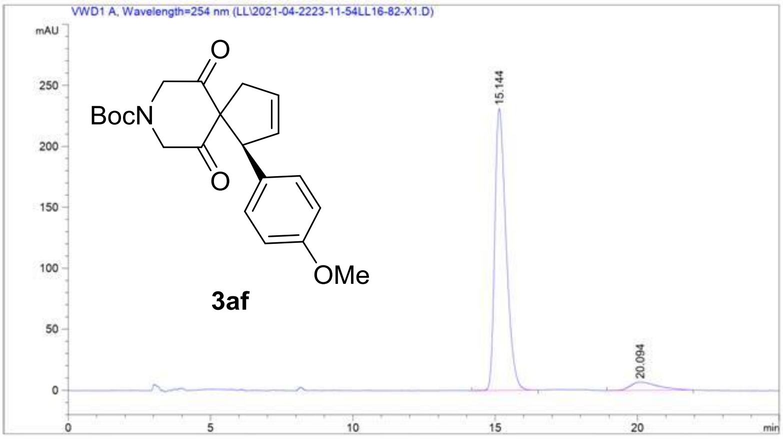


| Peak # | Ret. Time [min] | Type | Width [min] | Area [mAU*s] | Height [mAU] | Area % |
|---|---|---|---|---|---|---|
| 1 | 15.144 | VB R | 0.4238 | 6382.62207 | 231.19374 | 93.2714 |
| 2 | 20.094 | VB R | 0.9720 | 460.44061 | 6.79961 | 6.7286 |

**Supplementary Figure 13.** HPLC spectrum of **3af**

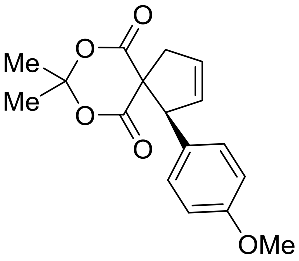


**3ag**

**(*S*)-1-(4-methoxyphenyl)-8,8-dimethyl-7,9-dioxaspiro[4.5]dec-2-ene-6,10-dione (3ag).** Following the *General Procedure I*: $BnN(Me)_2$ (4 equiv), 40 °C, 36 h. **3ag** was obtained as a yellow solid (20.2 mg, 67% yield, 88% ee). $[\alpha]_D^{20}$ = -17.9 (*c* 1.0, $CHCl_3$). **$^1$H NMR** (400 MHz, $CDCl_3$, ppm): $\delta$ 7.10 (d, *J* = 8.6 Hz, 2H), 6.83 (d, *J* = 8.6 Hz, 2H), 6.06-5.92 (m, 1H), 5.77-5.61 (m, 1H), 4.79-4.67 (m, 1H), 3.78 (s, 3H), 3.26-3.16 (m, 2H), 1.59 (s, 3H), 1.53 (s, 3H). **$^{13}$C NMR** (100 MHz, $CDCl_3$, ppm): $\delta$ 172.2, 167.7, 159.7, 130.5, 130.3, 129.9, 128.8, 113.8, 105.0, 66.0, 60.1, 55.3, 42.8, 30.5, 27.9. **HPLC analysis:** Daicel CHIRALPAK AD-H, *n*-hexane:*i*-PrOH = 80:20, flow rate = 1.0 mL/min, λ = 254 nm, retention time: $t_{major}$ = 6.8 min, $t_{minor}$ = 7.4 min. **HRMS** (ESI) calcd for $C_{17}H_{18}O_5$ $[M + H]^+$: 321.1227, found 321.1226.

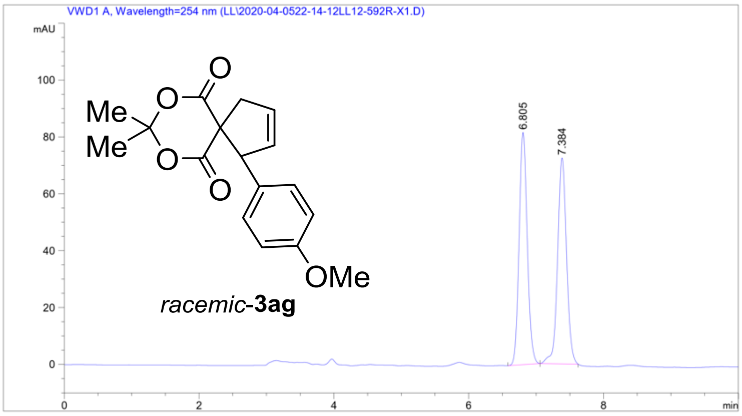


| Peak # | Ret. Time [min] | Type | Width [min] | Area [mAU*s] | Height [mAU] | Area % |
|---|---|---|---|---|---|---|
| 1 | 6.805 | BB | 0.1300 | 685.67621 | 81.58733 | 50.6182 |
| 2 | 7.384 | BBAS | 0.1417 | 668.92816 | 72.43753 | 49.3818 |

**Supplementary Figure 14.** HPLC spectrum of *racemic* **3ag**

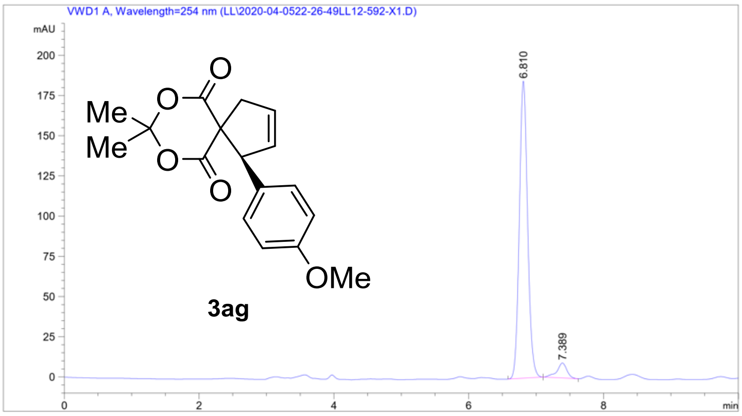


| Peak # | Ret. Time [min] | Type | Width [min] | Area [mAU*s] | Height [mAU] | Area % |
|---|---|---|---|---|---|---|
| 1 | 6.810 | BF | 0.1314 | 1573.01404 | 184.61836 | 94.2646 |
| 2 | 7.389 | BBAS | 0.1542 | 95.70759 | 9.28995 | 5.7354 |

**Supplementary Figure 15.** HPLC spectrum of **3ag**

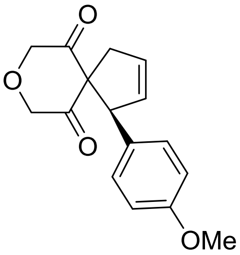


**3ah**

**(*S*)-1-(4-methoxyphenyl)-8-oxaspiro[4.5]dec-2-ene-6,10-dione (3ah).** Following the *General Procedure I*: BnN(Me)$_2$ (2 equiv), 40 $^{o}$C, 22h. **3ah** was obtained as a white solid (20.9 mg, 77% yield, 82% ee). mp 132.6 $^{o}$C. $[\alpha]_D^{20}$ = -11.3 (*c* 1.0, CHCl$_3$). **$^1$H NMR** (400 MHz, CDCl$_3$, ppm): $\delta$ 7.13 (d, *J* = 8.7 Hz, 2H), 6.81 (d, *J* = 8.7 Hz, 2H),

6.04-5.98 (m, 1H), 5.42-5.35 (m, 1H), 4.79 (dd, $J$ = 18.3 Hz, 1.4 Hz, 1H), 4.45-4.39 (m, 1H), 4.01 (d, $J$ = 18.4 Hz, 1H), 3.77 (s, 3H), 3.69 (s, 1H), 3.62-3.52 (m, 1H), 3.31-3.20 (m, 1H), 2.86-2.76 (m, 1H). **$^{13}$C NMR** (100 MHz, $CDCl_3$, ppm): $\delta$ 205.7, 204.9, 159.4, 131.1, 130.5, 129.7, 128.8, 113.9, 76.1, 73.2, 72.4, 60.0, 55.3, 30.9. **HPLC analysis:** Daicel CHIRALPAK IA, *n*-hexane:*i*-PrOH = 90:10, flow rate = 1.0 mL/min, λ = 254 nm, retention time: $t_{major}$ = 12.1 min, $t_{minor}$ = 14.5 min. **HRMS** (ESI) calcd for $C_{16}H_{14}O_4$ $[M + H]^+$: 273.1123, found 273.1121.

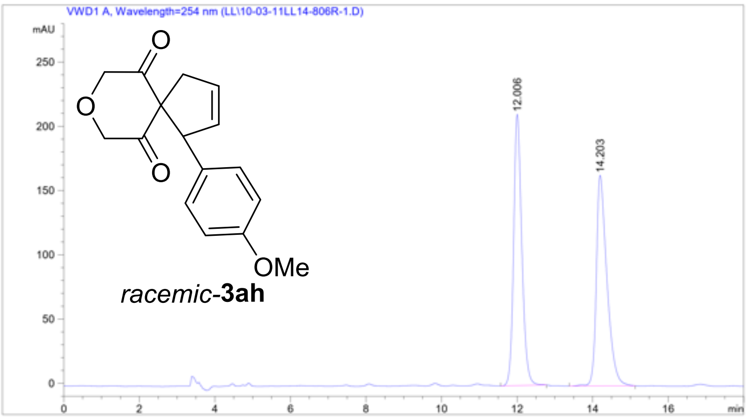


| Peak # | Ret. Time [min] | Type | Width [min] | Area [mAU*s] | Height [mAU] | Area % |
|---|---|---|---|---|---|---|
| 1 | 12.006 | BBA | 0.2314 | 3169.99365 | 210.83328 | 49.8394 |
| 2 | 14.203 | BBA | 0.2916 | 3190.41748 | 163.56157 | 50.1606 |

**Supplementary Figure 16.** HPLC spectrum of *racemic* **3ah**

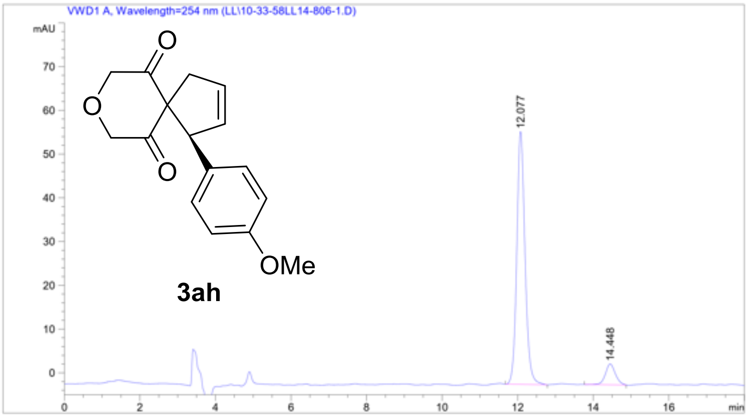


| Peak # | Ret. Time [min] | Type | Width [min] | Area [mAU*s] | Height [mAU] | Area % |
|---|---|---|---|---|---|---|
| 1 | 12.077 | BB | 0.2323 | 872.64227 | 57.74148 | 90.9030 |
| 2 | 14.448 | BBAS | 0.2827 | 87.32905 | 4.74610 | 9.0970 |

**Supplementary Figure 17.** HPLC spectrum of **3ah**

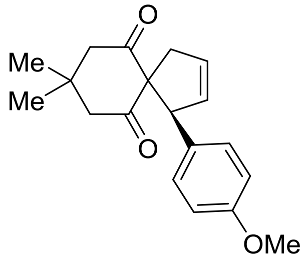

**3ai**

**(*S*)-1-(4-methoxyphenyl)-8,8-dimethylspiro[4.5]dec-2-ene-6,10-dione (3ai).** Following the *General Procedure I*: $BnN(Me)_2$ (2 equiv), 40 $^{o}$C, 22 h. **3ai** was obtained as a yellow solid (29.4 mg, 99% yield, 91% ee). $[\alpha]_D^{20}$ = -26.0 (*c* 1.0, $CHCl_3$). **$^1$H NMR** (400 MHz, $CDCl_3$, ppm): $\delta$ 7.00 (d, *J* = 8.6 Hz, 2H), 6.81 (d, *J* = 8.6 Hz, 2H), 6.03-5.89 (m, 1H), 5.37-5.23 (m, 1H), 4.13-4.03 (m, 1H), 3.77 (s, 3H), 3.26-3.13 (m, 2H), 3.06-2.90 (m, 2H), 2.54-2.41(m, 1H), 2.08-1.96 (m, 1H), 1.82-1.70 (m, 1H), 1.02 (s, 3H), 0.73 (s, 3H). **$^{13}$C NMR** (100 MHz, $CDCl_3$, ppm): $\delta$ 207.3, 206.6, 159.4, 131.4, 130.6, 130.4, 128.7, 114.1, 62.6, 55.3, 51.7, 33.9, 30.8, 30.3, 26.8. **HPLC analysis:** Daicel CHIRALPAK AD-H, *n*-hexane:*i*-PrOH = 90:10, flow rate = 1.0 mL/min, λ = 254 nm, retention time: $t_{minor}$ = 7.1 min, $t_{major}$ = 7.4 min. **HRMS** (ESI) calcd for $C_{19}H_{22}O_3$ $[M + H]^+$: 299.1642, found 299.1643.

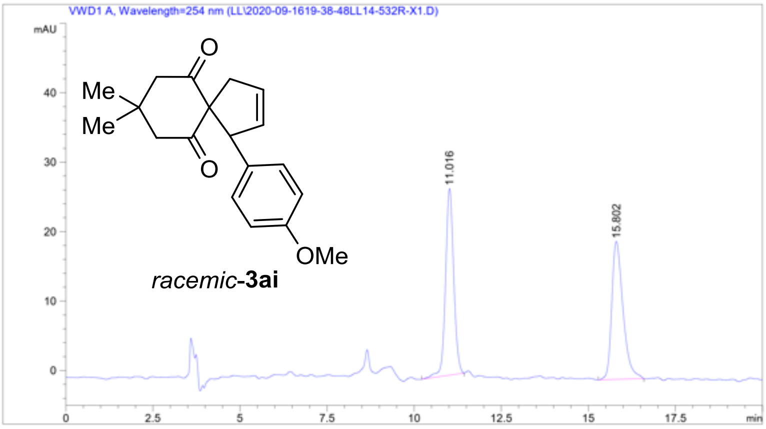


| Peak # | Ret. Time [min] | Type | Width [min] | Area [mAU*s] | Height [mAU] | Area % |
|---|---|---|---|---|---|---|
| 1 | 11.016 | BB | 0.2486 | 439.23160 | 26.87922 | 49.6197 |
| 2 | 15.802 | BBA | 0.3420 | 445.96497 | 19.86099 | 50.3803 |

**Supplementary Figure 18.** HPLC spectrum of *racemic* **3ai**

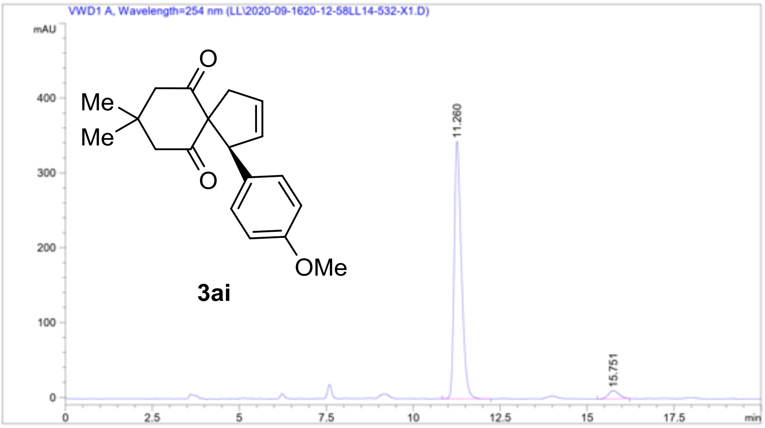


| Peak # | Ret. Time [min] | Type | Width [min] | Area [mAU*s] | Height [mAU] | Area % |
|---|---|---|---|---|---|---|
| 1 | 11.260 | BBA | 0.2179 | 4950.32324 | 343.97665 | 95.5820 |
| 2 | 15.751 | BBA | 0.3258 | 228.81532 | 10.85836 | 4.4180 |

**Supplementary Figure 19.** HPLC spectrum of **3ai**

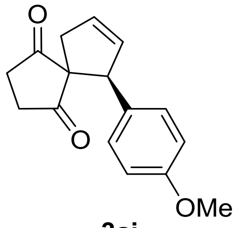


**3aj**

**(*S*)-6-(4-methoxyphenyl)spiro[4.4]non-7-ene-1,4-dione (3aj).** Following the *General Procedure I*: $BnN(Me)_2$ (2 equiv), 30 $^{o}$C, 48 h. **3aj** was obtained as a yellow oil (15.9 mg, 62% yield, 92% ee). mp 159.6 $^{o}$C. $[\alpha]_D^{20}$ = -32.7 (*c* 1.0, $CHCl_3$). $^{1}$**H NMR** (400 MHz, $CDCl_3$, ppm): $\delta$ 7.01 (d, $J$ = 8.7 Hz, 2H), 6.82 (d, $J$ = 8.6 Hz, 2H), 6.00-5.93 (m, 1H), 5.49-5.42 (m, 1H), 4.25-4.19 (m, 1H), 3.09 (s, 3H), 2.93-2.82 (m, 1H), 2.81-2.67 (m, 2H), 2.63-2.49 (m, 1H), 2.36-2.24 (m, 1H), 1.91-1.78 (m, 1H). $^{13}$**C NMR** (100 MHz, $CDCl_3$, ppm): $\delta$ 214.9, 213.1, 159.5, 131.1, 129.9, 129.7, 114.1, 68.8, 63.3, 55.4, 37.2, 35.9, 35.3. **HPLC analysis:** Daicel CHIRALPAK AD-H, *n*-hexane:*i*-PrOH = 90:10, flow rate = 1.0 mL/min, $\lambda$ = 254 nm, retention time: $t_{major}$ = 12.3 min, $t_{minor}$ = 13.4 min. **HRMS** (ESI) calcd for $C_{16}H_{17}O_3$ $[M + H]^+$: 257.1170, found 257.1172.

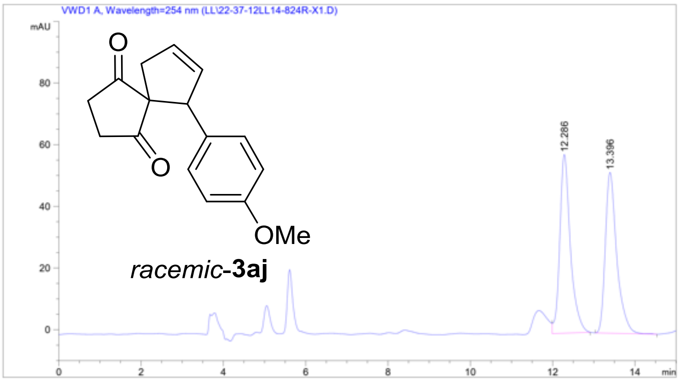


| Peak # | Ret. Time [min] | Type | Width [min] | Area [mAU*s] | Height [mAU] | Area % |
|---|---|---|---|---|---|---|
| 1 | 12.286 | VBA | 0.2688 | 1035.74536 | 57.86587 | 50.6423 |
| 2 | 13.396 | BB | 0.2939 | 1009.47406 | 52.14437 | 49.3577 |

**Supplementary Figure 20.** HPLC spectrum of *racemic* **3aj**

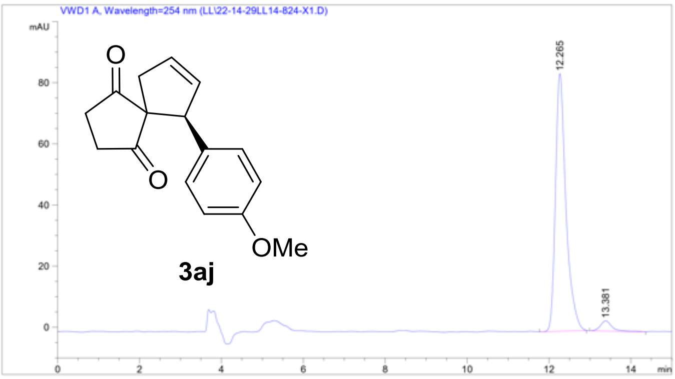


| Peak # | Ret. Time [min] | Type | Width [min] | Area [mAU*s] | Height [mAU] | Area % |
|---|---|---|---|---|---|---|
| 1 | 12.265 | VBAR | 0.2669 | 1492.95239 | 84.16907 | 95.8417 |
| 2 | 13.381 | BB | 0.2912 | 64.77430 | 3.32556 | 4.1583 |

**Supplementary Figure 21.** HPLC spectrum of **3aj**

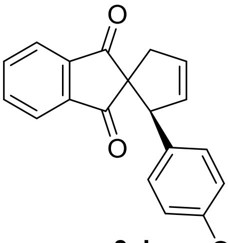

**3ak** OMe

**(*S*)-2-(4-methoxyphenyl)spiro[cyclopentane-1,2'-inden]-3-ene-1',3'-dione (3ak).** Following the *General Procedure I*: $BnN(Me)_2$ (3 equiv), 30 $^{o}$C, 20 h. **3ak** was obtained as a yellow solid (15.2 mg, 50 % yield, 91% ee). **$[\alpha]_D^{20}$** = -39.0 (*c* 1.0, $CHCl_3$). **$^1$H NMR** (400 MHz, $CDCl_3$, ppm): $\delta$ 7.99 (d, $J$ = 7.6 Hz, 1H), 7.77 (t, $J$ = 7.4

Hz, 1H), 7.68 (t, $J$ = 7.5 Hz, 1H), 7.56 (d, $J$ = 7.6 Hz, 1H), 6.77 (d, $J$ = 8.4 Hz, 2H), 6.61 (d, $J$ = 8.4 Hz, 2H), 6.11-6.00 (m, 1H), 5.80-5.67 (m, 1H), 4.77-4.36 (m, 1H), 3.70 (s, 3H), 2.90 (m, 2H). **$^{13}$C NMR** (100 MHz, $CDCl_3$, ppm): $\delta$ 203.5, 200.9, 158.8, 143.0, 142.0, 135.7, 135.2, 131.2, 130.7, 129.8, 129.8, 123.3, 123.2, 113.5, 64.8, 61.7, 55.2, 38.5.**HPLC analysis:** Daicel CHIRALPAK AD-H, *n*-hexane:*i*-PrOH = 90:10, flow rate = 1.0 mL/min, λ = 254 nm, retention time: $t_{major}$ = 12.0 min, $t_{minor}$ = 13.0 min. **HRMS** (ESI) calcd for $C_{20}H_{16}O_3Na$ [M + Na]$^+$: 327.0992, found 327.099.

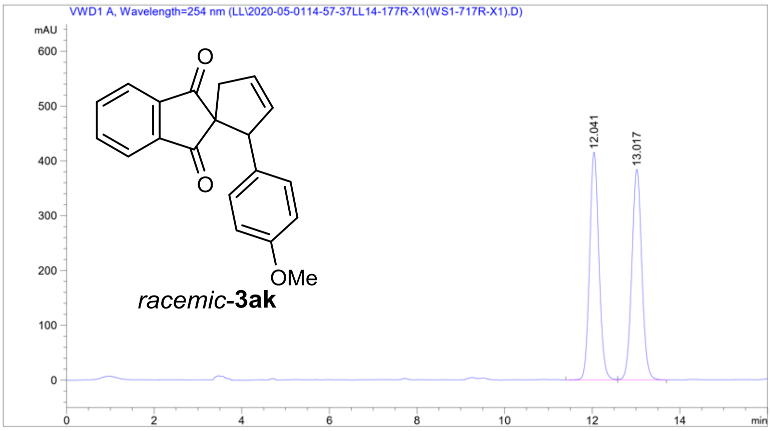


| Peak # | Ret. Time [min] | Type | Width [min] | Area [mAU*s] | Height [mAU] | Area % |
|---|---|---|---|---|---|---|
| 1 | 12.041 | BV | 0.2274 | 6138.56592 | 415.45535 | 50.1603 |
| 2 | 13.017 | VB | 0.2452 | 6099.33838 | 384.24942 | 49.8397 |

**Supplementary Figure 22.** HPLC spectrum of *racemic* **3ak**

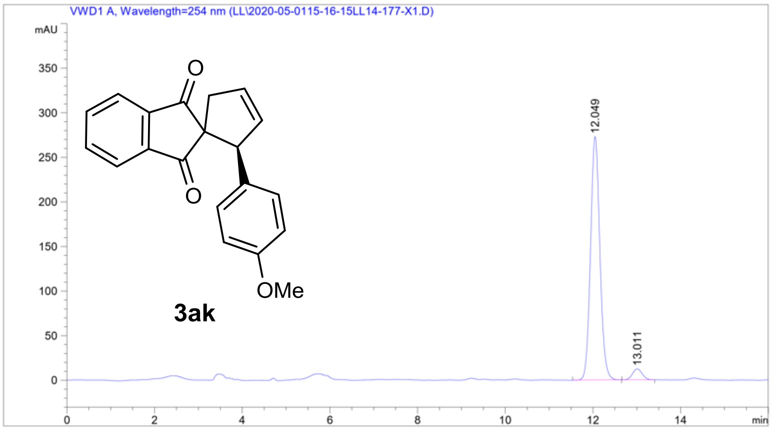


| Peak # | Ret. Time [min] | Type | Width [min] | Area [mAU*s] | Height [mAU] | Area % |
|---|---|---|---|---|---|---|
| 1 | 12.049 | BB | 0.2272 | 4028.50757 | 272.95605 | 95.5566 |
| 2 | 13.011 | BBA | 0.2392 | 187.32568 | 12.26591 | 4.4434 |

**Supplementary Figure 23.** HPLC spectrum of **3ak**

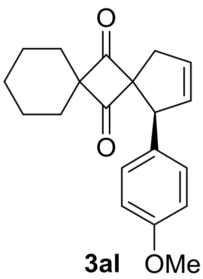


**3al**

**(*S*)-1-(4-methoxyphenyl)dispiro[4.1.57.15]tridec-2-ene-6,13-dione (3al).** Following the *General Procedure I*: $Et_3N$ (2 equiv), 40 °C, 48 h. **3al** was obtained as a white solid (10.1 mg, 65% yield, 86% ee). $[\alpha]_D^{20}$ = -31.9 (*c* 1.0, $CHCl_3$). **$^1$H NMR** (400 MHz, $CDCl_3$, ppm): $\delta$ 7.08 (d, *J* = 8.6 Hz, 2H), 6.82 (d, *J* = 8.6 Hz, 2H), 5.85-5.76 (m, 1H), 5.67-5.56 (m, 1H), 4.48-4.36 (m, 1H), 3.78 (s, 3H), 3.00-2.81 (m, 2H), 1.72-1.21 (m, 12H). **$^{13}$C NMR** (100 MHz, $CDCl_3$, ppm): $\delta$ 215.2, 211.9, 159.1, 132.6, 130.6, 129.7, 128.4, 113.9, 82.7, 76.2, 58.3, 55.3, 40.2, 28.4, 25.2, 24.9, 21.9, 21.8. **HPLC analysis:** Daicel CHIRALPAK AD-H, *n*-hexane:*i*-PrOH = 95:5, flow rate = 1.0 mL/min, $\lambda$ = 254 nm, retention time: $t_{major}$ = 6.4 min, $t_{minor}$ = 7.5 min. **HRMS** (ESI) calcd for $C_{20}H_{22}NO_3Na$ $[M + Na]^+$: 333.1461, found 333.1463.

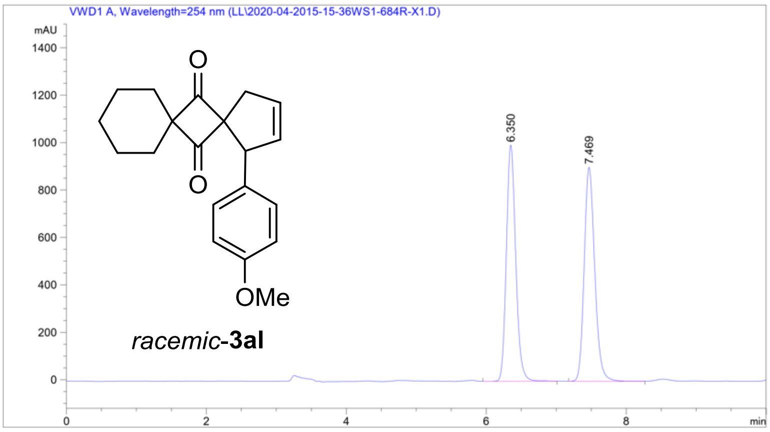


| Peak # | Ret. Time [min] | Type | Width [min] | Area [mAU*s] | Height [mAU] | Area % |
|---|---|---|---|---|---|---|
| 1 | 6.350 | BV R | 0.1434 | 9250.16602 | 995.88647 | 49.4816 |
| 2 | 7.469 | BV R | 0.1611 | 9443.97461 | 902.26569 | 50.5184 |

**Supplementary Figure 24.** HPLC spectrum of *racemic* **3al**

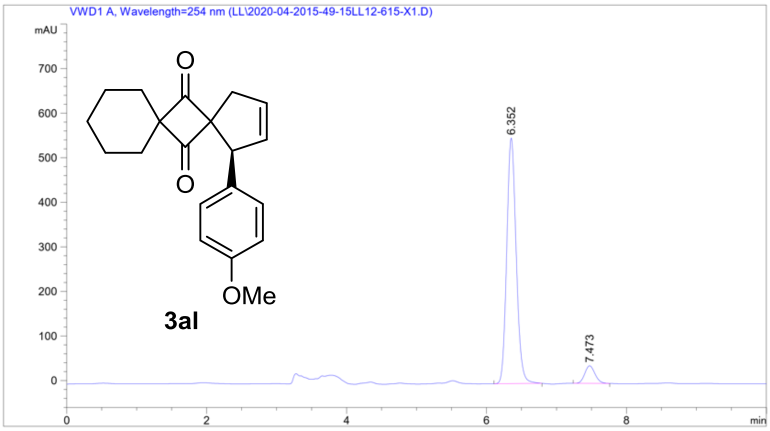


| Peak # | Ret. Time [min] | Type | Width [min] | Area [mAU*s] | Height [mAU] | Area % |
|---|---|---|---|---|---|---|
| 1 | 6.352 | BBA | 0.1452 | 5155.88525 | 550.86548 | 92.7805 |
| 2 | 7.473 | BBA | 0.1573 | 401.19562 | 39.55941 | 7.2195 |

**Supplementary Figure 25.** HPLC spectrum of **3al**

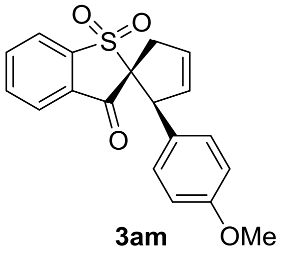


**3am**

**(2*R*,2'*S*)-2'-(4-methoxyphenyl)-3*H*-spiro[benzo[*b*]thiophene-2,1'-cyclopentan]-3'-en-3-one 1,1-dioxide (3am).** Following the *General Procedure I*: BnN(Me)$_2$ (2 equiv), 30 $^{o}$C, 22 h. **3am** was obtained as a white solid (32 mg, 94%, > 20/1 dr, 95% ee). **[α]$_D^{20}$** = -20.8 (*c* 1.0, CHCl$_3$). **$^{1}$H NMR** (600 MHz, CDCl$_3$, ppm): *δ* 7.99 (d, *J* = 7.8 Hz, 1H), 7.82 (t, *J* = 7.6 Hz, 1H), 7.59 (t, *J* = 7.6 Hz, 1H), 7.42 (d, *J* = 7.8 Hz, 1H), 6.79 (d, *J* = 8.5 Hz, 1H), 6.59 (d, *J* = 8.5 Hz, 3H), 6.07-5.97 (m, 1H), 5.73-5.65 (m, 1H), 5.10 (s, 1H), 3.69 (s, 3H), 3.33 (m, 1H), 3.19 (m, 1H). **$^{3}$C NMR** (150 MHz, CDCl$_3$, ppm): *δ* 191.9, 159.2, 145.5, 136.2, 134.0, 133.3, 131.0, 130.2, 130.0, 127.9, 124.3, 121.8, 113.6, 59.3, 55.2, 36.0. **HPLC analysis:** Daicel CHIRALPAK AD-H, *n*-hexane:*i*-PrOH = 80:20, flow rate = 1.0 mL/min, λ = 254 nm, retention time: $t_{major}$ = 10.3 min, $t_{minor}$ = 11.5 min. **HRMS** (ESI) calcd for $C_{19}H_{17}O_4S$ [M + H]$^+$: 341.0803, found 341.0842.

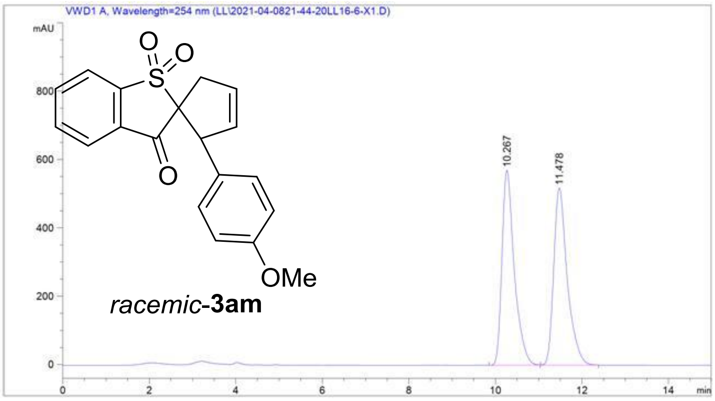


| Peak # | Ret. Time [min] | Type | Width [min] | Area [mAU*s] | Height [mAU] | Area % |
|---|---|---|---|---|---|---|
| 1 | 10.267 | BB | 0.2980 | 1.12388e4 | 570.18164 | 46.9654 |
| 2 | 11.478 | BBA | 0.3254 | 1.12478e4 | 517.91504 | 47.0030 |

**Supplementary Figure 26.** HPLC spectrum of *racemic* **3am**

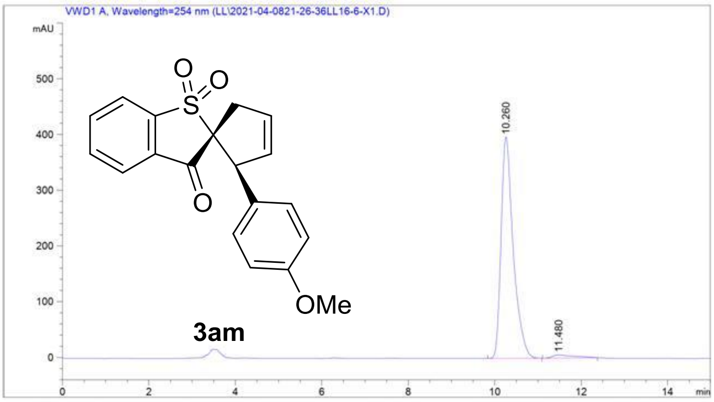


| Peak # | Ret. Time [min] | Type | Width [min] | Area [mAU*s] | Height [mAU] | Area % |
|---|---|---|---|---|---|---|
| 1 | 10.260 | BB | 0.2917 | 7742.97461 | 396.72485 | 97.5791 |
| 2 | 11.480 | BBA | 0.4736 | 192.09911 | 5.58037 | 2.4209 |

**Supplementary Figure 27.** HPLC spectrum of **3am**

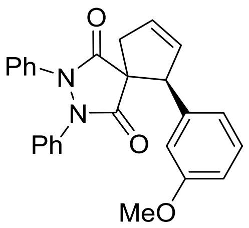

**3ba**

**(*S*)-6-(3-methoxyphenyl)-2,3-diphenyl-2,3-diazaspiro[4.4]non-7-ene-1,4-dione (3ba).** Following the *General Procedure I*: $BnN(Me)_2$ (2 equiv), 30 °C, 22 h. **3ba** was obtained as a white solid (33.1 mg, 63% yield, 94% ee). $[\alpha]_D^{20}$ = -10.0 (*c* 1.0, $CHCl_3$). **$^1$H NMR** (400 MHz, $CDCl_3$, ppm): $\delta$ 7.28 (t, *J* = 7.6 Hz, 2H), 7.23-7.09 (m, 6H), 7.05 (t, *J* = 7.3 Hz, 1H), 6.93-6.78 (m, 4H), 6.74-6.69 (m, 1H), 6.09-5.98 (m, 1H), 5.87-5.77 (m, 1H), 4.76-4.68 (m, 1H), 3.65 (s, 3H), 3.22-2.97 (m, 2H). **$^{13}$C NMR** (100 MHz, $CDCl_3$, ppm): $\delta$ 173.7, 171.2, 159.7, 138.9, 138.8, 135.6, 135.3, 130.6, 130.5, 129.5, 128.9, 128.7, 126.7, 126.5, 122.7, 122.6, 121.1, 114.3, 113.9, 63.6, 59.8, 55.3, 40.5. **HPLC analysis:** Daicel CHIRALPAK AD-H, *n*-hexane:*i*-PrOH = 80:20, flow rate = 1.0 mL/min, λ = 254 nm, retention time: $t_{major}$ = 12.2 min, $t_{minor}$ = 15.2 min. **HRMS** (ESI) calcd for $C_{26}H_{23}N_2O_3$ $[M + H]^+$: 411.1703, found 411.1699.

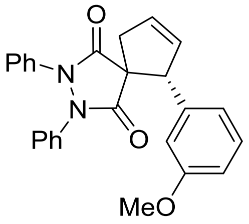

*ent*-**3ba**

**(*R*)-6-(3-methoxyphenyl)-2,3-diphenyl-2,3-diazaspiro[4.4]non-7-ene-1,4-dione (*ent*-3ba).** Following the *General Procedure II*: DIPEA (2 equiv), 30 °C, 12 h. *ent*-**3ba** was obtained as a white solid (27.5 mg, 67% yield, 91% ee). $[\alpha]_D^{20}$ = +15.8 (*c* 1.0, $CHCl_3$). **HPLC analysis:** Daicel CHIRALPAK AD-H, *n*-hexane:*i*-PrOH = 80:20, flow rate = 1.0 mL/min, λ = 254 nm, retention time: $t_{major}$ = 11.8 min, $t_{minor}$ = 15.0 min. **HRMS** (ESI) calcd for $C_{26}H_{23}N_2O_3$ $[M + H]^+$: 411.1703, found 411.1704.

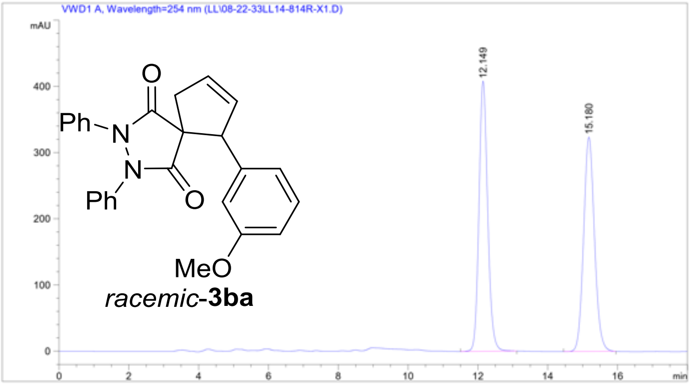


| Peak # | Ret. Time [min] | Type | Width [min] | Area [mAU*s] | Height [mAU] | Area % |
|---|---|---|---|---|---|---|
| 1 | 12.149 | BBA | 0.2663 | 7069.01953 | 407.73709 | 50.2349 |
| 2 | 15.180 | BBA | 0.3365 | 7002.91553 | 323.62399 | 49.7651 |

**Supplementary Figure 28.** HPLC spectrum of *racemic* **3ba**

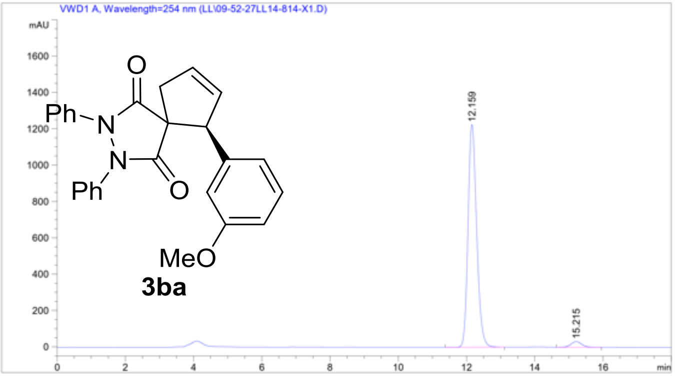


| Peak # | Ret. Time [min] | Type | Width [min] | Area [mAU*s] | Height [mAU] | Area % |
|---|---|---|---|---|---|---|
| 1 | 12.159 | BBA | 0.2687 | 2.15048e4 | 1225.55908 | 96.9250 |
| 2 | 15.215 | BBA | 0.3342 | 682.25940 | 31.31325 | 3.0750 |

**Supplementary Figure 29.** HPLC spectrum of **3ba**

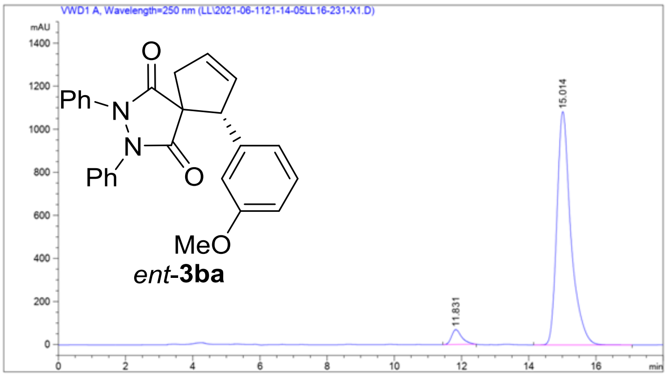


| Peak # | Ret. Time [min] | Type | Width [min] | Area [mAU*s] | Height [mAU] | Area % |
|---|---|---|---|---|---|---|
| 1 | 11.831 | BBA | 0.3167 | 1463.54980 | 69.22139 | 4.4757 |
| 2 | 15.014 | BB | 0.4293 | 3.12361e4 | 1083.74548 | 95.5243 |

**Supplementary Figure 30.** HPLC spectrum of *ent*-**3ba**

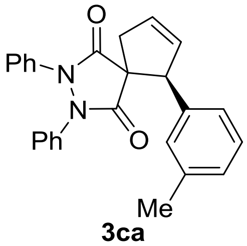

**3ca**

**(*S*)-2,3-diphenyl-6-(m-tolyl)-2,3-diazaspiro[4.4]non-7-ene-1,4-dione (3ca).** Following the *General Procedure I*: BnN(Me)$_2$ (2 equiv), 30 $^{o}$C, 22 h. **3ca** was obtained as a white solid (33.1 mg, 89% yield, 95% ee). **[α]$_D$$^{20}$** = -7.8 (*c* 1.0, CHCl$_3$). **$^1$H NMR** (400 MHz, CDCl$_3$, ppm): $\delta$ 7.30 (t, *J* = 7.8 Hz, 2H), 7.23-7.01 (m, 9H), 6.96 (s, 1H), 6.85 (d, *J* = 7.8 Hz, 2H), 6.06-5.99 (m, 1H), 5.86-5.76 (m, 1H), 4.76-4.66 (m, 1H), 3.22-2.95 (m, 2H), 2.24 (s, 3H). **$^{13}$C NMR** (100 MHz, CDCl$_3$, ppm): $\delta$ 173.9, 171.2, 138.0, 137.2, 135.7, 135.3, 130.6, 130.4, 129.6, 128.9, 128.9, 128.7, 128.5, 126.7, 126.5, 125.9, 122.7, 122.6. **HPLC analysis:** Daicel CHIRALPAK IA, *n*-hexane:*i*-PrOH = 90:10, flow rate = 1.0 mL/min, λ = 254 nm, retention time: $t_{major}$ = 10.1 min, $t_{minor}$ = 11.1 min. **HRMS** (ESI) calcd for $C_{26}H_{23}N_2O_2$ [M + H]$^+$: 395.1753, found 395.1754.

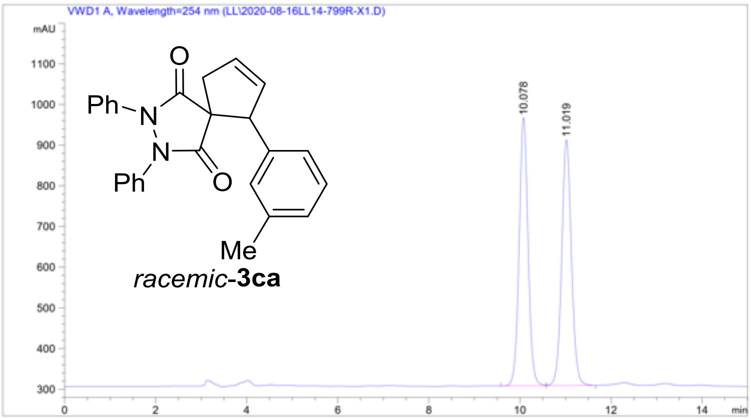


| Peak # | Ret. Time [min] | Type | Width [min] | Area [mAU*s] | Height [mAU] | Area % |
|---|---|---|---|---|---|---|
| 1 | 10.078 | BV | 0.2110 | 8970.33105 | 658.01959 | 49.9627 |
| 2 | 11.019 | VB | 0.2290 | 8947.48145 | 603.56860 | 49.8354 |

**Supplementary Figure 31.** HPLC spectrum of *racemic* **3ca**

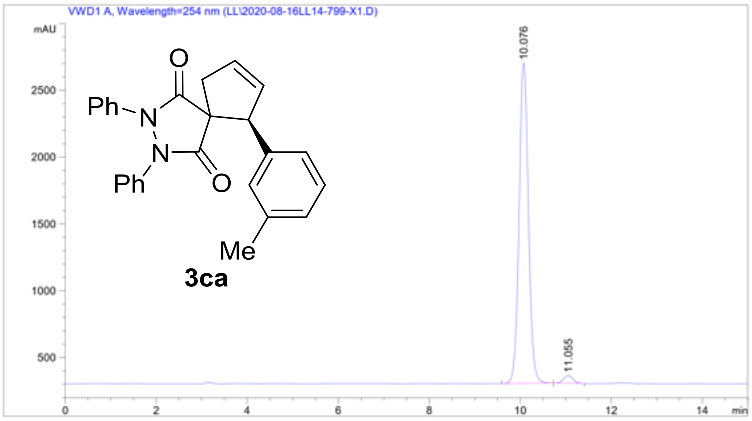


| Peak # | Ret. Time [min] | Type | Width [min] | Area [mAU*s] | Height [mAU] | Area % |
|---|---|---|---|---|---|---|
| 1 | 10.076 | BB | 0.2203 | 3.41709e4 | 2396.57764 | 97.6472 |
| 2 | 11.055 | VBAR | 0.2190 | 823.35687 | 58.23023 | 2.3528 |

**Supplementary Figure 32.** HPLC spectrum of **3ca**

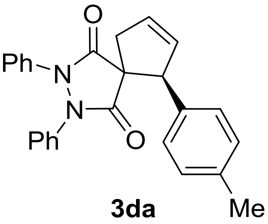


**3da**

**(*S*)-2,3-diphenyl-6-(*p*-tolyl)-2,3-diazaspiro[4.4]non-7-ene-1,4-dione (3da).** Following the *General Procedure I*: $BnN(Me)_2$ (2 equiv), 30 °C, 22 h. **3da** was obtained as a white solid (33.1 mg, 84% yield, 95% ee). $[\alpha]_D^{20}$ = -14.5 (*c* 1.0, $CHCl_3$). $^1$**H NMR** (400 MHz, $CDCl_3$, ppm): $\delta$ 7.30 (t, $J$ = 7.6 Hz, 2H), 7.20-7.01 (m, 10H), 6.88-6.83 (m, 2H), 6.07-5.97 (m, 1H), 5.84-5.75 (m, 1H), 4.77-4.69 (m, 1H), 5.83-5.71 (m, 1H), 4.76-4.65 (m, 1H), 3.22-3.98 (m, 2H), 2.32 (s, 3H). $^{13}$**C NMR** (100 MHz, $CDCl_3$, ppm): $\delta$ 173.9, 171.4, 137.8, 135.7, 135.3, 134.2, 130.7, 130.3, 129.1, 128.9, 128.7, 128.6, 126.8, 126.5, 122.8, 122.6, 63.3, 59.5, 40.5. **HPLC analysis:** Daicel CHIRALPAK AD-H, *n*-hexane:*i*-PrOH = 90:10, flow rate = 1.0 mL/min, λ = 254 nm, retention time: $t_{major}$ = 16.2 min, $t_{minor}$ = 18.0 min. **HRMS** (ESI) calcd for $C_{26}H_{23}N_2O_2$ $[M + H]^+$: 395.1753, found 395.1754.

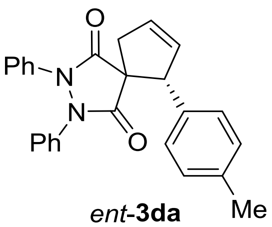

*ent*-**3da**

**(*R*)-2,3-diphenyl-6-(p-tolyl)-2,3-diazaspiro[4.4]non-7-ene-1,4-dione (*ent*-3da).** Following the *General Procedure I*: DIPEA (2 equiv), 30 °C, 12 h. *ent*-**3da** was obtained as a white solid (24 mg, 61% yield, 93% ee). **$[\alpha]_D^{20}$** = +12.8 (*c* 1.0, $CHCl_3$). **HPLC analysis:** Daicel CHIRALPAK AD-H, *n*-hexane:*i*-PrOH = 90:10, flow rate = 1.0 mL/min, λ = 254 nm, retention time: $t_{major}$ = 16.2 min, $t_{minor}$ = 17.6 min. **HRMS** (ESI) calcd for $C_{26}H_{23}N_2O_2$ $[M + H]^+$: 395.1753, found 395.1759.

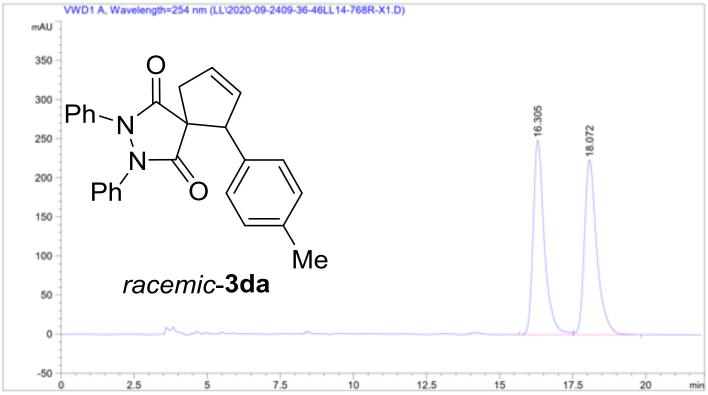


| Peak # | Ret. Time [min] | Type | Width [min] | Area [mAU*s] | Height [mAU] | Area % |
|---|---|---|---|---|---|---|
| 1 | 16.305 | BV | 0.3975 | 6593.41943 | 248.52063 | 50.2057 |
| 2 | 18.072 | VB | 0.4394 | 6539.38818 | 223.46127 | 49.7943 |

**Supplementary Figure 33.** HPLC spectrum of *racemic* **3da**

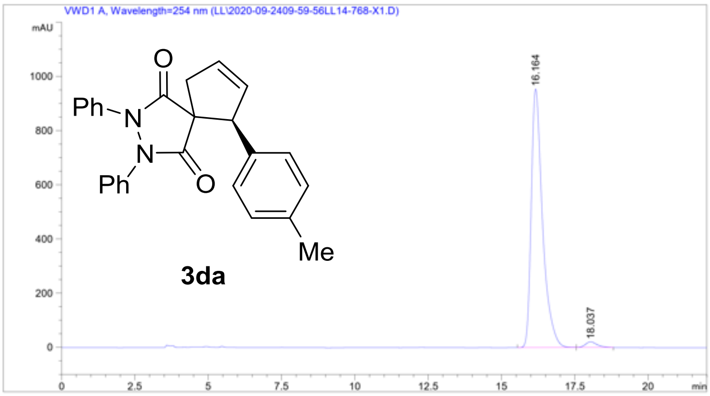


| Peak # | Ret. Time [min] | Type | Width [min] | Area [mAU*s] | Height [mAU] | Area % |
|---|---|---|---|---|---|---|
| 1 | 16.164 | BF | 0.4006 | 2.54812e4 | 953.94537 | 97.8525 |
| 2 | 18.037 | BBA | 0.4155 | 559.23041 | 20.48160 | 2.1475 |

**Supplementary Figure 34.** HPLC spectrum of **3da**

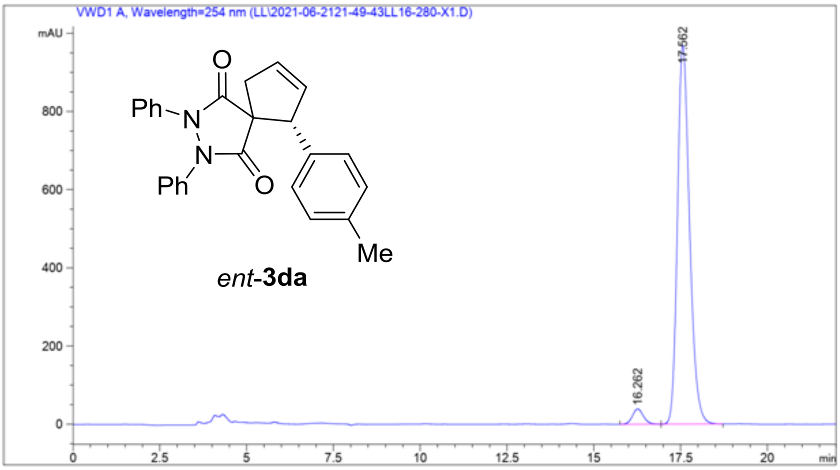


| Peak # | Ret. Time [min] | Type | Width [min] | Area [mAU*s] | Height [mAU] | Area % |
|---|---|---|---|---|---|---|
| 1 | 16.262 | BV | 0.3398 | 858.19165 | 39.14491 | 3.5258 |
| 2 | 17.562 | VB | 0.3726 | 2.34822e4 | 969.54834 | 96.4742 |

**Supplementary Figure 35.** HPLC spectrum of *ent*-**3da**

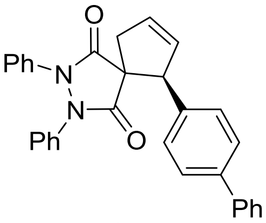


**3ea**

**(*S*)-6-([1,1'-biphenyl]-4-yl)-2,3-diphenyl-2,3-diazaspiro[4.4]non-7-ene-1,4-dione (3ea).** Following the *General Procedure I*: $BnN(Me)_2$ (2 equiv), 30 °C, 22 h. **3ea** was obtained as a white solid (32 mg, 70% yield, 96% ee). mp 163.1 °C. $[\alpha]_D^{20}$ = -21.9 (*c* 1.0, $CHCl_3$). **$^1$H NMR** (400 MHz, $CDCl_3$, ppm): $\delta$ 7.55 (d, *J* = 7.6 Hz, 2H), 7.50 (d, *J* = 8.0 Hz, 2H), 7.44 (t, *J* = 7.4 Hz, 2H), 7.39-7.25 (m, 5H), 7.20-6.96 (m, 6H), 6.83 (d, *J* = 8.0 Hz, 2H), 6.09-6.01 (m, 1H), 5.89-5.78 (m, 1H), 4.84-4.77 (m, 1H), 3.24-2.99 (m, 2H). **$^{13}$C NMR** (100 MHz, $CDCl_3$, ppm): $\delta$ 173.7, 171.3, 141.0, 140.8, 136.4, 135.6, 135.2, 130.7, 130.5, 129.3, 128.9, 128.7, 127.5, 127.2, 126.8, 126.6, 122.8, 122.7. **HPLC analysis:** Daicel CHIRALPAK AD-H, *n*-hexane:*i*-PrOH = 80:20, flow rate = 0 1.0 mL/min, λ = 254 nm, retention time: $t_{major}$ = 11.8 min, $t_{minor}$ = 16.4 min. **HRMS** (ESI) calcd for $C_{31}H_{25}N_2O_2$ $[M + H]^+$: 457.1907, found 457.1911.

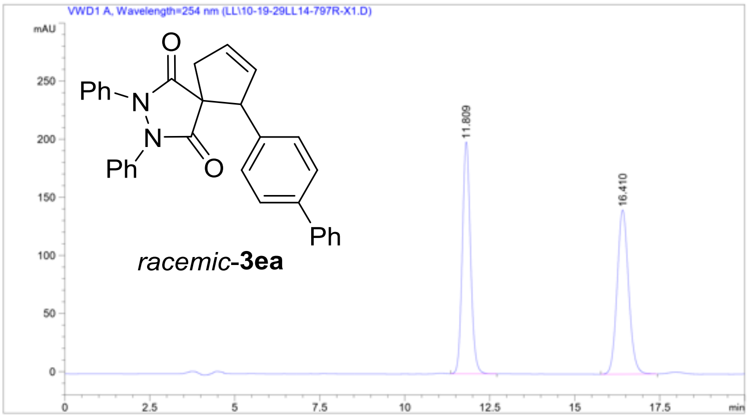


| Peak # | Ret. Time [min] | Type | Width [min] | Area [mAU*s] | Height [mAU] | Area % |
|---|---|---|---|---|---|---|
| 1 | 11.809 | HB | 0.2577 | 3349.02734 | 199.66058 | 50.2716 |
| 2 | 16.410 | HH | 0.3625 | 3312.83350 | 141.29733 | 49.7284 |

**Supplementary Figure 36.** HPLC spectrum of *racemic* **3ea**

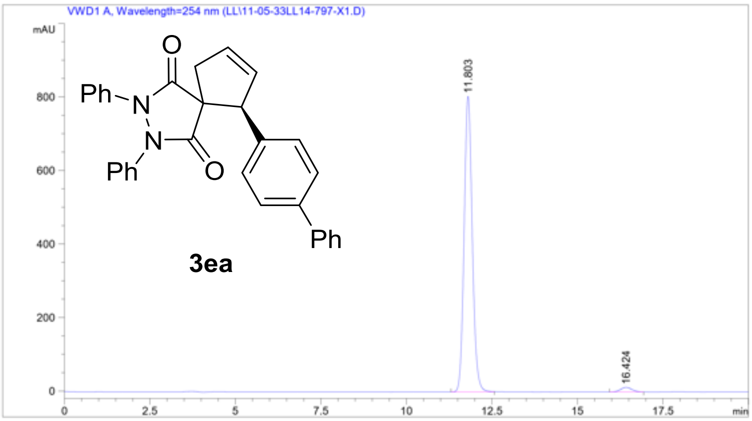


| Peak # | Ret. Time [min] | Type | Width [min] | Area [mAU*s] | Height [mAU] | Area % |
|---|---|---|---|---|---|---|
| 1 | 11.803 | HHA | 0.2564 | 1.33290e4 | 804.11316 | 97.9040 |
| 2 | 16.424 | HHA | 0.3528 | 285.35324 | 12.47663 | 2.0960 |

**Supplementary Figure 37.** HPLC spectrum of **3ea**

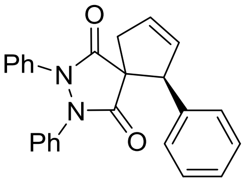

**3fa**

**(*S*)-2,3,6-triphenyl-2,3-diazaspiro[4.4]non-7-ene-1,4-dione (3fa).** Following the *General Procedure I*: BnN(Me)$_2$ (2 equiv), 30 $^{o}$C, 22 h. **3fa** was obtained as a white

soild (26.2 mg, 69% yield, 96% ee). **[α]$_D^{20}$** = -12.6 (*c* 1.0, $CHCl_3$). **$^1$H NMR** (400 MHz, $CDCl_3$, ppm): *δ* 7.31-7.26 (m, 5H), 7.25-7.20 (m, 2H), 7.09-7.09 (m, 5H), 7.07-7.00 (m, 1H), 6.87-6.78 (m, 2H), 6.08-5.98 (m, 1H), 5.87-5.77 (m, 1H), 4.80-4.71 (m, 1H), 3.23-2.96 (m, 2H). **$^{13}$C NMR** (100 MHz, $CDCl_3$, ppm): *δ* 173.8, 171.23, 137.4, 135.6, 135.2, 130.6, 130.5, 128.9, 128.7, 128.5, 128.1, 126.8, 126.6, 122.8, 122.7, 63.6, 59.6, 40.6. **HPLC analysis:** Daicel CHIRALPAK OD-H, *n*-hexane:*i*-PrOH = 90:10, flow rate = 1.0 mL/min, λ = 254 nm, retention time: $t_{major}$ = 15.3 min, $t_{minor}$ = 16.4 min. **HRMS** (ESI) calcd for $C_{25}H_{21}N_2O_2$ [M + H]$^+$: 381.1596, found 381.1598.

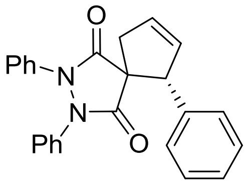


*ent*-**3fa**

**(*R*)-2,3,6-triphenyl-2,3-diazaspiro[4.4]non-7-ene-1,4-dione (*ent*-3fa).** Following the *General Procedure II*: DIPEA (2 equiv), 30 °C, 12 h. *ent*-**3fa** was obtained as a white soild (24.3 mg, 64% yield, 94% ee). **[α]$_D^{20}$** = +14.0 (*c* 1.0, $CHCl_3$). **HPLC analysis:** Daicel CHIRALPAK OD-H, *n*-hexane:*i*-PrOH = 90:10, flow rate = 1.0 mL/min, λ = 254 nm, retention time: $t_{major}$ = 15.4 min, $t_{minor}$ = 16.3 min. **HRMS** (ESI) calcd for $C_{25}H_{21}N_2O_2$ [M + H]$^+$: 381.1596, found 381.1598.

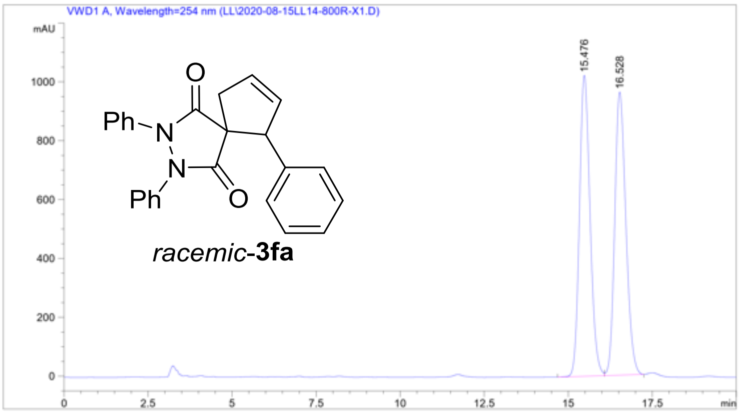


| Peak # | Ret. Time [min] | Type | Width [min] | Area [mAU*s] | Height [mAU] | Area % |
|---|---|---|---|---|---|---|
| 1 | 15.476 | BV | 0.3393 | 2.23759e4 | 1022.62805 | 50.2720 |
| 2 | 16.528 | VB | 0.3554 | 2.21337e4 | 962.07532 | 49.7280 |

**Supplementary Figure 38.** HPLC spectrum of *racemic* **3fa**

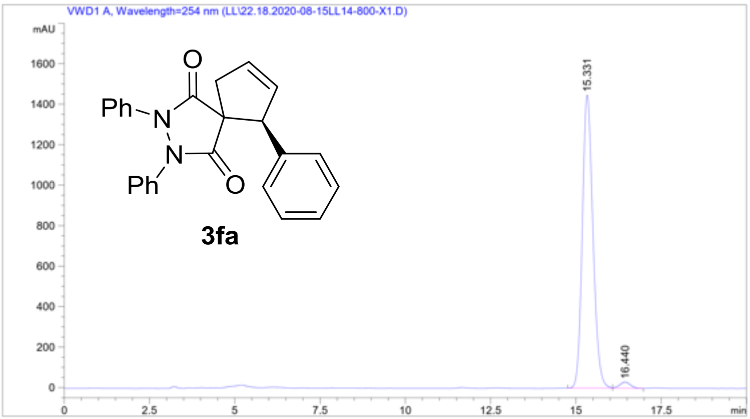


| Peak # | Ret. Time [min] | Type | Width [min] | Area [mAU*s] | Height [mAU] | Area % |
|---|---|---|---|---|---|---|
| 1 | 15.331 | BF | 0.3335 | 3.13368e4 | 1447.78564 | 97.9306 |
| 2 | 16.440 | VB R | 0.3306 | 662.18530 | 30.70007 | 2.0694 |

**Supplementary Figure 39.** HPLC spectrum of **3fa**

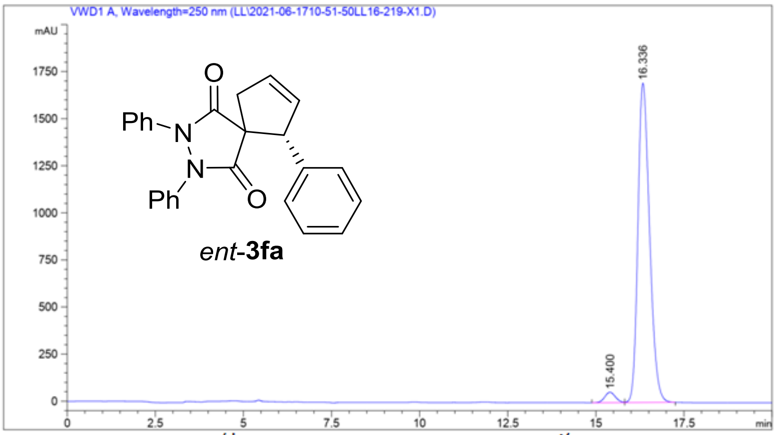


| Peak # | Ret. Time [min] | Type | Width [min] | Area [mAU*s] | Height [mAU] | Area % |
|---|---|---|---|---|---|---|
| 1 | 15.400 | BV | 0.3271 | 1153.30078 | 54.90415 | 2.8754 |
| 2 | 16.336 | VBA | 0.3550 | 3.89554e4 | 1695.68762 | 97.1246 |

**Supplementary Figure 40.** HPLC spectrum of *ent*-**3fa**

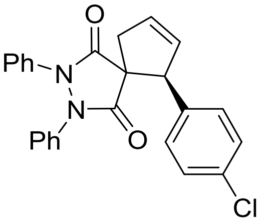


**3ga**

**(*S*)-6-(4-chlorophenyl)-2,3-diphenyl-2,3-diazaspiro[4.4]non-7-ene-1,4-dione (3ga).** Following the *General Procedure I*: BnN(Me)$_2$ (2 equiv), 30 $^{o}$C, 22 h. **3ga** was

obtained as a white solid (28.6 mg, 69% yield, 96% ee). **$[\alpha]_D^{20}$** = -20.0 (*c* 1.0, $CHCl_3$). **$^1$H NMR** (400 MHz, $CDCl_3$, ppm): $\delta$ 7.30 (t, *J* = 7.8 Hz, 2H), 7.26-7.22 (m, 2H), 7.21-7.11 (m, 7H), 7.10-7.04 (m, 1H), 6.92-6.83 (m, 2H), 6.08-5.99 (m, 1H), 5.81-5.73 (m, 1H), 3.21-3.98 (m, 2H). **$^{13}$C NMR** (100 MHz, $CDCl_3$, ppm): $\delta$ 173.6, 171.1, 135.9, 135.6, 135.2, 134.0, 130.9, 130.2, 130.1, 129.0, 128.8, 128.6, 126.9, 126.7, 122.6, 122.6, 62.6, 59.4, 40.8. **HPLC analysis:** Daicel CHIRALPAK AD-H, *n*-hexane:*i*-PrOH = 80:20, flow rate = 1.0 mL/min, λ = 254 nm, retention time: $t_{major}$ = 9.4 min, $t_{minor}$ = 10.9 min. **HRMS** (ESI) calcd for $C_{25}H_{20}ClN_2O_2$ $[M + H]^+$: 415.1208, found 415.1208.

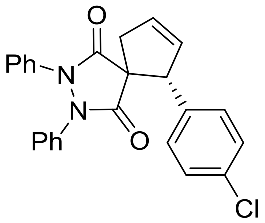


*ent*-**3ga**

**(*R*)-6-(4-chlorophenyl)-2,3-diphenyl-2,3-diazaspiro[4.4]non-7-ene-1,4-dione (*ent*-3ga).** Following the *General Procedure II*: DIPEA (2 equiv), 30 °C, 12 h. *ent*-**3ga** was obtained as a white solid (26.1 mg, 63% yield, 94% ee). **$[\alpha]_D^{20}$** = +14.7 (*c* 1.0, $CHCl_3$). **HPLC analysis:** Daicel CHIRALPAK AD-H, *n*-hexane:*i*-PrOH = 80:20, flow rate = 1.0 mL/min, λ = 254 nm, retention time: $t_{major}$ = 9.3 min, $t_{minor}$ = 10.8 min. **HRMS** (ESI) calcd for $C_{25}H_{20}ClN_2O_2$ $[M + H]^+$: 415.1208, found 415.1207.

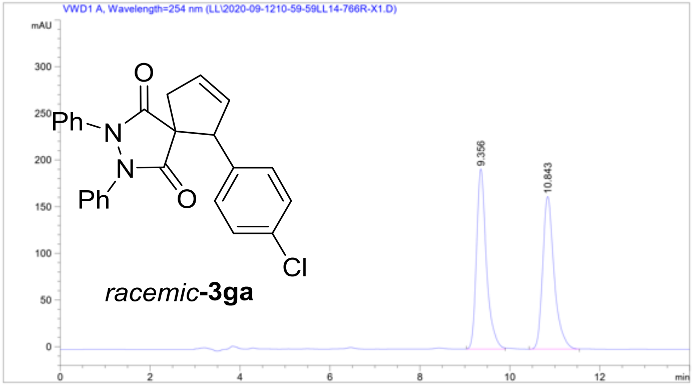


| Peak # | Ret. Time [min] | Type | Width [min] | Area [mAU*s] | Height [mAU] | Area % |
|---|---|---|---|---|---|---|
| 1 | 9.356 | FF | 0.2299 | 2941.14746 | 192.89392 | 50.4303 |
| 2 | 10.843 | FF | 0.2665 | 2890.95996 | 163.35089 | 49.5697 |

**Supplementary Figure 41.** HPLC spectrum of *racemic* **3ga**

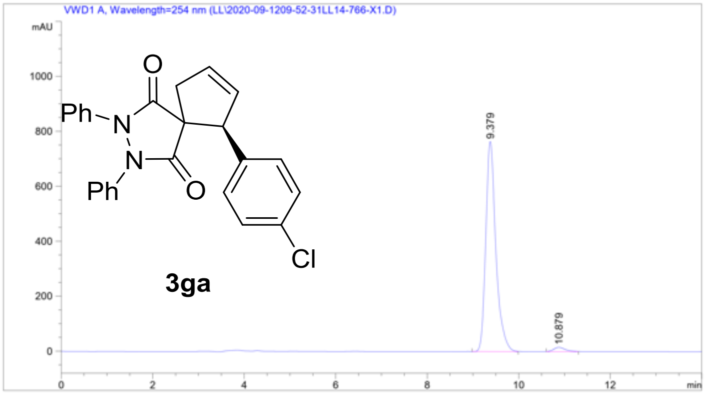


| Peak # | Ret. Time [min] | Type | Width [min] | Area [mAU*s] | Height [mAU] | Area % |
|---|---|---|---|---|---|---|
| 1 | 9.379 | BF | 0.2307 | 1.17102e4 | 764.44818 | 97.7308 |
| 2 | 10.879 | FF | 0.2643 | 271.90149 | 15.60178 | 2.2692 |

**Supplementary Figure 42.** HPLC spectrum of **3ga**

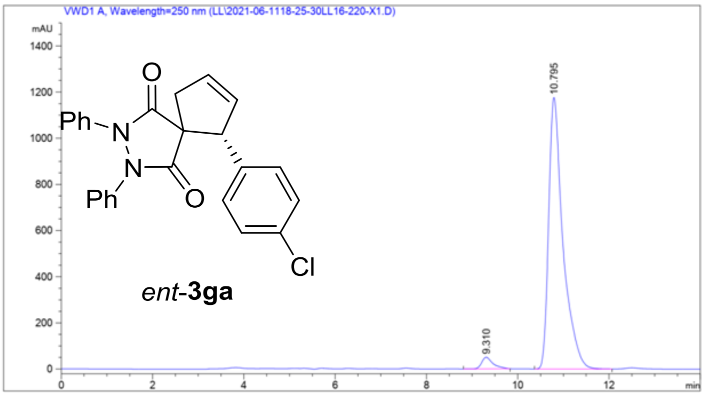


| Peak # | Ret. Time [min] | Type | Width [min] | Area [mAU*s] | Height [mAU] | Area % |
|---|---|---|---|---|---|---|
| 1 | 9.310 | BBA | 0.2536 | 856.70660 | 50.08592 | 3.2452 |
| 2 | 10.795 | BBA | 0.3193 | 2.55425e4 | 1176.50427 | 96.7548 |

**Supplementary Figure 43.** HPLC spectrum of *ent*-**3ga**

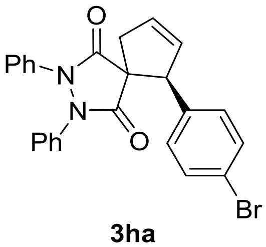

**3ha**

**(*S*)-6-(4-bromophenyl)-2,3-diphenyl-2,3-diazaspiro[4.4]non-7-ene-1,4-dione (3ha).** Following the *General Procedure I*: BnN(Me)$_2$ (2 equiv), 30 $^{o}$C, 22 h. **3ha** was obtained as a white solid (30.8 mg, 67% yield, 96% ee). **[α]$_D$$^{20}$** = 18.9 (*c* 1.0, $CHCl_3$). **$^1$H NMR** (400 MHz, $CDCl_3$, ppm): $\delta$ 7.42-7.36 (m, 2H), 7.33-7.27 (m, 2H), 7.21-7.05

(m, 8H), 6.90-6.83 (m, 2H), 6.07-6.01 (m, 1H), 5.79-5.72 (m, 1H), 4.73-4.67 (m, 1H), 3.19-2.98 (m, 2H). **$^{13}$C NMR** (100 MHz, $CDCl_3$, ppm): $\delta$ 173.5, 171.1, 136.4, 135.6, 135.2, 131.6, 131.0, 130.6, 130.0, 129.0, 128.8, 126.9, 126.8, 122.7, 122.6, 122.1, 62.7, 59.3, 40.8. **HPLC analysis:** Daicel CHIRALPAK AD-H, *n*-hexane:*i*-PrOH = 80:20, flow rate = 1.0 mL/min, λ = 254 nm, retention time: $t_{major}$ = 9.4 min, $t_{minor}$ = 11.4 min. **HRMS** (ESI) calcd for $C_{25}H_{20}BrN_2O_2$ $[M + H]^+$: 459.0701, found 459.0703.

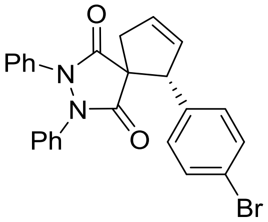


*ent*-**3ha**

**(*R*)-6-(4-bromophenyl)-2,3-diphenyl-2,3-diazaspiro[4.4]non-7-ene-1,4-dione (*ent*-3ha).** Following the *General Procedure II*: DIPEA (2 equiv), 30 $^{o}$C, 12 h. *ent*-**3ha** was obtained as a white solid (23.8 mg, 52% yield, 93% ee). **$[\alpha]_D^{20}$** = +22.3 (*c* 1.0, $CHCl_3$). **HPLC analysis:** Daicel CHIRALPAK AD-H, *n*-hexane:*i*-PrOH = 80:20, flow rate = 1.0 mL/min, λ = 254 nm, retention time: $t_{major}$ = 9.3 min, $t_{minor}$ = 11.4 min. **HRMS** (ESI) calcd for $C_{25}H_{20}BrN_2O_2$ $[M + H]^+$: 459.0703, found 459.0702.

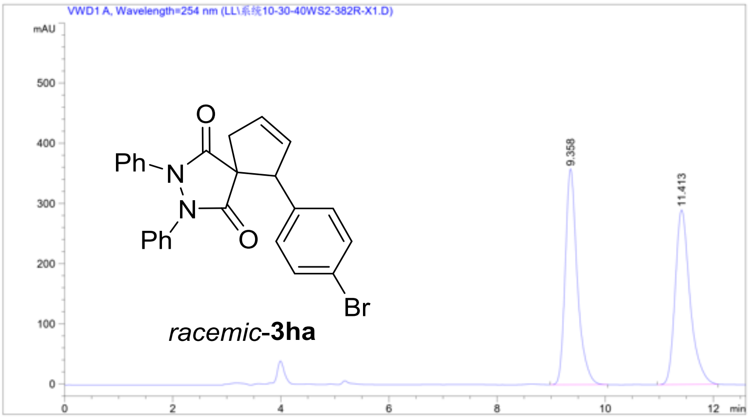


| Peak # | Ret. Time [min] | Type | Width [min] | Area [mAU*s] | Height [mAU] | Area % |
|---|---|---|---|---|---|---|
| 1 | 9.358 | BBA | 0.2331 | 5561.34717 | 358.24246 | 50.1307 |
| 2 | 11.413 | BBA | 0.2905 | 5532.33887 | 290.04147 | 49.8693 |

**Supplementary Figure 44.** HPLC spectrum of *racemic* **3ha**

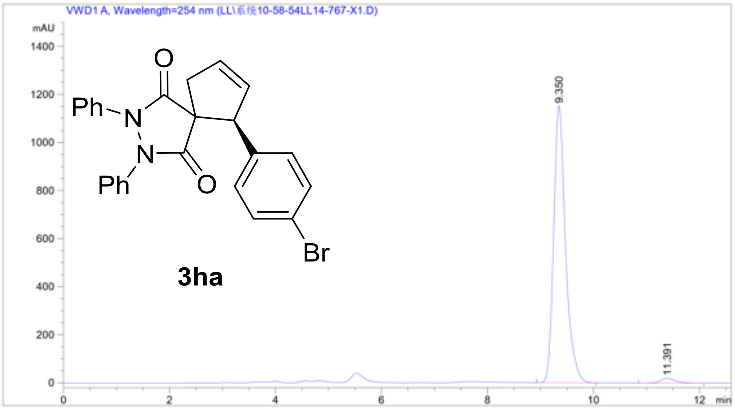


| Peak # | Ret. Time [min] | Type | Width [min] | Area [mAU*s] | Height [mAU] | Area % |
|---|---|---|---|---|---|---|
| 1 | 9.350 | BBA | 0.2372 | 1.78656e4 | 1150.30701 | 97.9302 |
| 2 | 11.391 | BBA | 0.2870 | 377.60306 | 19.75309 | 2.0698 |

**Supplementary Figure 45.** HPLC spectrum of **3ha**

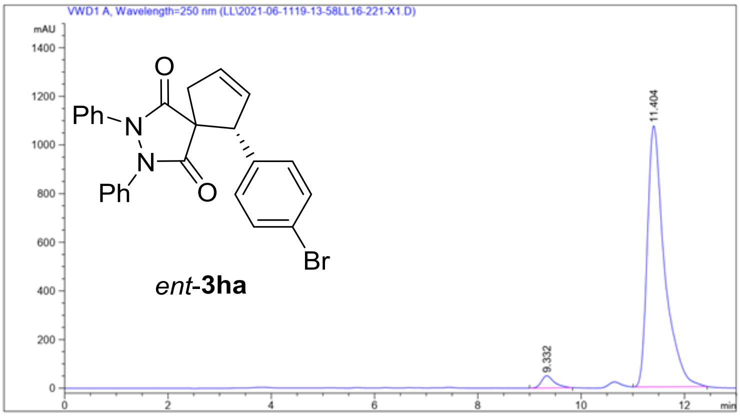


| Peak # | Ret. Time [min] | Type | Width [min] | Area [mAU*s] | Height [mAU] | Area % |
|---|---|---|---|---|---|---|
| 1 | 9.332 | BBA | 0.2523 | 854.25195 | 50.25657 | 3.3439 |
| 2 | 11.404 | BBA | 0.3385 | 2.46920e4 | 1072.88721 | 96.6561 |

**Supplementary Figure 46.** HPLC spectrum of *ent*-**3ha**

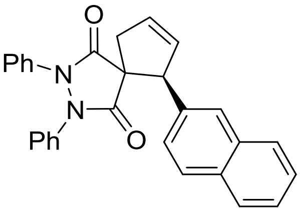


**3ia**

**(*S*)-6-(naphthalen-2-yl)-2,3-diphenyl-2,3-diazaspiro[4.4]non-7-ene-1,4-dione (3ia).** Following the *General Procedure I*: BnN(Me)$_2$ (2 equiv), 30 $^o$C, 22 h. **3ia** was

obtained as a white solid (25.8 mg, 60% yield, 96% ee). **[α]$_D^{20}$** = -21.9 (*c* 1.0, $CHCl_3$). **$^1$H NMR** (400 MHz, $CDCl_3$, ppm): $\delta$ 787-7.75 (m, 2H), 7.72-7.59 (m, 2H), 7.52-7.43 (m, 2H), 7.42-7.35 (m, 2H), 7.23 (d, $J$ = 8.0 Hz, 2H), 7.08-6.91 (m, 5H), 6.74-6.68 (m, 2H), 6.15-6.06 (m, 1H), 5.97-5.87 (m, 1H), 4.98-4.89 (m, 1H), 3.29-3.03 (m, 2H). **$^{13}$C NMR** (100 MHz, $CDCl_3$, ppm): $\delta$ 173.9, 171.2, 135.7, 135.1, 134.9, 133.2, 133.2, 130.8, 130.5, 129.0, 128.6, 128.2, 128.0, 127.8, 126.8, 126.7, 126.4, 126.3, 126.1, 122.7, 122.4, 63.8, 59.8, 40.6. **HPLC analysis:** Daicel CHIRALPAK AD-H, *n*-hexane:*i*-PrOH = 90:10, flow rate = 1.0 mL/min, λ = 254 nm, retention time: $t_{major}$ = 17.7 min, $t_{minor}$ = 25.9 min. **HRMS** (ESI) calcd for $C_{29}H_{23}N_2O_2$ [M + H]$^+$: 431.1753, found 431.1754.

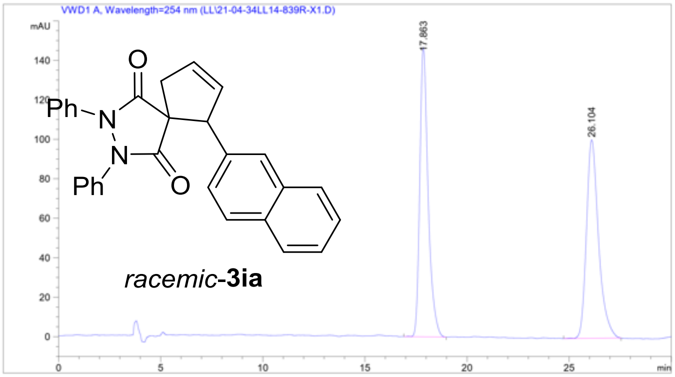


| Peak # | Ret. Time [min] | Type | Width [min] | Area [mAU*s] | Height [mAU] | Area % |
|---|---|---|---|---|---|---|
| 1 | 17.863 | VB R | 0.4235 | 4162.91846 | 146.51039 | 50.1352 |
| 2 | 26.104 | VBAR | 0.6246 | 4140.46045 | 100.51908 | 49.8648 |

**Supplementary Figure 47.** HPLC spectrum of *racemic* **3ia**

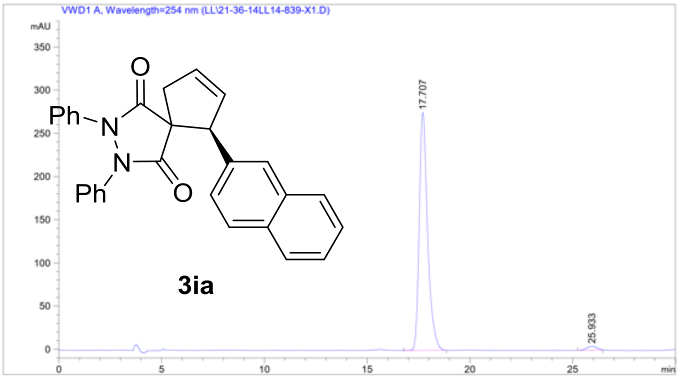


| Peak # | Ret. Time [min] | Type | Width [min] | Area [mAU*s] | Height [mAU] | Area % |
|---|---|---|---|---|---|---|
| 1 | 17.707 | BBA | 0.4263 | 7792.47314 | 275.25040 | 98.1325 |
| 2 | 25.933 | BBA | 0.5278 | 148.29086 | 4.50758 | 1.8675 |

**Supplementary Figure 48.** HPLC spectrum of **3ia**

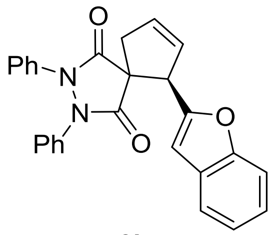

**3ja**

**(*R*)-6-(benzofuran-2-yl)-2,3-diphenyl-2,3-diazaspiro[4.4]non-7-ene-1,4-dione (3ja).** Following the *General Procedure I*: $BnN(Me)_2$ (2 equiv), 30 °C, 36 h. **3ja** was obtained as a white solid (22.7 mg, 54% yield, 94% ee). $[\alpha]_D^{20}$ = -12.5 (*c* 1.0, $CHCl_3$). **$^1$H NMR** (400 MHz, $CDCl_3$, ppm): $\delta$ 7.54-7.46 (m, 1H), 7.40-7.29 (m, 4H), 7.29-7.08 (m, 7H), 7.08-6.99 (m, 3H), 6.63 (s, 1H), 6.10-6.00 (m, 1H), 5.94-5.83 (m, 1H), 4.89 (s, 1H), 3.26-3.03 (m, 2H). **$^{13}$C NMR** (100 MHz, $CDCl_3$, ppm): $\delta$ 173.5, 170.9, 154.8, 154.6, 136.1, 135.6, 131.4, 129.0, 128.8, 128.4, 127.2, 126.9, 126.6, 124.2, 122.9, 122.7, 122.5, 121.1. **HPLC analysis:** Daicel CHIRALPAK AD-H, *n*-hexane:*i*-PrOH = 70:30, flow rate = 1.0 mL/min, λ = 254 nm, retention time: $t_{major}$ = 18.6 min, $t_{minor}$ = 21.0 min. **HRMS** (ESI) calcd for $C_{27}H_{21}N_2O_3$ $[M + H]^+$: 421.1552, found 421.1574.

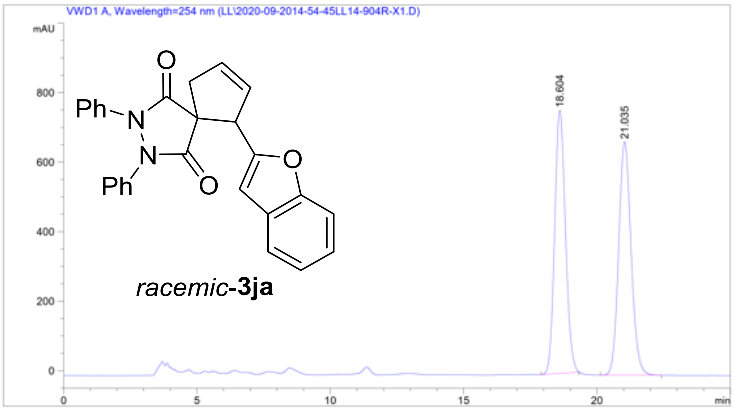


| Peak # | Ret. Time [min] | Type | Width [min] | Area [mAU*s] | Height [mAU] | Area % |
|---|---|---|---|---|---|---|
| 1 | 18.604 | BBA | 0.4334 | 2.11494e4 | 755.90936 | 49.3411 |
| 2 | 21.035 | BBA | 0.5016 | 2.17142e4 | 670.84607 | 50.6589 |

**Supplementary Figure 49.** HPLC spectrum of *racemic* **3ja**

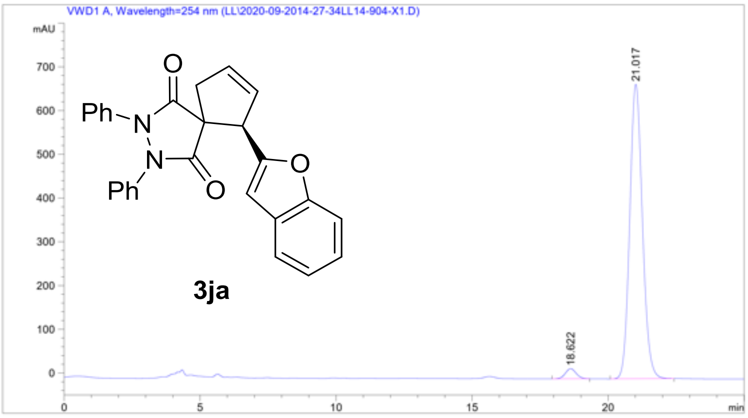


| Peak # | Ret. Time [min] | Type | Width [min] | Area [mAU*s] | Height [mAU] | Area % |
|---|---|---|---|---|---|---|
| 1 | 18.622 | BBA | 0.4311 | 641.53680 | 23.16592 | 2.8643 |
| 2 | 21.017 | BBA | 0.5001 | 2.17559e4 | 673.10406 | 97.1357 |

**Supplementary Figure 50.** HPLC spectrum of **3ja**

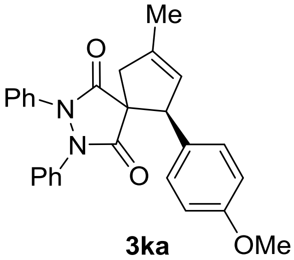

**3ka**

**(*S*)-6-(4-methoxyphenyl)-8-methyl-2,3-diphenyl-2,3-diazaspiro[4.4]non-7-ene-1,4-dione (3ka).** Following the *General Procedure I*: $BnN(Me)_2$ (2 equiv), 30 °C, 22 h. **3ka** was obtained as a yellow oil (27.6 mg, 65%, 93% ee). $[\alpha]_D^{20}$ = -14.3 (*c* 1.0, $CHCl_3$). **$^1$H NMR** (400 MHz, $CDCl_3$, ppm): $\delta$ 7.33-7.24 (m, 2H), 7.20-7.10 (m, 7H), 7.08-7.00 (m, 1H), 6.90-6.85 (m, 2H), 6.81-6.75 (m, 2H), 5.41-5.33 (m, 1H), 4.72-4.62 (m, 1H), 3.78 (s, 3H), 3.12-2.99 (m, 1H), 2.88-2.75 (m, 1H), 1.90 (s, 3H). **$^{13}$C NMR** (100 MHz, $CDCl_3$, ppm): $\delta$ 173.9, 171.6, 159.4, 140.2, 135.7, 135.4, 130.0, 130.0, 128.9, 128.6, 126.7, 126.4, 124.5, 122.6, 122.5, 113.7, 63.0, 60.3, 55.4, 43.9, 16.4. **HPLC analysis:** Daicel CHIRALPAK AD-H, *n*-hexane:*i*-PrOH = 80:20, flow rate = 1.0 mL/min, $\lambda$ = 254 nm, retention time: $t_{major}$ = 9.0 min, $t_{minor}$ = 17.9 min. **HRMS** (ESI) calcd for $C_{27}H_{25}N_2O_3$ $[M + H]^+$: 425.1820, found 421.1860.

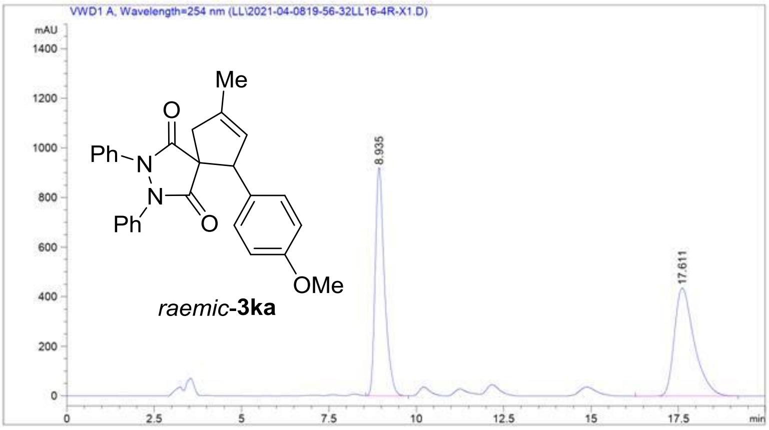


| Peak # | Ret. Time [min] | Type | Width [min] | Area [mAU*s] | Height [mAU] | Area % |
|---|---|---|---|---|---|---|
| 1 | 8.935 | VBAS | 0.2752 | 1.69834e4 | 920.56158 | 49.6382 |
| 2 | 17.611 | BBAS | 0.5972 | 1.72310e4 | 435.91959 | 50.3618 |

**Supplementary Figure 51.** HPLC spectrum of *racemic* **3ka**

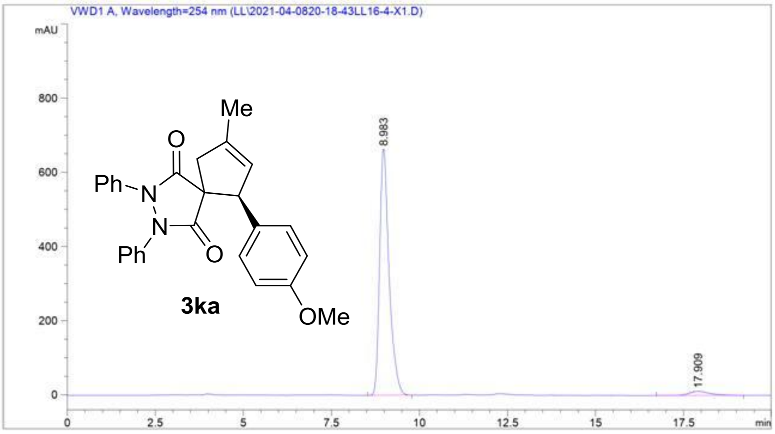


| Peak # | Ret. Time [min] | Type | Width [min] | Area [mAU*s] | Height [mAU] | Area % |
|---|---|---|---|---|---|---|
| 1 | 8.983 | VBAS | 0.2784 | 1.24263e4 | 663.94043 | 96.6179 |
| 2 | 17.909 | BBAS | 0.5842 | 434.98016 | 11.12307 | 3.3821 |

**Supplementary Figure 52.** HPLC spectrum of **3ka**

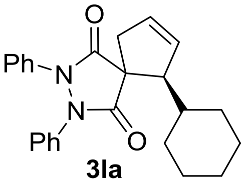

**3la**

**(*R*)-6-cyclohexyl-2,3-diphenyl-2,3-diazaspiro[4.4]non-7-ene-1,4-dione (3la).** Following the *General Procedure I*: $BnN(Me)_2$ (2 equiv), 40 $^oC$, 36 h. **3la** was obtained as a yellow oil (22.5 mg, 58% yield, 96% ee). $[\alpha]_D^{20}$ = -12.5 (*c* 1.0, $CHCl_3$). **$^1$H NMR** (400 MHz, $CDCl_3$, ppm): $\delta$ 7.36-7.27 (m, 8H), 7.22-7.13 (m, 2H), 5.94-5.83 (m, 1H), 5.78-5.66 (m, 1H), 3.23 (d, *J* = 10 Hz, 1H), 2.97 (dd, *J* = 35 Hz, 16 Hz, 2H),

1.96 (d, $J$ = 11 Hz, 1H), 1.82 (q, $J$ = 11 Hz, 1H), 1.82 (d, $J$ = 13 Hz, 1H), 1.67-1.48 (m, 4H), 1.36-0.83 (m, 5H). **$^{13}$C NMR** (100 MHz, $CDCl_3$, ppm): $\delta$ 174.7, 172.9, 136.3, 131.4, 129.1, 129.1, 126.7, 126.3, 122.4, 122.2, 61.8, 55.6, 44.6, 37.7, 32.7, 32.2, 26.3, 26.0, 25.8. **HPLC analysis:** Daicel CHIRALPAK IA, *n*-hexane:*i*-PrOH = 80:20, flow rate = 1.0 mL/min, λ = 254 nm, retention time: $t_{major}$ = 8.6 min, $t_{minor}$ = 11.7 min. **HRMS** (ESI) calcd for $C_{25}H_{27}N_2O_2$ $[M + H]^+$: 387.2073, found 387.2067.

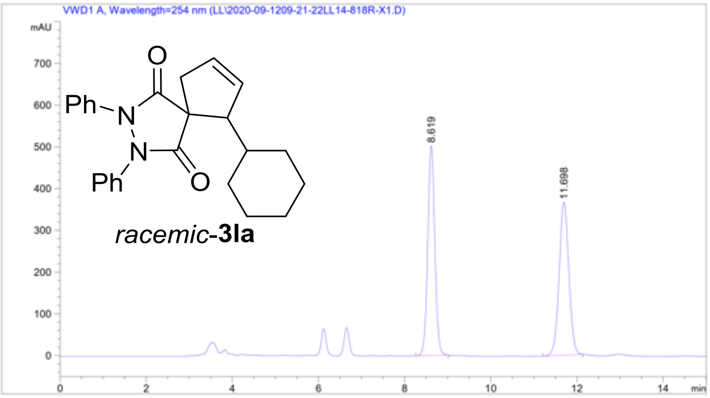


| Peak # | Ret. Time [min] | Type | Width [min] | Area [mAU*s] | Height [mAU] | Area % |
|---|---|---|---|---|---|---|
| 1 | 8.619 | BBA | 0.1746 | 5718.10010 | 503.13672 | 49.9907 |
| 2 | 11.698 | BBA | 0.2411 | 5720.23730 | 366.45078 | 50.0093 |

**Supplementary Figure 53.** HPLC spectrum of *racemic* **3la**

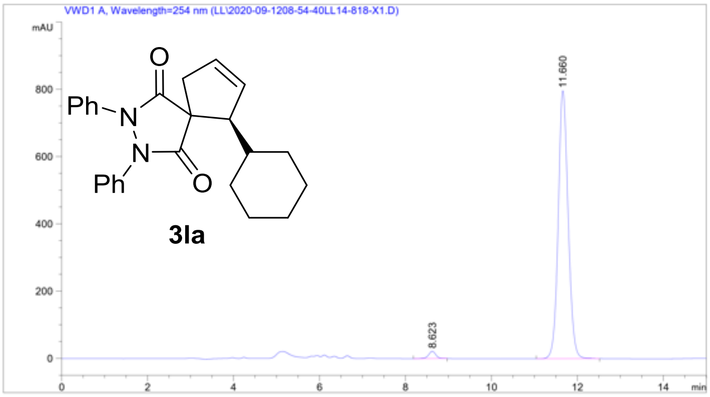


| Peak # | Ret. Time [min] | Type | Width [min] | Area [mAU*s] | Height [mAU] | Area % |
|---|---|---|---|---|---|---|
| 1 | 8.623 | BBA | 0.1796 | 252.70131 | 21.57474 | 1.9473 |
| 2 | 11.660 | BBA | 0.2444 | 1.27243e4 | 796.46411 | 98.0527 |

**Supplementary Figure 54.** HPLC spectrum of **3la**

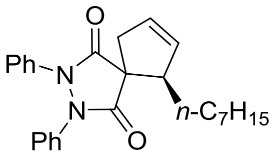

**3ma**

**(*R*)-6-heptyl-2,3-diphenyl-2,3-diazaspiro[4.4]non-7-ene-1,4-dione (3ma).** Following the *General Procedure I*: $BnN(Me)_2$ (2 equiv), 30 °C, 36 h. **3ma** was obtained as a yellow oil (29.4 mg, 73% yield, 94% ee). $[\alpha]_D^{20}$ = -11.5 (*c* 1.0, $CHCl_3$). **$^1$HNMR** (400 MHz, $CDCl_3$, ppm): $\delta$ 7.38-7.28 (m, 10H), 7.22-7.13 (m, 2H), 5.78-5.63 (m, 2H), 3.49-3.35 (m, 1H), 3.11-2.87 (m, 2H), 1.74-1.56 (m, 2H), 1.40-1.13 (m, 10H), 0.85 (t, *J* = 6.7 Hz, 3H). **$^{13}$C NMR** (100 MHz, $CDCl_3$, ppm): $\delta$ 174.8, 172.6, 136.2, 136.1, 132.4, 129.1, 129.0, 127.0, 126.7, 126.7, 122.4, 122.3, 57.0, 55.8, 42.8, 31.8, 30.7, 29.6, 29.2, 28.6, 22.7, 14.2. **HPLC analysis:** Daicel CHIRALPAK IA, *n*-hexane:*i*-PrOH = 95:5, flow rate = 1.0 mL/min, λ = 254 nm, retention time: $t_{major}$ = 15.5 min, $t_{minor}$ = 16.4 min. **HRMS** (ESI) calcd for $C_{26}H_{31}N_2O_2$ $[M + H]^+$: 403.2380, found 403.2379.

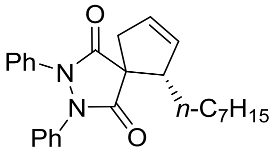

*ent*-**3ma**

**(*S*)-6-heptyl-2,3-diphenyl-2,3-diazaspiro[4.4]non-7-ene-1,4-dione (*ent*-3ma).** Following the *General Procedure I*: DIPEA (2 equiv), 40 °C, 36 h. *ent*-**3ma** was obtained as a yellow oil (22.9 mg, 57% yield, 85% ee). $[\alpha]_D^{20}$ = +3.8 (*c* 1.0, $CHCl_3$). **HPLC analysis:** Daicel CHIRALPAK IA, *n*-hexane:*i*-PrOH = 95:5, flow rate = 1.0 mL/min, λ = 254 nm, retention time: $t_{minor}$ = 15.5 min, $t_{major}$ = 16.3 min. **HRMS** (ESI) calcd for $C_{26}H_{31}N_2O_2$ $[M + H]^+$: 403.2380, found 403.2382.

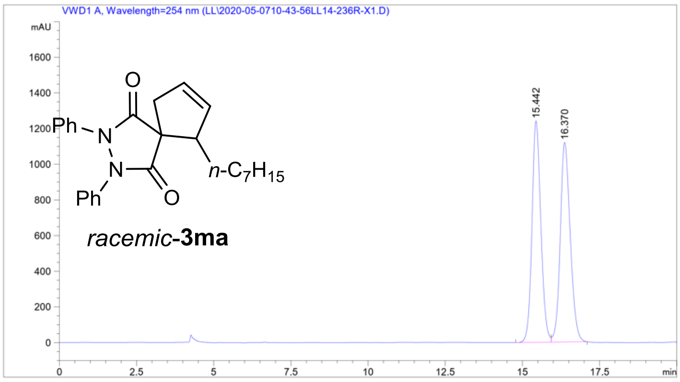


| Peak # | Ret. Time [min] | Type | Width [min] | Area [mAU*s] | Height [mAU] | Area % |
|---|---|---|---|---|---|---|
| 1 | 15.442 | BF | 0.3096 | 2.48510e4 | 1240.89893 | 49.7265 |
| 2 | 16.370 | VBA | 0.3458 | 2.51243e4 | 1119.47327 | 50.2735 |

**Supplementary Figure 55.** HPLC spectrum of *racemic* **3ma**

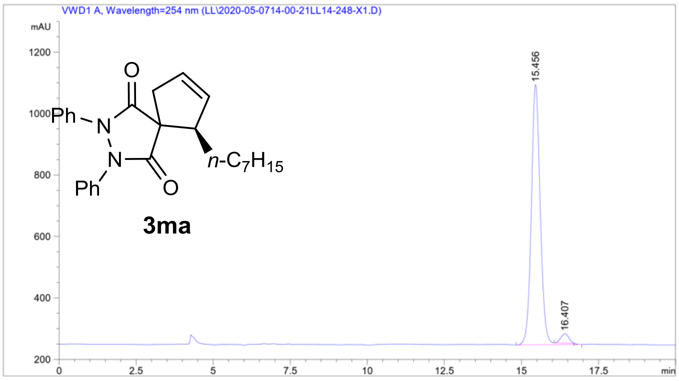


| Peak # | Ret. Time [min] | Type | Width [min] | Area [mAU*s] | Height [mAU] | Area % |
|---|---|---|---|---|---|---|
| 1 | 15.456 | BV R | 0.3046 | 1.71025e4 | 846.77521 | 96.9060 |
| 2 | 16.407 | VB E | 0.2883 | 546.04834 | 30.28784 | 3.0940 |

**Supplementary Figure 56.** HPLC spectrum of **3ma**

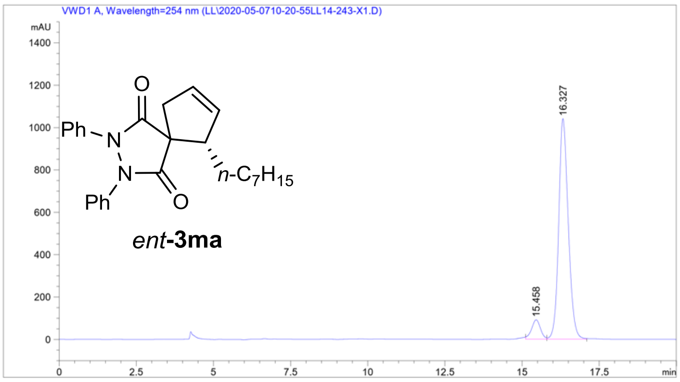


| Peak # | Ret. Time [min] | Type | Width [min] | Area [mAU*s] | Height [mAU] | Area % |
|---|---|---|---|---|---|---|
| 1 | 15.458 | FF | 0.3112 | 1842.44678 | 91.39951 | 7.6032 |
| 2 | 16.327 | FFAR | 0.3309 | 2.23902e4 | 1041.28601 | 92.3968 |

**Supplementary Figure 57.** HPLC spectrum of *ent*-**3ma**

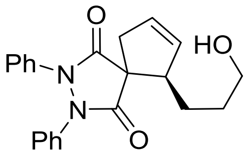


**3na**

**(*R*)-6-(3-hydroxypropyl)-2,3-diphenyl-2,3-diazaspiro[4.4]non-7-ene-1,4-dione (3na).** Following the *General Procedure I*: $BnN(Me)_2$ (2 equiv), 30 °C, 36 h. **3na** was obtained as a yellow oil (22.5 mg, 60% yield, 95% ee). $[\alpha]_D^{20}$ = -6.7 (*c* 1.0, $CHCl_3$). **$^1$H NMR** (400 MHz, $CDCl_3$, ppm): $\delta$ 7.39-7.27 (m, 8H), 7.23-7.12 (m, 2H), 5.81-5.65 (m, 2H), 3.65-3.53 (m, 2H), 3.50-3.36 (m, 1H), 2.99 (q, $J$ = 16 Hz, 2H), 1.84-1.44 (m, 4H). **$^{13}$C NMR** (100 MHz, $CDCl_3$, ppm): $\delta$ 174.6, 172.5, 136.1, 136.0, 132.0, 129.1, 129.1, 127.4, 126.9, 122.6, 122.4, 62.5, 55.8, 42.8, 31.5, 27.0. **HPLC analysis:** Daicel CHIRALPAK IA, *n*-hexane:*i*-PrOH = 80:20, flow rate = 1.0 mL/min, λ = 254 nm, retention time: $t_{major}$ = 16.0 min, $t_{minor}$ = 17.4 min. **HRMS** (ESI) calcd for $C_{22}H_{22}N_2O_3Na$ $[M + Na]^+$: 385.1520, found 385.1523.

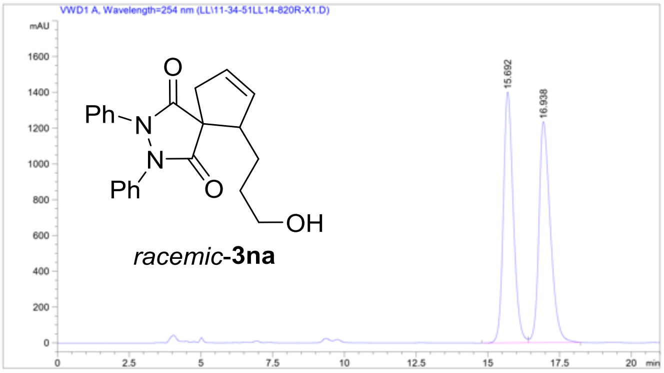


| Peak # | Ret. Time [min] | Type | Width [min] | Area [mAU*s] | Height [mAU] | Area % |
|---|---|---|---|---|---|---|
| 1 | 15.692 | BV | 0.3699 | 3.38690e4 | 1401.91382 | 49.7314 |
| 2 | 16.938 | VBA | 0.4234 | 3.42348e4 | 1234.85193 | 50.2686 |

**Supplementary Figure 58.** HPLC spectrum of *racemic* **3na**

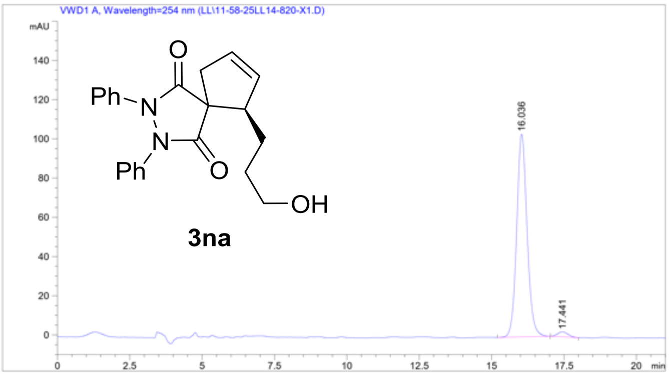


| Peak # | Ret. Time [min] | Type | Width [min] | Area [mAU*s] | Height [mAU] | Area % |
|---|---|---|---|---|---|---|
| 1 | 16.036 | BF | 0.3831 | 2579.58203 | 103.39506 | 97.6271 |
| 2 | 17.441 | BBA | 0.3884 | 62.69934 | 2.53605 | 2.3729 |

**Supplementary Figure 59.** HPLC spectrum of **3na**

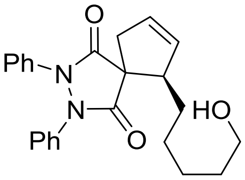


**3oa**

**(*R*)-6-(5-hydroxypentyl)-2,3-diphenyl-2,3-diazaspiro[4.4]non-7-ene-1,4-dione (3oa).** Following the *General Procedure I*: $BnN(Me)_2$ (2 equiv), 30 °C, 36 h. **3oa** was obtained as a yellow oil (25.7 mg, 66% yield, 93% ee). $[\alpha]_D^{20}$ = -0.2 (*c* 1.0, $CHCl_3$). **$^1$H NMR** (400 MHz, $CDCl_3$, ppm): $\delta$ 7.34-7.27 (m, 8H), 7.23-7.14 (m, 2H), 5.81-5.60 m, 2H), 3.54 (t, $J$ = 6.5 Hz, 2H), 3.48-3.37 (m, 1H), 3.09-2.87 (m, 2H), 2.31 (s, 1H), 1.77-1.56 (m, 3H), 1.54-1.21 (s, 7H). **$^{13}$C NMR** (100 MHz, $CDCl_3$, ppm): $\delta$ 174.8, 172.6, 136.2, 136.1, 132.3, 129.1, 129.1, 127.1, 126.8, 122.4, 122.3, 62.8, 56.7, 55.8, 42.8, 32.5, 30.6, 28.3, 25.7. **HPLC analysis:** Daicel CHIRALPAK AD-H, *n*-hexane:*i*-PrOH = 80:20, flow rate = 1.0 mL/min, λ = 254 nm, retention time: $t_{major}$ = 20.5 min, $t_{minor}$ = 21.9 min. **HRMS** (ESI) calcd for $C_{24}H_{27}N_2O_3$ [M + H]$^+$: 391.2011, found 391.2016.

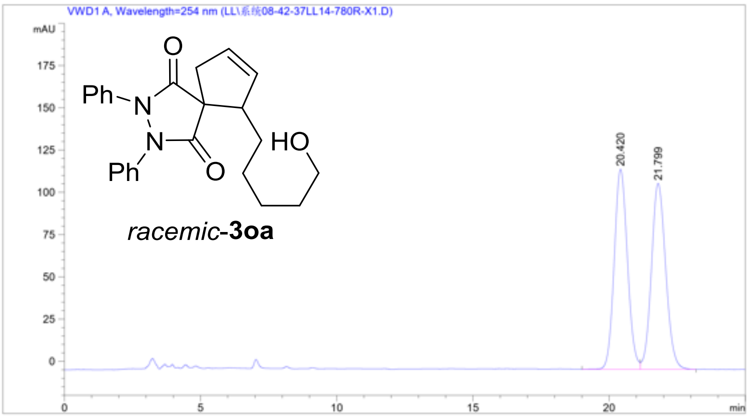


| Peak # | Ret. Time [min] | Type | Width [min] | Area [mAU*s] | Height [mAU] | Area % |
|---|---|---|---|---|---|---|
| 1 | 20.420 | BV | 0.5249 | 3994.11816 | 118.28478 | 49.8791 |
| 2 | 21.799 | VB | 0.5639 | 4013.47607 | 109.94421 | 50.1209 |

**Supplementary Figure 60.** HPLC spectrum of *racemic* **3oa**

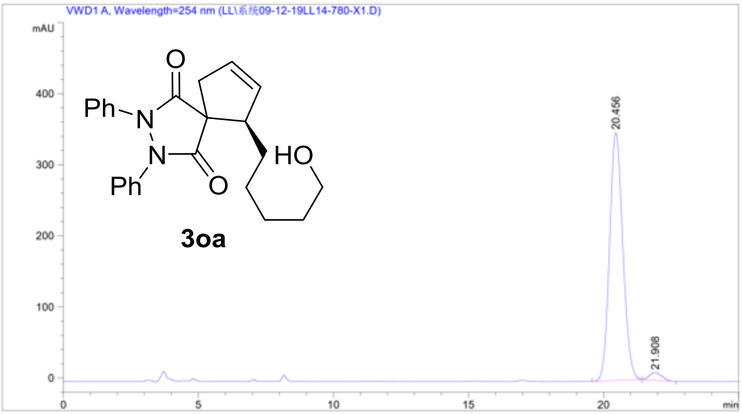


| Peak # | Ret. Time [min] | Type | Width [min] | Area [mAU*s] | Height [mAU] | Area % |
|---|---|---|---|---|---|---|
| 1 | 20.456 | BB | 0.5253 | 1.18378e4 | 348.45612 | 97.1771 |
| 2 | 21.908 | VB R | 0.5098 | 343.87177 | 10.61866 | 2.8229 |

**Supplementary Figure 61.** HPLC spectrum of **3oa**

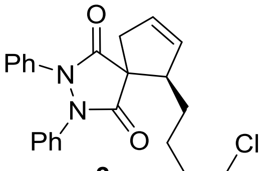


**3pa**

**(*R*)-6-(4-chlorobutyl)-2,3-diphenyl-2,3-diazaspiro[4.4]non-7-ene-1,4-dione (3pa).** Following the *General Procedure I*: $BnN(Me)_2$ (2 equiv), 40 $^{o}C$, 36 h. **3pa** was obtained as a yellow oil (23.2 mg, 59% yield, 95% ee). $[\alpha]_D^{20}$ = -6.6 (*c* 1.0, $CHCl_3$). **$^1$H NMR** (400 MHz, $CDCl_3$, ppm): $\delta$ 7.38-7.28 (m, 8H), 7.23-7.14 (m, 2H), 5.81-5.64 (m, 2H), 3.51-3.37 (m, 3H), 3.11-2.89 (m, 2H), 1.83-1.64 (m, 4H), 1.56-1.39 (m, 2H).

$^{13}$C NMR (100 MHz, $CDCl_3$, ppm): $\delta$ 174.6, 172.5, 136.1, 136.1, 132, 129.1, 129.1, 127.4, 126.9, 122.5, 122.4, 55.6, 55.6, 44.9, 43.0, 32.5, 30.0, 25.9. **HPLC analysis:** Daicel CHIRALPAK AD-H, *n*-hexane:*i*-PrOH = 90:10, flow rate = 1.0 mL/min, λ = 254 nm, retention time: $t_{minor}$ = 18.2 min, $t_{major}$ = 19.5 min. **HRMS** (ESI) calcd for $C_{23}H_{24}ClN_2O_2$ [M + H]$^+$: 395.1521, found 395.1516.

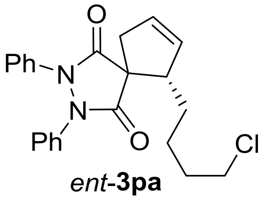


*ent*-**3pa**

**(*S*)-6-(4-chlorobutyl)-2,3-diphenyl-2,3-diazaspiro[4.4]non-7-ene-1,4-dione (*ent*-3pa).** Following the *General Procedure II*: DIPEA (2 equiv), 40 °C, 36 h. *ent*-**3pa** was obtained as a yellow oil (23.5 mg, 60% yield, 86% ee). **[α]$_D^{20}$** = +5.7 (*c* 1.0, $CHCl_3$). **HPLC analysis:** Daicel CHIRALPAK AD-H, *n*-hexane:*i*-PrOH = 90:10, flow rate = 1.0 mL/min, λ = 254 nm, retention time: $t_{minor}$ = 18.0 min, $t_{major}$ = 19.4 min. **HRMS** (ESI) calcd for $C_{23}H_{24}ClN_2O_2$ [M + H]$^+$: 395.1520, found 395.1521.

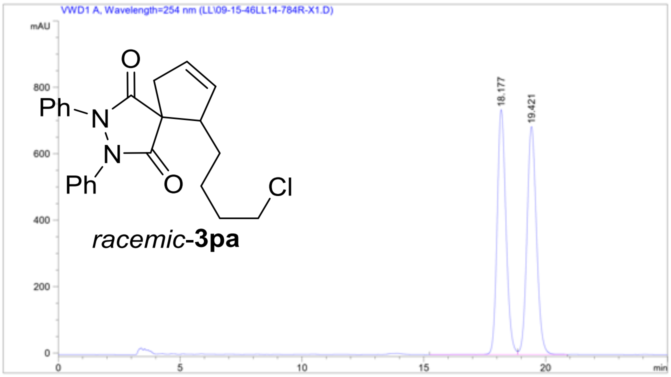


| Peak # | Ret. Time [min] | Type | Width [min] | Area [mAU*s] | Height [mAU] | Area % |
|---|---|---|---|---|---|---|
| 1 | 18.177 | VV R | 0.3887 | 1.85397e4 | 737.32111 | 49.9400 |
| 2 | 19.421 | VB | 0.4199 | 1.85842e4 | 686.24017 | 50.0600 |

**Supplementary Figure 62.** HPLC spectrum of *racemic* **3pa**

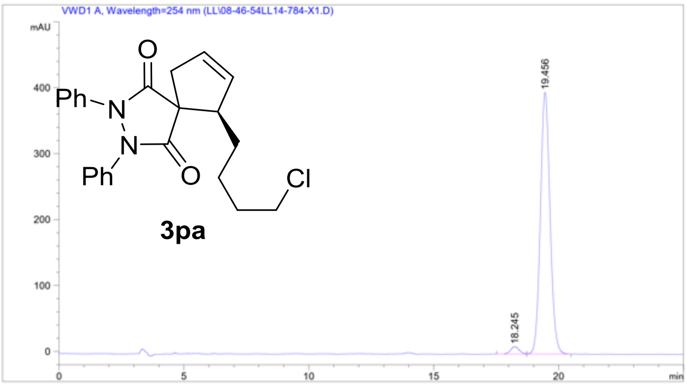


| Peak # | Ret. Time [min] | Type | Width [min] | Area [mAU*s] | Height [mAU] | Area % |
|---|---|---|---|---|---|---|
| 1 | 18.245 | BV | 0.3688 | 266.18353 | 11.21749 | 2.4486 |
| 2 | 19.456 | VBA | 0.4120 | 1.06046e4 | 396.59625 | 97.5514 |

**Supplementary Figure 63.** HPLC spectrum of **3pa**

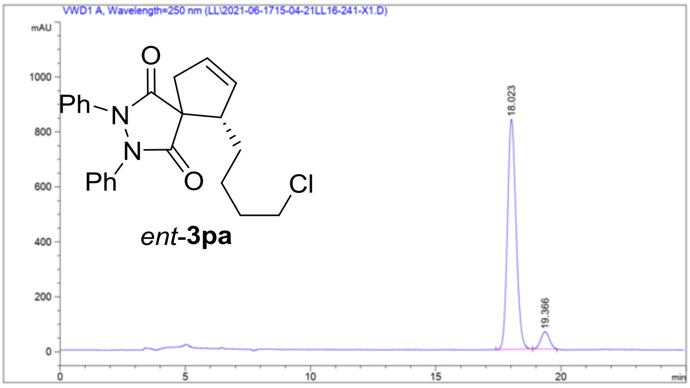


| Peak # | Ret. Time [min] | Type | Width [min] | Area [mAU*s] | Height [mAU] | Area % |
|---|---|---|---|---|---|---|
| 1 | 18.023 | BB | 0.3698 | 1.99312e4 | 836.94153 | 92.9883 |
| 2 | 19.366 | BBA | 0.3765 | 1502.89685 | 62.51593 | 7.0117 |

**Supplementary Figure 64.** HPLC spectrum of *ent*-**3pa**

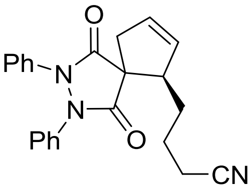


**3qa**

**(*R*)-4-(1,4-dioxo-2,3-diphenyl-2,3-diazaspiro[4.4]non-7-en-6-yl)butanenitrile (3qa).** Following the *General Procedure I*: BnN(Me)$_2$ (2 equiv), 40 °C, 36 h. **3qa** was obtained as a yellow oil (19.7 mg, 53% yield, 92% ee). **[α]$_D^{20}$** = -6.0 (*c* 1.0, $CHCl_3$). **$^1$H NMR** (400 MHz, $CDCl_3$, ppm): $\delta$ 7.36-7.29 (m, 8H), 7.24-7.15 (m, 8H), 5.85-5.61 (m, 2H), 3.49-3.33 (m, 1H), 3.11-2.93 (m, 2H), 2.38-2.23 (m, 2H), 1.86-1.56 (m, 4H).

**$^{13}$C NMR** (100 MHz, $CDCl_3$, ppm): $\delta$ 174.3, 172.2, 135.9, 135.8, 131.0, 129.2, 129.2, 129.1, 128.3, 127.1, 127.0, 122.6, 122.4, 119.2, 55.6, 42.7, 29.9, 24.4, 17.3. **HPLC analysis:** Daicel CHIRALPAK OD-H, *n*-hexane:*i*-PrOH = 80:20, flow rate = 1.0 mL/min, λ = 254 nm, retention time: $t_{major}$ = 20.5 min, $t_{minor}$ = 28.5 min. **HRMS** (ESI) calcd for $C_{23}H_{21}N_3O_2$ $[M + H]^+$: 372.1707, found 372.1704.

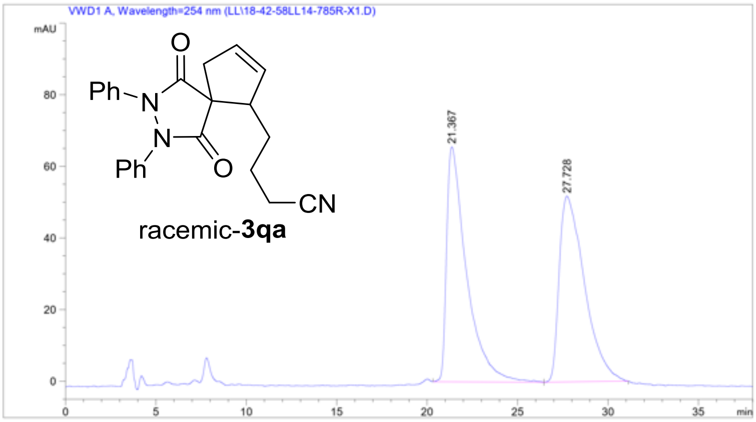


| Peak # | Ret. Time [min] | Type | Width [min] | Area [mAU*s] | Height [mAU] | Area % |
|---|---|---|---|---|---|---|
| 1 | 21.367 | BV R | 1.1389 | 5008.76025 | 65.59249 | 49.8606 |
| 2 | 27.728 | BBA | 1.3411 | 5036.77344 | 51.86148 | 50.1394 |

**Supplementary Figure 65.** HPLC spectrum of *racemic* **3qa**

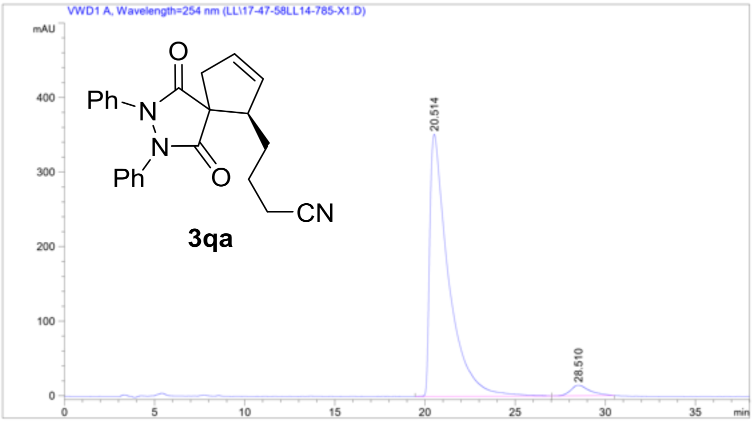


| Peak # | Ret. Time [min] | Type | Width [min] | Area [mAU*s] | Height [mAU] | Area % |
|---|---|---|---|---|---|---|
| 1 | 20.514 | BB | 1.0079 | 2.54320e4 | 351.61044 | 96.1037 |
| 2 | 28.510 | BBA | 1.0458 | 1031.07153 | 13.95749 | 3.8963 |

**Supplementary Figure 66.** HPLC spectrum of **3qa**

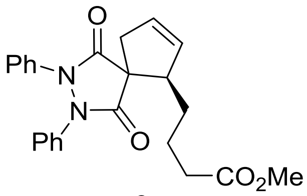


**3ra**

**Methyl (*R*)-4-(1,4-dioxo-2,3-diphenyl-2,3-diazaspiro[4.4]non-7-en-6-yl)butanoate (3ra).** Following the *General Procedure I*: BnN(Me)$_2$ (2 equiv), 30 $^o$C, 36 h. **3ra** was obtained as a yellow oil (19.7 mg, 61% yield, 93% ee). **[α]$_D$$^{20}$** = -6.9 (*c* 1.0, CHCl$_3$). **$^1$H NMR** (400 MHz, CDCl$_3$, ppm): $\delta$ 7.37-7.27 (m, 8H), 7.22-7.14 (m, 2H), 5.79-5.65 (m, 2H), 3.61 (s, 3H), 3.48-3.37 (m, 1H), 3.09-2.89 (m, 2H), 2.35-2.23 (m, 2H), 1.76-1.62 (m, 4H). **$^{13}$C NMR** (100 MHz, CDCl$_3$, ppm): $\delta$ 174.5, 173.6, 172.4, 136.1, 136.0, 131.9,129.1, 129.1, 127.5, 126.9, 122.7, 122.5, 56.4, 55.7, 51.7, 42.8, 34.0, 30.2, 23.9. **HPLC analysis:** Daicel CHIRALPAK IA, *n*-hexane:*i*-PrOH = 85:15, flow rate = 1.0 mL/min, λ = 254 nm, retention time: $t_{major}$ = 16.1 min, $t_{minor}$ = 17.0 min. **HRMS** (ESI) calcd for $C_{24}H_{25}N_2O_4$ [M + H]$^+$: 405.1804, found 405.1809.

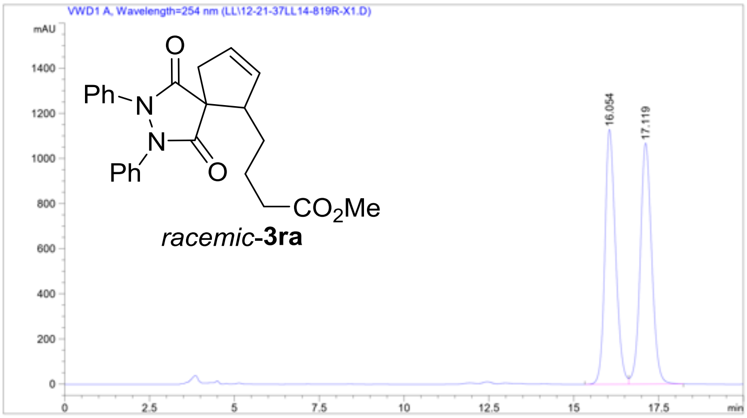


| Peak # | Ret. Time [min] | Type | Width [min] | Area [mAU*s] | Height [mAU] | Area % |
|---|---|---|---|---|---|---|
| 1 | 16.054 | BV | 0.3348 | 2.46702e4 | 1129.40552 | 49.5953 |
| 2 | 17.119 | VBA | 0.3619 | 2.50728e4 | 1067.80640 | 50.4047 |

**Supplementary Figure 67.** HPLC spectrum of *racemic* **3ra**

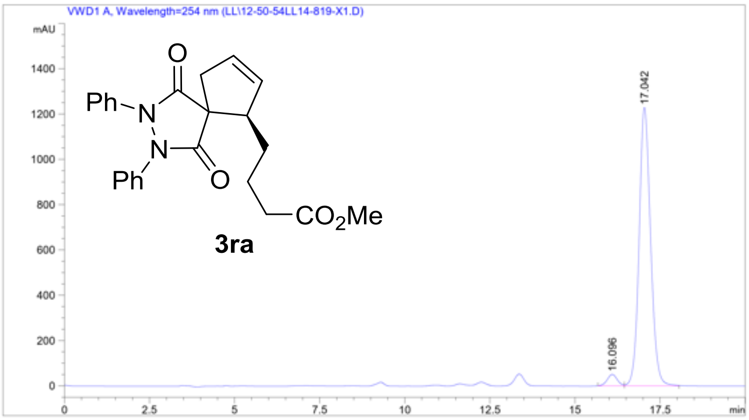


| Peak # | Ret. Time [min] | Type | Width [min] | Area [mAU*s] | Height [mAU] | Area % |
|---|---|---|---|---|---|---|
| 1 | 16.096 | HH | 0.3165 | 1053.40576 | 51.10772 | 3.5348 |
| 2 | 17.042 | HHA | 0.3570 | 2.87474e4 | 1228.49158 | 96.4652 |

**Supplementary Figure 68.** HPLC spectrum of **3ra**

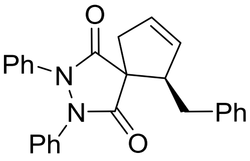


**3sa**

**(*R*)-6-benzyl-2,3-diphenyl-2,3-diazaspiro[4.4]non-7-ene-1,4-dione (3sa).** Following the *General Procedure I*: BnN(Me)$_2$ (2 equiv), 40 $^{o}$C, 36 h. **3sa** was obtained as a yellow oil, 22.9 mg, 58% yield, 88% ee. **[α]$_D$$^{20}$** = -1.5 (*c* 1.0, CHCl$_3$). **$^1$H NMR** (400 MHz, CDCl$_3$, ppm): $\delta$ 7.25 (t, *J* = 4.0 Hz, 2H), 7.23-7.16 (m, 7H), 7.15-7.05 (m, 4H), 5.81-5.61 (m, 2H), 4.02-3.88 (m, 1H), 3.15-2.93 (m, 4H). **$^{13}$C NMR** (100 MHz, CDCl$_3$, ppm): $\delta$ 174.5, 172.0, 139.0, 135.7, 135.6, 132.7, 129.3, 128.8, 128.8, 128.8, 127.1, 126.7, 126.7, 126.6, 122.8, 122.7, 56.7, 54.6, 44.1, 36.5. **HPLC analysis:** Daicel CHIRALPAK AD-H, *n*-hexane:*i*-PrOH = 80:20, flow rate = 1.0 mL/min, λ = 254 nm, retention time: $t_{major}$ = 12.4 min, $t_{minor}$ = 14.1 min. **HRMS** (ESI) calcd for $C_{26}H_{23}N_2O_2$ [M + H]$^+$: 395.1758, found 395.1754.

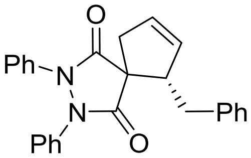

*ent*-**3sa**

**(*S*)-6-benzyl-2,3-diphenyl-2,3-diazaspiro[4.4]non-7-ene-1,4-dione (*ent*-3sa).** Following the *General Procedure I*: **L8** as ligand, DIPEA (2 equiv), 40 °C, 22 h. *ent*-**3sa** was obtained as a yellow oil (22.9 mg, 58% yield, 88% ee). $[\alpha]_D^{20}$ = +5.6 (*c* 1.0, $CHCl_3$). **HPLC analysis:** Daicel CHIRALPAK AD-H, *n*-hexane:*i*-PrOH = 80:20, flow rate = 1.0 mL/min, λ = 254 nm, retention time: $t_{major}$ = 12.7 min, $t_{minor}$ = 14.2 min. **HRMS** (ESI) calcd for $C_{26}H_{23}N_2O_2$ $[M + H]^+$: 395.1758, found 395.1754.

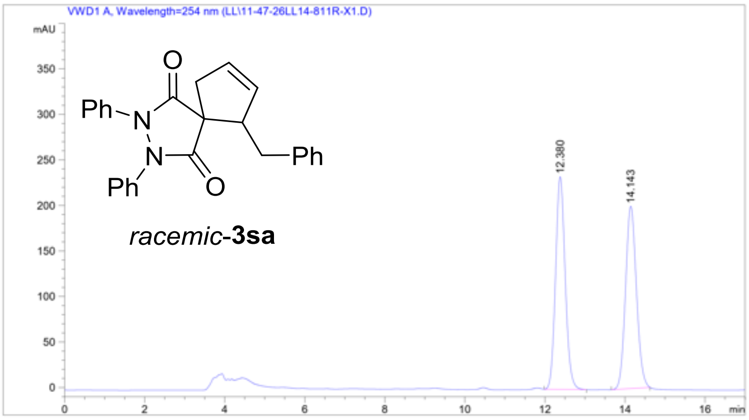


| Peak # | Ret. Time [min] | Type | Width [min] | Area [mAU*s] | Height [mAU] | Area % |
|---|---|---|---|---|---|---|
| 1 | 12.380 | BBA | 0.2574 | 3890.43774 | 233.50565 | 50.6389 |
| 2 | 14.143 | BBA | 0.2930 | 3792.26172 | 200.24002 | 49.3611 |

**Supplementary Figure 69.** HPLC spectrum of *racemic* **3sa**

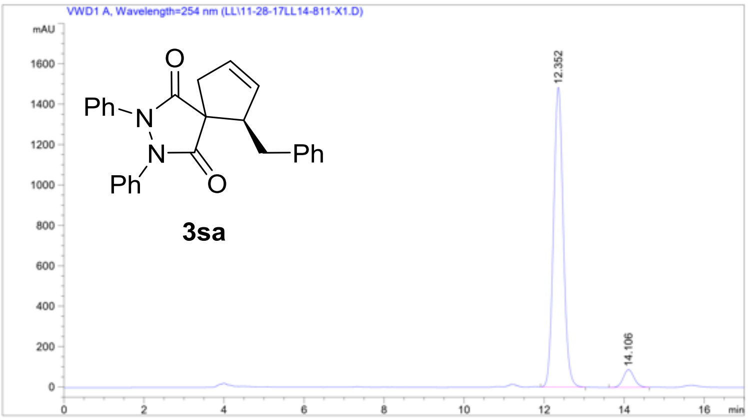


| Peak # | Ret. Time [min] | Type | Width [min] | Area [mAU*s] | Height [mAU] | Area % |
|---|---|---|---|---|---|---|
| 1 | 12.352 | BBA | 0.2629 | 2.50460e4 | 1483.75842 | 93.8063 |
| 2 | 14.106 | BBA | 0.2923 | 1653.69019 | 87.59360 | 6.1937 |

**Supplementary Figure 70.** HPLC spectrum of **3sa**

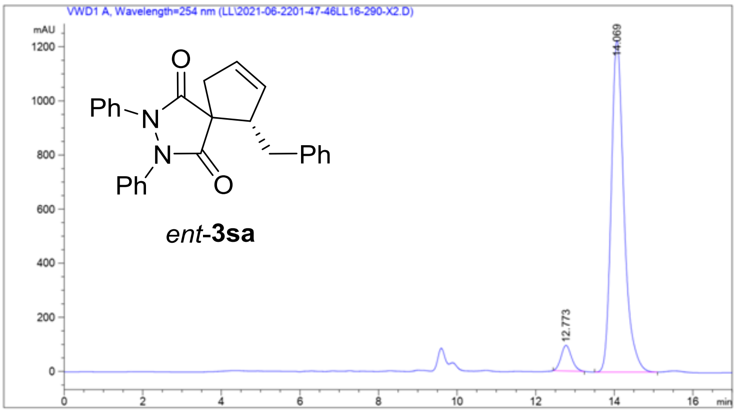


| Peak # | Ret. Time [min] | Type | Width [min] | Area [mAU*s] | Height [mAU] | Area % |
|---|---|---|---|---|---|---|
| 1 | 12.773 | HHA | 0.2867 | 1758.28003 | 94.65311 | 5.9631 |
| 2 | 14.069 | HB | 0.3457 | 2.77275e4 | 1226.83984 | 94.0369 |

**Supplementary Figure 71.** HPLC spectrum of *ent*-**3sa**

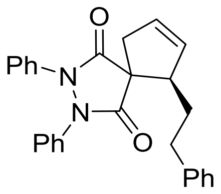

**3ta**

**(*R*)-6-phenethyl-2,3-diphenyl-2,3-diazaspiro[4.4]non-7-ene-1,4-dione (3ta).** Following the *General Procedure I*: $BnN(Me)_2$ (2 equiv), 30 °C, 36 h. **3ta** was obtained as a yellow oil (24.5mg, 60% yield, 88% ee). **$[\alpha]_D^{20}$** = -9.9 (*c* 1.0, $CHCl_3$). **$^1$H NMR** (400 MHz, $CDCl_3$, ppm): $\delta$ 7.38-7.26 (m, 8H), 7.25-7.20 (m, 2H), 7.20-7.13

(m, 3H), 7.12-7.04 (m, 2H), 5.81-5.64 (m, 2H), 3.54-3.41 (m, 1H), 3.10-2.92 (m, 2H), 2.72-2.52 (m, 2H), 2.07-1.89 (m, 2H). **$^{13}$C NMR** (100 MHz, $CDCl_3$, ppm): $\delta$ 174.6, 172.5, 141.4, 136.1, 132.0, 129.1, 129.1, 128.6, 128.4, 127.4, 126.8, 126.2, 122.5, 122.3, 56.5, 55.7, 42.9, 34.8, 32.7. **HPLC analysis:** Daicel CHIRALPAK IA, *n*-hexane:*i*-PrOH = 90:10, flow rate = 1.0 mL/min, λ = 254 nm, retention time: $t_{minor}$ = 17.1 min, $t_{major}$ = 18.5 min. **HRMS** (ESI) calcd for $C_{27}H_{24}N_2O_2$ $[M + H]^+$: 409.1901, found 409.1911.

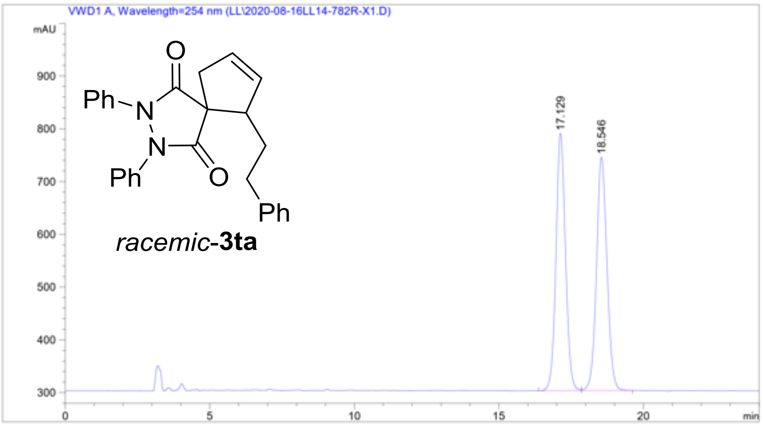


| Peak # | Ret. Time [min] | Type | Width [min] | Area [mAU*s] | Height [mAU] | Area % |
|---|---|---|---|---|---|---|
| 1 | 17.129 | BV | 0.3543 | 1.11306e4 | 487.65454 | 49.9959 |
| 2 | 18.546 | VBA | 0.3858 | 1.11325e4 | 442.03888 | 50.0041 |

**Supplementary Figure 72.** HPLC spectrum of *racemic* **3ta**

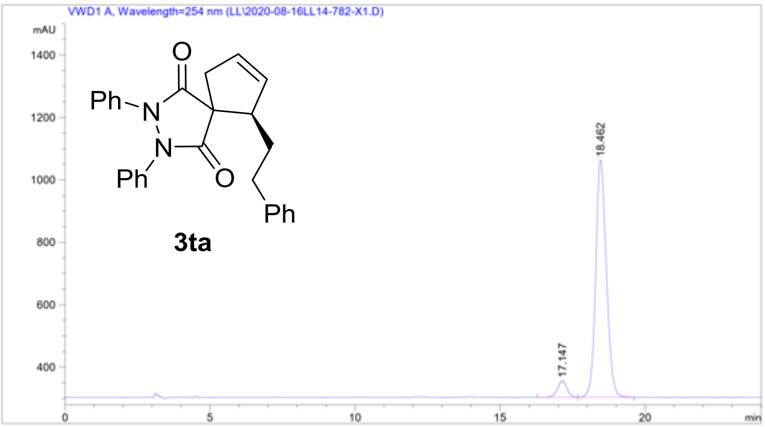


| Peak # | Ret. Time [min] | Type | Width [min] | Area [mAU*s] | Height [mAU] | Area % |
|---|---|---|---|---|---|---|
| 1 | 17.147 | BV | 0.3491 | 1190.98755 | 53.22528 | 5.8324 |
| 2 | 18.462 | VBA | 0.3875 | 1.92291e4 | 759.21527 | 94.1676 |

**Supplementary Figure 73.** HPLC spectrum of **3ta**

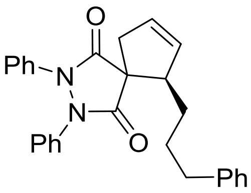

**3ua**

**(*R*)-2,3-diphenyl-6-(3-phenylpropyl)-2,3-diazaspiro[4.4]non-7-ene-1,4-dione (3ua).** Following the *General Procedure I*: BnN(Me)$_2$ (2 equiv), 30 $^o$C, 36 h. **3ua** was obtained as a yellow oil (26.2 mg, 62% yield, 94% ee). **[α]$_D^{20}$** = -6.6 (*c* 1.0, $CHCl_3$). **$^1$H NMR** (400 MHz, $CDCl_3$, ppm): *δ* 7.34-7.19 (m, 10H), 7.19-7.12 (m, 3H), 7.09-7.03 (m, 2H), 5.78-5.63 (m, 2H), 3.53-3.39 (m, 1H), 3.09-2.88 (m, 2H), 2.68-2.48 (m, 2H), 1.77-1.52 (m, 4H). **$^{13}$C NMR** (100 MHz, $CDCl_3$, ppm): *δ* 174.7, 172.5, 142.0, 136.0, 136.0, 132.1,129.1, 129.0, 128.5, 128.4, 127.3, 126.8, 122.5, 122.4, 56.6, 55.8, 42.7, 35.7, 30.4, 30.2. **HPLC analysis:** Daicel CHIRALPAK IA, *n*-hexane:*i*-PrOH = 90:10, flow rate = 1.0 mL/min, λ = 254 nm, retention time: $t_{major}$ = 12.4 min, $t_{minor}$ = 14.4 min. **HRMS** (ESI) calcd for $C_{28}H_{27}N_2O_2$ [M + H]$^+$: 423.2067, found 423.2072.

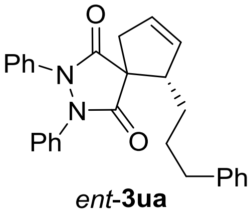

***ent*-3ua**

**(*S*)-2,3-diphenyl-6-(3-phenylpropyl)-2,3-diazaspiro[4.4]non-7-ene-1,4-dione (*ent*-3ua).** Following the *General Procedure II*: DIPEA (2 equiv), 40 $^o$C, 36 h. *ent*-**3ua** was obtained as a yellow oil (27.8 mg, 66% yield, 89% ee). **[α]$_D^{20}$** = +8.9 (*c* 1.0, $CHCl_3$). **HPLC analysis:** Daicel CHIRALPAK IA, *n*-hexane:*i*-PrOH = 90:10, flow rate = 1.0 mL/min, λ = 254 nm, retention time: $t_{major}$ = 12.7 min, $t_{minor}$ = 14.3 min. **HRMS** (ESI) calcd for $C_{28}H_{27}N_2O_2$ [M + H]$^+$: 423.2067, found 423.2066.

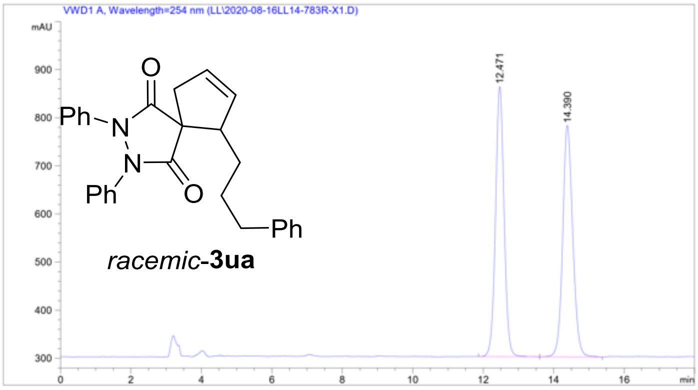


| Peak # | Ret. Time [min] | Type | Width [min] | Area [mAU*s] | Height [mAU] | Area % |
|---|---|---|---|---|---|---|
| 1 | 12.471 | BB | 0.2569 | 9463.67090 | 560.83331 | 49.8063 |
| 2 | 14.390 | BB | 0.3035 | 9537.27246 | 480.65152 | 50.1937 |

**Supplementary Figure 74.** HPLC spectrum of *racemic* **3ua**

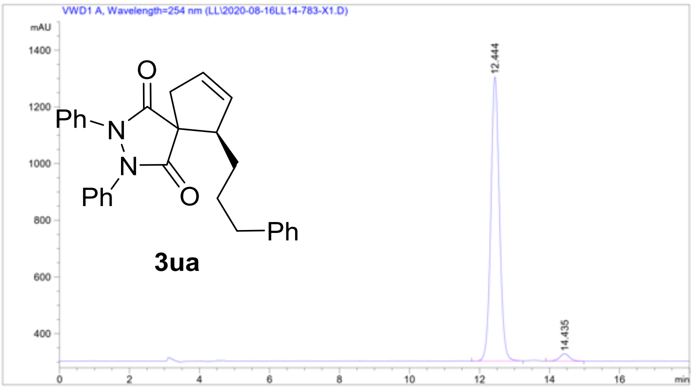


| Peak # | Ret. Time [min] | Type | Width [min] | Area [mAU*s] | Height [mAU] | Area % |
|---|---|---|---|---|---|---|
| 1 | 12.444 | BBA | 0.2666 | 1.70353e4 | 1000.94867 | 97.1859 |
| 2 | 14.435 | BBA | 0.2984 | 493.27020 | 25.42183 | 2.8141 |

**Supplementary Figure 75.** HPLC spectrum of **3ua**

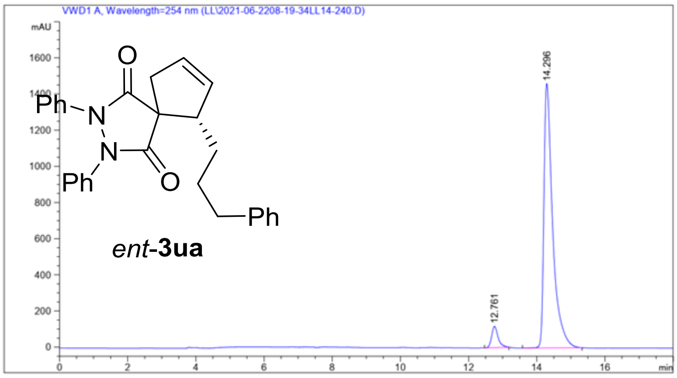


| Peak # | Ret. Time [min] | Type | Width [min] | Area [mAU*s] | Height [mAU] | Area % |
|---|---|---|---|---|---|---|
| 1 | 12.761 | BBA | 0.1997 | 1548.31445 | 117.49582 | 5.6229 |
| 2 | 14.296 | BBA | 0.2644 | 2.59875e4 | 1461.65881 | 94.3771 |

**Supplementary Figure 76.** HPLC spectrum of *ent*-**3ua**

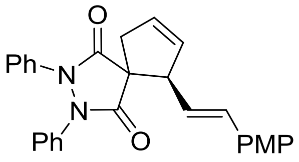


**3va**

**(*S*,*E*)-6-(4-methoxystyryl)-2,3-diphenyl-2,3-diazaspiro[4.4]non-7-ene-1,4-dione (3va).** Following the *General Procedure I*: $BnN(Me)_2$ (2 equiv), 10 $^oC$, 48 h. **3va** was obtained as a yellow oil (21.9 mg, 50% yield, 80% ee). $[\alpha]_D^{20}$ = -19.9 (*c* 1.0, $CHCl_3$). **$^1$H NMR** (400 MHz, $CDCl_3$, ppm): $\delta$ 7.27-7.23 (m, 4H), 7.21-7.13 (m, 7H), 7.11-7.03 (m, 1H), 6.87-6.77 (m, 2H), 6.46 (d, *J* = 16 Hz, 1H), 6.14 (dd, *J* = 16 Hz, 9.8 Hz, 1H), 5.92-5.84 (m, 1H), 5.67-5.59 (m, 1H), 4.26-4.15 (m, 1H), 3.81 (m, 3H), 3.14-2.96 (m, 2H). **$^{13}$C NMR** (100 MHz, $CDCl_3$, ppm): $\delta$ 174.3, 171.9, 159.5, 136.1, 135.7, 133.6, 131.0, 129.6, 129.3, 129.0, 128.9, 127.9, 126.9, 126.7, 124.3, 122.9, 122.8, 114.1, 61.6, 58.2, 55.4, 40.5. **HPLC analysis:** Daicel CHIRALPAK IA, *n*-hexane:*i*-PrOH = 80:20, flow rate = 1.0 mL/min, λ = 254 nm, retention time: $t_{major}$ = 14.2 min, $t_{minor}$ = 17.0 min. **HRMS** (ESI) calcd for $C_{28}H_{24}N_2O_3Na$ $[M + Na]^+$: 459.1673, found 459.1679.

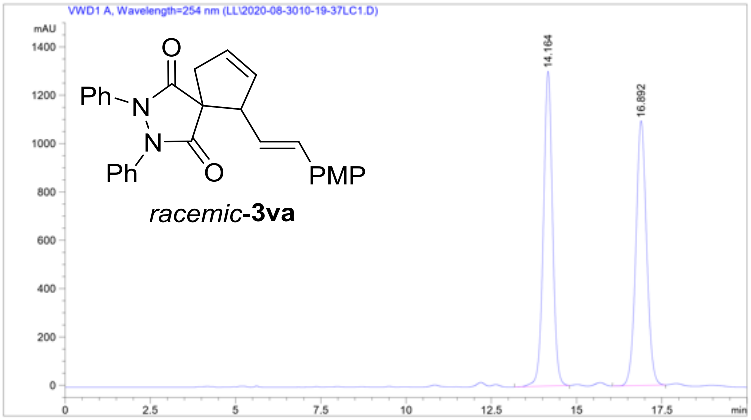


| Peak # | Ret. Time [min] | Type | Width [min] | Area [mAU*s] | Height [mAU] | Area % |
|---|---|---|---|---|---|---|
| 1 | 14.164 | BB | 0.2838 | 2.41989e4 | 1302.31152 | 50.2689 |
| 2 | 16.892 | BB | 0.3341 | 2.39401e4 | 1094.88599 | 49.7311 |

**Supplementary Figure 77.** HPLC spectrum of *racemic* **3va**

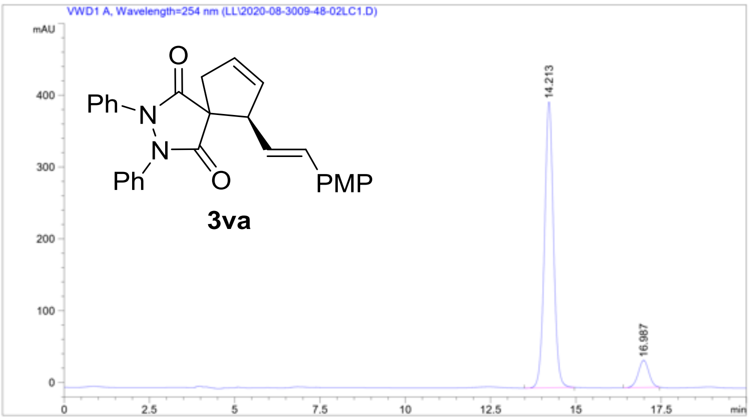


| Peak # | Ret. Time [min] | Type | Width [min] | Area [mAU*s] | Height [mAU] | Area % |
|---|---|---|---|---|---|---|
| 1 | 14.213 | BBA | 0.2801 | 7300.44775 | 397.81754 | 89.9367 |
| 2 | 16.987 | BBAS | 0.3309 | 816.87384 | 37.83904 | 10.0633 |

**Supplementary Figure 78.** HPLC spectrum of **3va**

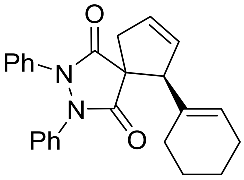

**3wa**

**(*S*)-6-(cyclohex-1-en-1-yl)-2,3-diphenyl-2,3-diazaspiro[4.4]non-7-ene-1,4-dione (3wa).** Following the *General Procedure I*: $BnN(Me)_2$ (2 equiv), 30 °C, 36 h. **3wa** was obtained as a yellow oil, 24.6mg, 64% yield, 85% ee. **$[\alpha]_D^{20}$** = -5.0 (*c* 1.0, $CHCl_3$). **$^1$H NMR** (400 MHz, $CDCl_3$, ppm): $\delta$ 7.36-7.22 (m, 8H), 7.21-7.10 (m, 2H), 5.89-5.79 (m, 1H), 5.70-5.56 (m, 2H), 4.10-3.96 (m, 1H), 3.05-2.87 (m, 2H), 2.15-1.74 (m, 4H), 1.30-1.38 (m, 4H). **$^{13}$C NMR** (100 MHz, $CDCl_3$, ppm): $\delta$ 174.7, 172.2, 136.2, 136.1, 134.8, 130.4, 129.0, 128.9, 126.9, 126.5, 126.4, 122.1, 122.0, 65.0, 57.6, 41.1, 28.2, 25.5, 23.0, 22.3. **HPLC analysis:** Daicel CHIRALPAK IA, *n*-hexane:*i*-PrOH = 90:10, flow rate = 1.0 mL/min, λ = 254 nm, retention time: $t_{minor}$ = 12.2 min, $t_{major}$ =15.6 min. **HRMS** (ESI) calcd for $C_{25}H_{25}N_2O_2$ $[M + H]^+$: 385.1911, found 385.1916.

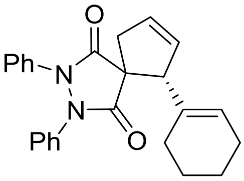

*ent*-**3wa**

**(*R*)-6-(cyclohex-1-en-1-yl)-2,3-diphenyl-2,3-diazaspiro[4.4]non-7-ene-1,4-dione(*ent*-3wa).** Following the *General Procedure II*: **L8** as ligand, DIPEA (2 equiv), 40 $^{o}$C, 22 h. *ent*-**3wa** was obtained as a yellow oil (23.8 mg, 62% yield, 93% ee). $[\alpha]_D^{20}$ = +10.7 (*c* 1.0, $CHCl_3$). **HPLC analysis:** Daicel CHIRALPAK IA, *n*-hexane:*i*-PrOH = 90:10, flow rate = 1.0 mL/min, λ = 254 nm, retention time: $t_{minor}$ = 12.1 min, $t_{major}$ =15.4 min. **HRMS** (ESI) calcd for $C_{25}H_{25}N_2O_2$ $[M + H]^+$: 385.1911, found 385.1910.

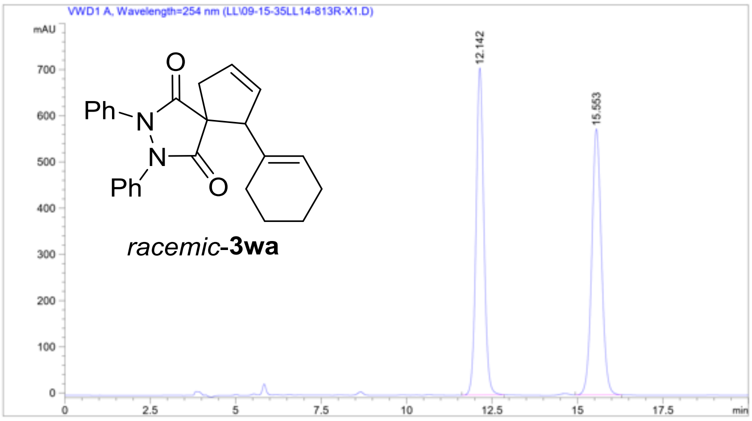


| Peak # | Ret. Time [min] | Type | Width [min] | Area [mAU*s] | Height [mAU] | Area % |
|---|---|---|---|---|---|---|
| 1 | 12.142 | BBA | 0.2419 | 1.12188e4 | 707.96887 | 49.7391 |
| 2 | 15.553 | BBA | 0.3038 | 1.13365e4 | 575.63953 | 50.2609 |

**Supplementary Figure 79.** HPLC spectrum of *racemic* **3wa**

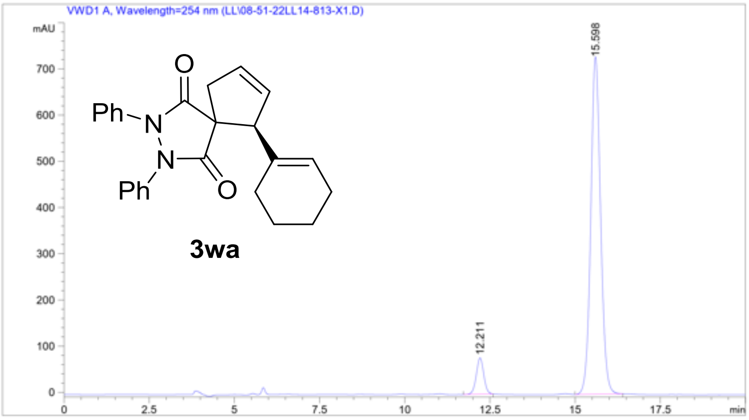


| Peak # | Ret. Time [min] | Type | Width [min] | Area [mAU*s] | Height [mAU] | Area % |
|---|---|---|---|---|---|---|
| 1 | 12.211 | BBA | 0.2339 | 1195.66711 | 78.42017 | 7.5669 |
| 2 | 15.598 | BBA | 0.3084 | 1.46056e4 | 730.11499 | 92.4331 |

**Supplementary Figure 80.** HPLC spectrum of **3wa**

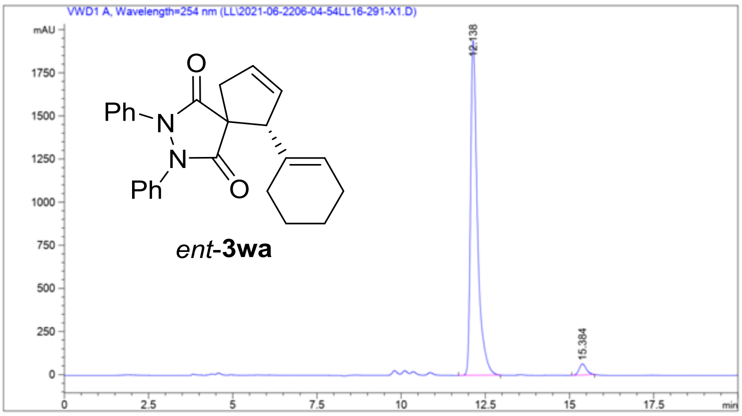


| Peak # | Ret. Time [min] | Type | Width [min] | Area [mAU*s] | Height [mAU] | Area % |
|---|---|---|---|---|---|---|
| 1 | 12.138 | BBA | 0.2128 | 2.75874e4 | 1940.94409 | 96.7003 |
| 2 | 15.384 | BBA | 0.2256 | 941.35443 | 63.99927 | 3.2997 |

**Supplementary Figure 81.** HPLC spectrum of *ent*-**3wa**

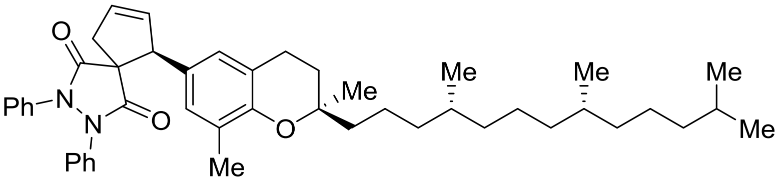

**3xa**

**6-((*R*)-2,8-dimethyl-2-((4*R*,8*R*)-4,8,12-trimethyltridecyl)chroman-6-yl)-2,3-diphenyl-2,3-diazaspiro[4.4]non-7-ene-1,4-dione (3xa).** Following the *General Procedure I*: (*from tocopherol*) $BnN(Me)_2$ (2 equiv), 40 °C, 48 h. **3xa** was obtained as a yellow oil (28.0 mg, 40% yield, > 20:1 dr). $[\alpha]_D^{20}$ = -9.8 (*c* 1.0, $CHCl_3$). **$^1$H NMR** (400 MHz, $CDCl_3$, ppm): $\delta$ 7.33-7.23 (m, 2H), 7.20-7.12 (m, 3H), 7.11-7.06 (m, 2H), 7.06-7.00 (m, 1H), 6.91-6.83 (m, 2H), 6.80-6.69 (m, 2H), 6.04-5.91 (m, 1H), 5.83-5.72 (m, 1H), 4.63 (s, 1H), 3.19-2.92 (m, 4H), 2.72-2.52 (m, 2H), 2.08 (s, 3H), 1.84-1.68 (m, 2H), 1.59-0.88 (m, 26H). **$^{13}$C NMR** (100 MHz, $CDCl_3$, ppm): $\delta$ 174.2, 171.4, 152.1, 135.8, 135.5, 131.2, 129.9, 128.8, 128.6, 127.2, 127.1, 126.6, 126.3, 126.2, 122.7, 122.4, 120.4, 76.2, 63.7, 63, 40.16, 39.9, 37.7, 37.6, 37.6, 37.4, 32.9, 31.4, 28.1, 24.9, 24.6, 24.3, 22.9, 22.8, 22.3, 21.2, 19.9, 18.8, 16.0. **HRMS** (ESI) calcd for $C_{46}H_{61}N_2O_3$[M + H]$^+$: 689.4677, found 689.4680.

## 1.5 Synthesis of The Double Hydroalkylation Product 4aa

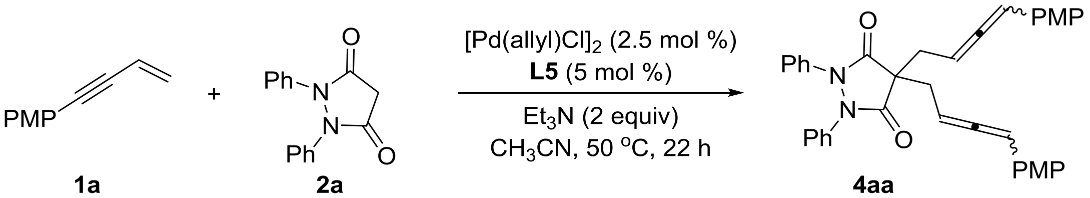


**1a** **2a** **4aa**

In an Ar-filled glovebox, dissolving the $[Pd(allyl)Cl]_2$ (0.91 mg, 2.5 mol %), and **L5** (3.2 mg, 5 mol %) in $CH_3CN$ (0.5 mL, 0.2 M) was stirred for 15 min at room temperature. Subsequently, conjugated enyne **1a** (0.12 mmol, 1.2 equiv), **2a** (0.1 mmol, 1 equiv), and $Et_3N$ (2 equiv) were added. The reaction mixture was stirred at 50 °C outside the glove box. The solution was concentrated in vacuo and the crude product was purified by column chromatography on silica gel (*n*-hexane:EtOAc = 95:5) to afford the product **4aa** as a white solid (26.9 mg, 79% yield). **4,4-bis(4-(4-methoxyphenyl)buta-2,3-dien-1-yl)-1,2-diphenylpyrazolidine-3,5-dione (4aa):** $^1$**H NMR** (500 MHz, $CDCl_3$, ppm): $\delta$ 7.45-6.99 (m, 18H), 6.82-6.73 (m, 4H), 6.05-5.90 (m, 2H), 5.53-5.45 (m, 2H), 3.79 (m, 6H), 2.85-2.71 (m, 4H). $^{13}$**C NMR** (150 MHz, $CDCl_3$, ppm): $\delta$ 206.1, 171.9, 171.6, 159.1, 135.7, 135.6, 129.2, 128.9, 128.9, 128.3, 126.6, 126.1, 126.0, 122.6, 122.5, 122.5, 114.3, 95.7, 88.5, 88.5, 55.4, 54.0, 53.9, 36.0. **HRMS** (ESI) calcd for $C_{37}H_{33}N_2O_4[M + H]^+$: 569.2435, found 569.2439.

## 1.6 Gram-scale Reaction

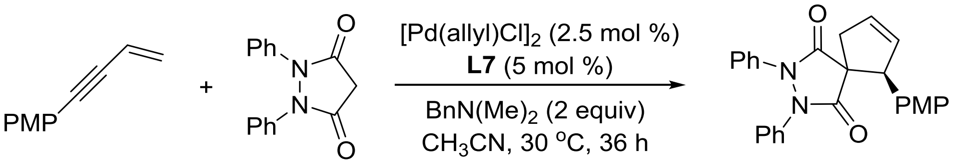


**1a**, 1.2 equiv **2a**, 4 mmol **3aa**, 1.1 g, 70%, 93% ee

In an Ar-filled glovebox, dissolving the $[Pd(allyl)Cl]_2$ (36.4 mg, 2.5 mol %), and **L7** (148 mg, 5 mol %) in $CH_3CN$ (0.5 mL, 0.2 M) was stirred for 15 min at room temperature. Subsequently, conjugated enyne **1a** (4.8 mmol, 1.2 equiv), **2a** (4 mmol, 1 equiv), and base were added. The reaction mixture was stirred outside the glove box. The solution was concentrated in vacuo and the crude product was purified by column chromatography on silica gel (*n*-hexane:EtOAc = 95:5) to afford the chiral spiro product **3aa** (1.1 g, 70% yield, 93% ee).

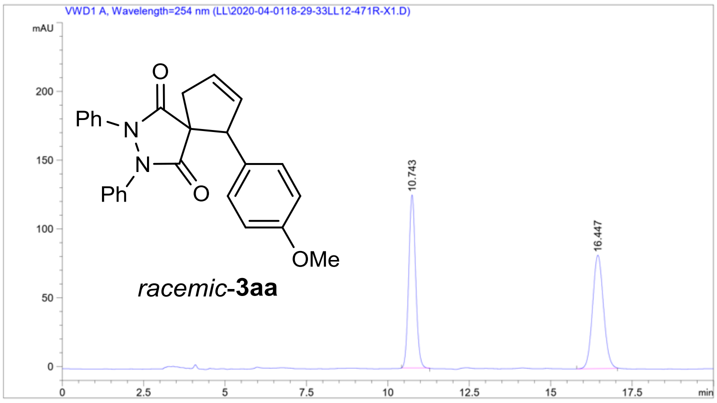


| Peak # | Ret. Time [min] | Type | Width [min] | Area [mAU*s] | Height [mAU] | Area % |
|---|---|---|---|---|---|---|
| 1 | 10.743 | HHAS | 0.2281 | 1855.06421 | 125.74533 | 49.0860 |
| 2 | 16.447 | HHA | 0.3624 | 1924.14746 | 82.40914 | 50.9140 |

**Supplementary Figure 82.** HPLC spectrum of *racemic* **3aa**

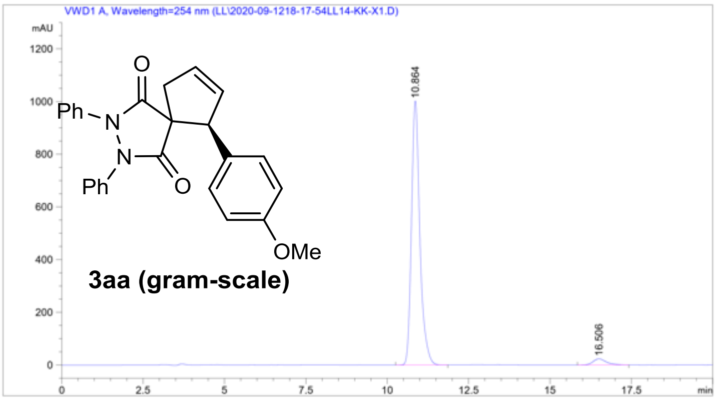


| Peak # | Ret. Time [min] | Type | Width [min] | Area [mAU*s] | Height [mAU] | Area % |
|---|---|---|---|---|---|---|
| 1 | 10.864 | BBA | 0.2692 | 1.79539e4 | 1001.25592 | 96.4705 |
| 2 | 16.506 | BBA | 0.4306 | 656.87634 | 23.18160 | 3.5295 |

**Supplementary Figure 83.** HPLC spectrum of **3aa** in gram-scale synthesis

## 1.7 Synthetic Transformations

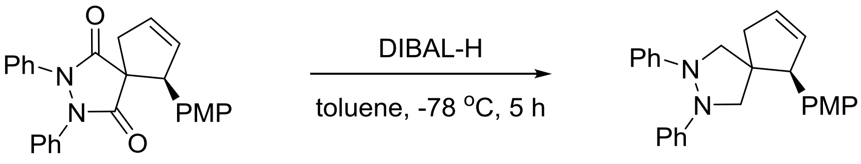


**3aa** (93% ee) **5** (68%, 92% ee)

***Transformation A:*** To a stirred solution of **3aa** (0.1 mmol, 1.0 equiv) was dissolved in toluene, DIBAL-H (16 equiv) was added at -78 $^{o}$C. The resulting mixture was

stirred at -78 °C for 5 h, the reaction was quenched by $Me_3NHCl$ (sat. aq. 1 mL) and was extracted with ethyl acetate (2×20 mL) and then washed with $H_2O$ and brine, dried over $Na_2SO_4$ and concentrated in vacuum. The crude product was purified by flash column chromatography on silica gel to yield the pure product **5** as a yellow oil (26.1 mg, 68% yield, 92% ee). **(*S*)-6-(4-methoxyphenyl)-2,3-diphenyl-2,3-diazaspiro[4.4]nonane (5)**: **$[\alpha]_D^{20}$** = -17.3 (*c* 1.0, $CHCl_3$). **$^1$H NMR** (400 MHz, $CDCl_3$, ppm): $\delta$ 7.38-7.11 (m, 5H), 6.98-6.55 (m, 10H), 6.01-5.68 (m, 2H), 3.91-3.56 (m, 6H), 3.50-2.94 (m, 2H), 2.56-2.14 (m, 2H). **$^{13}$C NMR** (100 MHz, $CDCl_3$, ppm): $\delta$ 158.7, 151.5, 134.4, 132.3, 130.4, 129.3, 129.0, 128.9, 119.5, 114.4, 113.9, 65.4, 59.1, 56.8, 55.9, 55.4, 42.9. **HPLC analysis:** Daicel CHIRALPAK AD-H, *n*-hexane:*i*-PrOH = 80:20, flow rate = 1.0 mL/min, λ = 254 nm, retention time: $t_{minor}$ = 5.2 min, $t_{major}$ = 6.8 min. **HRMS** (ESI) calcd for $C_{26}H_{27}N_2O$ $[M + H]^+$: 383.2115, found 383.2118.

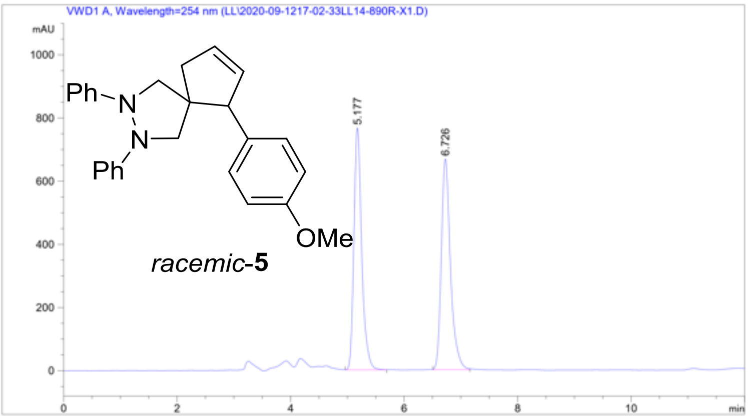


| Peak # | Ret. Time [min] | Type | Width [min] | Area [mAU*s] | Height [mAU] | Area % |
|---|---|---|---|---|---|---|
| 1 | 5.177 | FFA | 0.1459 | 7343.58105 | 765.79041 | 49.4821 |
| 2 | 6.726 | FFA | 0.1690 | 7497.29248 | 667.80310 | 50.5179 |

**Supplementary Figure 84.** HPLC spectrum of *racemic* **5**

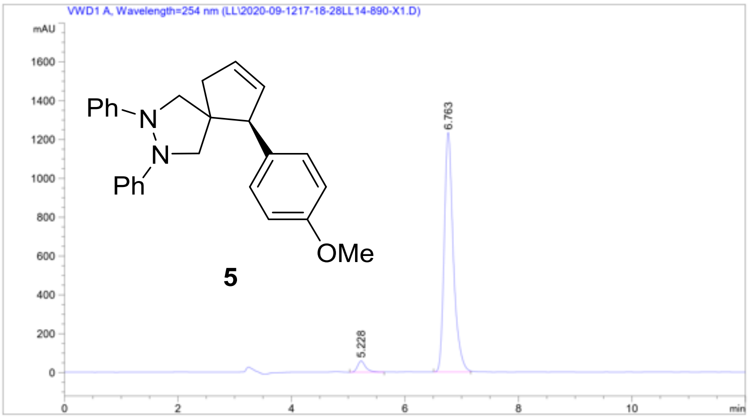


| Peak # | Ret. Time [min] | Type | Width [min] | Area [mAU*s] | Height [mAU] | Area % |
|---|---|---|---|---|---|---|
| 1 | 5.228 | FF | 0.1488 | 562.75769 | 57.20902 | 3.9333 |
| 2 | 6.763 | FFA | 0.1681 | 1.37446e4 | 1232.90967 | 96.0667 |

**Supplementary Figure 85.** HPLC spectrum of **5**

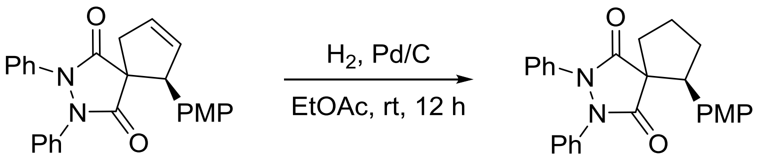


***Transformation B:*** To a stirred solution of **3aa** (0.1 mmol, 1.0 equiv) was dissolved in ethyl acetate and Pd/C (5 mmol%) was added. The resulting mixture was stirred at room temperature under $H_2$ atmosphere. After stirring for 12 h, the reaction was extracted with ethyl acetate (2×20 mL) and then washed with $H_2O$ and brine, dried over $Na_2SO_4$ and concentrated in vacuum. The crude product was purified by flash column chromatography on silica gel to yield the pure product **6** as a yellow oil (38.3 mg, 93% yield, 93% ee). **(*S*)-6-(4-methoxyphenyl)-2,3-diphenyl-2,3-diazaspiro-[4.4] -nonane-1,4-dione (6):** $[\alpha]_D^{20}$ = -1.5 (*c* 1.0, $CHCl_3$). **$^1$H NMR** (400 MHz, $CDCl_3$, ppm): $\delta$ 7.23-7.00 (m, 8H), 6.94-6.83 (m, 4H), 6.77-6.67 (m, 2H), 3.74 (s, 3H), 3.65 (dd, *J* = 12.9 Hz, *J* = 5.7 Hz, 1H), 2.77-2.55 (m, 1H), 2.47-2.17 (m, 3H), 2.16-1.98 (m, 2H). **$^{13}$C NMR** (100 MHz, $CDCl_3$, ppm): $\delta$ 173.6, 173.0, 159.5, 135.6, 135.4, 129.3, 128.7, 128.7, 126.7, 122.9, 122.7, 114.0, 60.2, 58.1, 55.4, 33.2, 30.7, 24.1. **HPLC analysis:** Daicel CHIRALPAK AD-H, *n*-hexane:*i*-PrOH = 80:20, flow rate = 1.0 mL/min, λ = 254 nm, retention time: $t_{major}$ = 8.9 min, $t_{minor}$ = 10.0 min. **HRMS** (ESI) calcd for $C_{26}H_{25}N_2O_3$ [M + H]$^+$: 413.1862, found 413.1860.

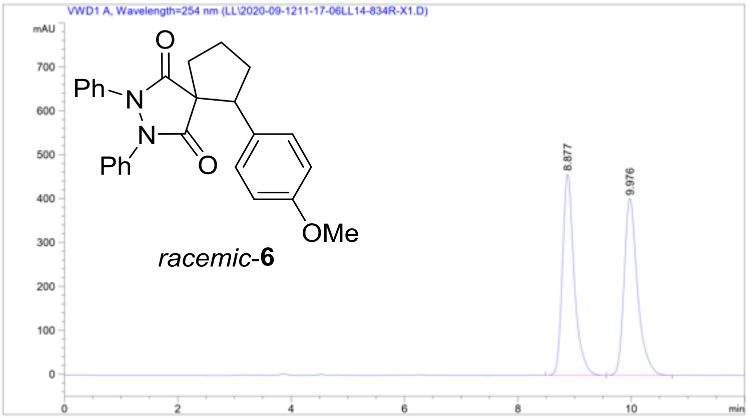


| Peak # | Ret. Time [min] | Type | Width [min] | Area [mAU*s] | Height [mAU] | Area % |
|---|---|---|---|---|---|---|
| 1 | 8.877 | BB | 0.2159 | 6592.88477 | 458.23337 | 49.9254 |
| 2 | 9.976 | BB | 0.2476 | 6612.57764 | 402.58710 | 50.0746 |

**Supplementary Figure 86.** HPLC spectrum of *racemic* **6**

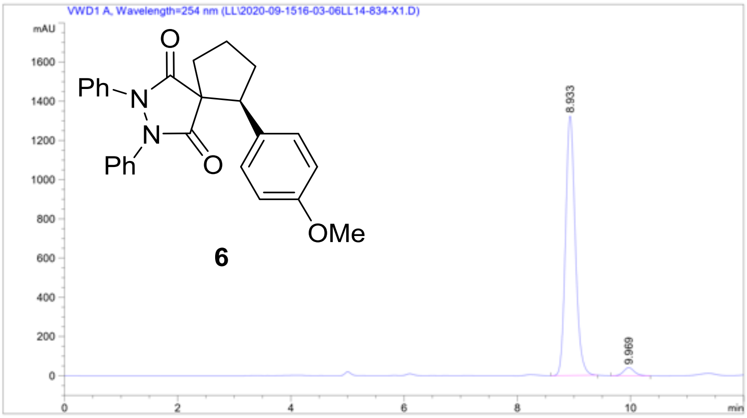


| Peak # | Ret. Time [min] | Type | Width [min] | Area [mAU*s] | Height [mAU] | Area % |
|---|---|---|---|---|---|---|
| 1 | 8.933 | BBA | 0.1843 | 1.57981e4 | 1323.56177 | 96.6506 |
| 2 | 9.969 | BBA | 0.2020 | 547.47656 | 41.46726 | 3.3494 |

**Supplementary Figure 87.** HPLC spectrum of **6**

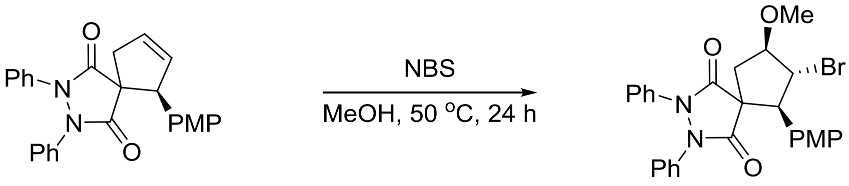


**3aa** (93% ee)

**7**
77%, 93% ee
> 20:1 dr

***Transformation C:*** To a stirred solution of **3aa** (0.1 mmol, 1.0 equiv) in $CH_3OH$ (1.0 mL) was added NBS (26.7 mg, 0.15 mmol, 1.5 equiv) at room temperature. After

stirring for 24 h at 50 °C, the reaction was extracted with ethyl acetate (2×20 mL) and then washed with $H_2O$ and brine, dried over $Na_2SO_4$ and concentrated in vacuum. The crude product was purified by flash column chromatography on silica gel to yield the pure product **7** as a yellow oil (20 mg, 77% yield, > 20:1 dr, 93% ee). **(6*S*,7*R*,8*R*)-7-bromo-8-methoxy-6-(4-methoxyphenyl)-2,3-diphenyl-2,3-diazaspiro [4.4]nonane-1,4-dione (7): $[\alpha]_D^{20}$** = -3.0 (*c* 1.0, $CHCl_3$). **$^1$H NMR** (400 MHz, $CDCl_3$, ppm): $\delta$ 7.45-7.36 (m, 2H), 7.21 (t, $J$ = 7.7 Hz, 7H), 7.17-7.01 (m, 6H), 6.86-6.73 (m, 4H), 4.70-4.57 (m, 2H), 4.28 (d, $J$ = 6.8 Hz 1H), 3.76 (s, 3H), 3.53 (s, 3H), 2.86 (dd, $J$ = 14.0 Hz, 6.0 Hz, 1H), 2.37 (dd, $J$ = 14.0 Hz, 4.7 Hz, 1H). **$^{13}$C NMR** (100 MHz, $CDCl_3$, ppm): $\delta$ 172.7, 171.4, 160.0, 135.7, 135.3, 131.9, 128.8, 127.0, 126.8, 126.1, 122.7, 122.7, 113.6, 89.8, 58.9, 58.0, 57.9, 56.1, 55.4, 37.5. **HPLC analysis:** Daicel CHIRALPAK IE-3, *n*-hexane:*i*-PrOH = 70:30, flow rate = 1.0 mL/min, λ = 254 nm, retention time: $t_{major}$ = 17.7 min, $t_{minor}$ = 25.9 min. **HRMS** (ESI) calcd for $C_{27}H_{26}BrN_2O_4$ $[M + H]^+$: 521.1070, found 521.1070.

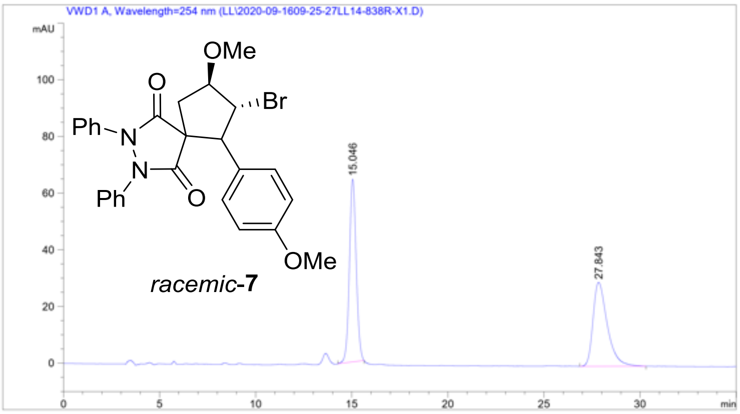


| Peak # | Ret. Time [min] | Type | Width [min] | Area [mAU*s] | Height [mAU] | Area % |
|---|---|---|---|---|---|---|
| 1 | 15.046 | BBAS | 0.3787 | 1584.40674 | 64.47995 | 50.8950 |
| 2 | 27.843 | BB | 0.7743 | 1528.68335 | 29.60989 | 49.1050 |

**Supplementary Figure 88.** HPLC spectrum of *racemic* **7**

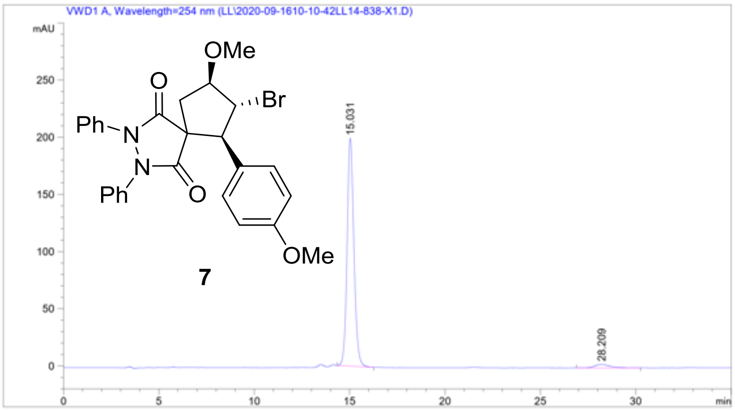


| Peak # | Ret. Time [min] | Type | Width [min] | Area [mAU*s] | Height [mAU] | Area % |
|---|---|---|---|---|---|---|
| 1 | 15.031 | BB | 0.3776 | 4912.13281 | 199.23215 | 96.7274 |
| 2 | 28.209 | BB | 0.7752 | 166.19331 | 3.07183 | 3.2726 |

**Supplementary Figure 89.** HPLC spectrum of **7**

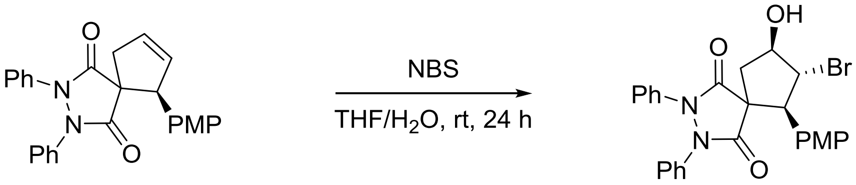


**3aa** (93% ee)

**8**
71%, 93% ee
> 20:1 dr

***Transformation D:*** To a stirred solution of **3aa** (0.1 mmol, 1.0 equiv) in THF/$H_2O$ (1.0 mL, v/v = 9:1) was added *N*-Bromosuccinimide (NBS, 21.4 mg, 0.12 mmol, 1.2 equiv) at room temperature. After stirring for 12 h at room temperature, the reaction was extracted with ethyl acetate (2×20 mL) and then washed with $H_2O$ and brine, dried over $Na_2SO_4$ and concentrated in vacuum. The crude product was purified by flash column chromatography on silica gel to yield the pure product **8** as a yellow oil (18.0 mg, 71% yield, > 20/1 dr, 93% ee). **(6*S*,7*R*,8*R*)-7-bromo-8-hydroxy-6-(4-methoxyphenyl)-2,3-diphenyl-2,3-diazaspiro[4.4]nonane-1,4-dione (8):** $[\alpha]_D^{20}$ = -6.0 (*c* 1.0, $CHCl_3$). $^1$**H NMR** (400 MHz, $CDCl_3$, ppm): $\delta$ 7.46 (d, *J* = 8.8 Hz, 2H), 7.25-7.07 (m, 6H), 7.02-6.90 (m, 4H), 4.80 (s, 1H), 4.53 (d, *J* = 5.5 Hz, 1H), 4.43 (m, *J* = 5.7 Hz 1H), 3.77 (m, 4H), 3.21 (dd, *J* = 14.4 Hz, 4.9Hz, 1H), 2.30 (d, *J* = 14.6 Hz 1H). $^{13}$**C NMR** (100 MHz, $CDCl_3$, ppm): $\delta$ 175.0, 170.7, 160.1, 135.1, 135.0, 132.1, 129.1, 128.9, 127.4, 127.1, 125.8, 123.0, 123.0, 113.7, 80.1, 60.3, 60.0, 59.0, 58.2, 55.4, 39.5. **HPLC analysis:** Daicel CHIRALPAK AD-H, *n*-hexane:*i*-PrOH = 80:20, flow rate = 1.0 mL/min, $\lambda$ = 254 nm, retention time: $t_{major}$ = 26.8 min, $t_{minor}$ = 36.2 min.

**HRMS** (ESI) calcd for $C_{26}H_{24}BrN_2O_4$ $[M + H]^+$: 507.0908, found 507.0914.

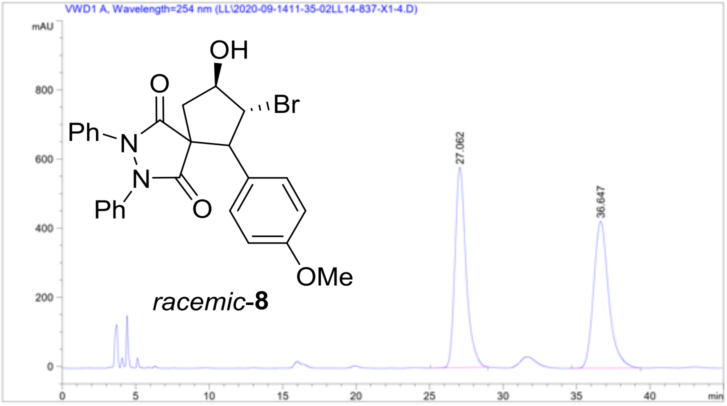


| Peak # | Ret. Time [min] | Type | Width [min] | Area [mAU*s] | Height [mAU] | Area % |
|---|---|---|---|---|---|---|
| 1 | 27.062 | BBA | 0.8028 | 3.03191e4 | 579.11407 | 50.0229 |
| 2 | 36.647 | BBA | 1.0879 | 3.02913e4 | 424.81845 | 49.9771 |

**Supplementary Figure 90.** HPLC spectrum of *racemic* **8**

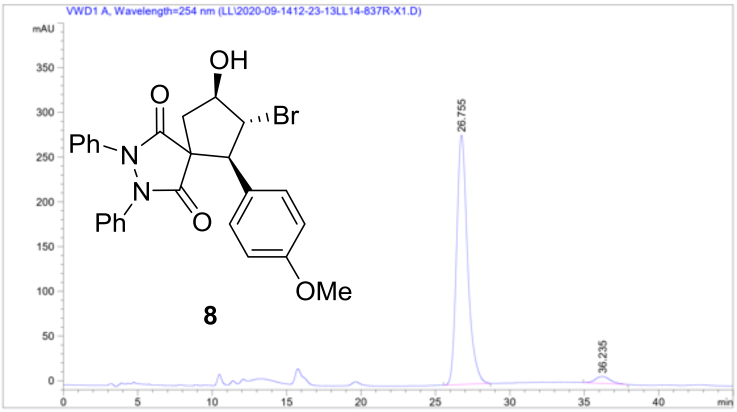


| Peak # | Ret. Time [min] | Type | Width [min] | Area [mAU*s] | Height [mAU] | Area % |
|---|---|---|---|---|---|---|
| 1 | 26.755 | BBA | 0.7765 | 1.43411e4 | 278.63968 | 96.3876 |
| 2 | 36.235 | BBA | 1.0605 | 537.48041 | 7.71723 | 3.6124 |

**Supplementary Figure 91.** HPLC spectrum of **8**

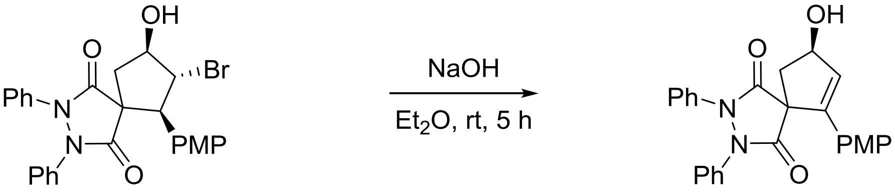


**8**
71%, 93% ee
>20/1 dr

**9**, 86%

***Transformation E:*** To a stirred solution of **8** (0.1 mmol, 1.0 equiv) in $Et_2O$ (1.0 mL) was added NaOH (3 equiv) at room temperature. After stirring for 5 h at room

temperature, the reaction was extracted with ethyl acetate (2×20 mL) and then washed with $H_2O$ and brine, dried over $Na_2SO_4$ and concentrated in vacuum. The crude product was purified by flash column chromatography on silica gel to yield the pure product **9** as a yellow oil (15.1 mg, 86% yield). **(*R*)-8-hydroxy-6-(4-methoxy phenyl)-2,3-diphenyl-2,3-diazaspiro[4.4]non-6-ene-1,4-dione (9): $[\alpha]_D^{20}$** = -0.5 (*c* 1.0, $CHCl_3$). **$^1$H NMR** (400 MHz, $CDCl_3$, ppm): $\delta$ 7.37-7.13 (m, 14H), 6.77 (d, *J* = 8.7 Hz 2H), 6.48 (d, *J* = 2.5 Hz, 1H), 4.97 (s, 1H), 3.77 (s, 3H), 3.02 (d, *J* = 12.1 Hz 1H), 2.88 (dd, *J* = 13.7 Hz, 6.3 Hz 1H), 2.41 (d, *J* = 13.8 Hz, 1H). **$^{13}$C NMR** (100 MHz, $CDCl_3$, ppm): $\delta$ 172.8, 171.3, 160.3, 144.0, 135.7, 135.5, 135.4, 129.3, 129.2, 128.3, 127.5, 127.3, 125.8, 122.9, 114.3, 75.7, 62.6, 55.4, 44.7. **HRMS** (ESI) calcd for $C_{26}H_{22}N_2O_4$ $[M + H]^+$: 427.0653, found 427.1652.

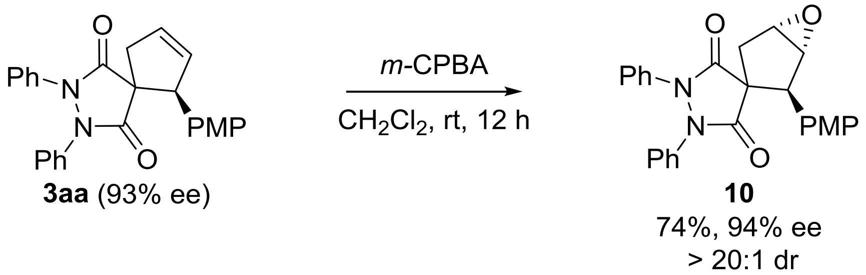


***Transformation F:*** To a stirred solution of **3aa** (0.1 mmol, 1.0 equiv) in $CH_2Cl_2$ (2.0 mL) was added *m*-CPBA (2 equiv) at room temperature. After stirring for 12 h at room temperature, the reaction was extracted with ethyl acetate (2×20 mL) and then washed with $H_2O$ and brine, dried over $Na_2SO_4$ and concentrated in vacuum. The crude product was purified by flash column chromatography on silica gel to yield the pure product **10** as a yellow oil (15.7 mg, 74% yield, > 20/1 dr, 94% ee). **(1*R*,2*S*,5*S*)-2-(4-methoxyphenyl)-1',2'-diphenyl-6-oxaspiro[bicyclo[3.1.0]hexane-3 ,4'-pyrazolidine]-3',5'-dione (10): $[\alpha]_D^{20}$** = -10.9 (*c* 1.0, $CHCl_3$). **$^1$H NMR** (400 MHz, $CDCl_3$, ppm): $\delta$ 7.34-7.29 (m, 2H), 7.28-7.22 (m, 2H), 7.19-7.00 (m, 6H), 6.88-6.74 (m, 4H), 3.93 (s, 1H), 3.84-3.79 (m, 1H), 3.77 (s, 3H), 3.74-3.69 (m, 1H), 2.75-2.51 (m, 2H). **$^{13}$C NMR** (100 MHz, $CDCl_3$, ppm): $\delta$ 172.5, 170.3, 159.8, 135.5, 130.3, 128.9, 128.6, 126.9, 126.9, 126.5, 122.7, 114.0, 59.9, 57.2, 56.2, 55.4, 54.4, 54.4, 35.4. **HPLC analysis:** Daicel CHIRALPAK AD-H, *n*-hexane:*i*-PrOH = 70:30,

flow rate = 1.0 mL/min, $\lambda$ = 254 nm, retention time: $t_{major}$ = 12.0 min, $t_{minor}$ = 26.5 min.

**HRMS** (ESI) calcd for $C_{26}H_{22}N_2O_4$ $[M + H]^+$: 427.0653, found 427.1653.

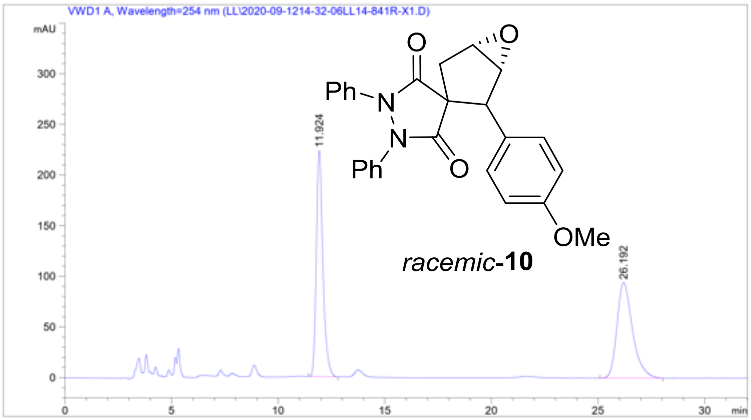


| Peak # | Ret. Time [min] | Type | Width [min] | Area [mAU*s] | Height [mAU] | Area % |
|---|---|---|---|---|---|---|
| 1 | 11.924 | BB | 0.3230 | 4765.36719 | 223.26941 | 49.9342 |
| 2 | 26.192 | BB | 0.7697 | 4777.92432 | 94.37104 | 50.0658 |

**Supplementary Figure 92.** HPLC spectrum of *racemic* **10**

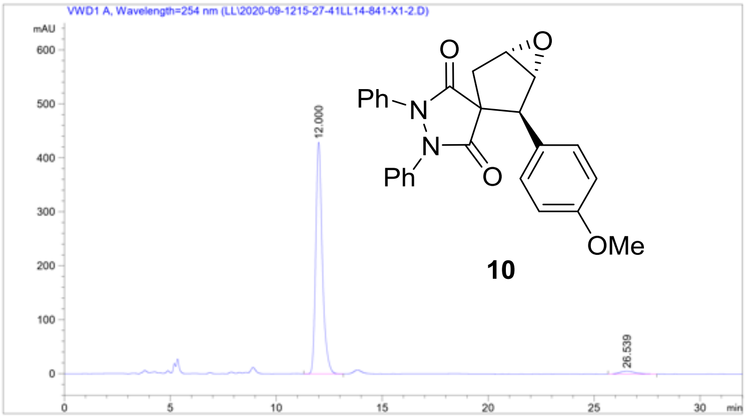


| Peak # | Ret. Time [min] | Type | Width [min] | Area [mAU*s] | Height [mAU] | Area % |
|---|---|---|---|---|---|---|
| 1 | 12.000 | BB | 0.3267 | 9264.09473 | 429.40616 | 97.2403 |
| 2 | 26.539 | BB | 0.7569 | 262.91483 | 5.30774 | 2.7597 |

**Supplementary Figure 93.** HPLC spectrum of **10**

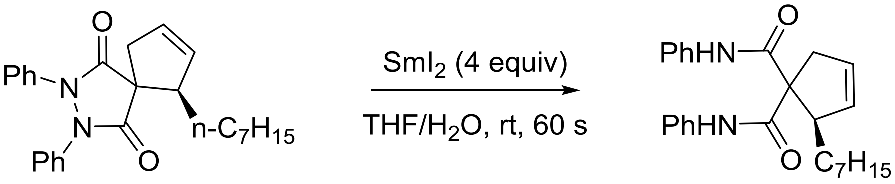


**3ma** (94% ee) **11** 77%, 94% ee

***Transformation G:*** To a stirred solution of **3aa** (0.1 mmol, 1.0 equiv) in THF/$H_2O$ (1.0 mL, v/v = 9:1) followed by a rapid injection of $SmI_2$ (in THF, typically 4.0 equiv) with vigorous stirring at room temperature. After stirring for 60 s at room temperature, the reaction was quenched by bubbling air through the reaction mixture, diluted with ethyl acetate (2×20 mL) and then washed with $H_2O$ and brine, dried over $Na_2SO_4$ and concentrated in vacuum. The crude product was purified by flash column chromatography on silica gel to yield the pure product **11** as a yellow oil (40.2 mg, 77% yield, 94% ee). **(*R*)-2-heptyl-*N*,*N'*-diphenylcyclopent-3-ene-1,1-dicarboxamide (11):** $^1$**H NMR** (400 MHz, $CDCl_3$, ppm): $\delta$ 9.79 (s, 1H), 8.58 (s, 1H), 7.57 (d, $J$ = 7.6 Hz, 2H), 7.47 (d, $J$ = 7.6 Hz, 2H), 7.33 (q, $J$ = 7.4 Hz, 4H), 7.20-7.09 (m, 2H), 6.03-5.85 (m, 2H), 3.59-3.46 (m, 1H), 3.38-3.18 (m, 2H), 1.77-1.61 (m, 1H), 1.49-1.11 (m, 12H), 0.83 (t, $J$ = 7.1 Hz, 3H). $^{13}$**C NMR** (100 MHz, $CDCl_3$, ppm): $\delta$ 172.0, 171.0, 137.9, 137.2, 134.7, 129.2, 128.9, 125.1, 124.7, 120.9, 120.6, 63.3, 56.4, 40.7, 31.8, 30.9, 29.7, 29.3, 28.3, 22.7, 14.2. **HPLC analysis:** Daicel CHIRALPAK OJ-H, *n*-hexane:*i*-PrOH = 90/10, flow rate = 1.0 mL/min, λ = 254 nm, retention time: $t_{minor}$ = 10.2 min, $t_{major}$ = 14.6 min. **HRMS** (ESI) calcd for $C_{26}H_{33}N_2O_2$ $[M + H]^+$: 405.2535, found 405.2537.

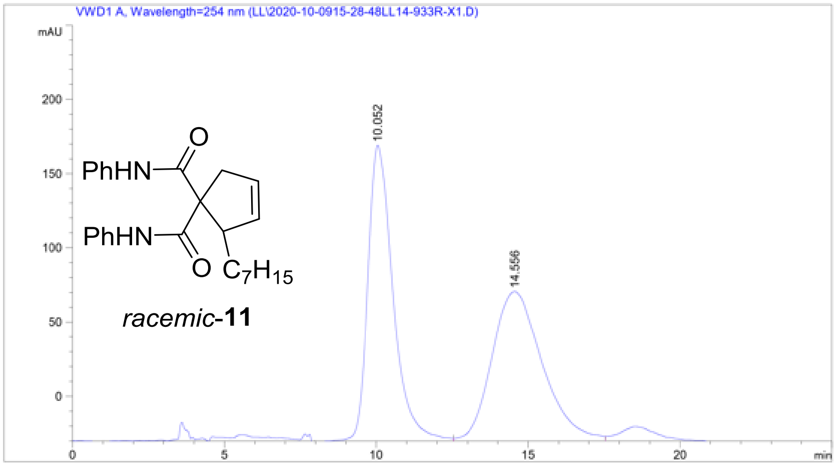


| Peak # | Ret. Time [min] | Type | Width [min] | Area [mAU*s] | Height [mAU] | Area % |
|---|---|---|---|---|---|---|
| 1 | 10.052 | BV | 0.8819 | 1.18771e4 | 200.85144 | 50.1628 |
| 2 | 14.556 | VF | 1.6840 | 1.18000e4 | 101.52287 | 49.8372 |

**Supplementary Figure 94.** HPLC spectrum of *racemic* **11**

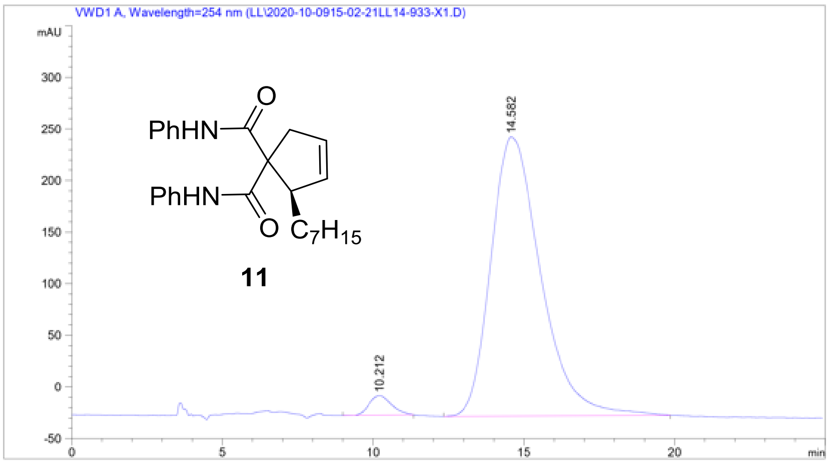


| Peak # | Ret. Time [min] | Type | Width [min] | Area [mAU*s] | Height [mAU] | Area % |
|---|---|---|---|---|---|---|
| 1 | 10.212 | VBAR | 0.7794 | 957.92493 | 18.64470 | 2.9865 |
| 2 | 14.582 | BBA | 1.7514 | 3.11172e4 | 270.23456 | 97.0135 |

**Supplementary Figure 95.** HPLC spectrum of **11**

# 2. Supplementary Discussion

## 2.1 Control Experiments

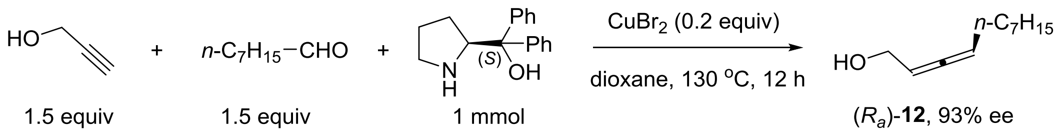


According to the known method[3], the reaction of $CuBr_2$ (45.1 mg, 0.2 mmol), (*S*)-diphenylprolinol (253.6 mg, 1.0 mmol), propargyl alcohol (84.5 mg, 1.5 mmol), and octanal (192.5 mg, 1.5 mmol) in dioxane (3 mL) afforded ($R_a$)-**12** as a yellow oil (93% ee, 60% yield). **($R_a$)-2,3-undecadien-1-ol ($R_a$-12): HPLC analysis:** Chiralcel As-H column, *n*-hexane:*i*-PrOH = 98.5/1.5, 1.0 mL/min, λ = 214 nm, $t_{major}$ = 6.3 min, $t_{minor}$ = 6.9 min.

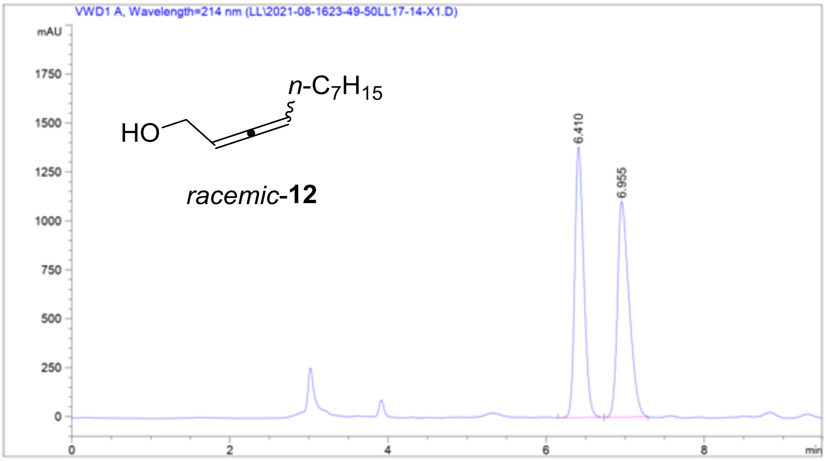


| Peak # | Ret. Time [min] | Type | Width [min] | Area [mAU*s] | Height [mAU] | Area % |
|---|---|---|---|---|---|---|
| 1 | 6.410 | BB | 0.1223 | 1.09359e4 | 1382.40918 | 49.0865 |
| 2 | 6.955 | BBA | 0.1540 | 1.13429e4 | 1102.89880 | 50.9135 |

**Supplementary Figure 96.** HPLC spectrum of *racemic* **12**

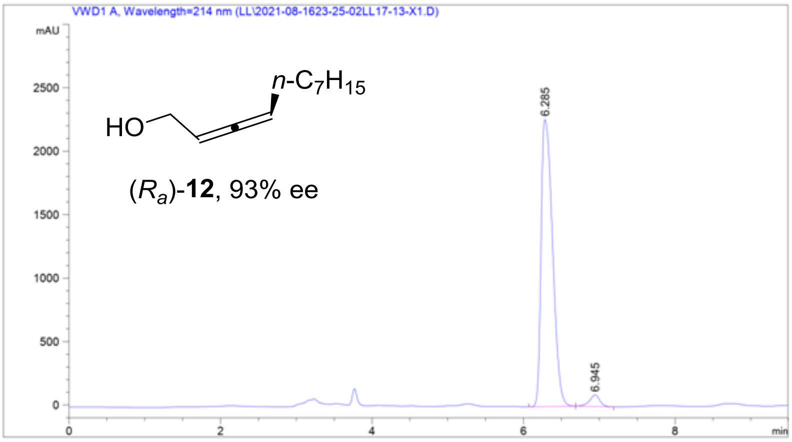


| Peak # | Ret. Time [min] | Type | Width [min] | Area [mAU*s] | Height [mAU] | Area % |
|---|---|---|---|---|---|---|
| 1 | 6.285 | BB | 0.1671 | 2.36741e4 | 2262.73486 | 96.5086 |
| 2 | 6.945 | BB | 0.1399 | 856.45569 | 91.73205 | 3.4914 |

**Supplementary Figure 97.** HPLC spectrum of (*R*$_a$)-**12**

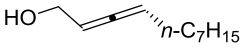


(*S*$_a$)-**12**, 94% ee

Following the above general procedure: $CuBr_2$ (45.1 mg, 0.2 mmol), (*R*)-diphenylprolinol (253.6 mg, 1.0 mmol), propargyl alcohol (84.5 mg, 1.5 mmol), and octanal (192.5 mg, 1.5 mmol) in dioxane (3 mL). **(*S*$_a$)-2,3-undecadien-1-ol (*S*$_a$-12)** was obtained as a yellow oil (60% yield, 94% ee). **HPLC analysis:** Chiralcel As-H column, *n*-hexane:*i*-PrOH = 98.5/1.5, 1.0 mL/min, λ = 214 nm, $t_{minor}$ = 6.4 min,

$t_{major}$ = 6.8 min.

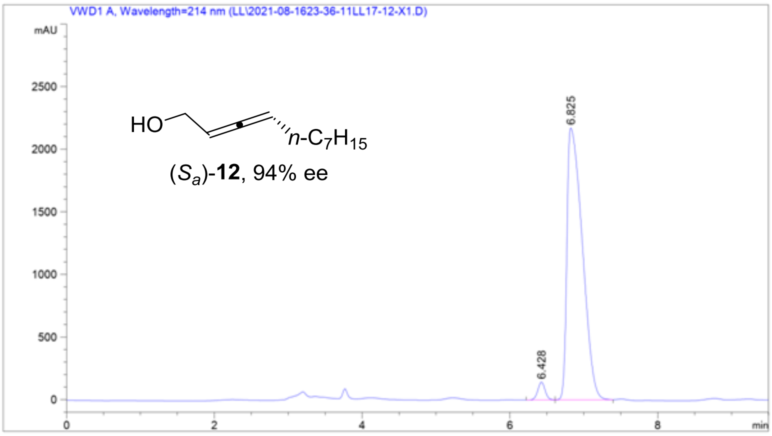


| Peak # | Ret. Time [min] | Type | Width [min] | Area [mAU*s] | Height [mAU] | Area % |
|---|---|---|---|---|---|---|
| 1 | 6.428 | BV | 0.1070 | 990.14966 | 142.52687 | 3.0624 |
| 2 | 6.825 | VB | 0.2324 | 3.13420e4 | 2171.14795 | 96.9376 |

**Supplementary Figure 98.** HPLC spectrum of (*S*$_a$)-**12**

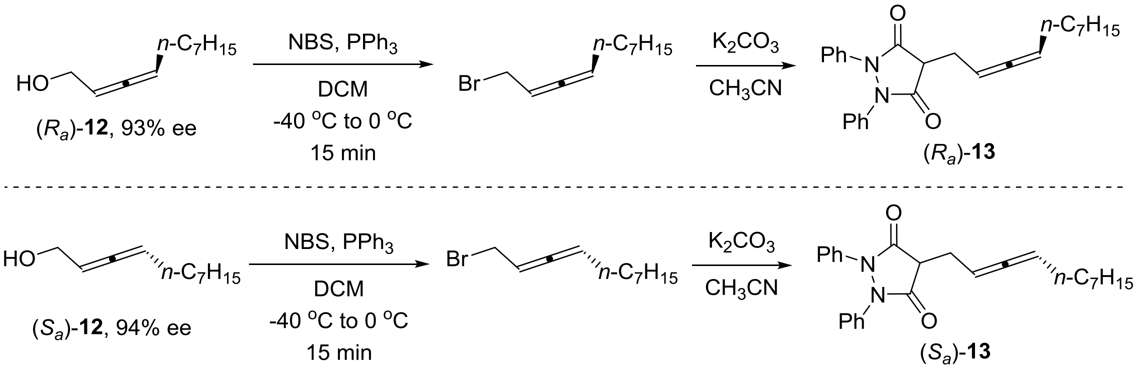


To a solution of allenyl alcohol and triphenylphosphine (1.3 equiv) in dichloromethane at -40 ℃ was added N-bromosuccinimide (1 equiv) in three equal portions 1 min apart. The resulting solution was transferred to a 0 ℃ ice bath and stirred for 15 min. The crude product was purified by flash column chromatography on silica gel to yield the pure product allenyl bromide (50% yield).

A round bottom flask was charged with $CH_3CN$, dimethyl malonate (3 equiv) and $K_2CO_3$ (3 equiv) and the resulting solution stirred for 60 min. The flask was cooled to 0 ℃ and the allenyl bromide (1 equiv) was added dropwise to give a concentration of 0.2 M (Injection pump, 0.2 mL/h). The solution was stirred 5 h. The

crude product was purified by flash column chromatography on silica gel to yield the pure product ($R_a$)-**13** or ($S_a$)-**13** (15% yield). **(*R*)-1,2-diphenyl-4-(undeca-2,3-dien-1-yl) pyrazolidine-3,5-dione (13): $^{1}$H NMR** (400 MHz, $CDCl_3$, ppm): $\delta$ 7.35-7.29 (m, 8H), 7.21-7.15 (m, 2H), 5.21-4.98 (m, 2H), 3.52 (t, $J$ = 4.9 Hz, 1H), 2.89-2.72 (m, 2H), 1.87-1.78 (m, 2H), 1.40-1.16 (m, 12H), 0.88 (t, $J$ = 6.9 Hz, 3H). **$^{13}$C NMR** (100 MHz, $CDCl_3$, ppm): $\delta$ 205.0, 169.7, 169.6, 136.1, 136.0, 129.0, 129.0, 126.8, 122.5, 93.4, 85.3, 77.5, 77.4, 77.2, 76.8, 46.5, 32.0, 29.2, 28.7, 27.3, 22.8, 14.2. **HRMS** (ESI) calcd for $C_{26}H_{31}N_2O_2$ $[M + H]^+$: 403.2380, found 403.2383.

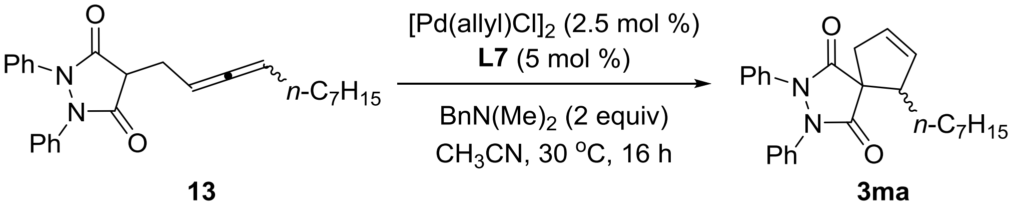


($R_a$)-**13** (95% ee) → ($R$)-**3ma:** 62%, 94% ee

($S_a$)-**13** (91% ee) → ($S$)-**3ma:** 65%, 92% ee

(±)-**13** (0% ee) → (±)-**3ma**: 61%, 0% ee

In an Ar-filled glovebox, dissolving the $[Pd(allyl)Cl]_2$ (0.91 mg, 2.5 mol %) and **L7** (3.7 mg, 5 mol %) in $CH_3CN$ (0.5 mL) was stirred for 15 min at room temperature. Subsequently, ($R_a$)-**13** (0.05 mmol) and base were added. The reaction mixture was stirred outside the glove box. The solution was concentrated in vacuo and the crude product was purified by column chromatography on silica gel (*n*-hexane:EtOAc = 95:5) to afford the chiral spiro product ($R$)-**3ma** (62%, 94% ee).

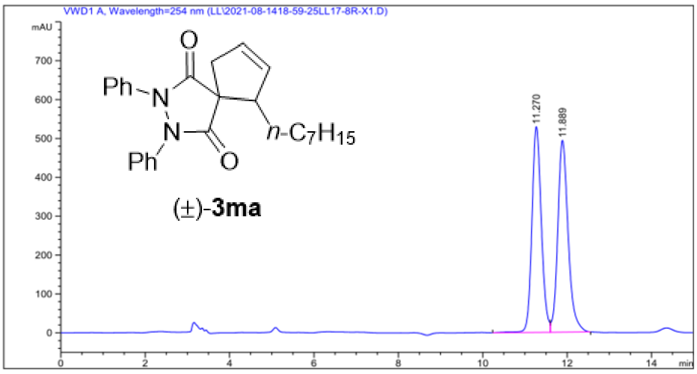


| Peak # | Ret. Time [min] | Type | Width [min] | Area [mAU*s] | Height [mAU] | Area % |
|---|---|---|---|---|---|---|
| 1 | 11.270 | VV R | 0.2391 | 8209.09961 | 528.83374 | 49.2901 |
| 2 | 11.889 | VBAS | 0.2608 | 8445.55957 | 493.15543 | 50.7099 |

**Supplementary Figure 99.** HPLC spectrum of *racemic* **3ma**

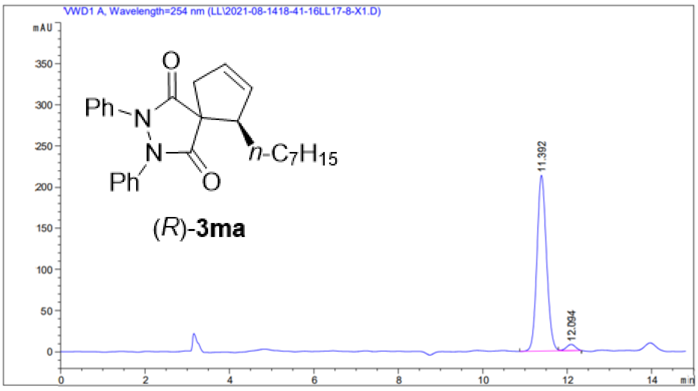


| Peak # | Ret. Time [min] | Type | Width [min] | Area [mAU*s] | Height [mAU] | Area % |
|---|---|---|---|---|---|---|
| 1 | 11.392 | BV R | 0.2414 | 3353.91846 | 213.40166 | 96.8267 |
| 2 | 12.094 | VBAE | 0.2320 | 109.91891 | 7.49780 | 3.1733 |

**Supplementary Figure 100.** HPLC spectrum of (*R*)-**3ma**

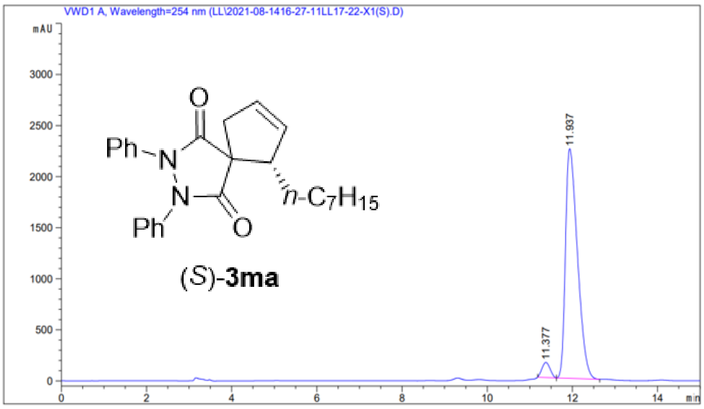


| Peak # | Ret. Time [min] | Type | Width [min] | Area [mAU*s] | Height [mAU] | Area % |
|---|---|---|---|---|---|---|
| 1 | 11.377 | HB | 0.2133 | 1936.63269 | 147.34885 | 4.2296 |
| 2 | 11.937 | HB | 0.3026 | 4.38507e4 | 2248.12207 | 95.7704 |

**Supplementary Figure 101.** HPLC spectrum of (*S*)-**3ma**

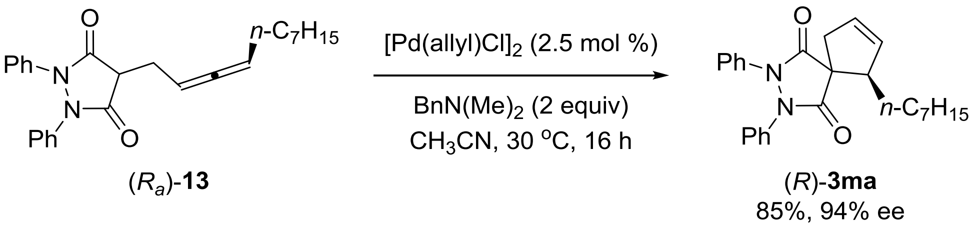


In an Ar-filled glovebox, dissolving the [Pd(allyl)Cl]$_2$ (0.45 mg, 2.5 mol %) and ($R_a$)-**13** (0.05 mmol) in $CH_3CN$ (0.3 mL) was stirred. Subsequently, BnN(Me)$_2$ (2 equiv) were added. The reaction mixture was stirred outside the glove box. The solution was concentrated in vacuo and the crude product was purified by column chromatography on silica gel (*n*-hexane:EtOAc = 95:5) to afford the chiral spiro product (*R*)-**3ma** (85% yield, 94% ee).

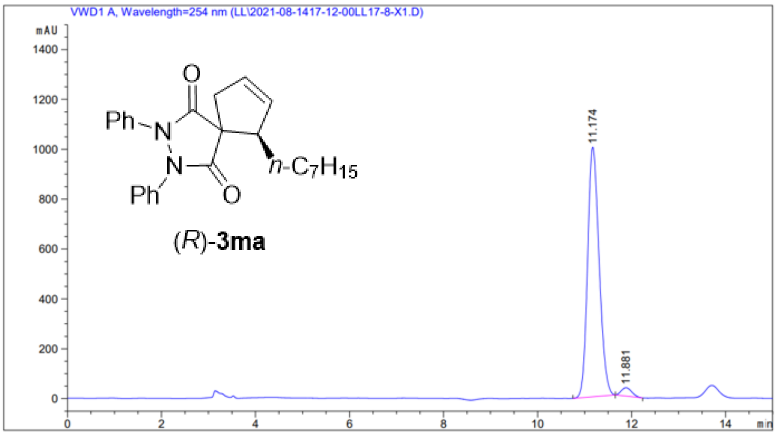


| Peak # | Ret. Time [min] | Type | Width [min] | Area [mAU*s] | Height [mAU] | Area % |
|---|---|---|---|---|---|---|
| 1 | 11.174 | BB | 0.2639 | 1.69797e4 | 1001.03668 | 97.0410 |
| 2 | 11.881 | BB | 0.2471 | 517.74310 | 33.72185 | 2.9590 |

**Supplementary Figure 102.** HPLC spectrum of chiral (*R*)-**3ma**

## 2.2 Proposed Reaction Mechanism

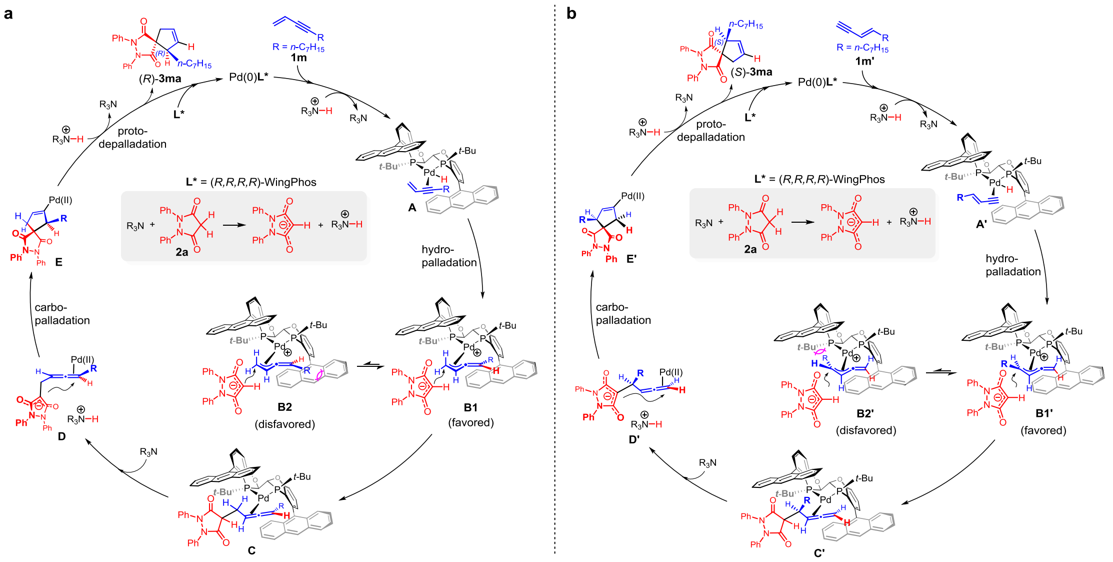

**Supplementary Figure 103. a** The cycloaddition reaction with 1,3-enyne **1m** having a terminal double bond. **b** The cycloaddition reaction with 1,3-enyne **1m'** having a terminal triple bond.

# 3. Supplementary Data

## 3.1 X-Ray Diffraction Data

The data of product **3ea** was collected by using copper irradiation source for the determination of absolute configuration of (*S*)-**3ea**.

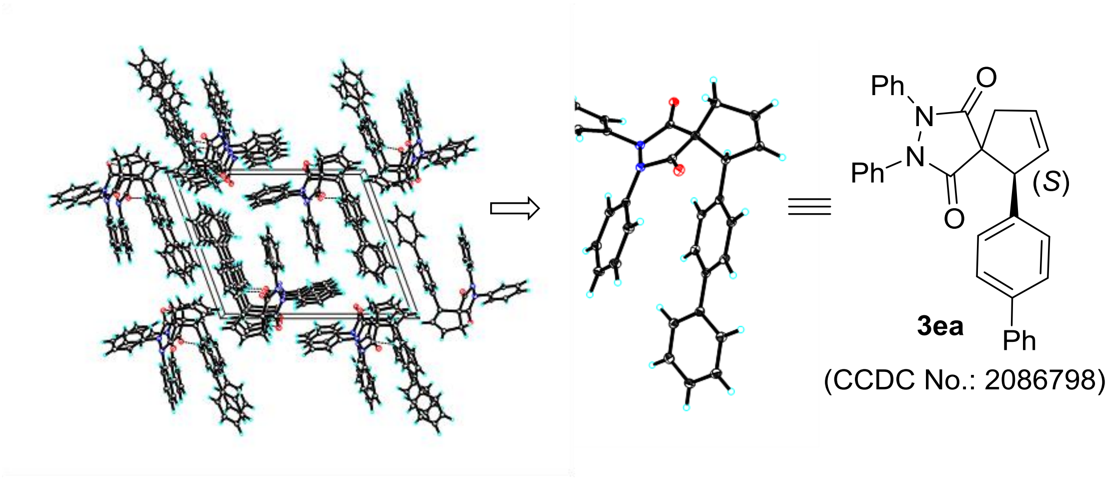


**Supplementary Figure 104.** ORTEP structure of (*S*)-**3ea**

**Supplementary Table 4.** Crystal data and structure refinement for (*S*)-**3ea**

| Identification code | global |
|---|---|
| Empirical formula | $C_{31}H_{24}N_2O_2$ |
| Formula weight | 456.52 |
| Temperature | 100(2) K |
| Wavelength | 1.54178 Å |
| Crystal system | Monoclinic |
| space group | P 1 21 1 |
| Unit cell dimensions | a = 12.6929(3) Å α= 90 °. |
| | b = 6.02310(10) Å β= 110.2810(10) °. |
| | c = 16.0222(3) Å γ = 90 °. |
| Volume | 1148.97(4) $Å^3$ |
| Z | 2 |
| Calculated density | 1.320 $Mg/m^3$ |
| Absorption coefficient | 0.654 $mm^{-1}$ |
| F(000) | 480 |
| Crystal size | 0.450 x 0.210 x 0.070 $mm^3$ |
| Theta range for data collection | 2.94 to 72.35 °. |
| Index ranges | -15<=h<=15, -7<=k<=6, -19<=l<=19 |
| Reflections collected | 25872 |
| Independent reflections | 4465 [R(int) = 0.0298] |
| Completeness to theta = 72.35 ° | 99.8 % |
| Absorption correction | Semi-empirical from equivalents |
| Max. and min. transmission | 0.96 and 0.83 |
| Refinement method | Data / restraints / parameters 4465 / 1 / 316 |
| Goodness-of-fit on F^2 | Final R indices [I>2sigma(I)] |
| Final R indices [I>2sigma(I)] | R1 = 0.0253, wR2 = 0.0628 |
| R indices (all data) | R1 = 0.0259, wR2 = 0.0632 |
| Absolute structure parameter | 0.09(5) |
| Largest diff. peak and hole | 0.156 and -0.134 e.$Å^3$ |

The data of product **3aj** was collected by using copper irradiation source for the determination of absolute configuration of (*S*)-**3aj**.

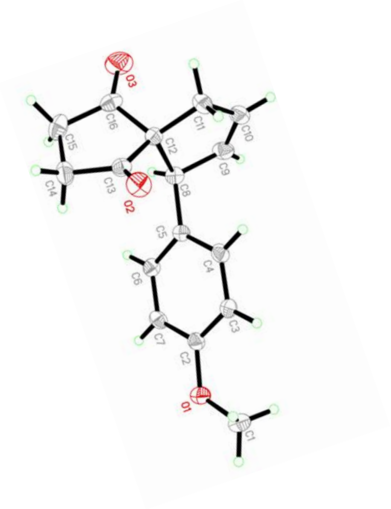

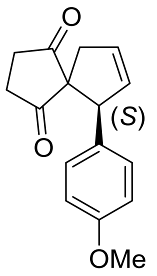


**3aj**

(CCDC No.: 2086799)

**Supplementary Figure 105.** ORTEP Structure of (*S*)-**3aj**

**Supplementary Table 5.** Crystal data and structure refinement for (*S*)-**3aj**.

| Identification code | (*S*)-**3aj** |
|---|---|
| Empirical formula | $C_{16}H_{16}O_3$ |
| Formula weight | 56.29 |
| Temperature | 293(2) K |
| Wavelength | 1.54178 A |
| Crystal system, space group | Orthorhombic, P2(1)2(1)2(1) |
| Unit cell dimensions | a = 5.791(4) A alpha = 90 deg.<br>b = 12.980(11) A beta = 90 deg.<br>c = 17.380(8) A gamma = 90 deg. |
| Volume | 1306.5(15) A^3 |
| Z, Calculated density | 4, 1.303 Mg/m^3 |
| F(000) | 544 |
| Crystal size | 0.260 x 0.200 x 0.160 mm |
| Theta range for data collection | 4.251 to 68.337 deg. |
| Limiting indices | -6<=h<=6, -15<=k<=15, -20<=l<=18 |
| Reflections collected / unique | 10014 / 2369 [R(int) = 0.1160] |

| | |
|---|---|
| Completeness to theta = 67.679 | 99.6 % |
| Max. and min. transmission | 0.891 and 0.841 |
| Refinement method | Full-matrix least-squares on F^2 |
| Data / restraints / parameters | 2369 / 0 / 174 |
| Goodness-of-fit on F^2 | 1.106 |
| Final R indices [I>2sigma(I)] | R1 = 0.0508, wR2 = 0.1164 |
| R indices (all data) | R1 = 0.0582, wR2 = 0.1217 |
| Absolute structure parameter | 0.2(3) |
| Extinction coefficient | 0.025(2) |
| Largest diff. peak and hole | 0.198 and -0.165 e.A^-3 |

The data of **3ah** was collected by using copper irradiation source for the determination of absolute configuration of (*S*)-**3ah**.

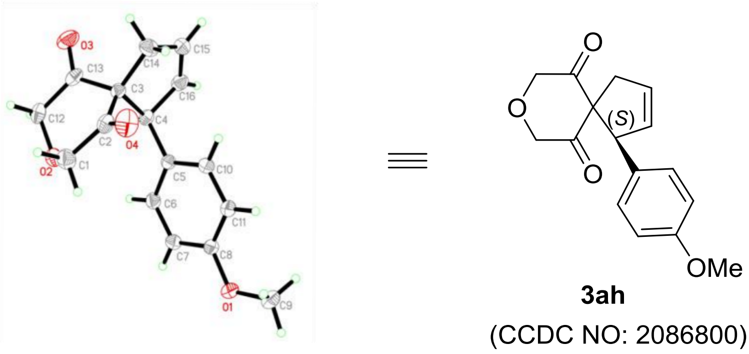


**3ah**

(CCDC NO: 2086800)

**Supplementary Figure 106.** ORTEP structure of (*S*)-**3ah**

**Supplementary Table 6.** Crystal data and structure refinement for (*S*)-**3ah**.

| Identification code | (*S*)-**3ah** |
|---|---|
| Empirical formula | $C_{16}H_{16}O_4$ |
| Formula weight | 272.29 |
| Temperature | 298(2) K |
| Wavelength | 1.54178 A |
| Crystal system, space group | Orthorhombic, P2(1)2(1)2(1) |
| Unit cell dimensions | a = 6.15340(10) A alpha = 90 deg.<br>b = 12.8331(3) A beta = 90 deg. |

| | |
|---|---|
| | c = 17.3936(4) A    gamma = 90 deg. |
| Volume | 1373.52(5) A^3 |
| Z, Calculated density | 4,    1.317 Mg/m^3 |
| Absorption coefficient | 0.776 mm^-1 |
| F(000) | 576 |
| Crystal size | 0.210 x 0.180 x 0.140 mm |
| Theta range for data collection | 4.281 to 59.029 deg. |
| Limiting indices | -6<=h<=6, -14<=k<=13, -19<=l<=18 |
| Reflections collected / unique | 6683 / 1949 [R(int) = 0.0355] |
| Completeness to theta = 59.029 | 98.9 % |
| Max. and min. transmission | 0.897 and 0.850 |
| Refinement method | Full-matrix least-squares on F^2 |
| Data / restraints / parameters | 1949 / 0 / 182 |
| Goodness-of-fit on F^2 | 1.132 |
| Final R indices [I>2sigma(I)] | R1 = 0.0349, wR2 = 0.0780 |
| R indices (all data) | R1 = 0.0403, wR2 = 0.0820 |
| Absolute structure parameter | 0.04(12) |
| Extinction coefficient | n/a |
| Largest diff. peak and hole | 0.111 and -0.135 e.A^-3 |

## 3.2 $^{1}H$ and $^{13}C$ NMR Spectra for New Compounds

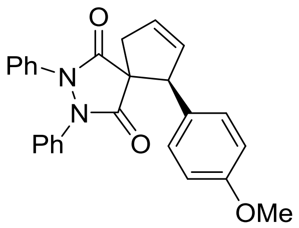


**3aa**

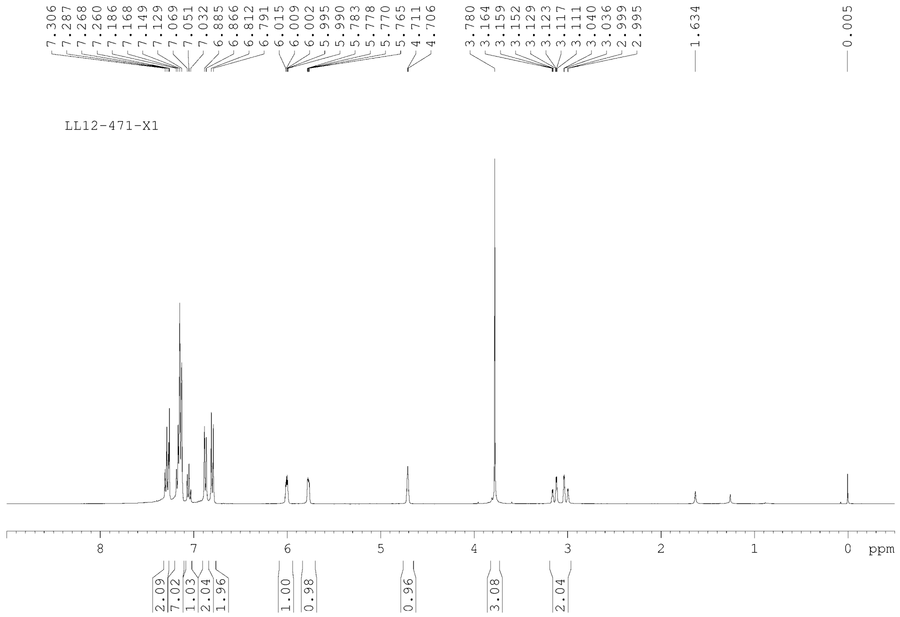


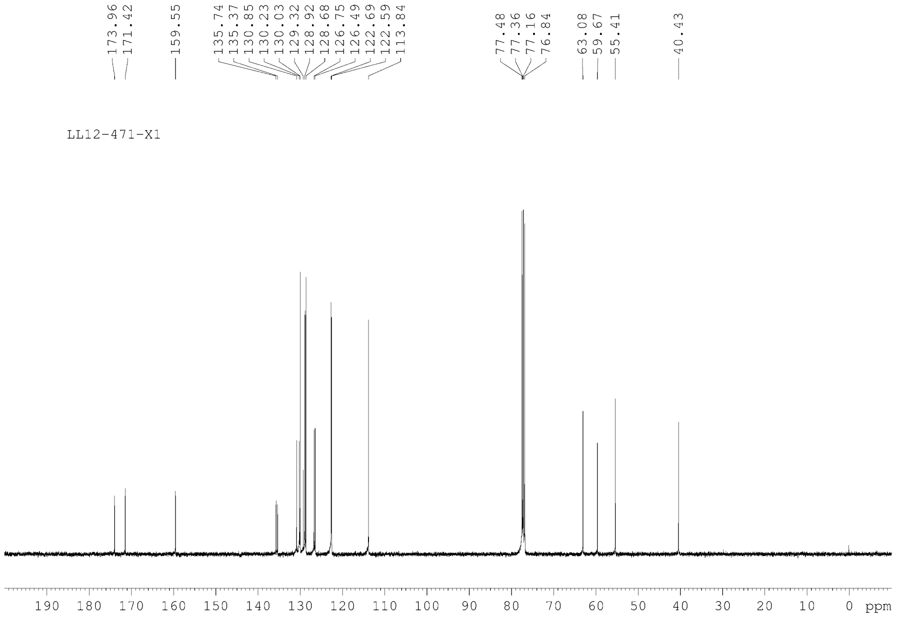


**Supplementary Figure 107**. $^{1}H$ and $^{13}C$ NMR spectra of product **3aa**

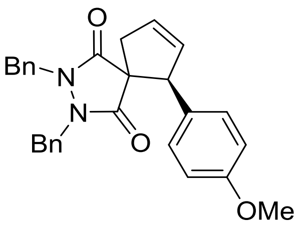


**3ab**

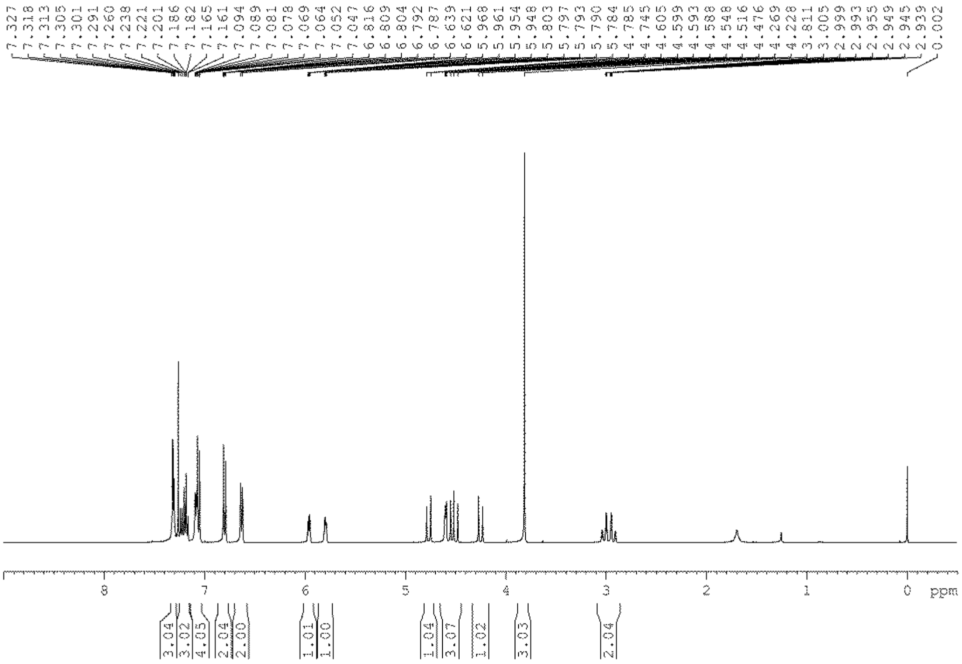


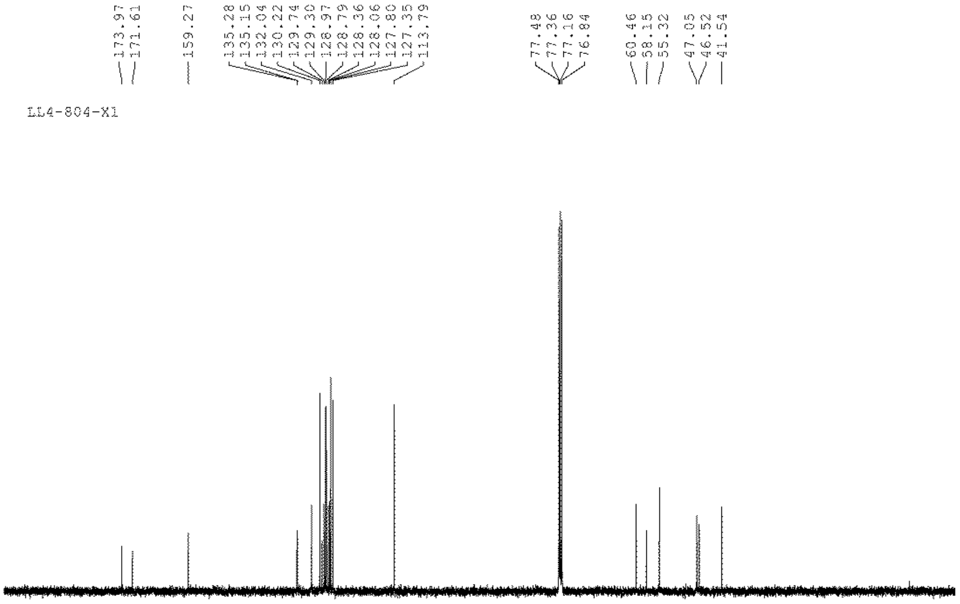


**Supplementary Figure 108**. $^{1}H$ and $^{13}C$ NMR spectra of product **3ab**

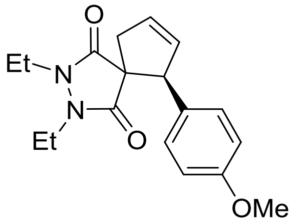


**3ac**

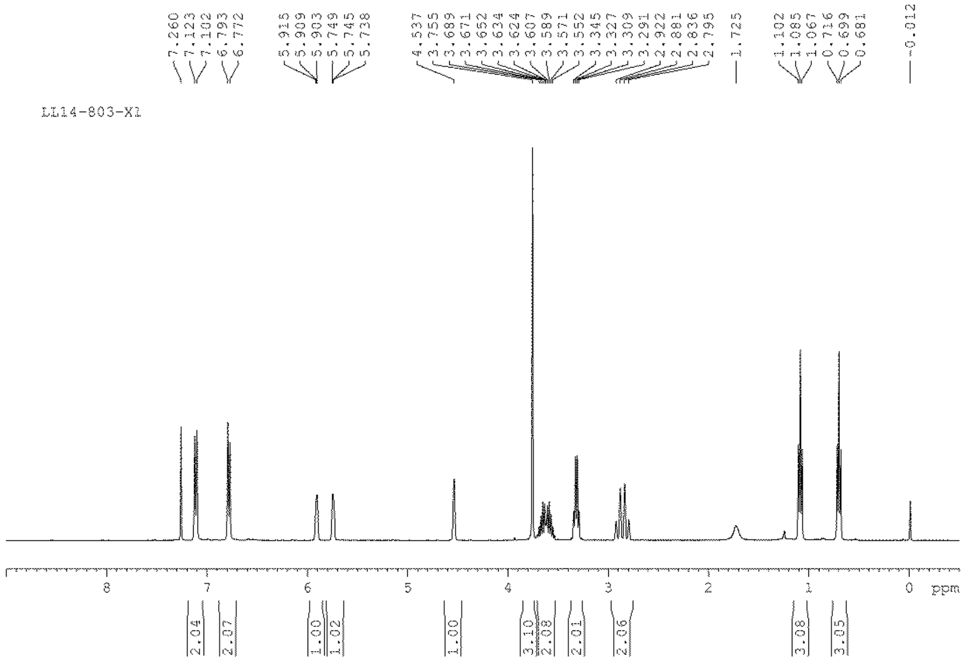


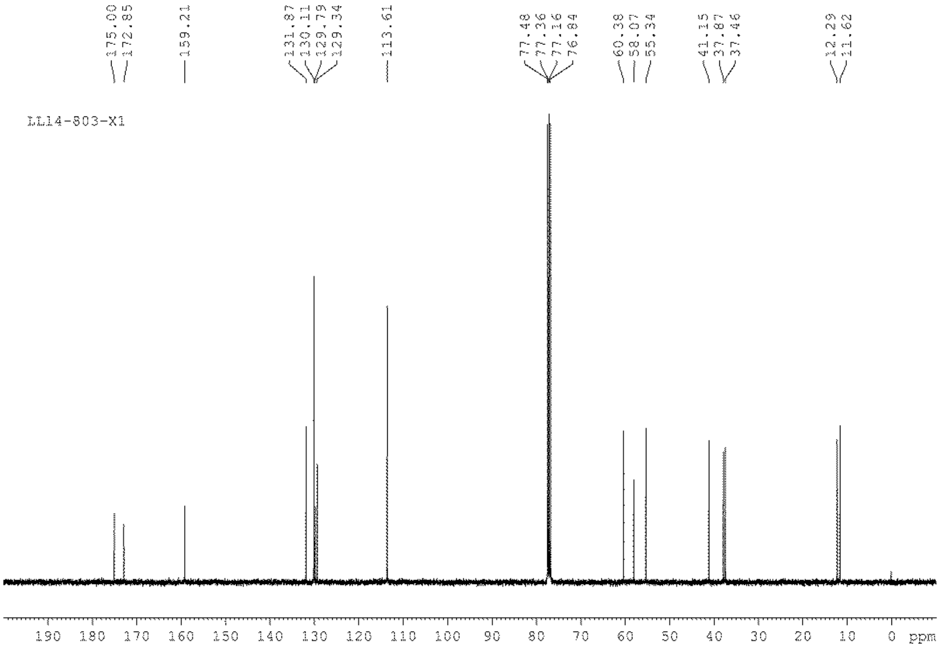


**Supplementary Figure 109**. $^{1}$H and $^{13}$C NMR spectra of product **3ac**

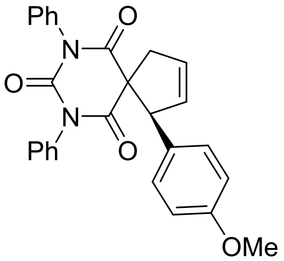


**3ad**

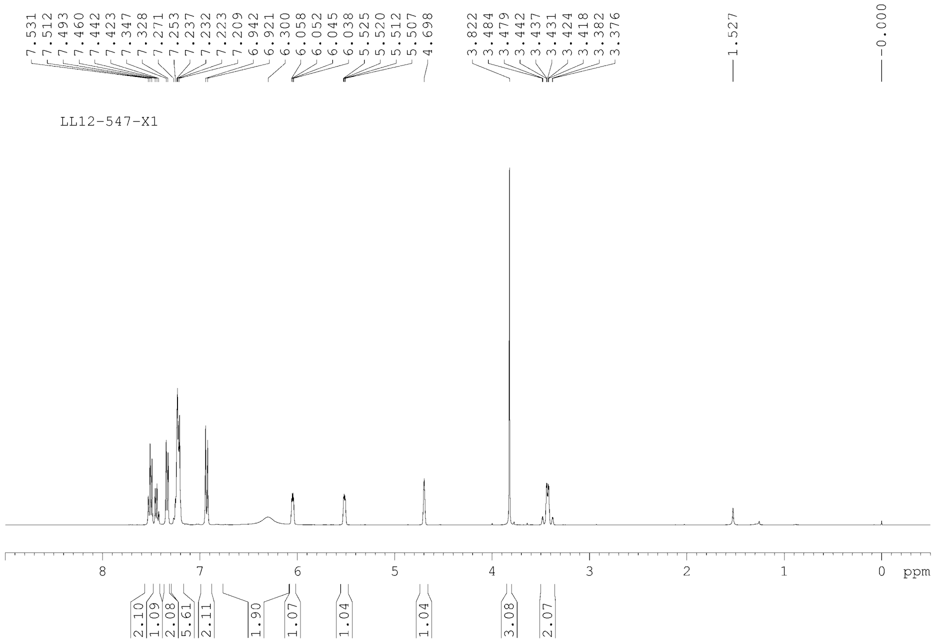


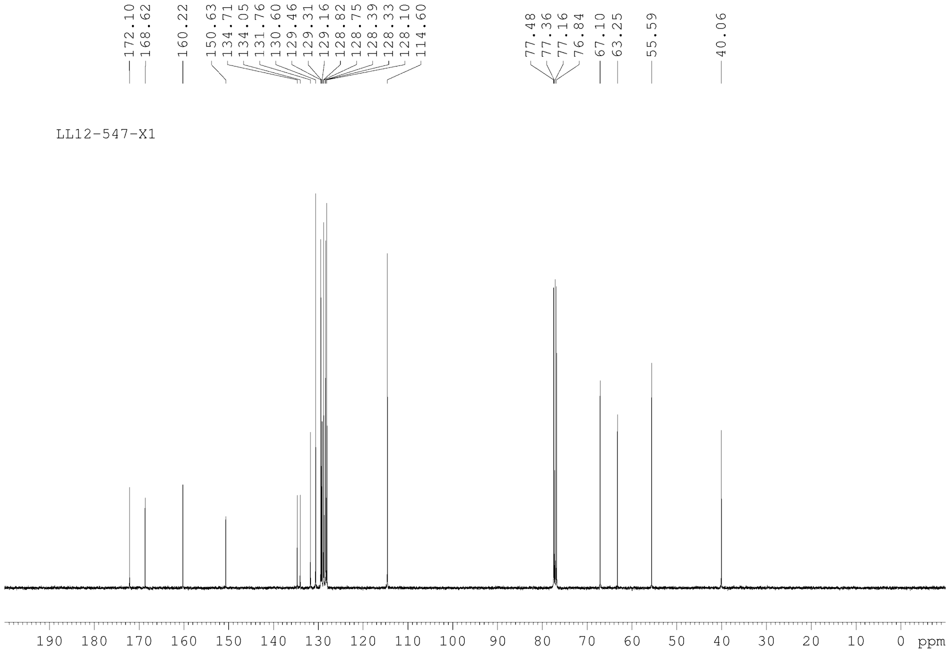


**Supplementary Figure 110**. $^{1}$H and $^{13}$C NMR spectra of product **3ad**

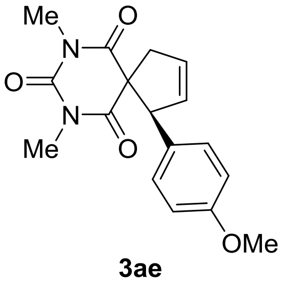


**3ae**

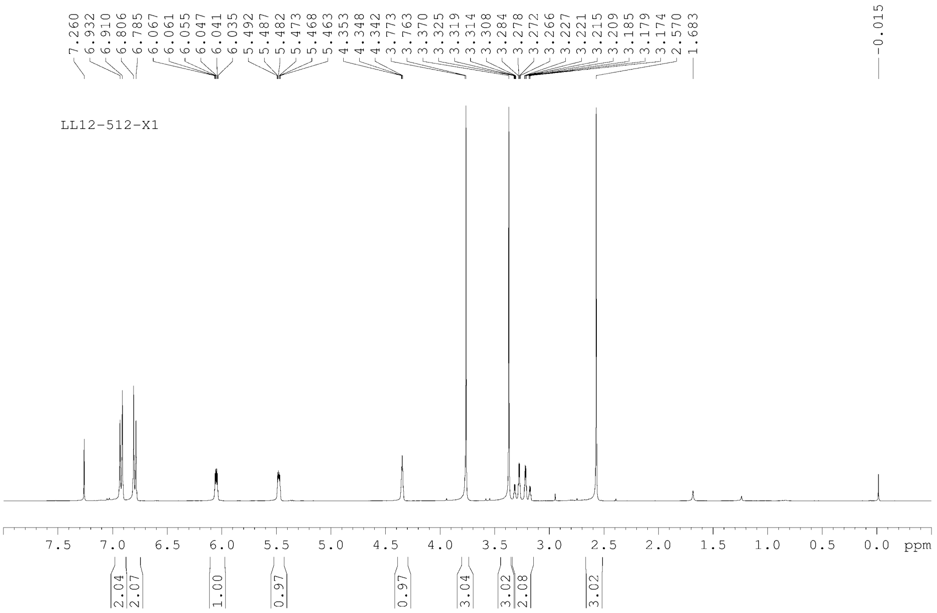


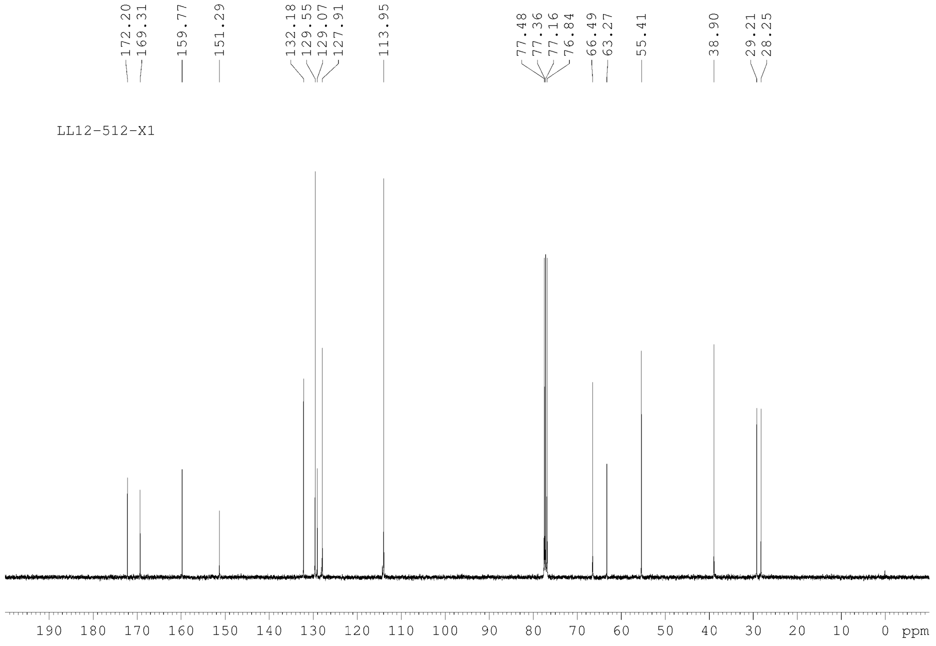


**Supplementary Figure 111**. $^{1}$H and $^{13}$C NMR spectra of product **3ae**

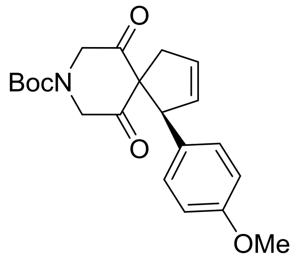


3af

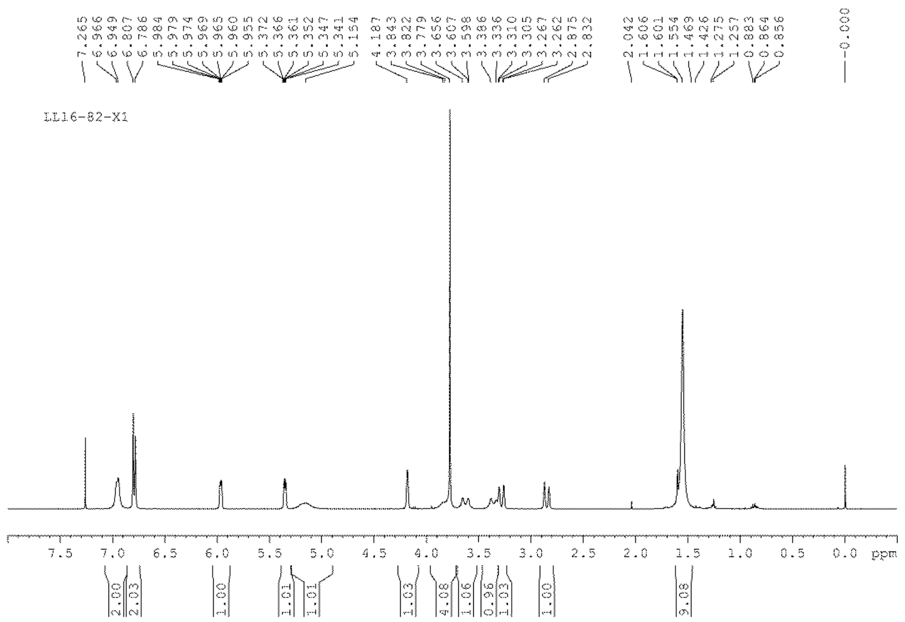


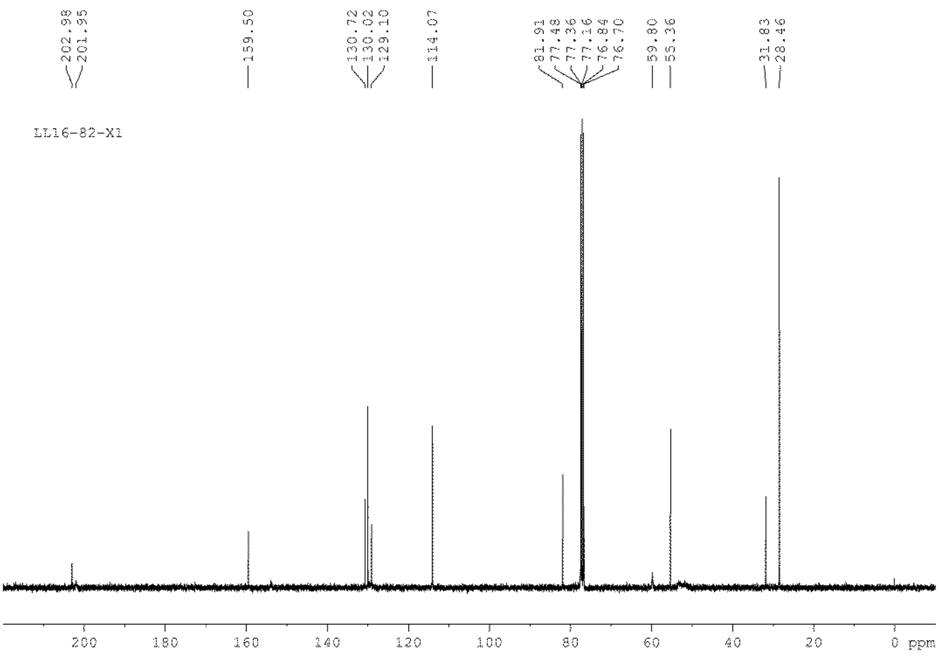


**Supplementary Figure 112**. $^{1}H$ and $^{13}C$ NMR spectra of product **3af**

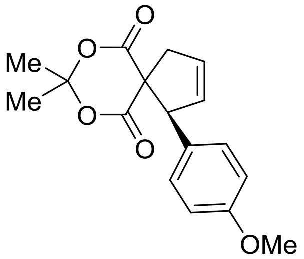


**3ag**

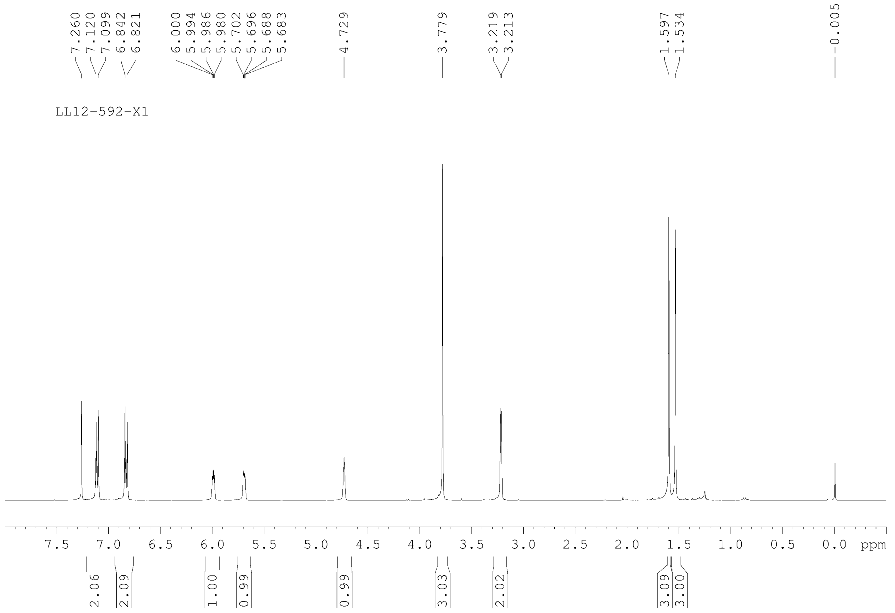


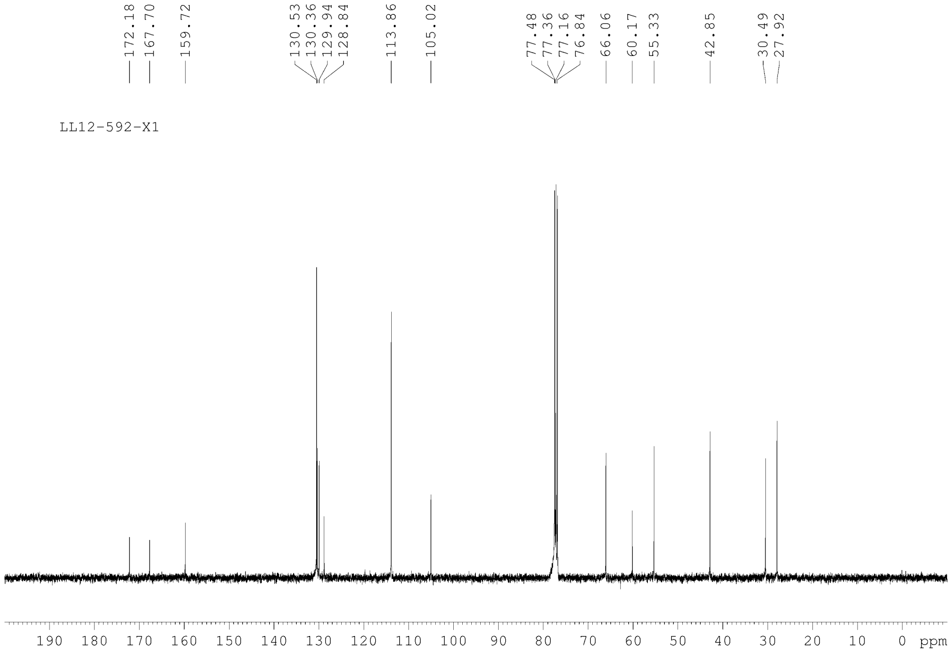


**Supplementary Figure 113**. $^{1}$H and $^{13}$C NMR spectra of product **3ag**

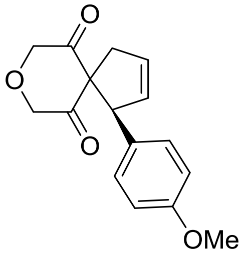


**3ah**

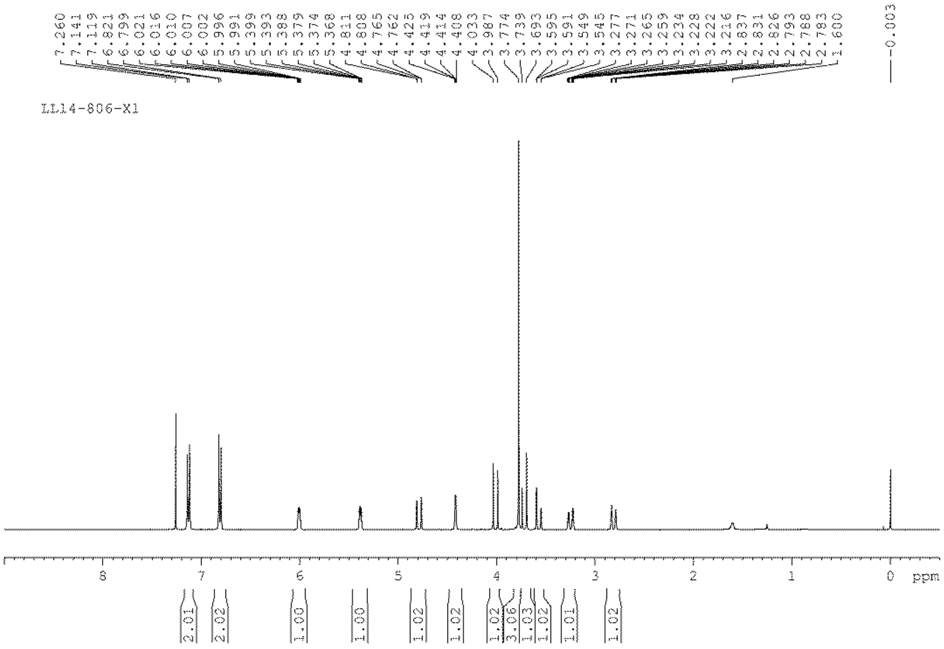


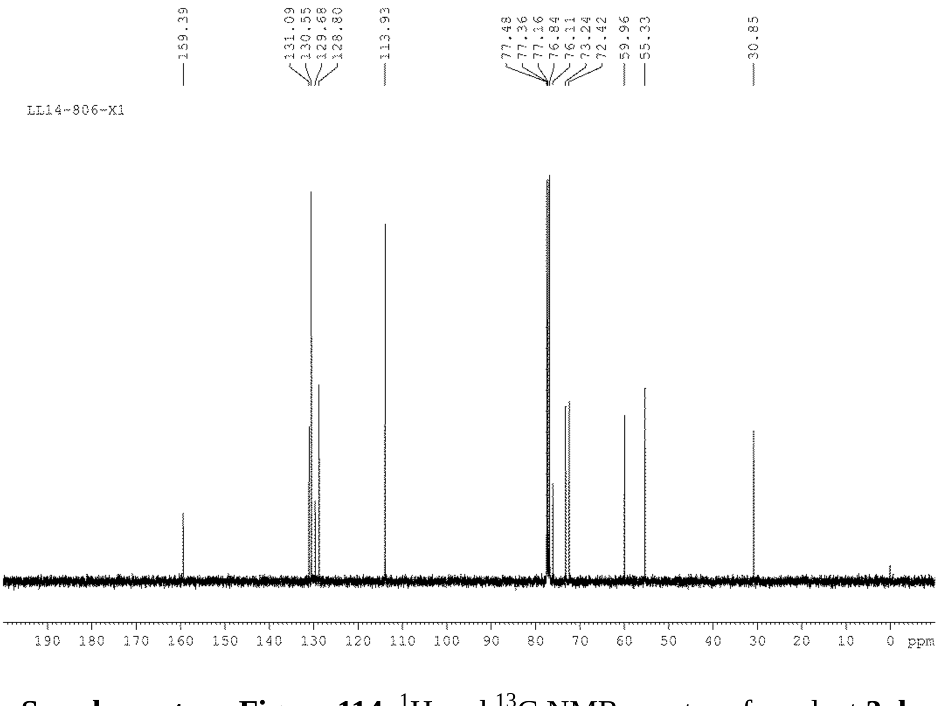


**Supplementary Figure 114**. $^{1}H$ and $^{13}C$ NMR spectra of product **3ah**

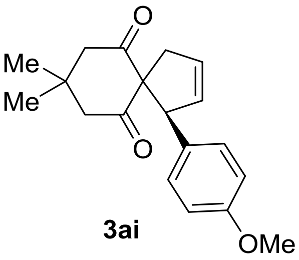

**3ai**

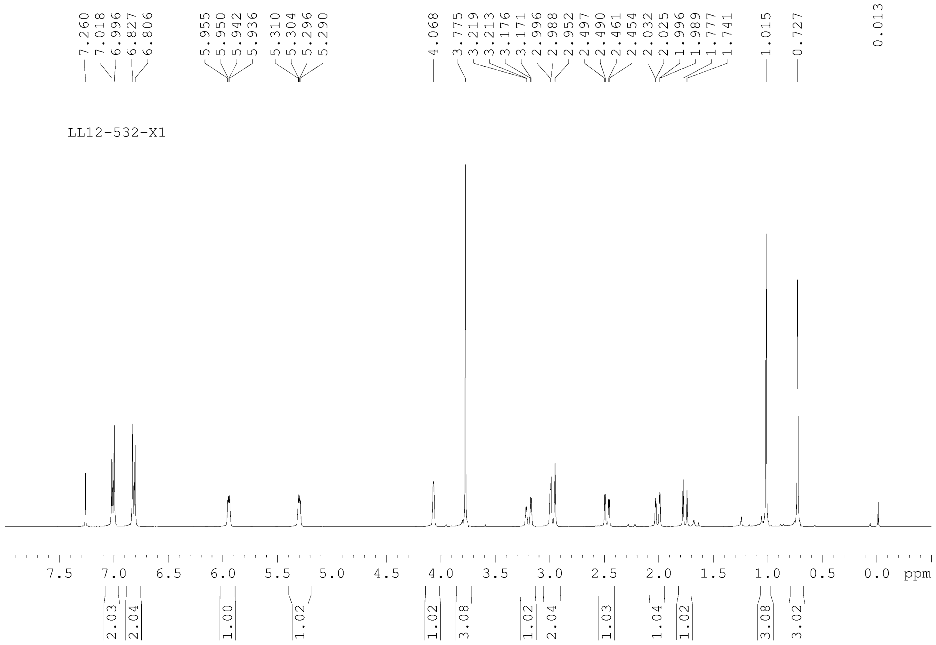


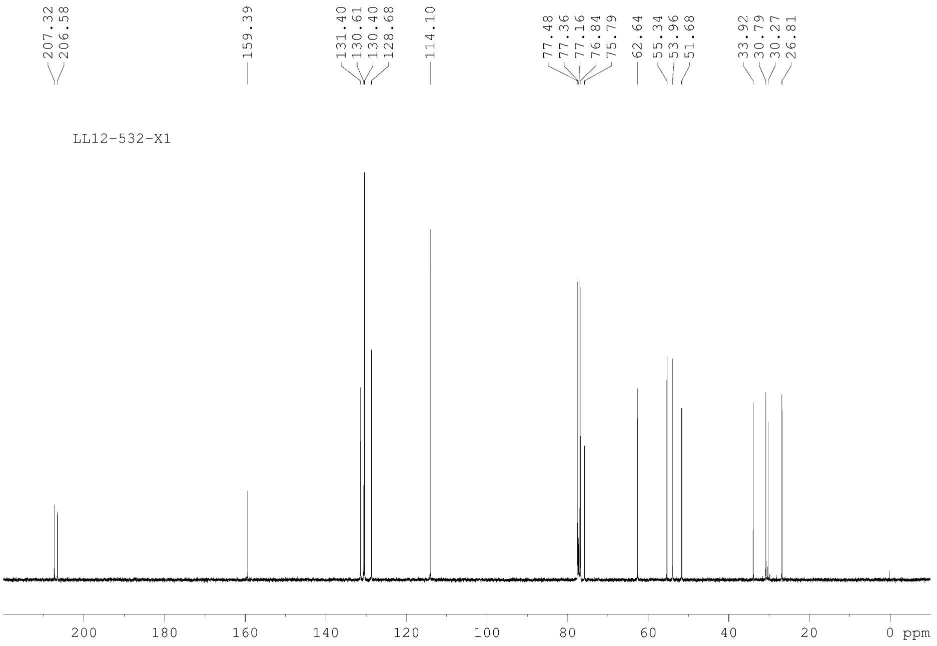


**Supplementary Figure 115**. $^{1}$H and $^{13}$C NMR spectra of product **3ai**

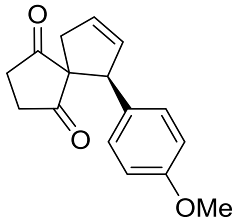

**3aj**

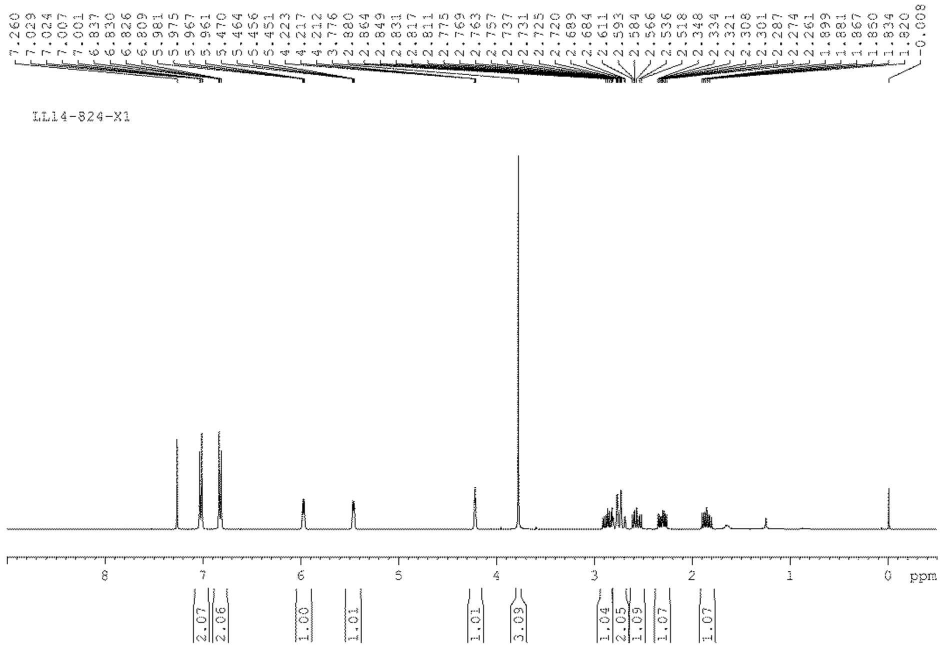


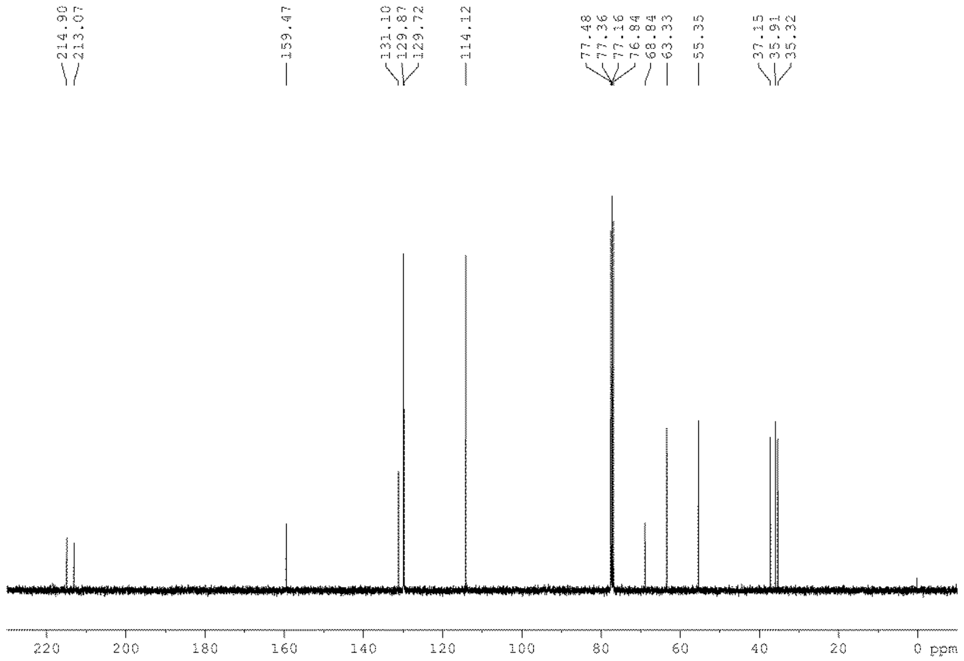


**Supplementary Figure 116**. $^{1}$H and $^{13}$C NMR spectra of product **3aj**

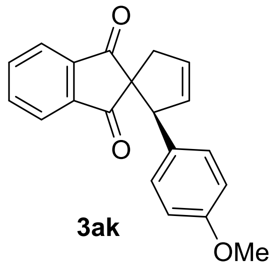

3ak

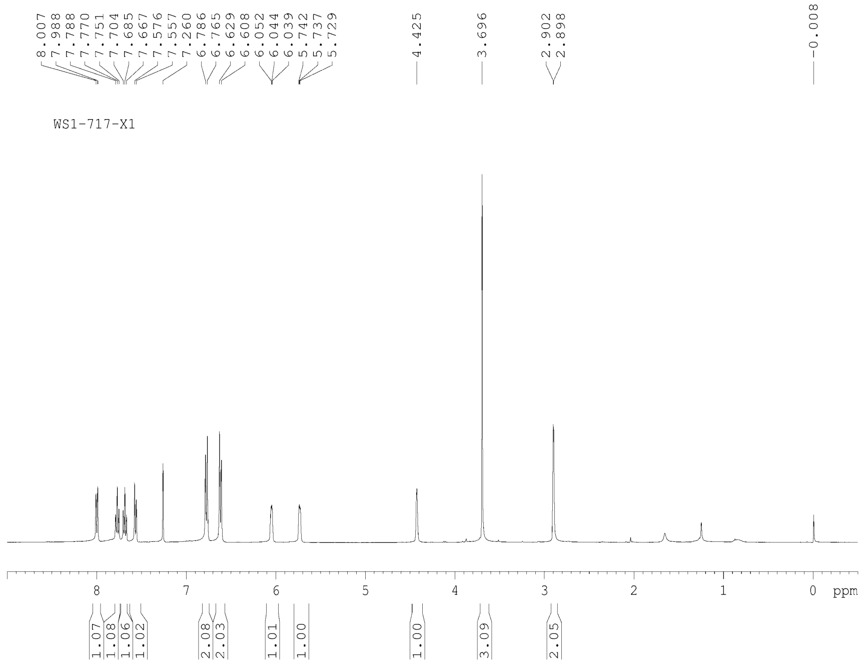

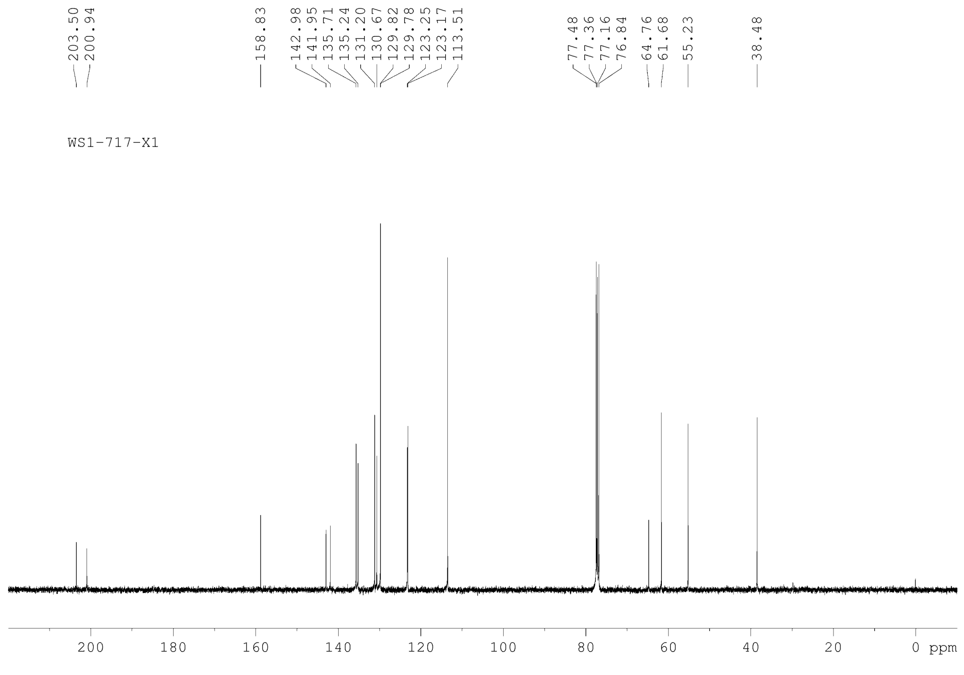

**Supplementary Figure 117**. $^{1}H$ and $^{13}C$ NMR spectra of product **3ak**

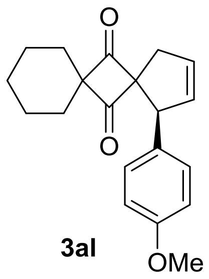


**3al**

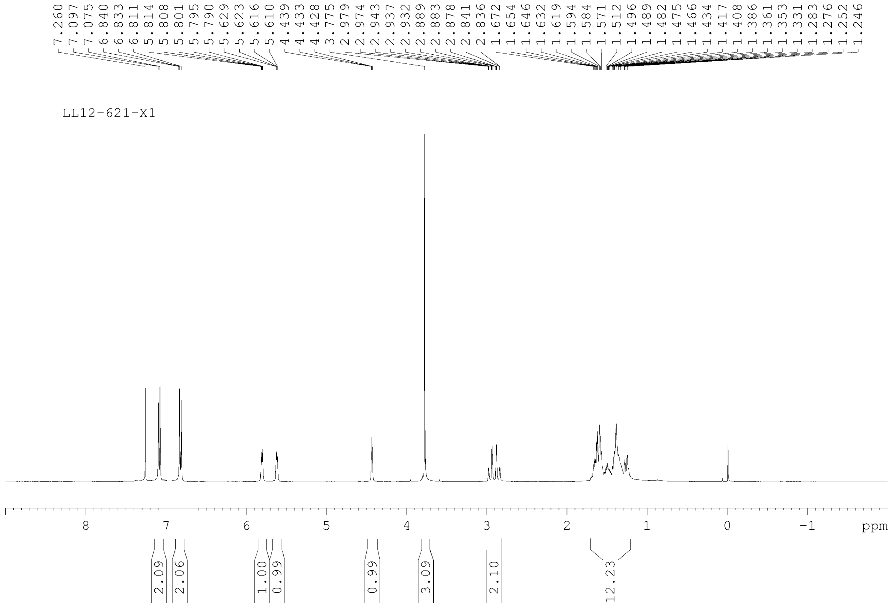


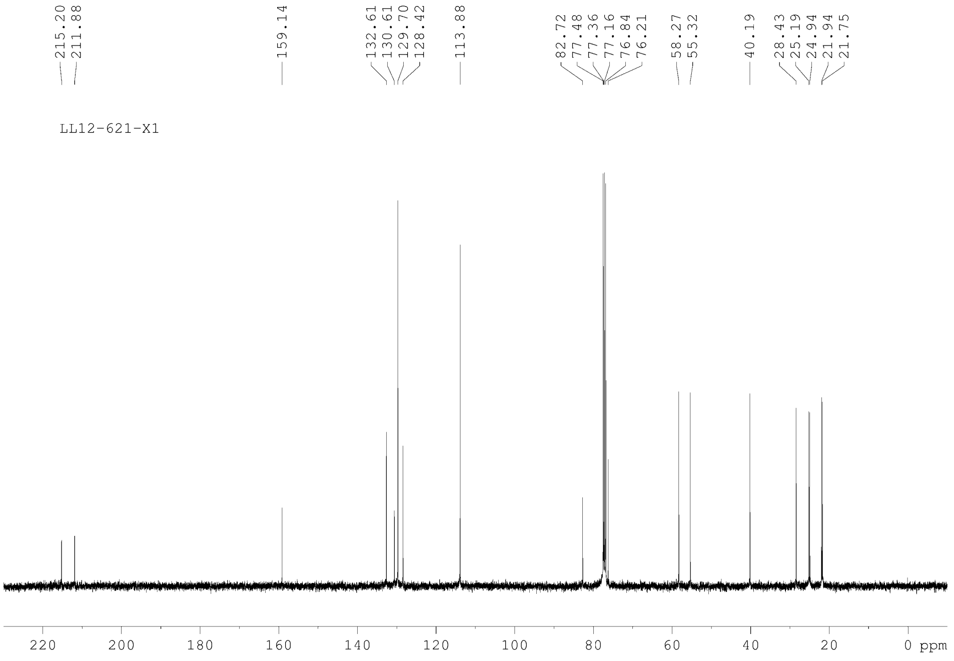


**Supplementary Figure 118**. $^{1}$H and $^{13}$C NMR spectra of product **3al**

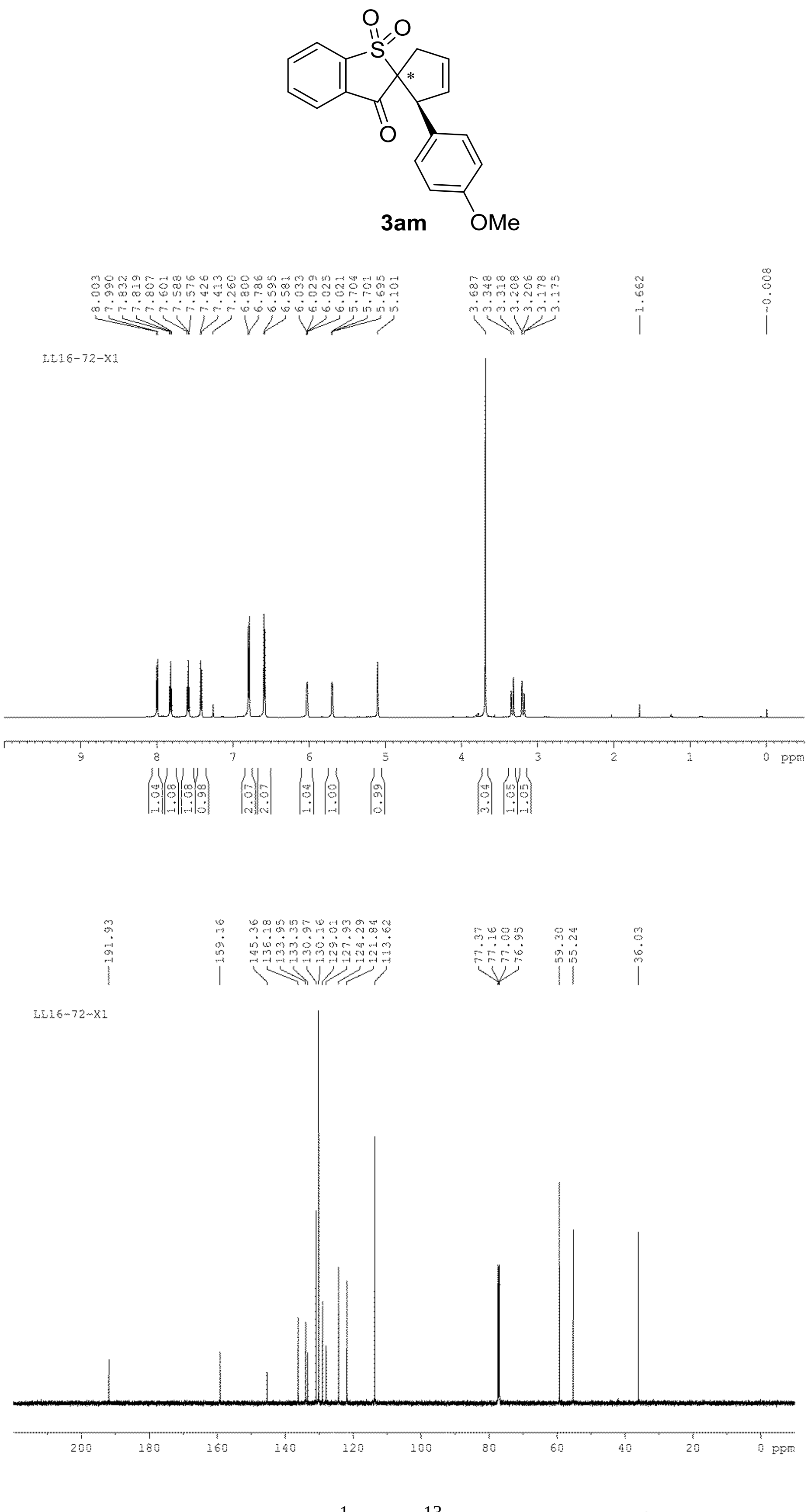


**Supplementary Figure 119**. $^{1}$H and $^{13}$C NMR spectra of product **3am**

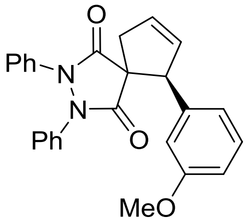


**3ba**

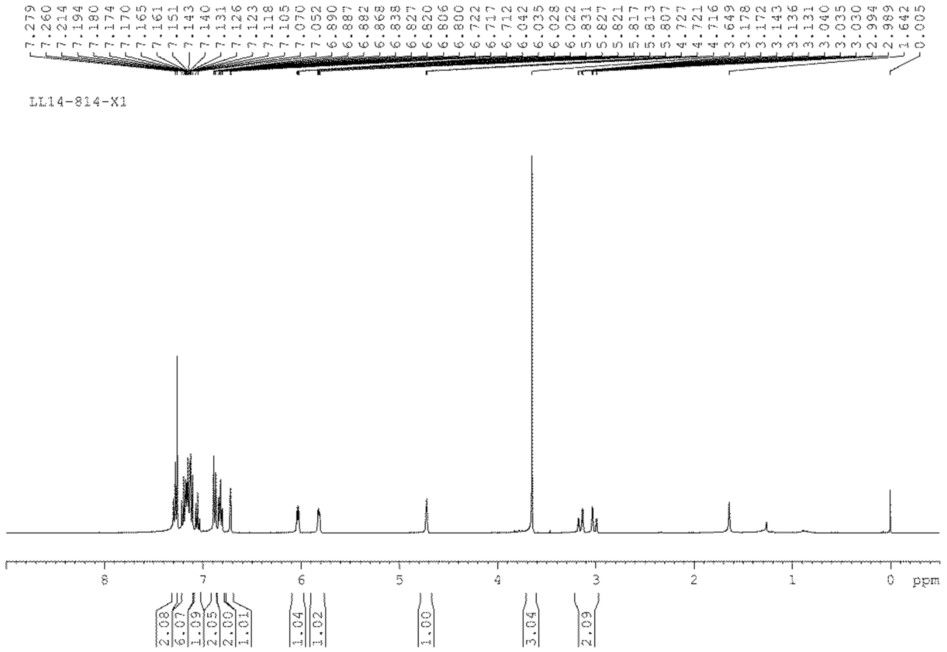


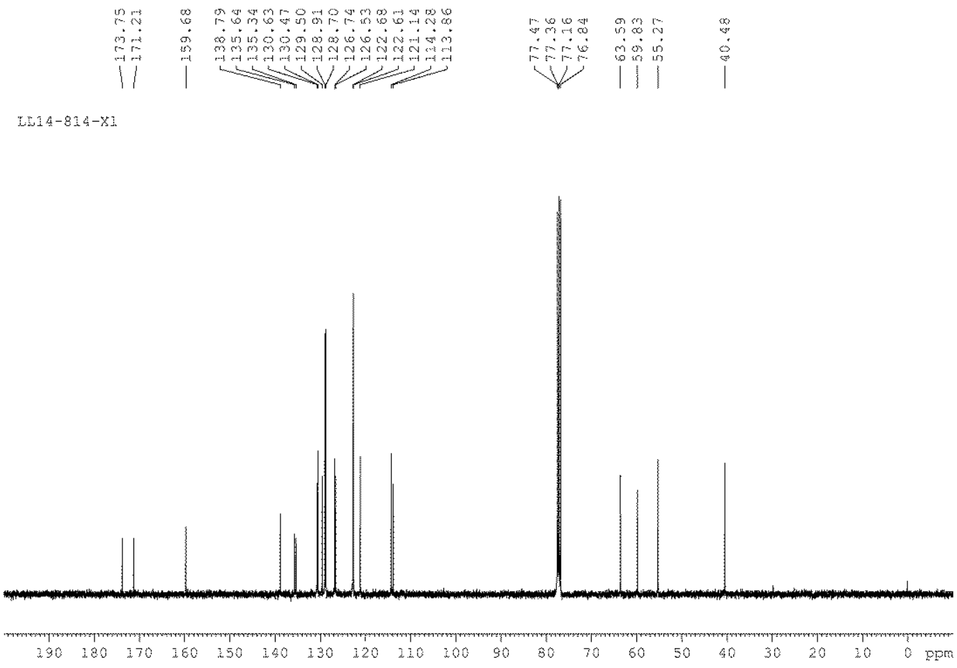


**Supplementary Figure 120**. $^{1}$H and $^{13}$C NMR spectra of product **3ba**

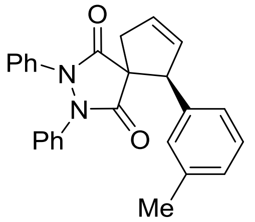


**3ca**

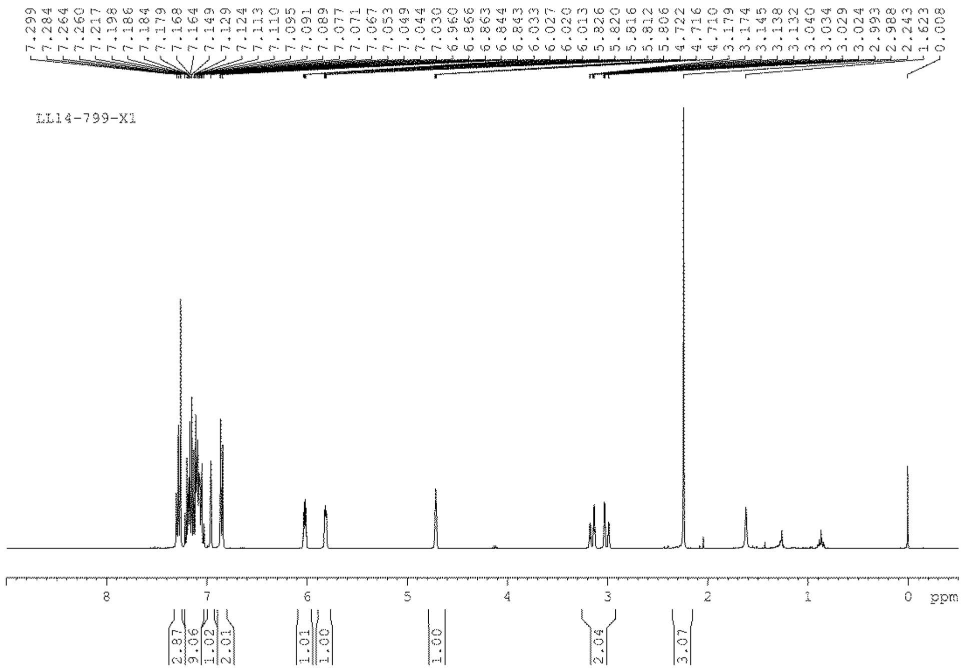


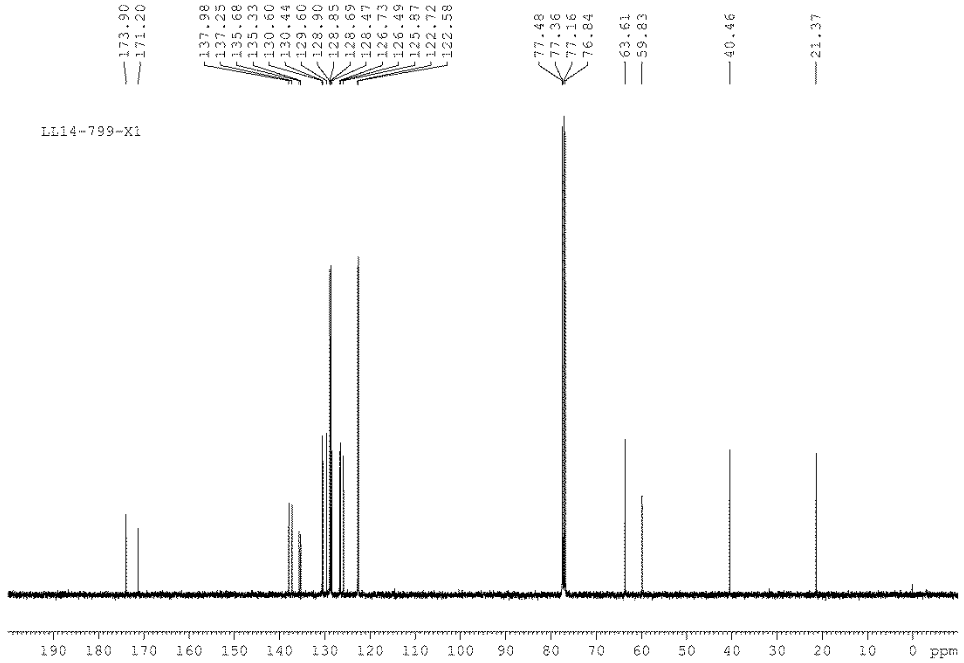


**Supplementary Figure 121**. $^{1}$H and $^{13}$C NMR spectra of product **3ca**

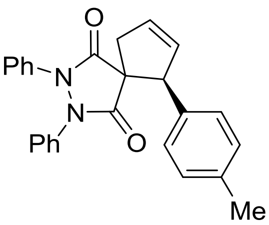


**3da**

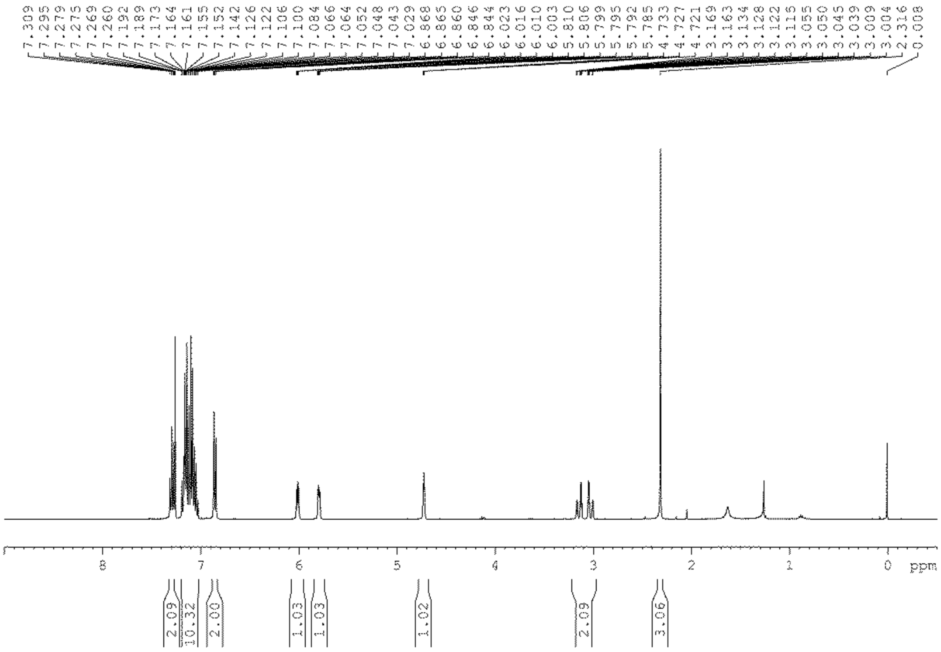


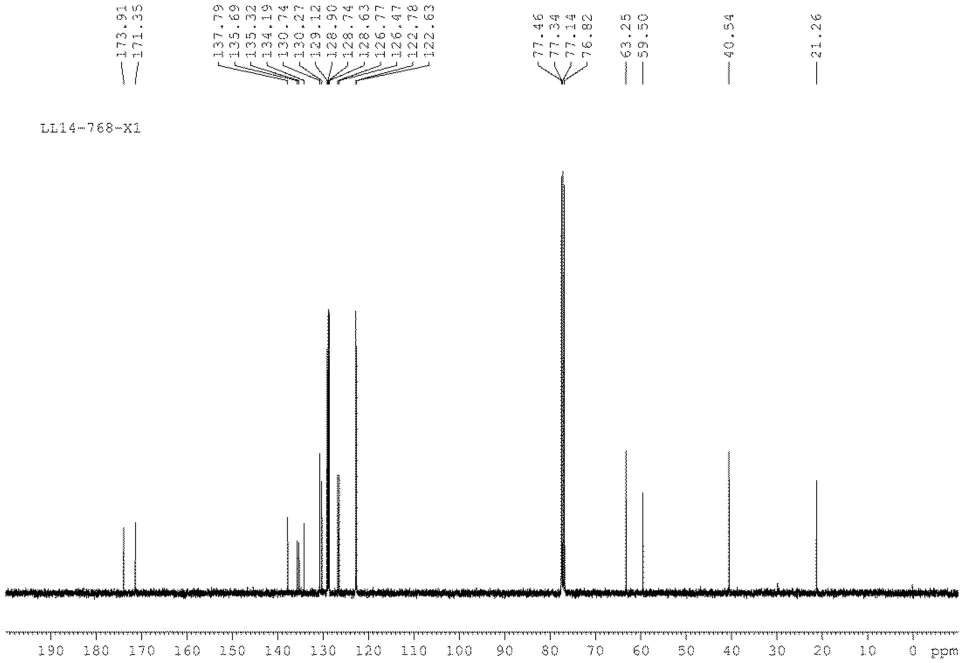


**Supplementary Figure 122**. $^{1}H$ and $^{13}C$ NMR spectra of product **3da**

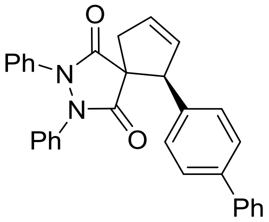

**3ea**

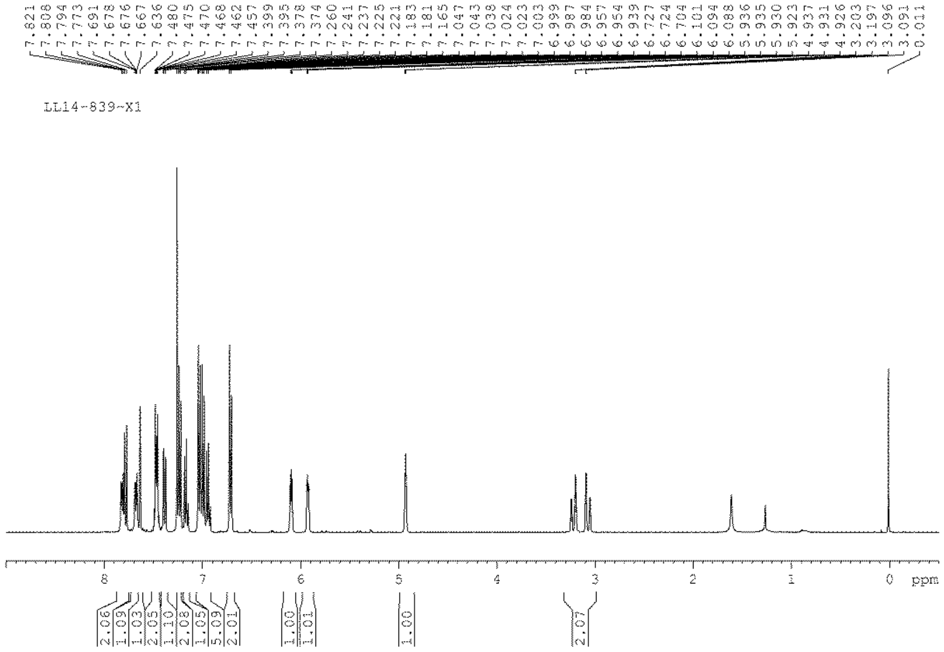

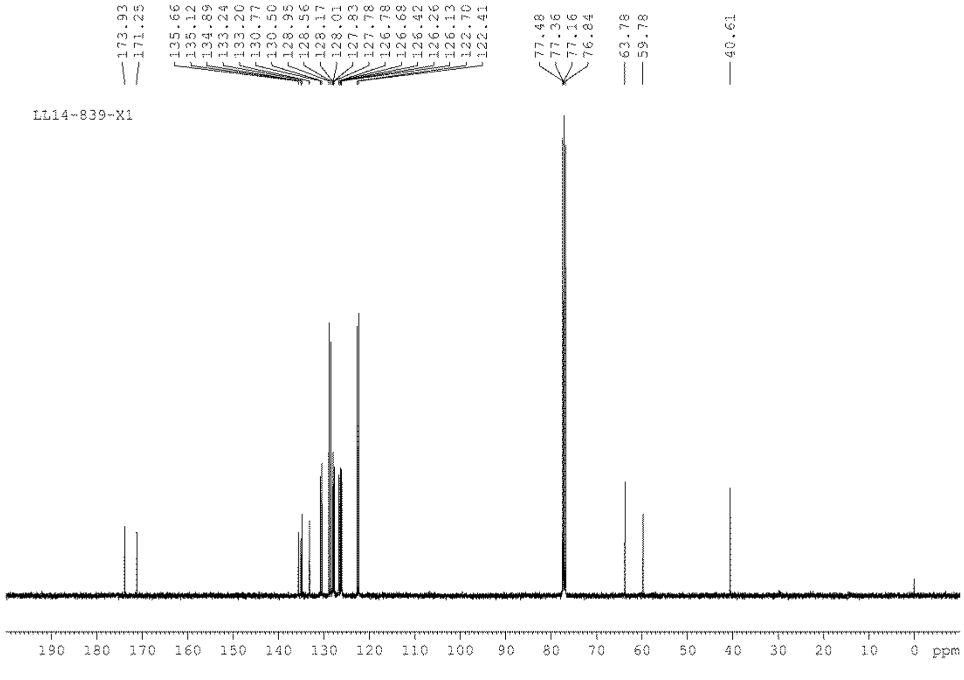

**Supplementary Figure 123**. $^{1}$H and $^{13}$C NMR spectra of product **3ea**

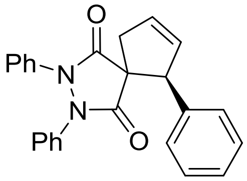


**3fa**

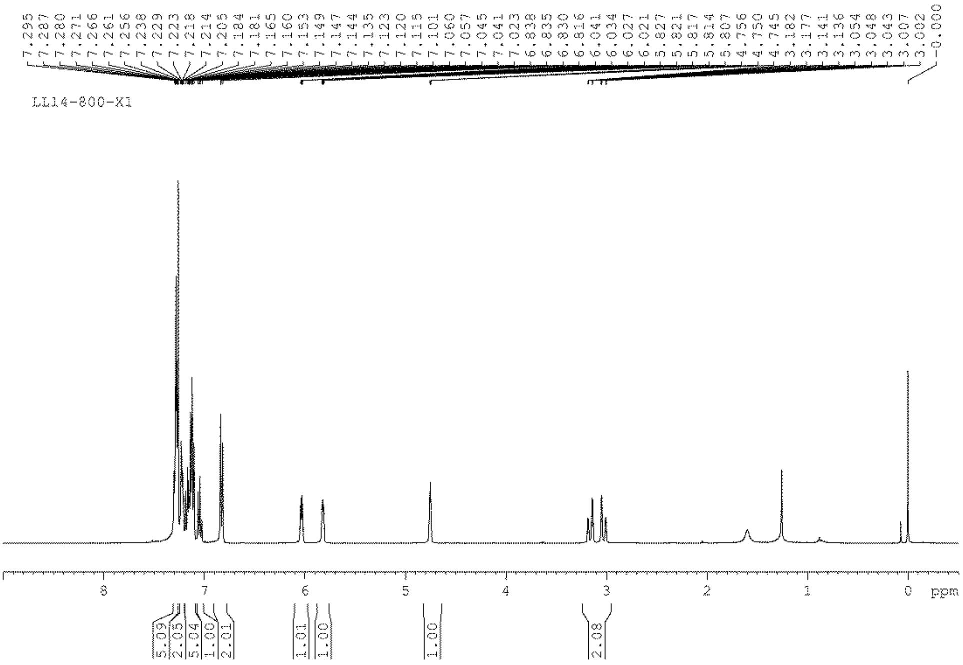


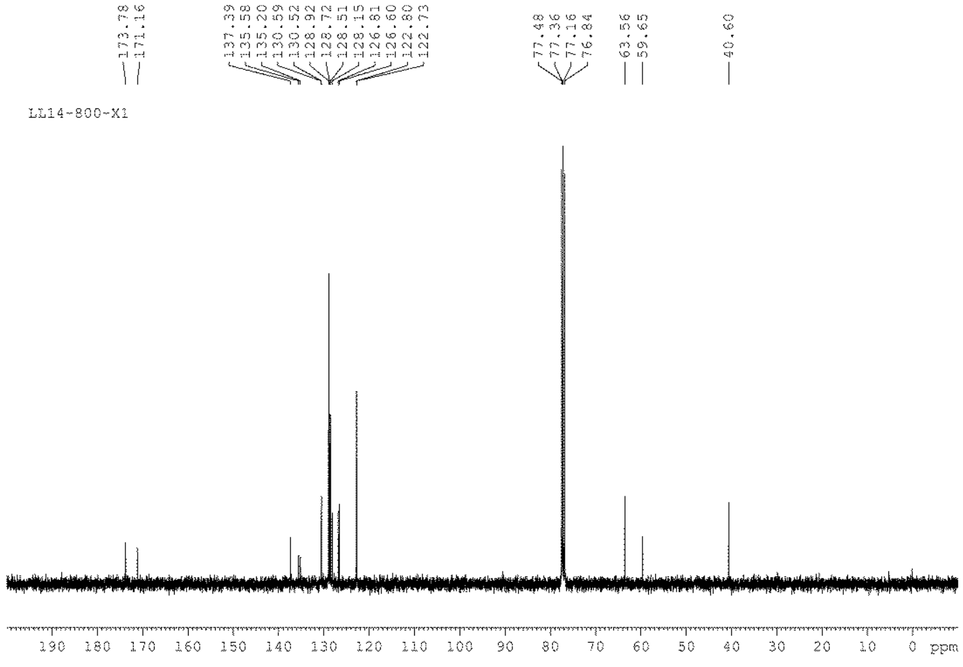


**Supplementary Figure 124**. $^{1}$H and $^{13}$C NMR spectra of product **3fa**

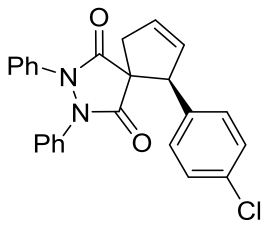


**3ga**

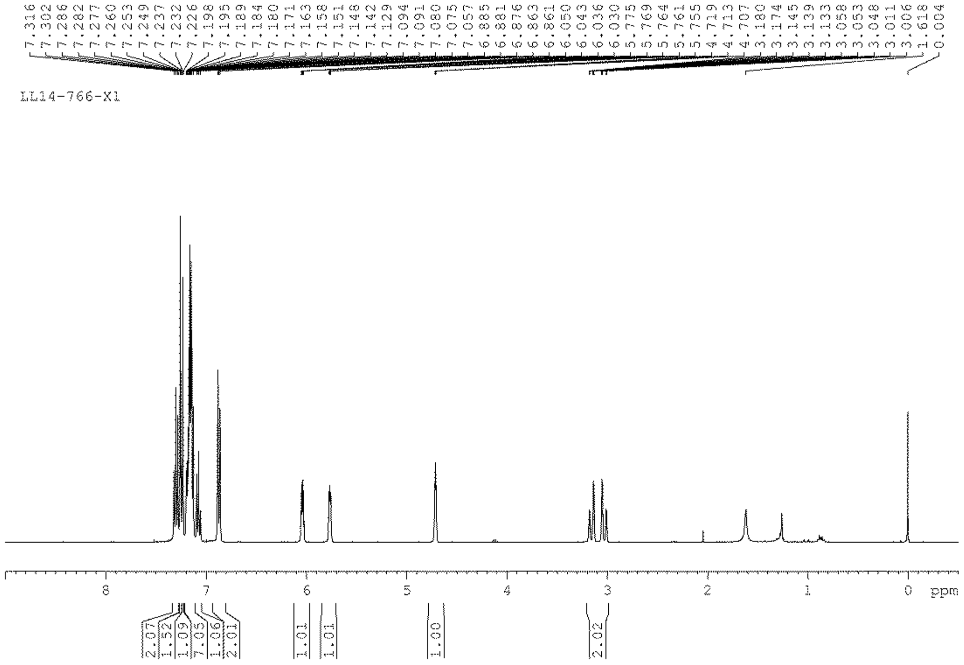


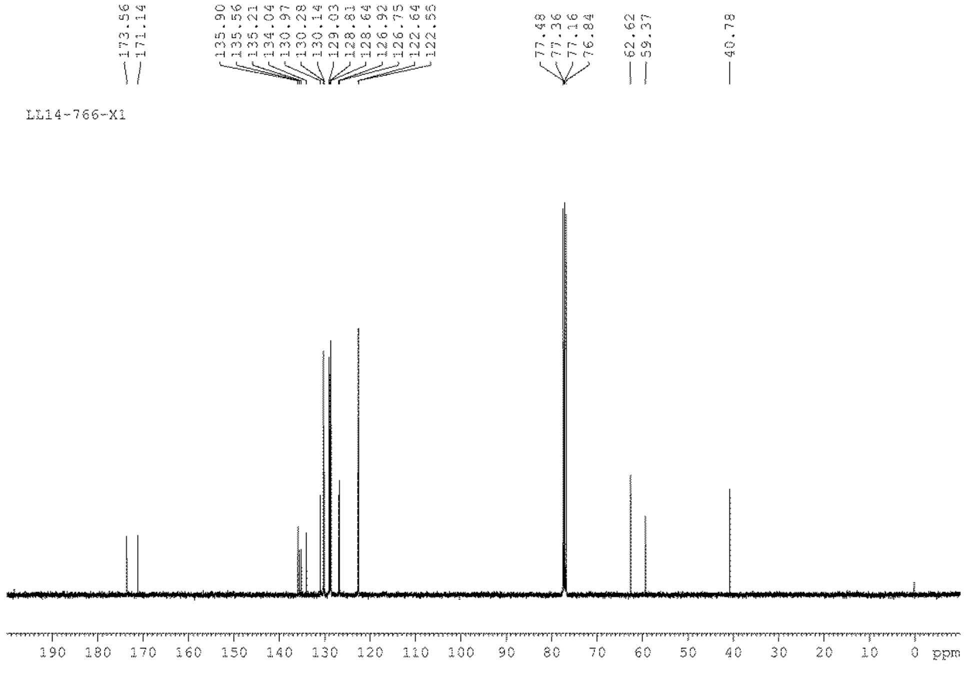


**Supplementary Figure 125**. $^{1}H$ and $^{13}C$ NMR spectra of product **3ga**

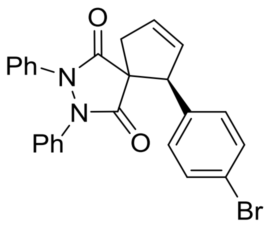


**3ha**

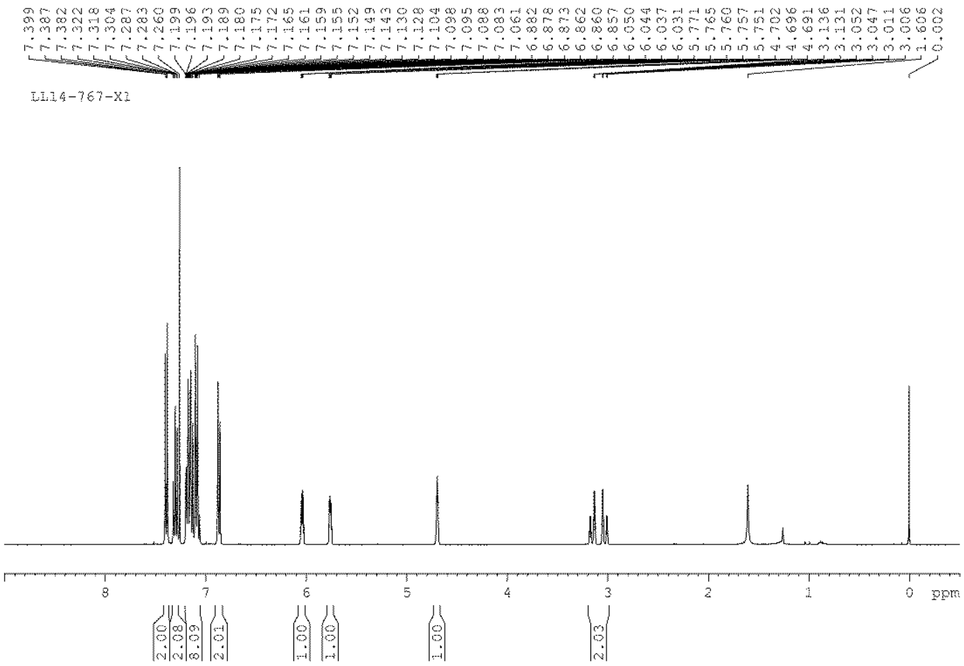


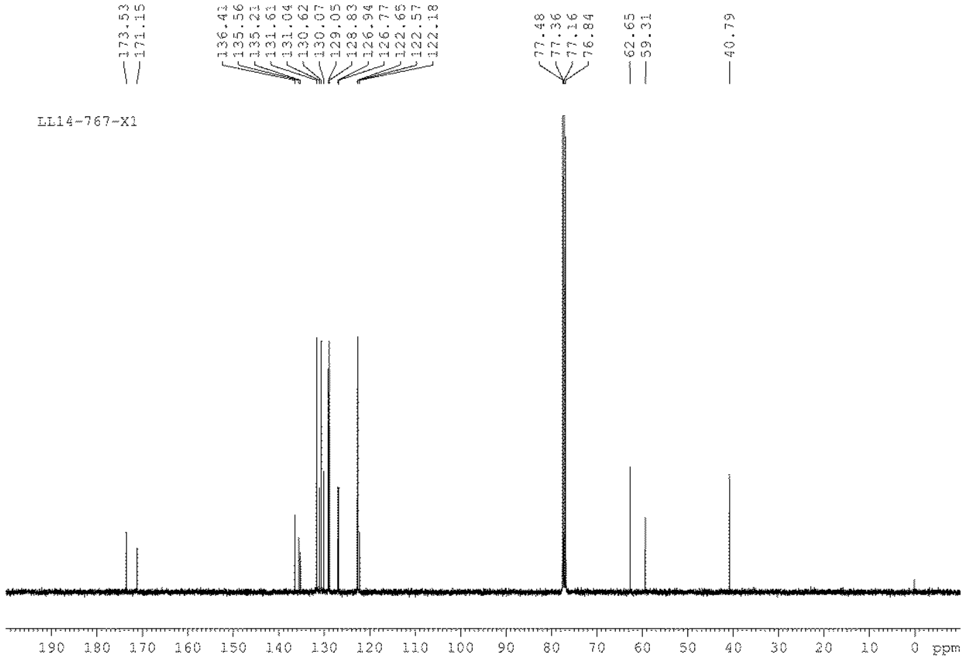


**Supplementary Figure 126**. $^{1}H$ and $^{13}C$ NMR spectra of product **3ha**

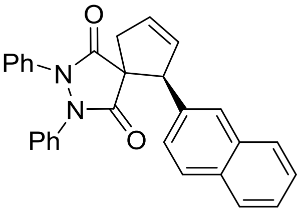

**3ia**

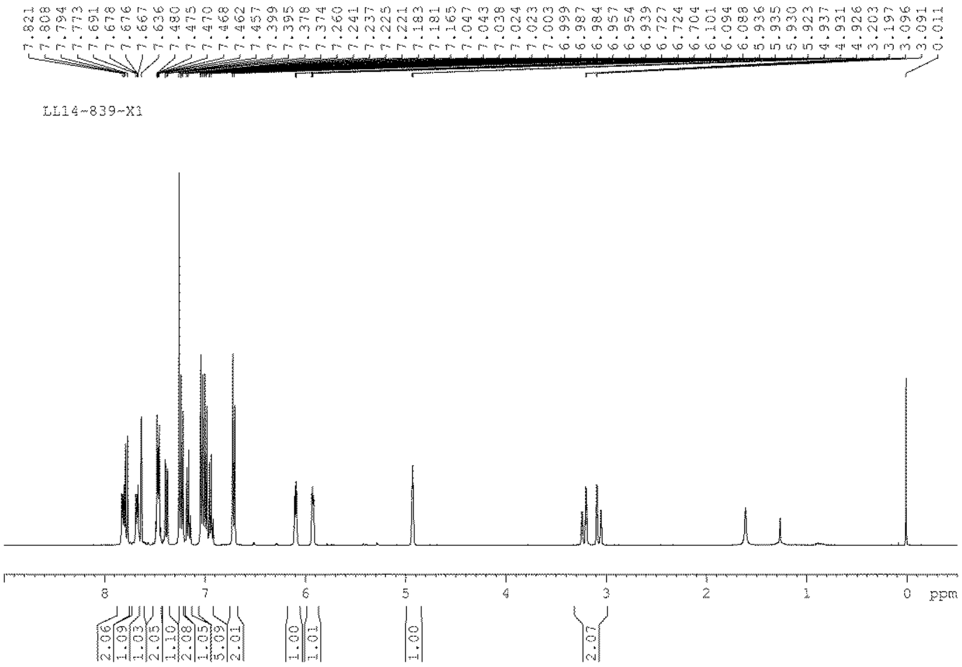

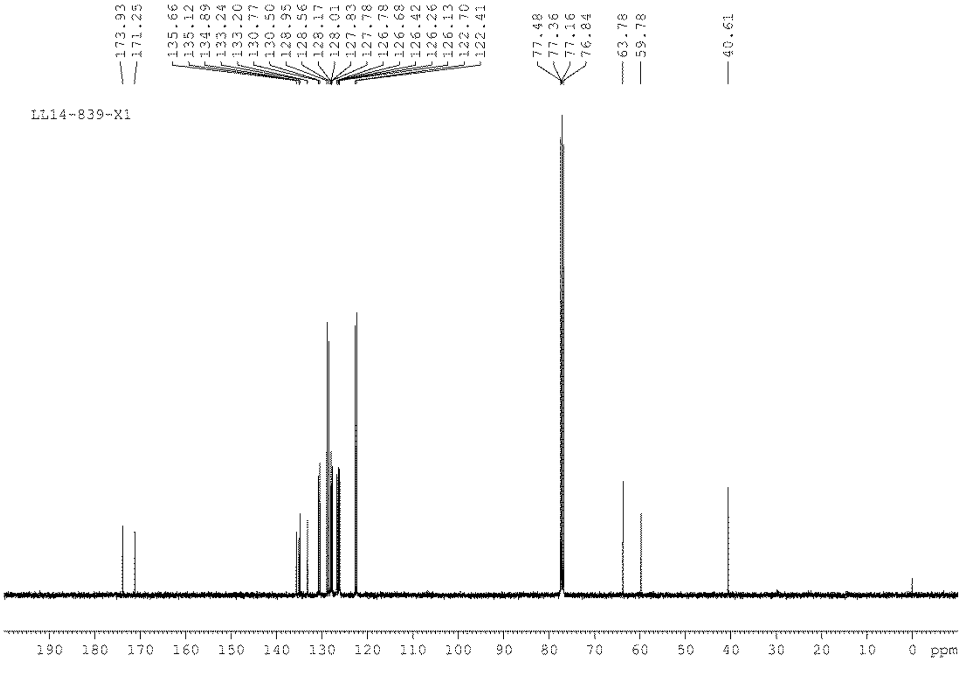

**Supplementary Figure 127**. $^{1}H$ and $^{13}C$ NMR spectra of product **3ia**

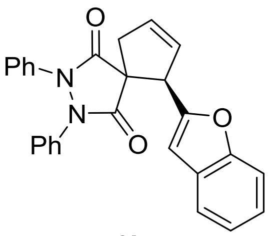


**3ja**

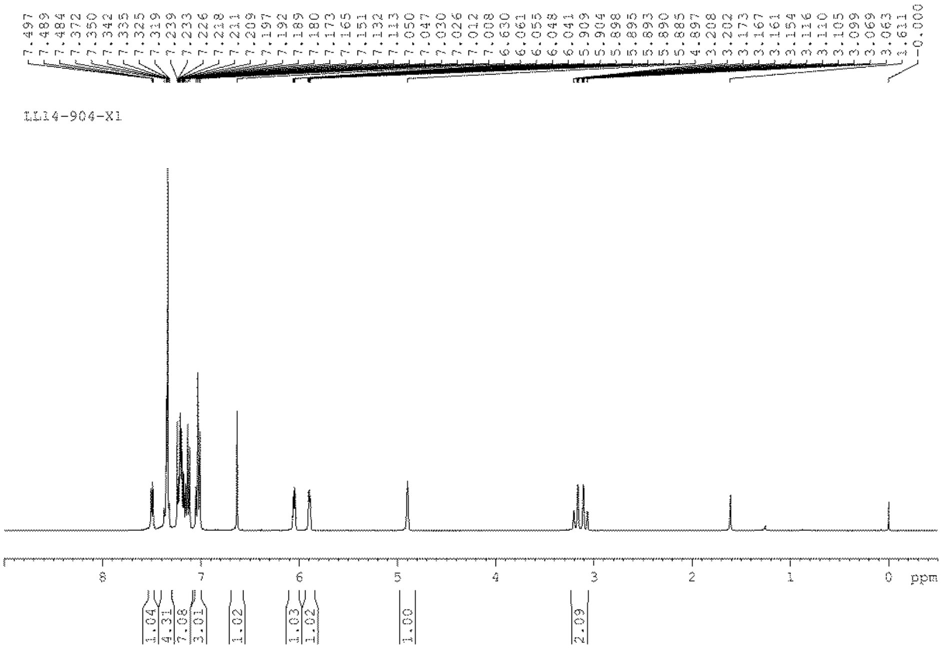


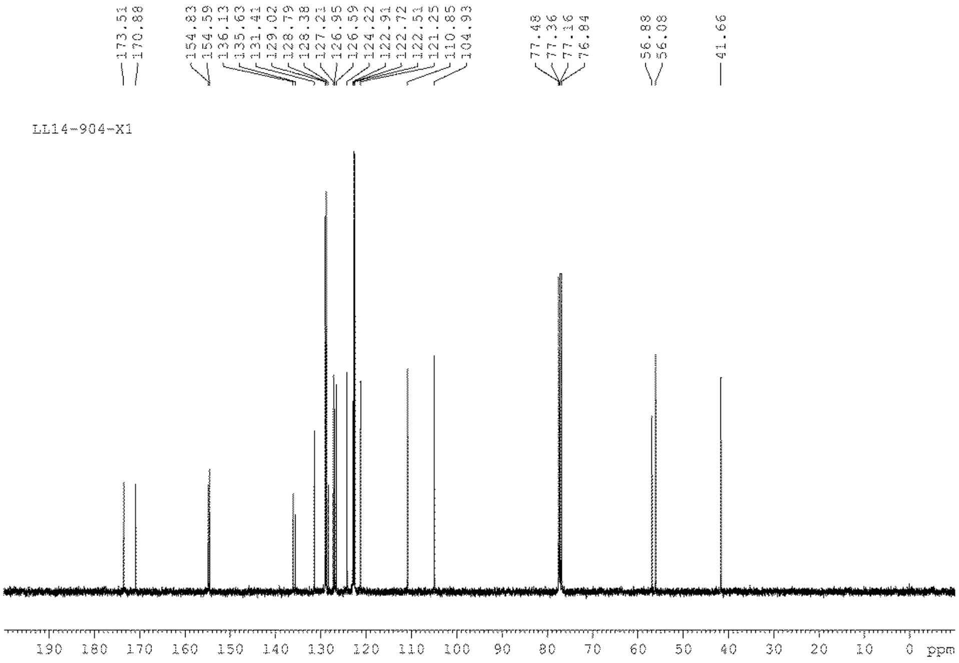


**Supplementary Figure 128**. $^{1}$H and $^{13}$C NMR spectra of product **3ja**

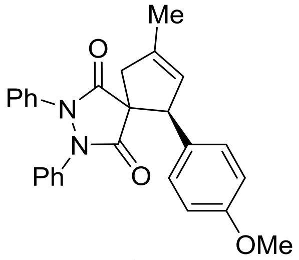


**3ka**

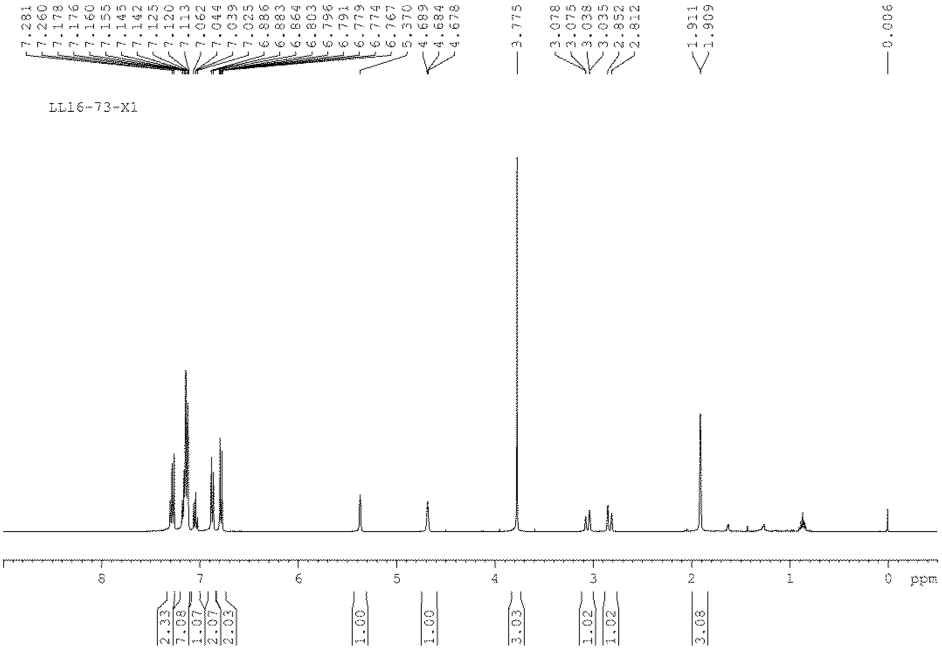


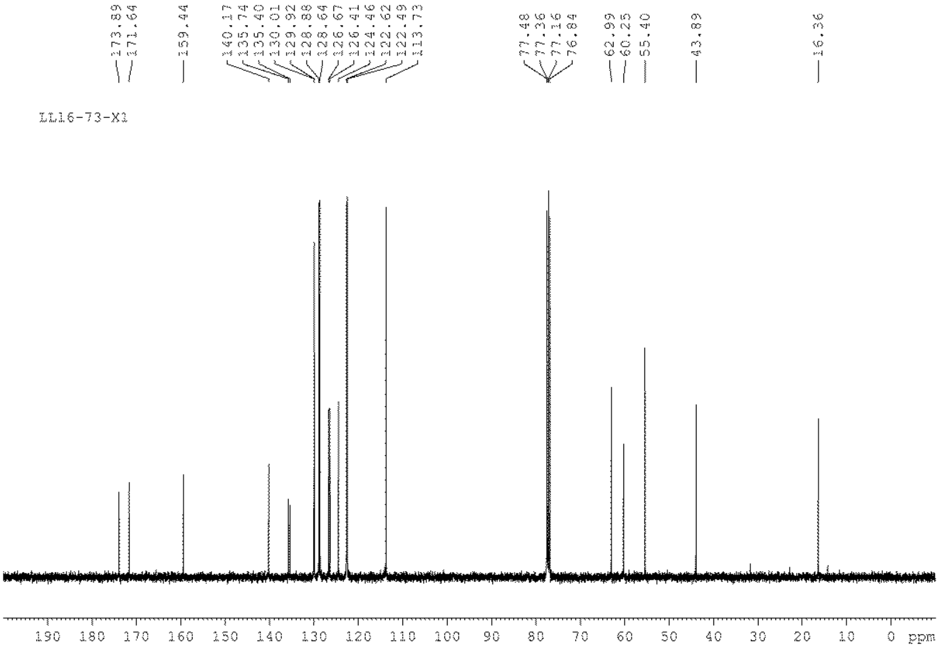


**Supplementary Figure 129**. $^{1}H$ and $^{13}C$ NMR spectra of product **3ka**

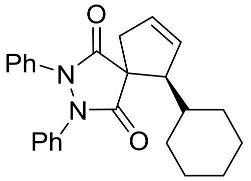


**3la**

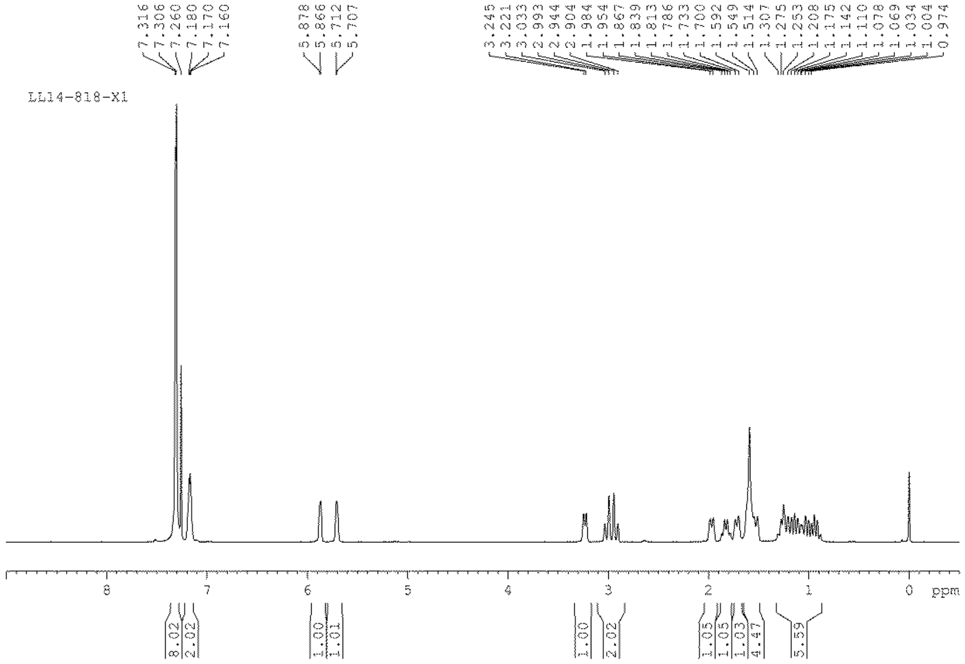


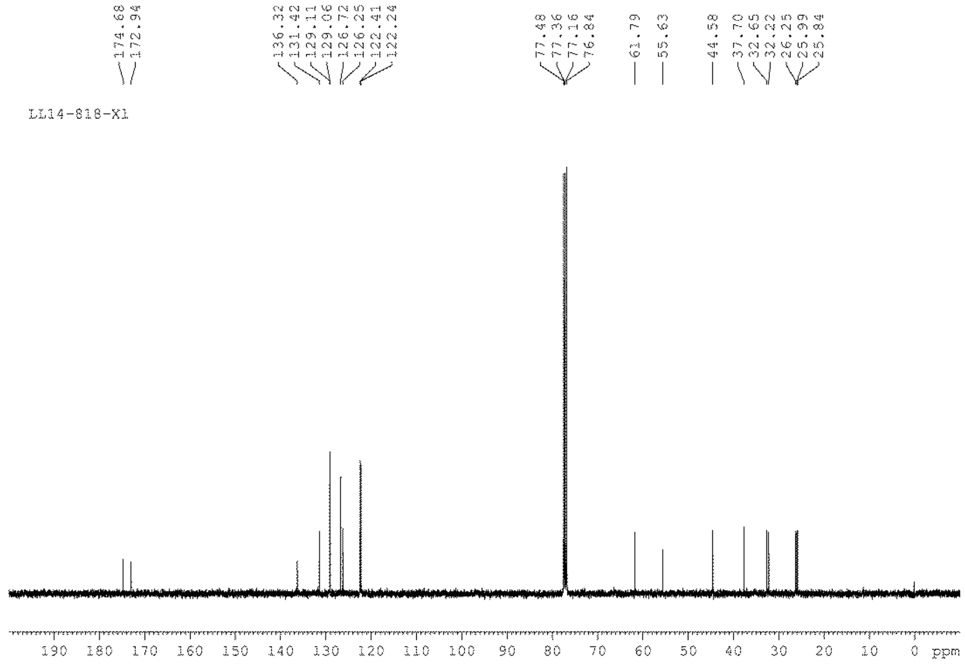


**Supplementary Figure 130**. $^{1}$H and $^{13}$C NMR spectra of product **3la**

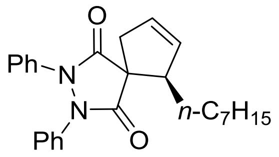

**3ma**

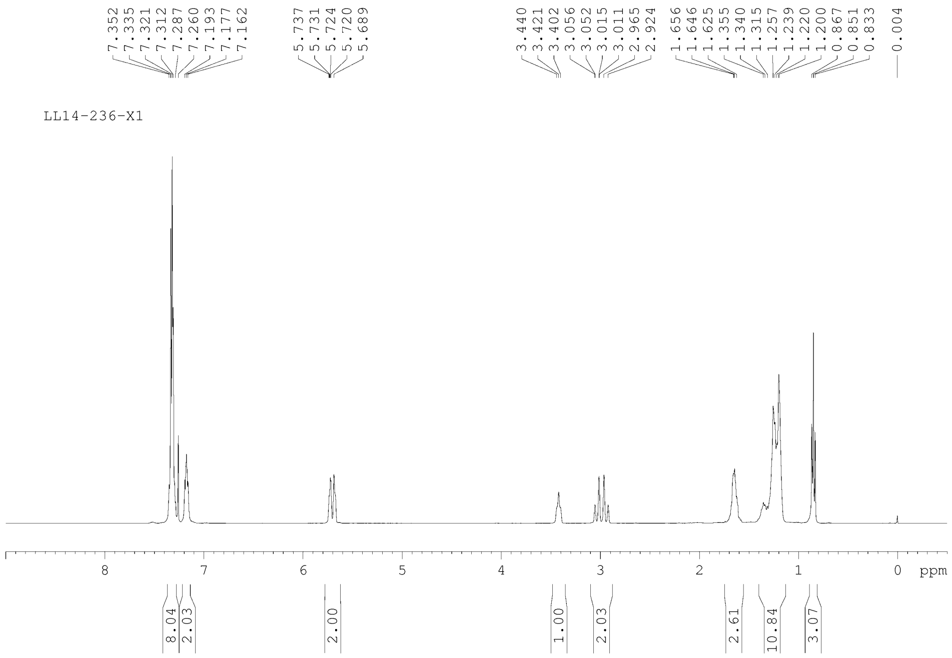


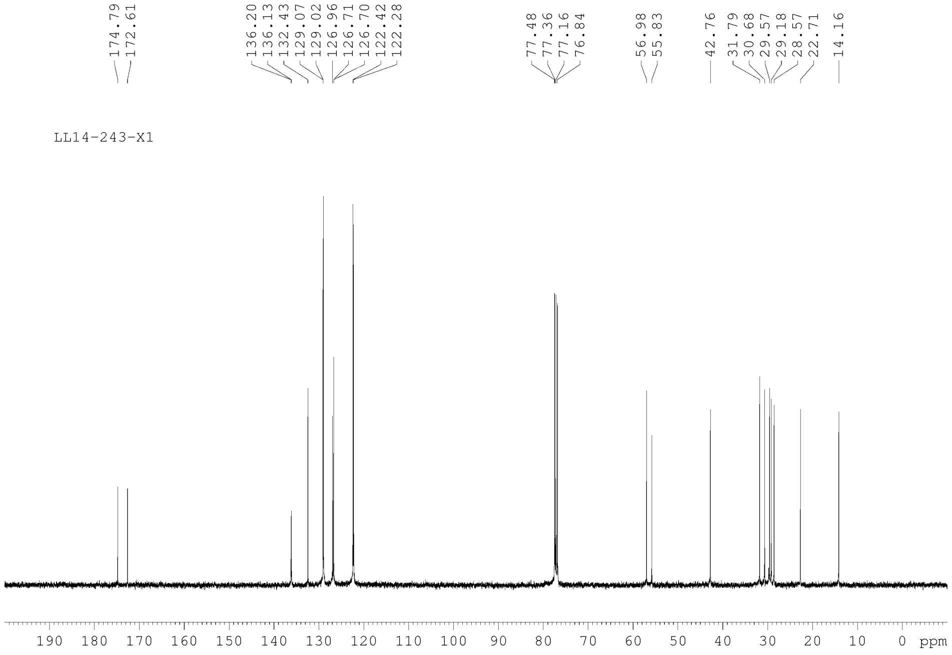


**Supplementary Figure 131**. $^{1}$H and $^{13}$C NMR spectra of product **3ma**

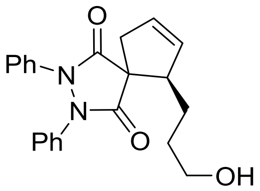


**3na**

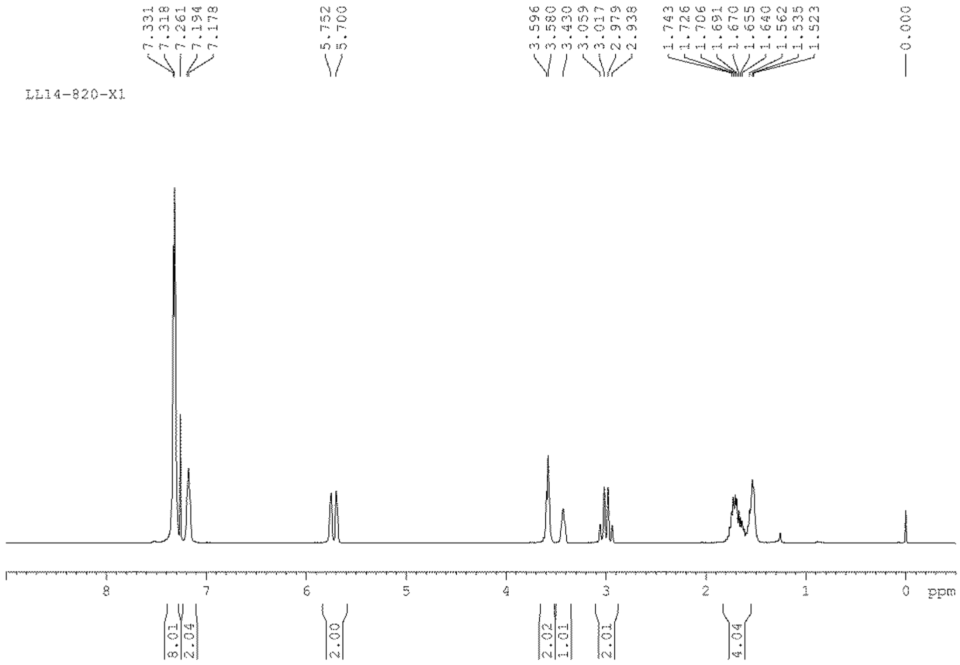


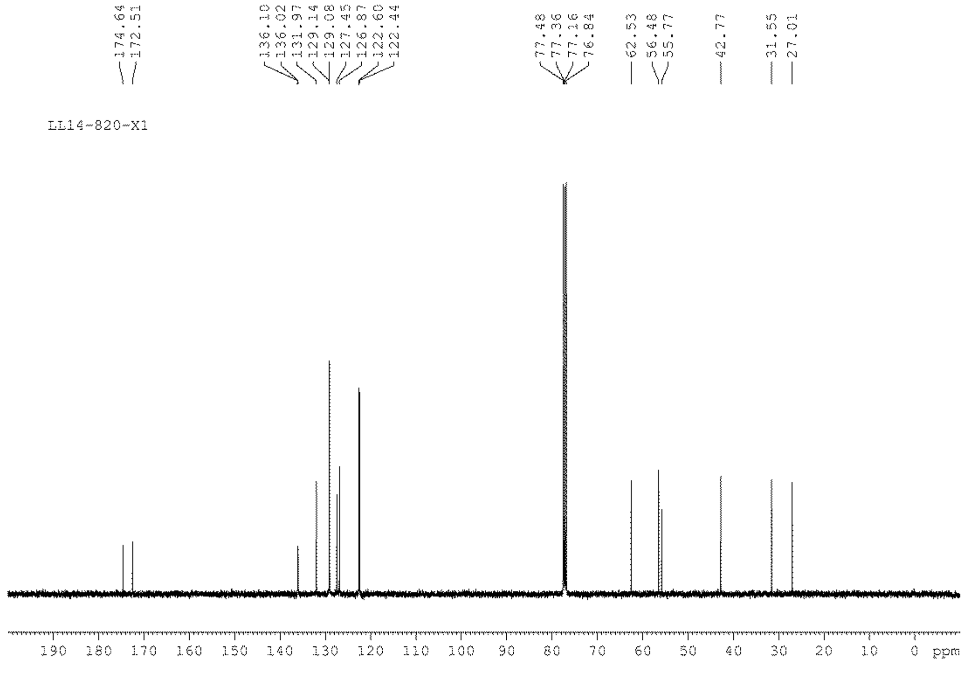


**Supplementary Figure 132**. $^{1}$H and $^{13}$C NMR spectra of product **3na**

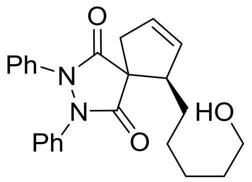


**3oa**

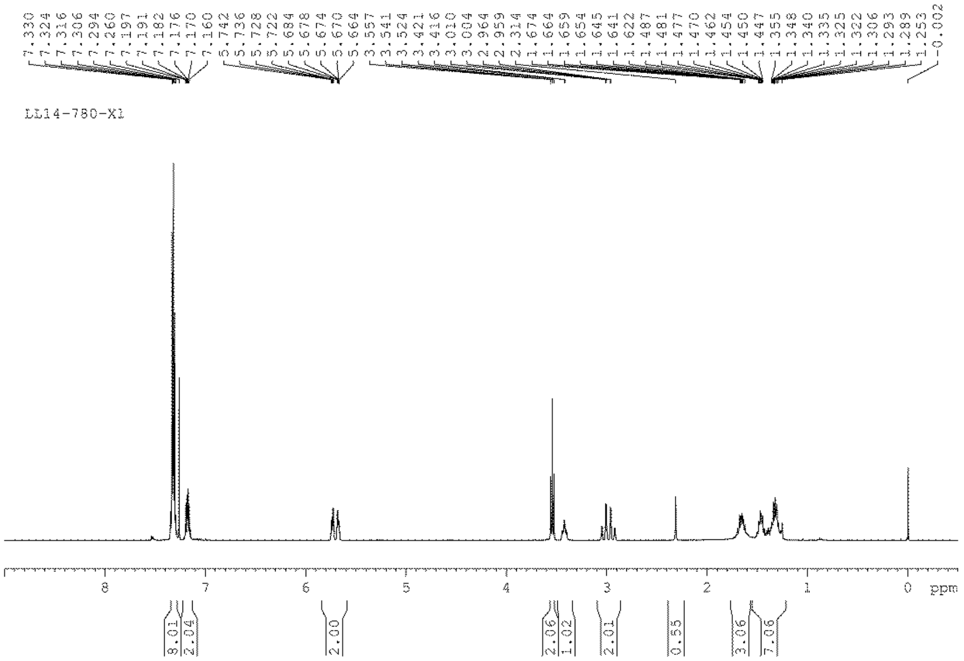


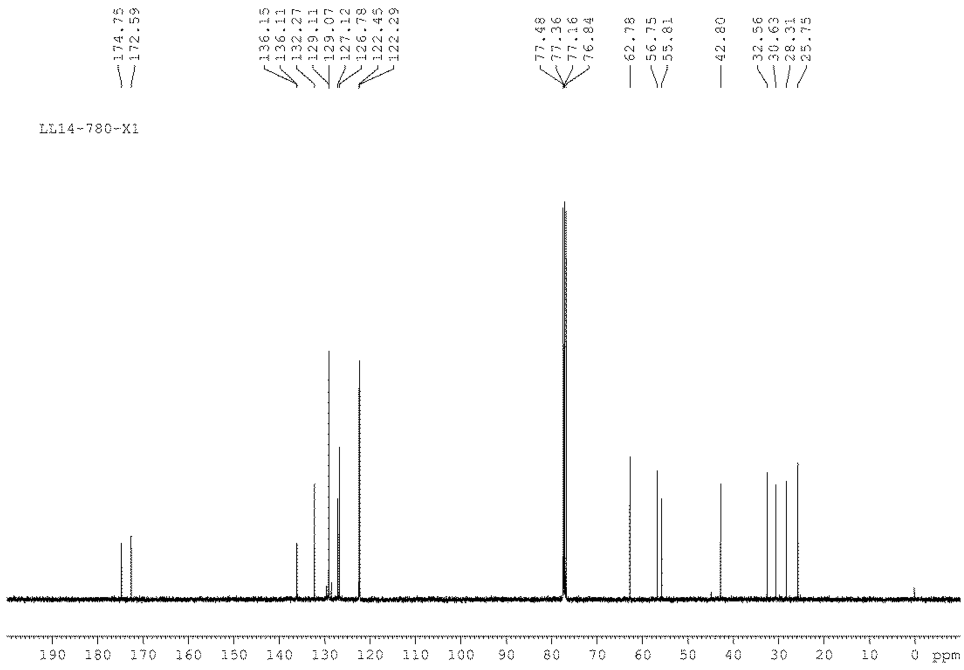


**Supplementary Figure 133**. $^{1}$H and $^{13}$C NMR spectra of product **3oa**

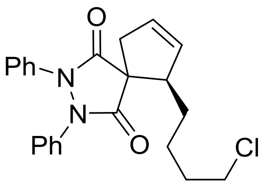


**3pa**

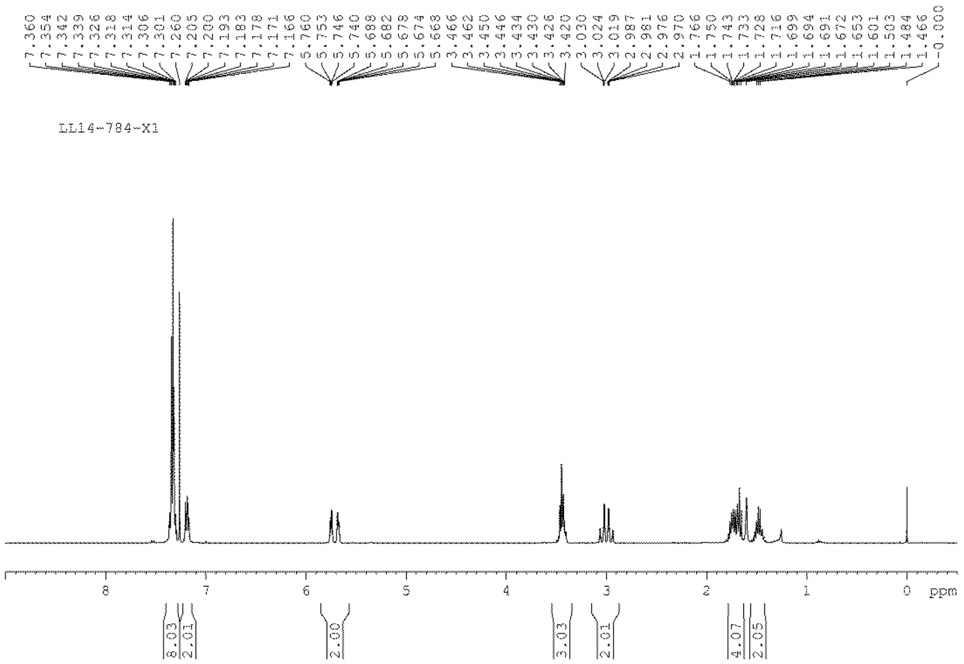


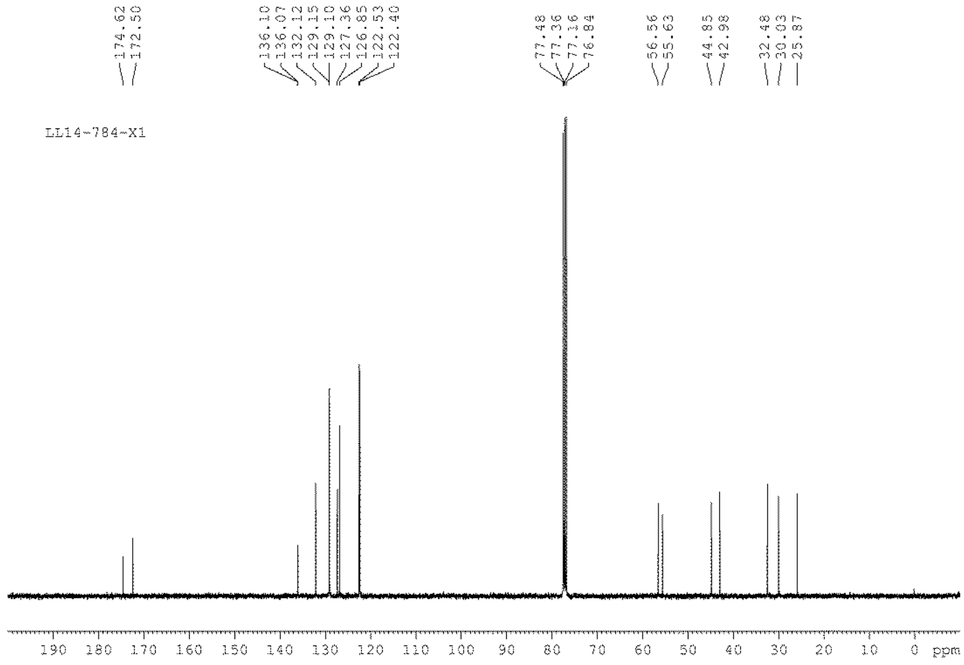


**Supplementary Figure 134**. $^{1}$H and $^{13}$C NMR spectra of product **3pa**

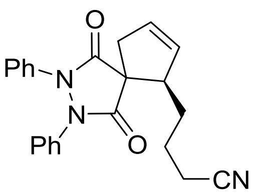


**3qa**

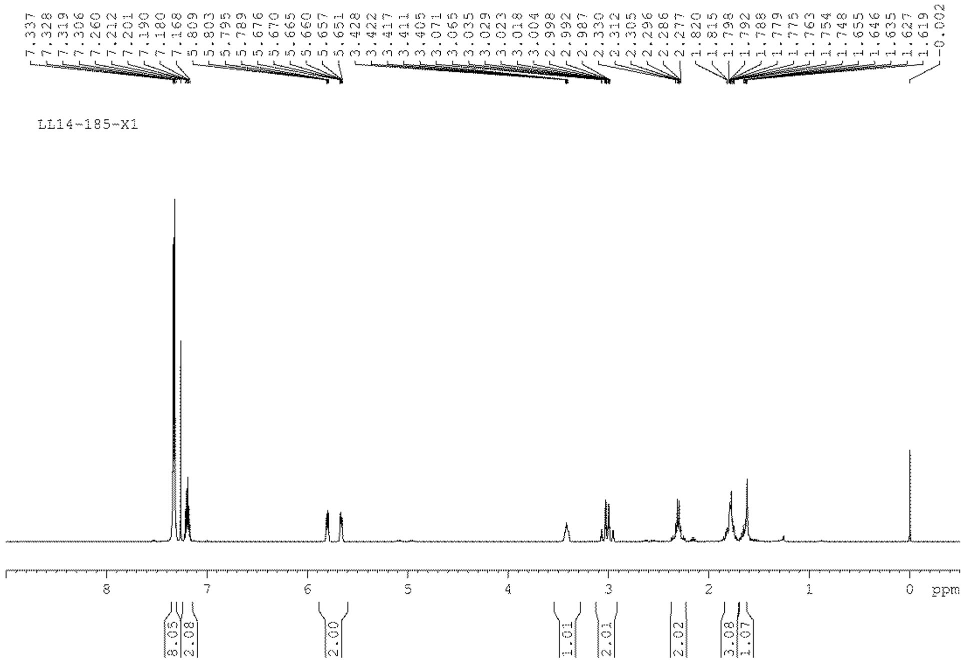


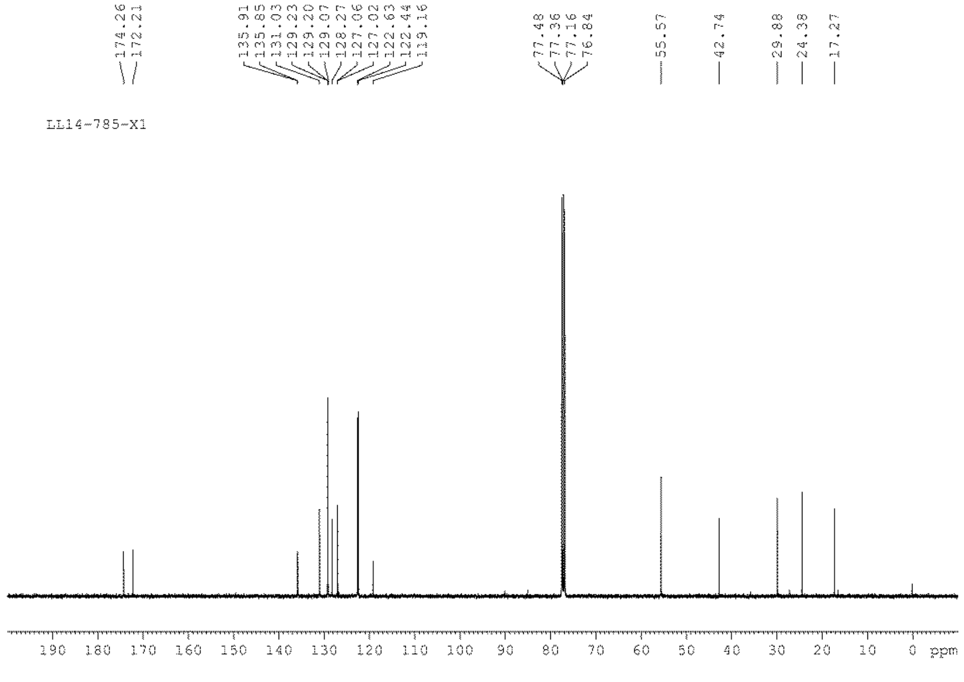


**Supplementary Figure 135**. $^{1}$H and $^{13}$C NMR spectra of product **3qa**

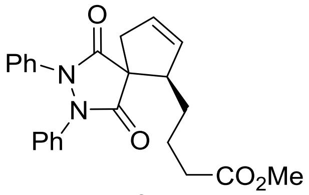


**3ra**

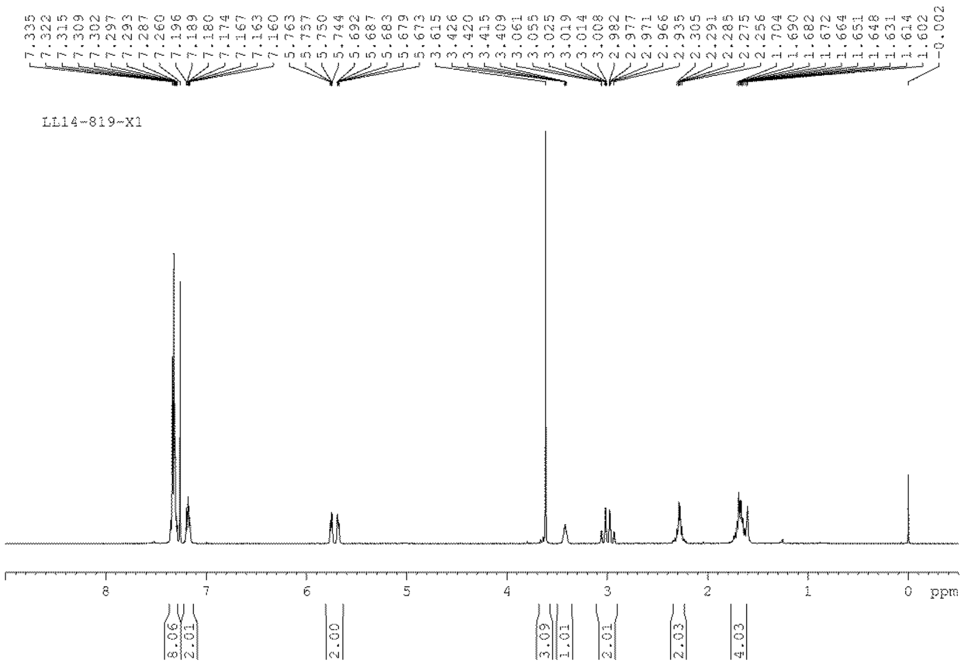


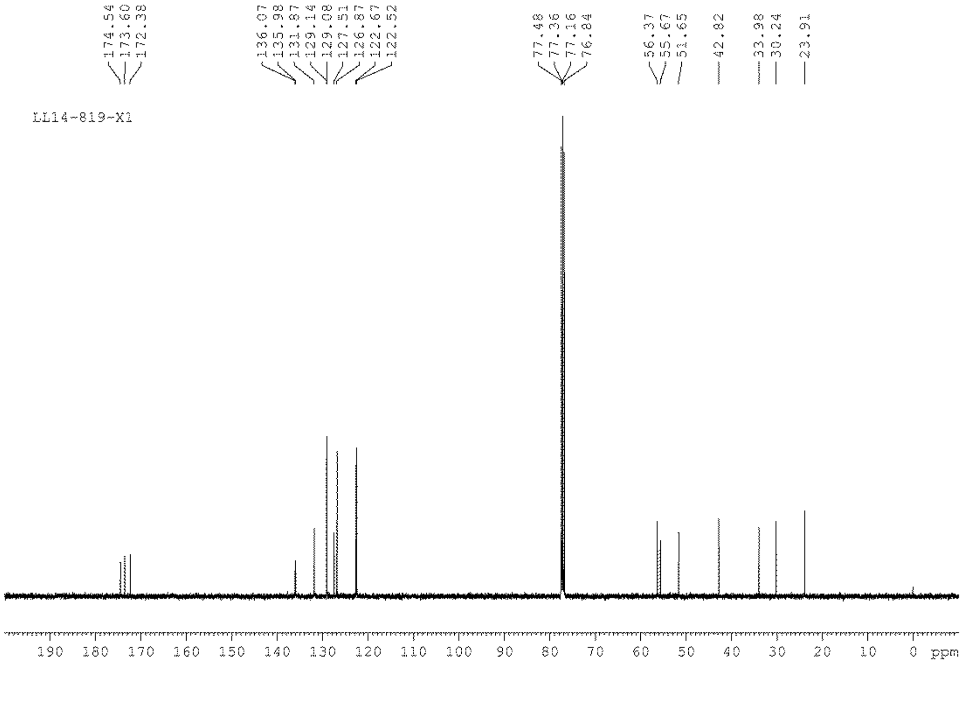


**Supplementary Figure 136**. $^{1}$H and $^{13}$C NMR spectra of product **3ra**

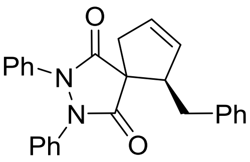


**3sa**

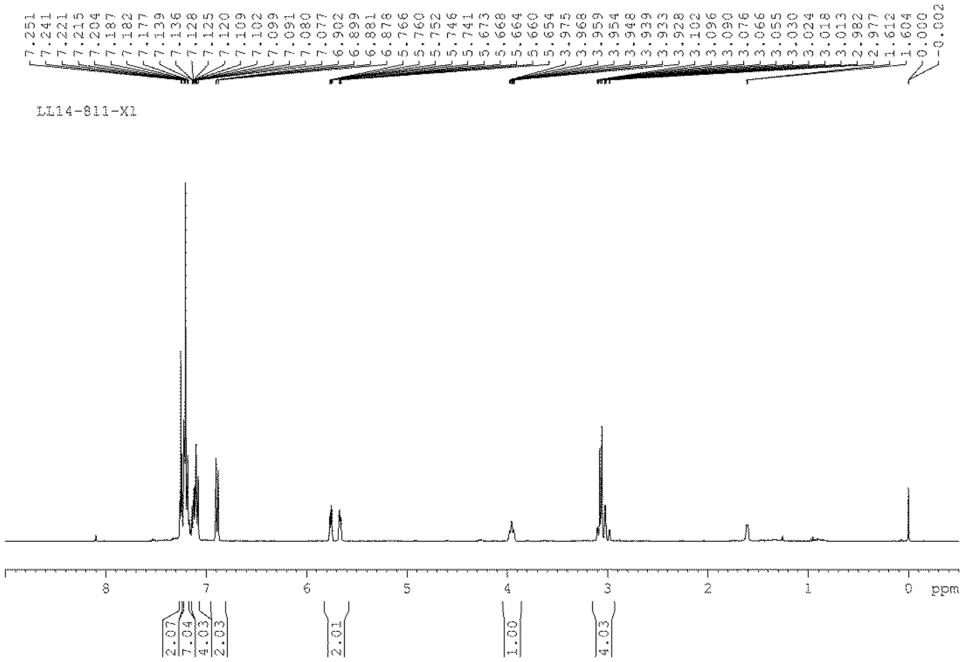


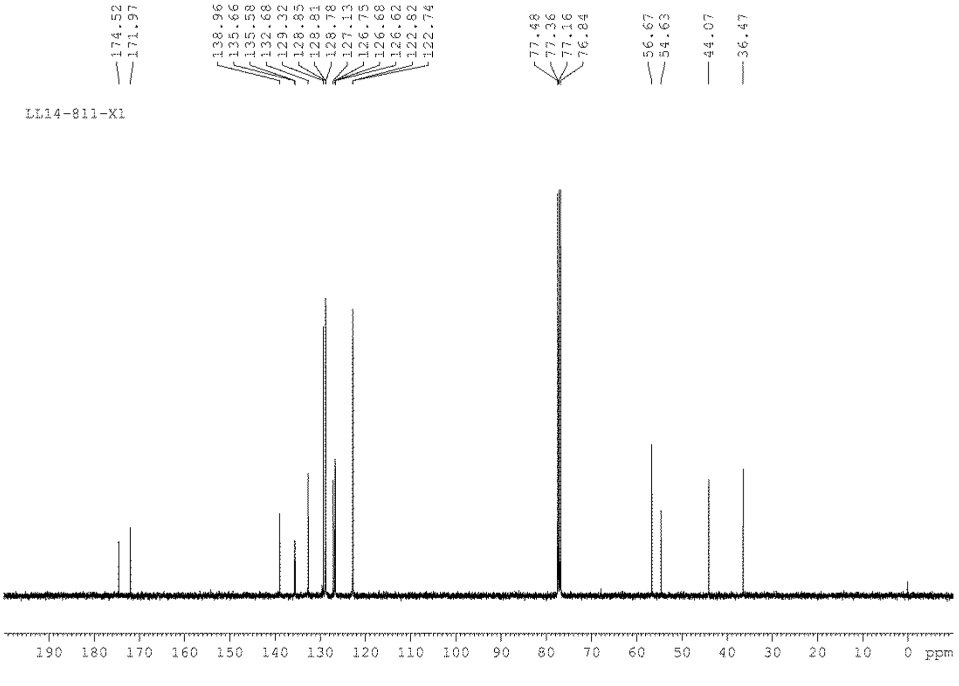


**Supplementary Figure 137**. $^{1}$H and $^{13}$C NMR spectra of product **3sa**

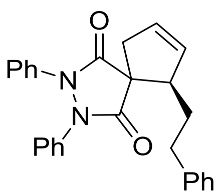


**3ta**

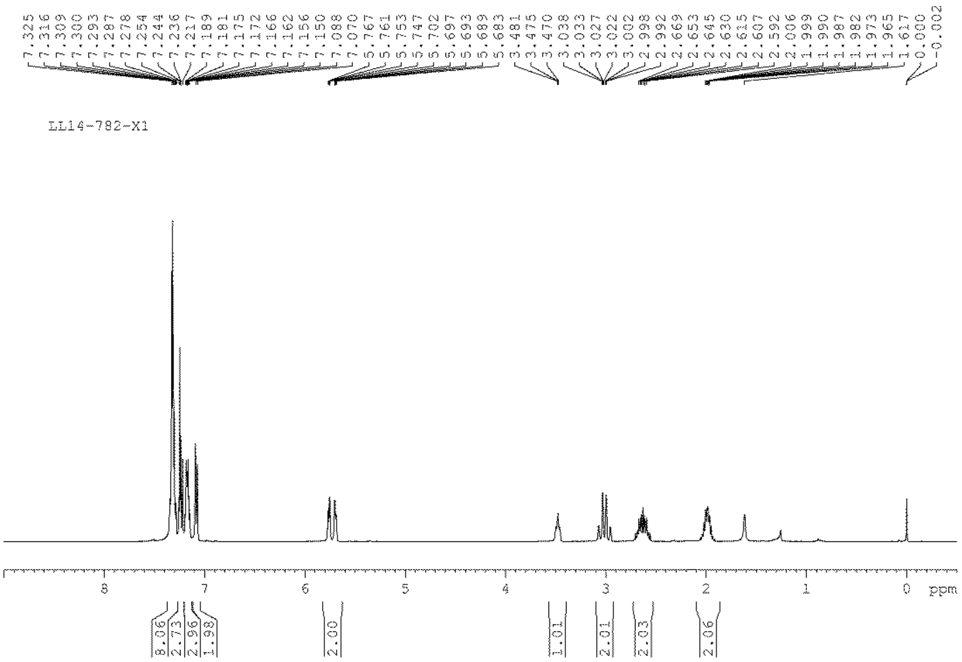


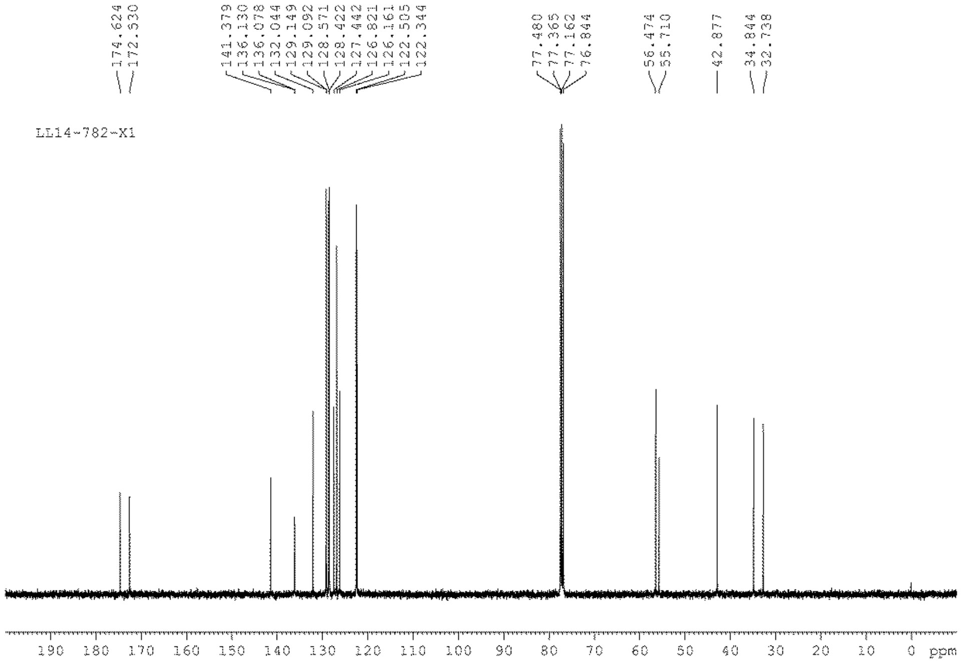


**Supplementary Figure 138**. $^{1}$H and $^{13}$C NMR spectra of product **3ta**

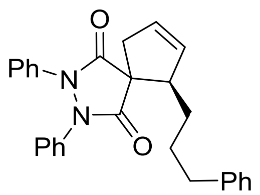


**3ua**

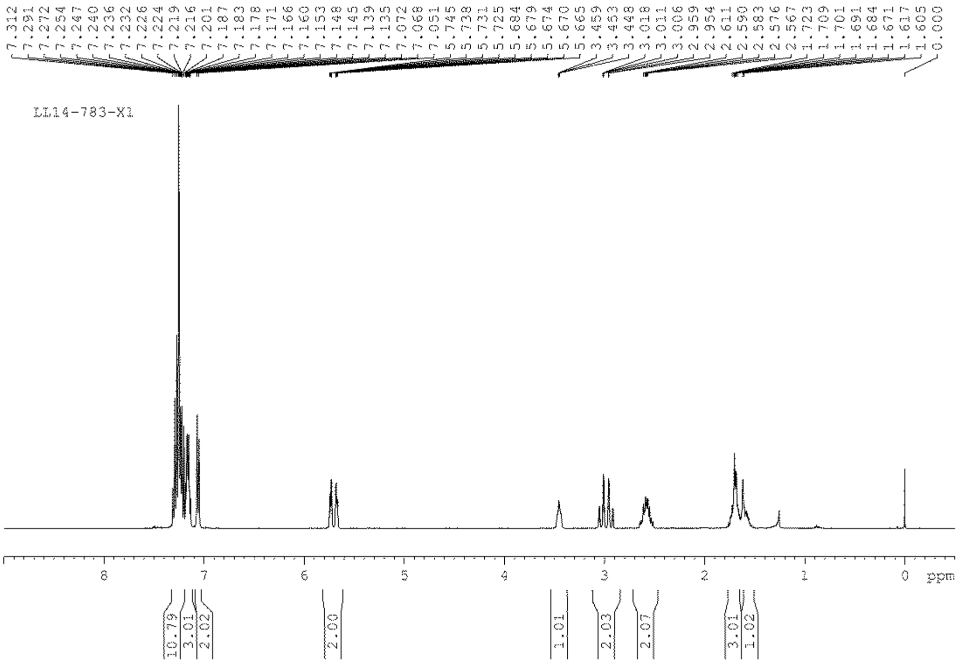


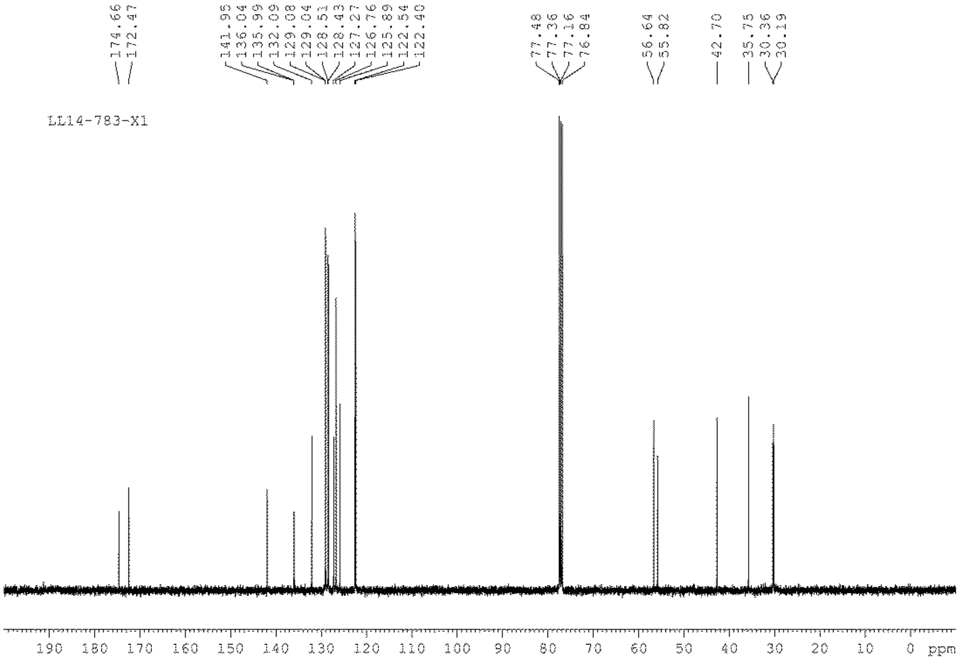


**Supplementary Figure 139**. $^{1}$H and $^{13}$C NMR spectra of product **3ua**

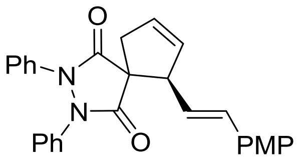


**3va**

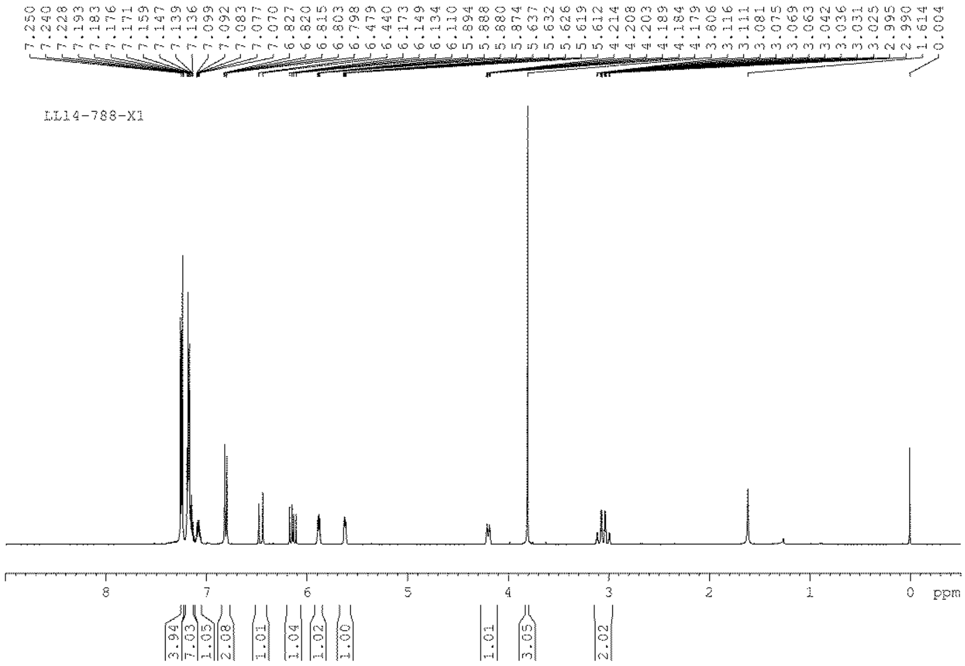


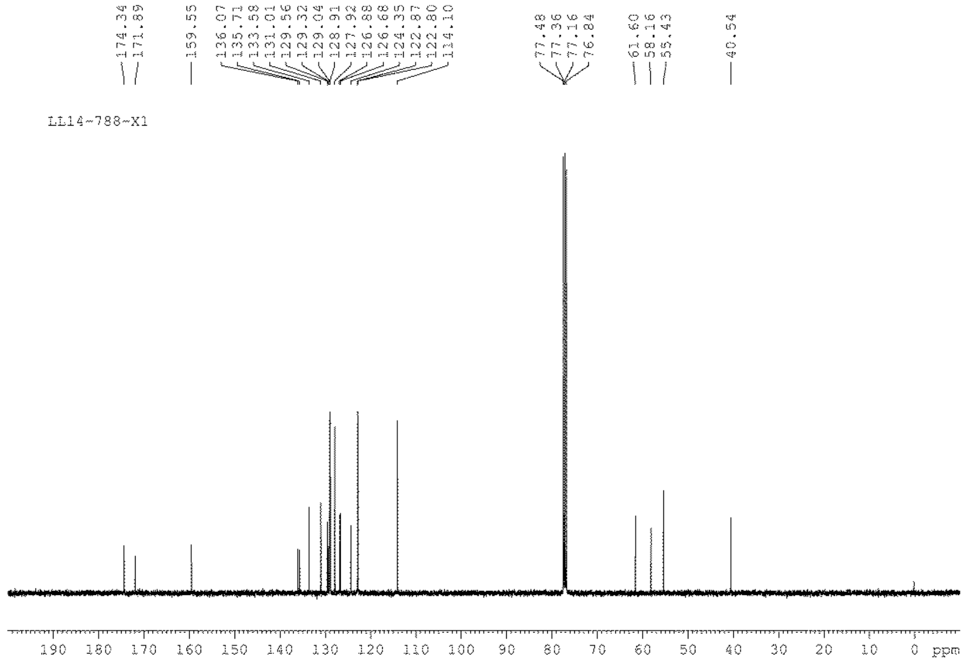


**Supplementary Figure 140**. $^{1}$H and $^{13}$C NMR spectra of product **3va**

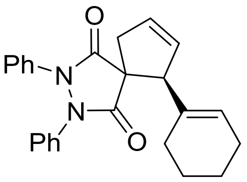

**3wa**

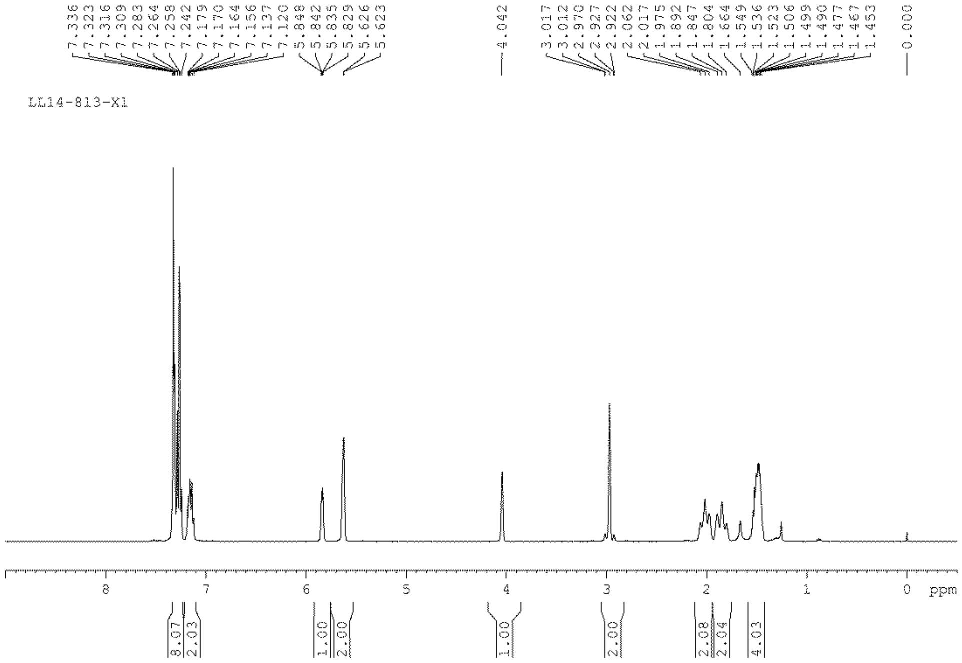


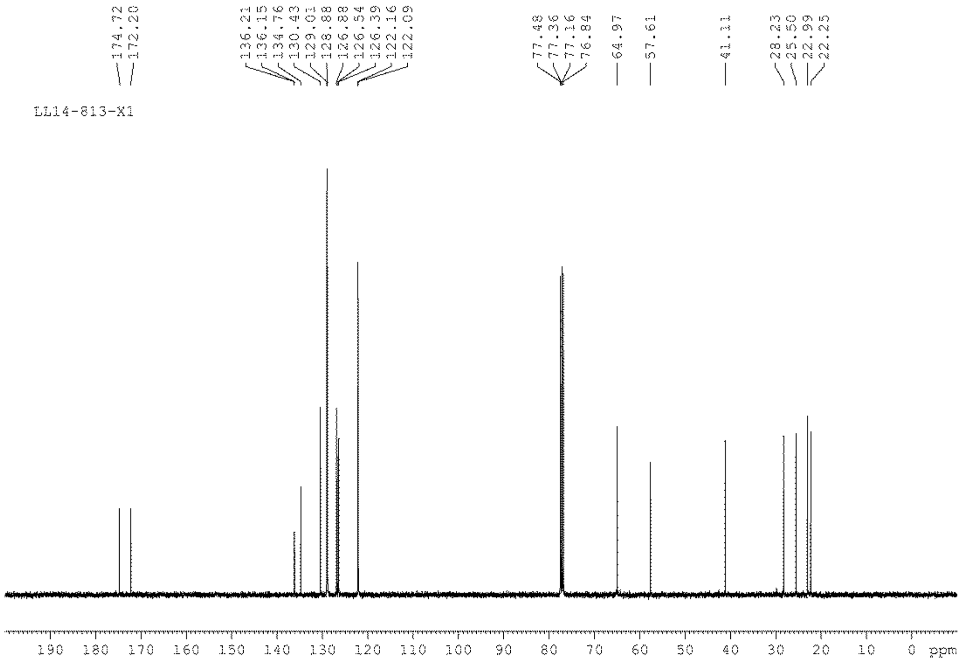


**Supplementary Figure 141**. $^{1}H$ and $^{13}C$ NMR spectra of product **3wa**

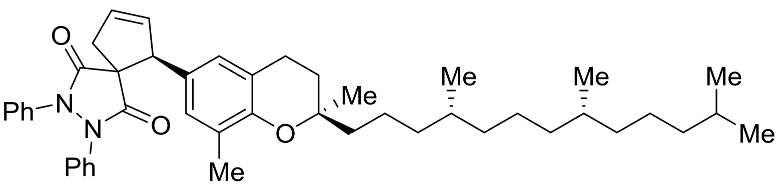


**3xa**

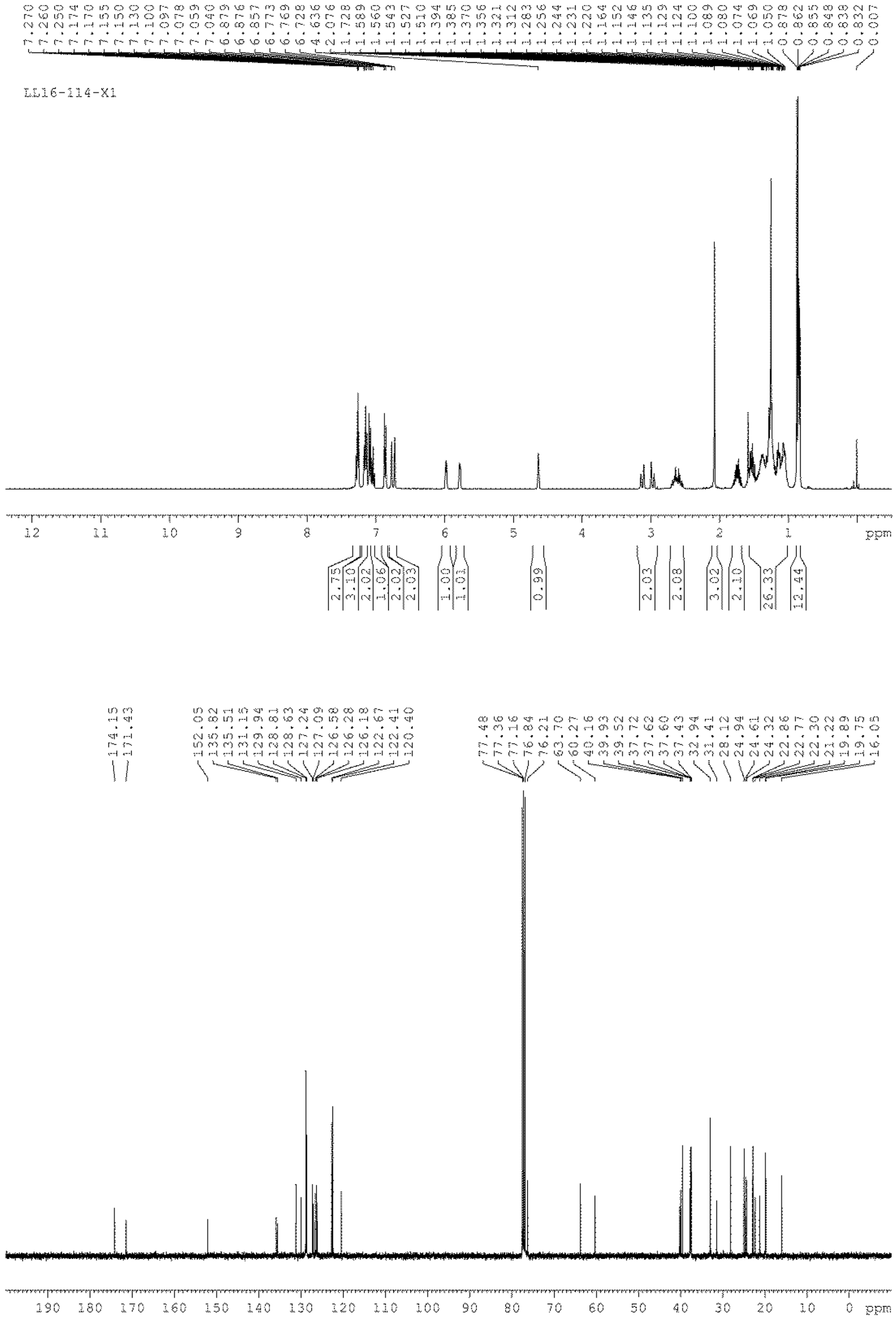


**Supplementary Figure 142**. $^{1}H$ and $^{13}C$ NMR spectra of product **3xa**

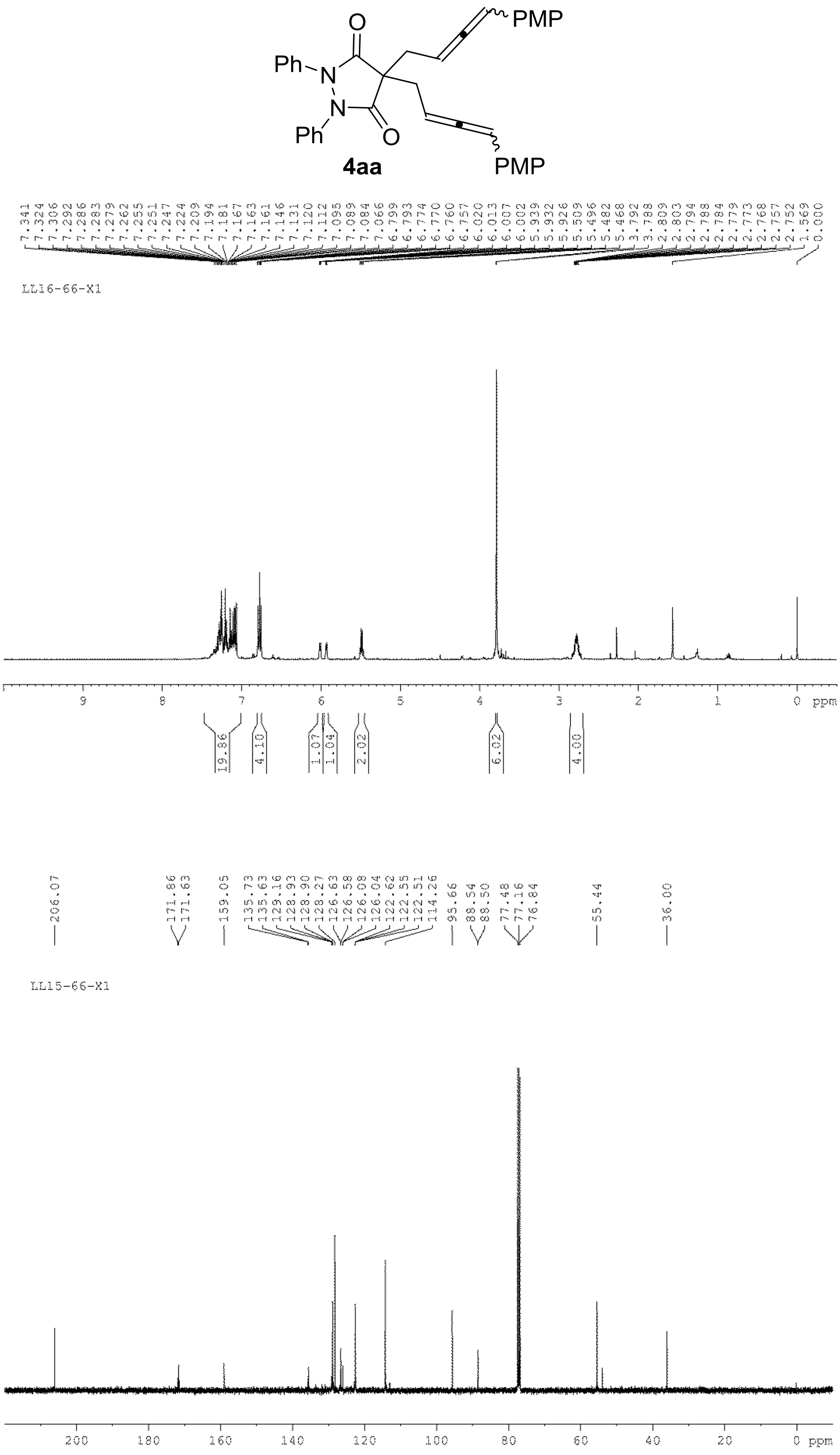


**Supplementary Figure 143**. $^{1}H$ and $^{13}C$ NMR spectra of product **4aa**

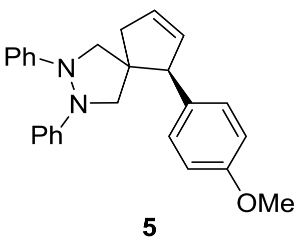


**5**

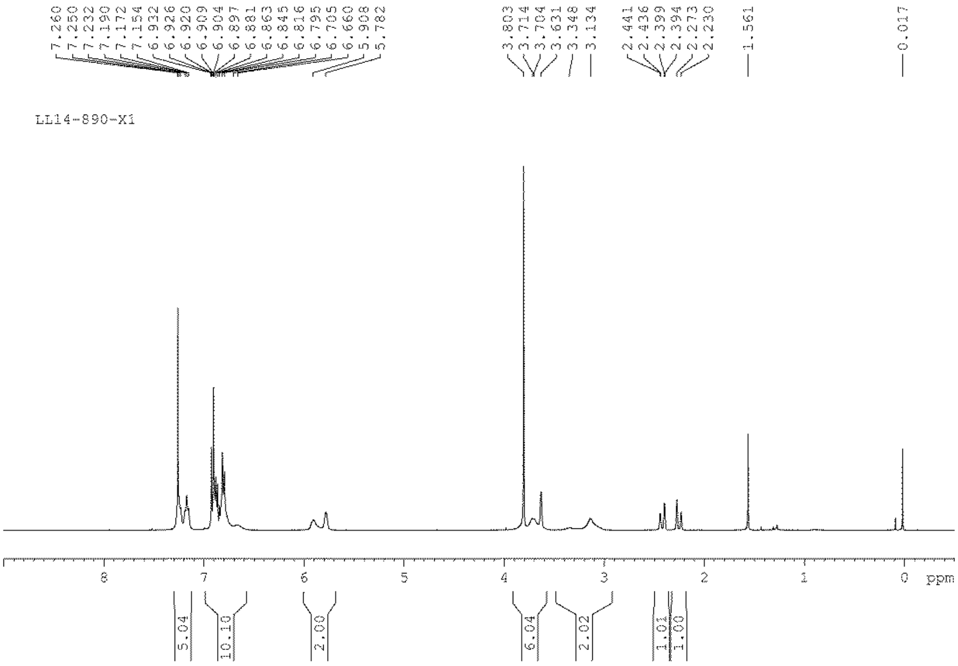


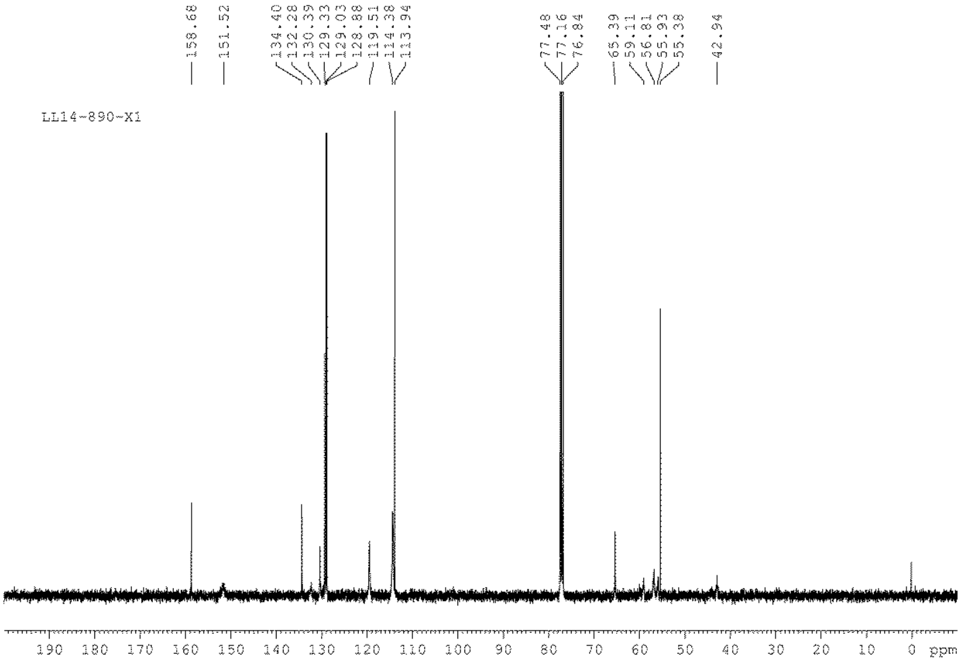


**Supplementary Figure 144**. $^1$H and $^{13}$C NMR spectra of product **5**

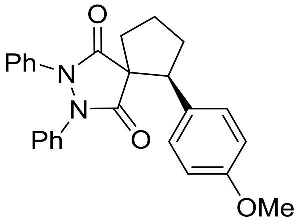


**6**

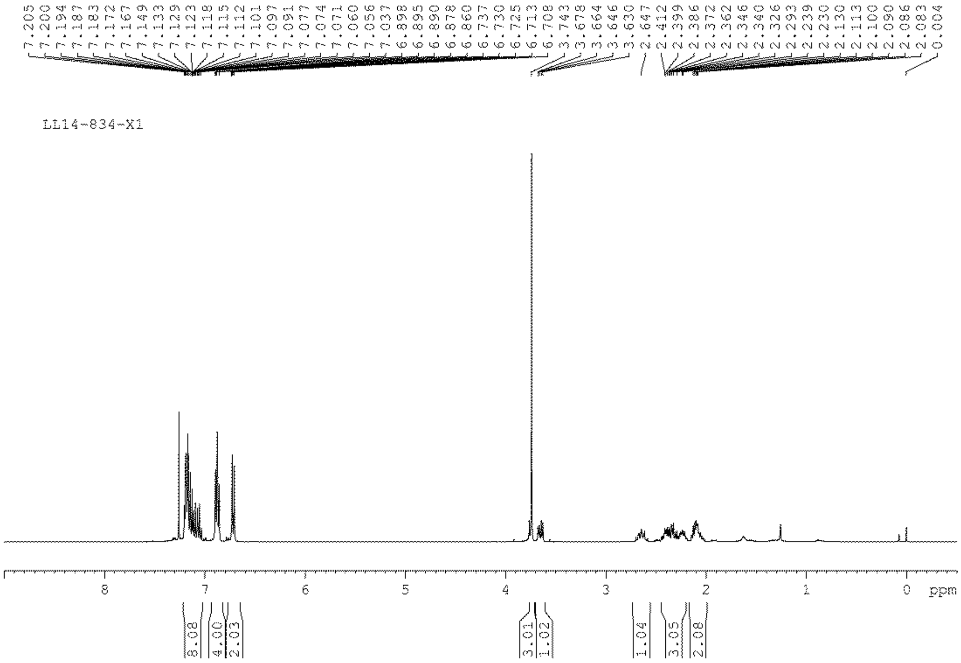


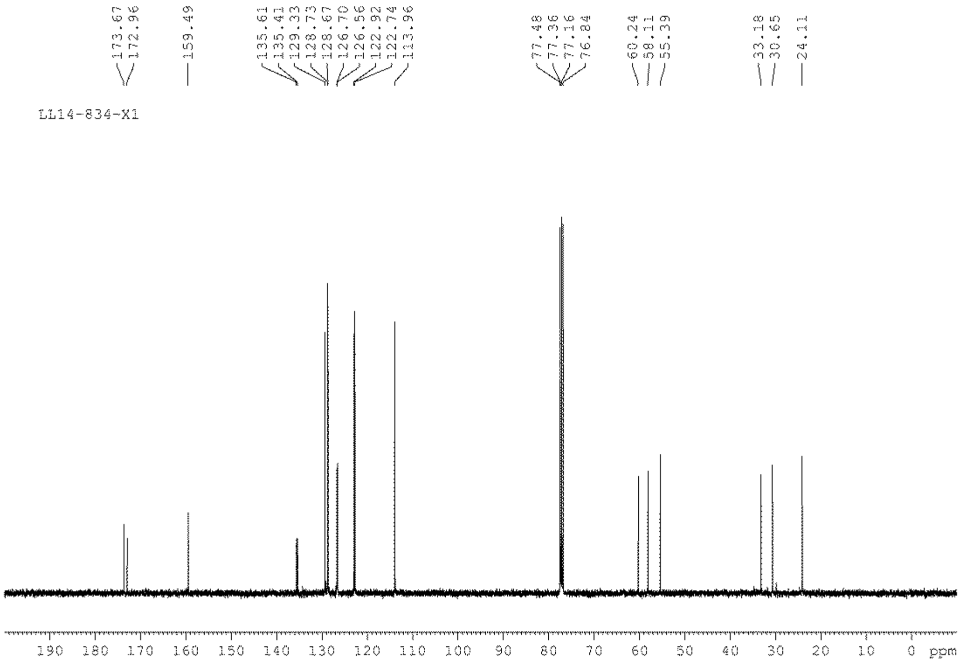


**Supplementary Figure 145**. $^{1}$H and $^{13}$C NMR spectra of product **6**

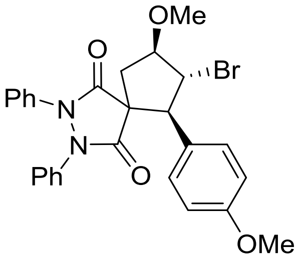


**7**

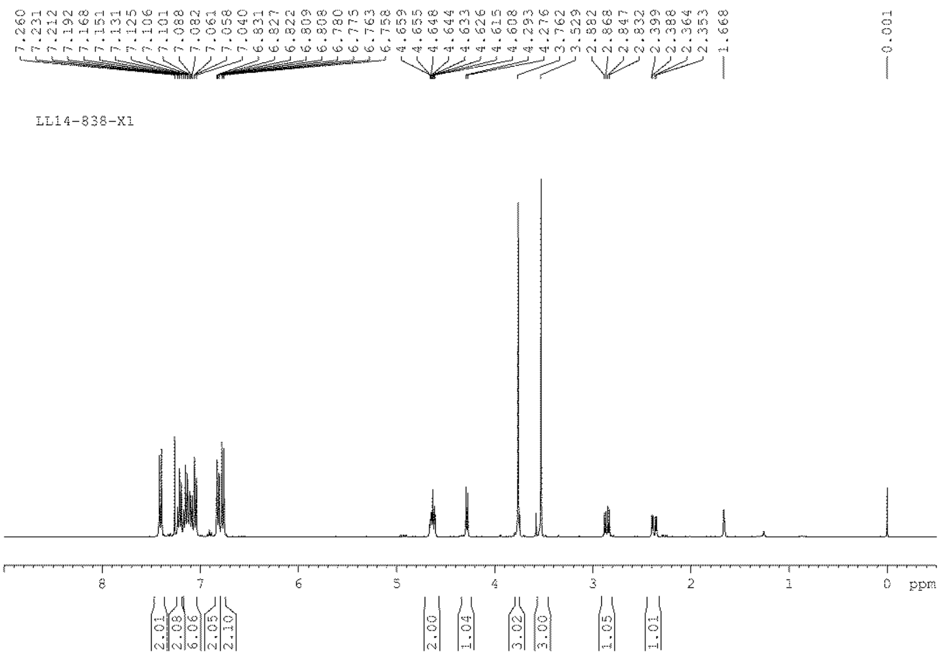


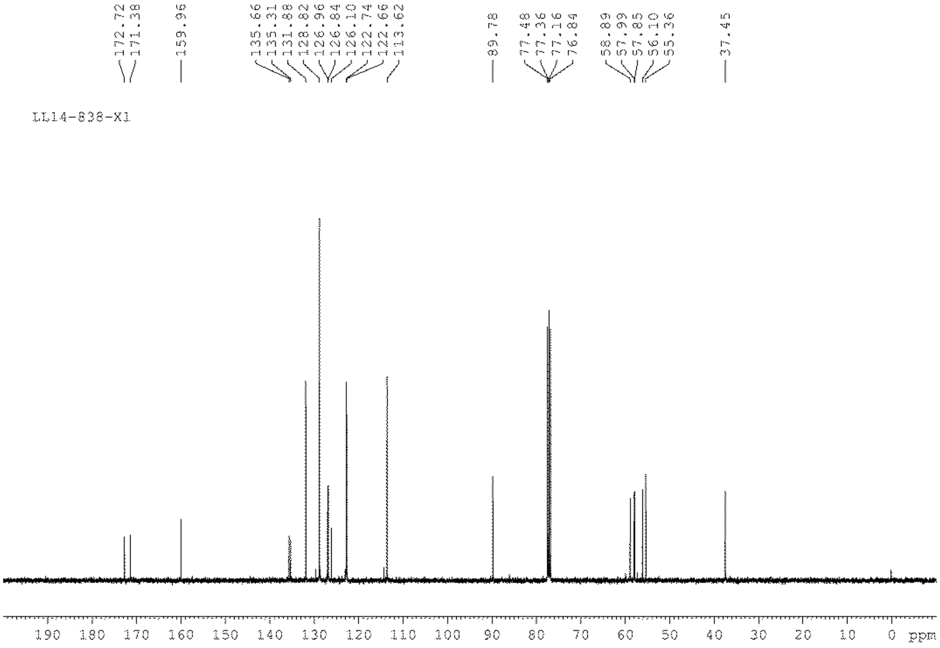


**Supplementary Figure 146**. $^{1}$H and $^{13}$C NMR spectra of product **7**

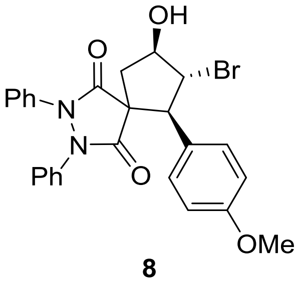

**8**

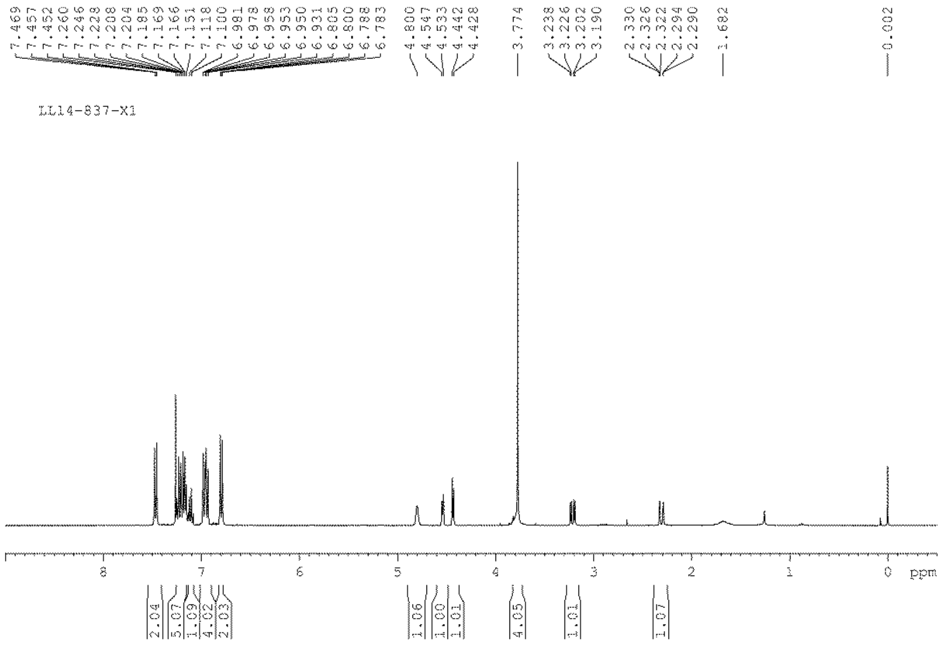

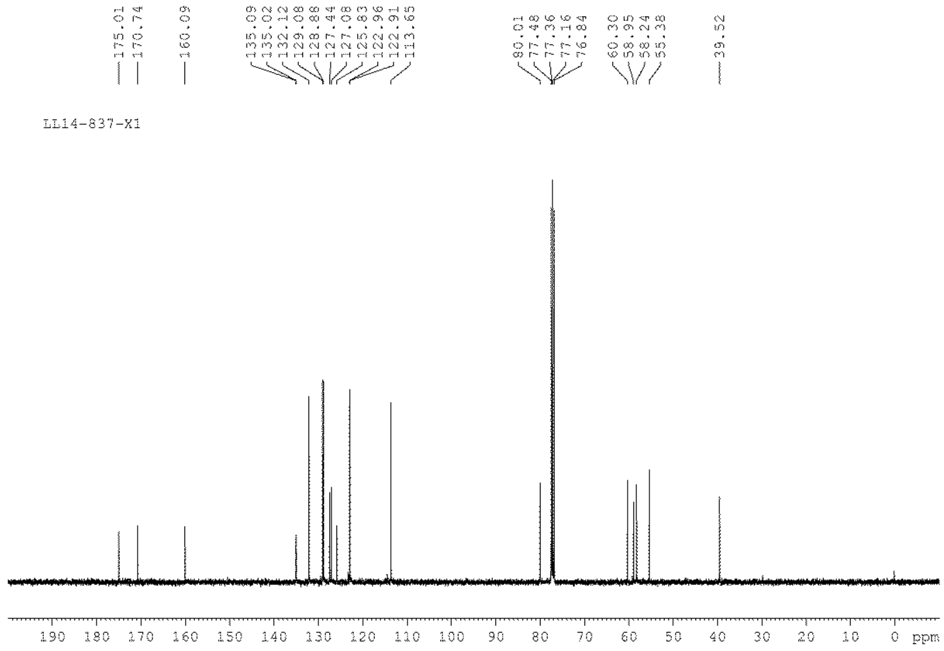

**Supplementary Figure 147**. $^{1}$H and $^{13}$C NMR spectra of product **8**

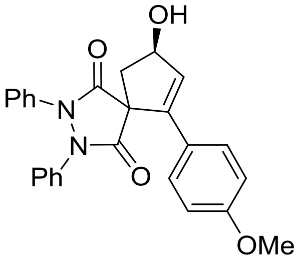


**9**

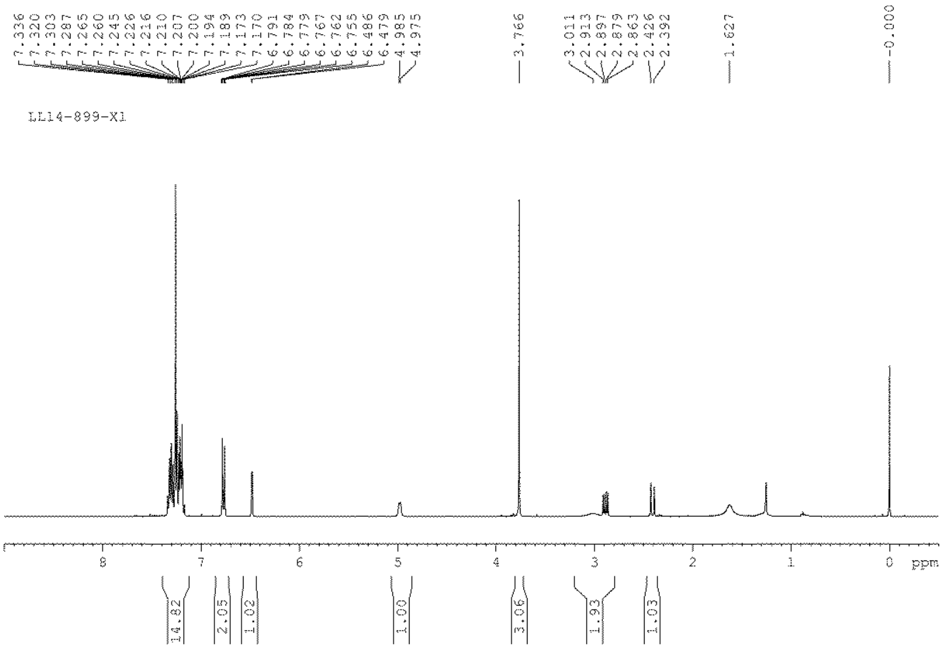


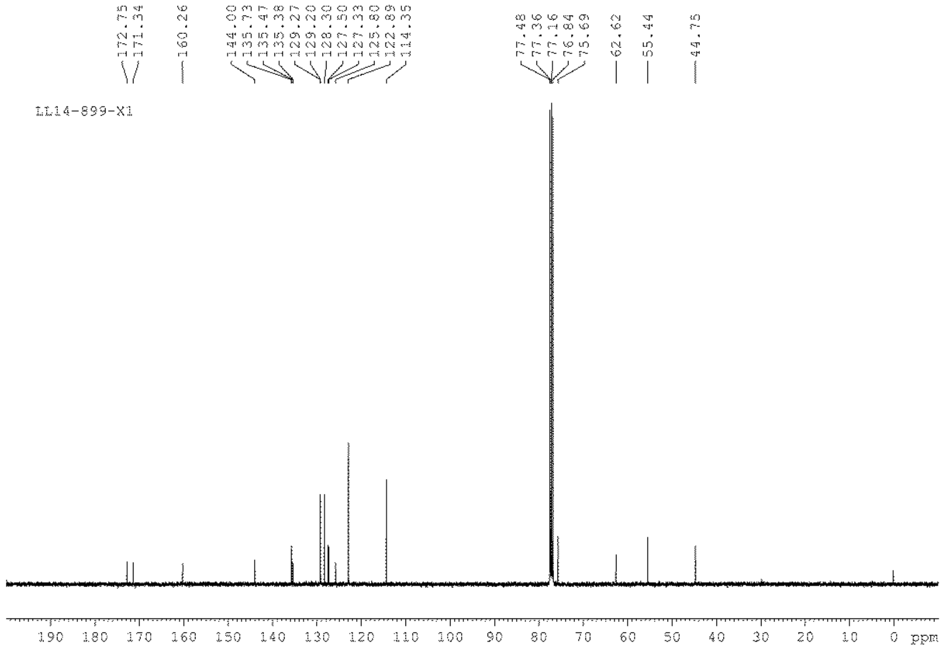


**Supplementary Figure 148**. $^{1}$H and $^{13}$C NMR spectra of product **9**

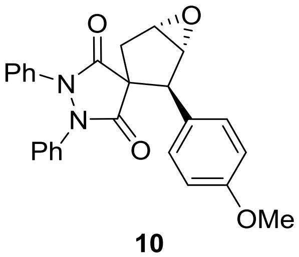


**10**

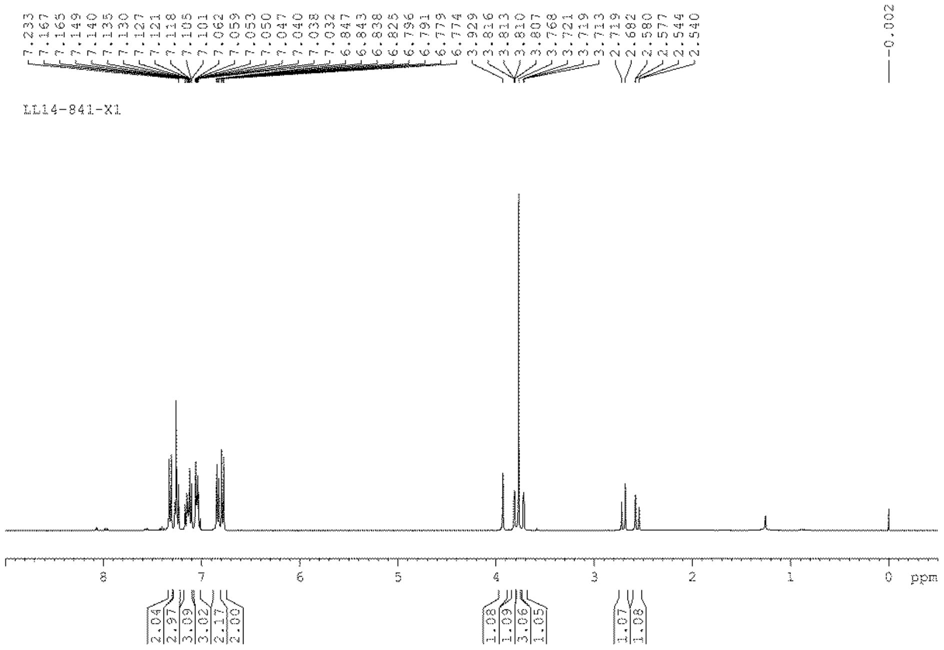


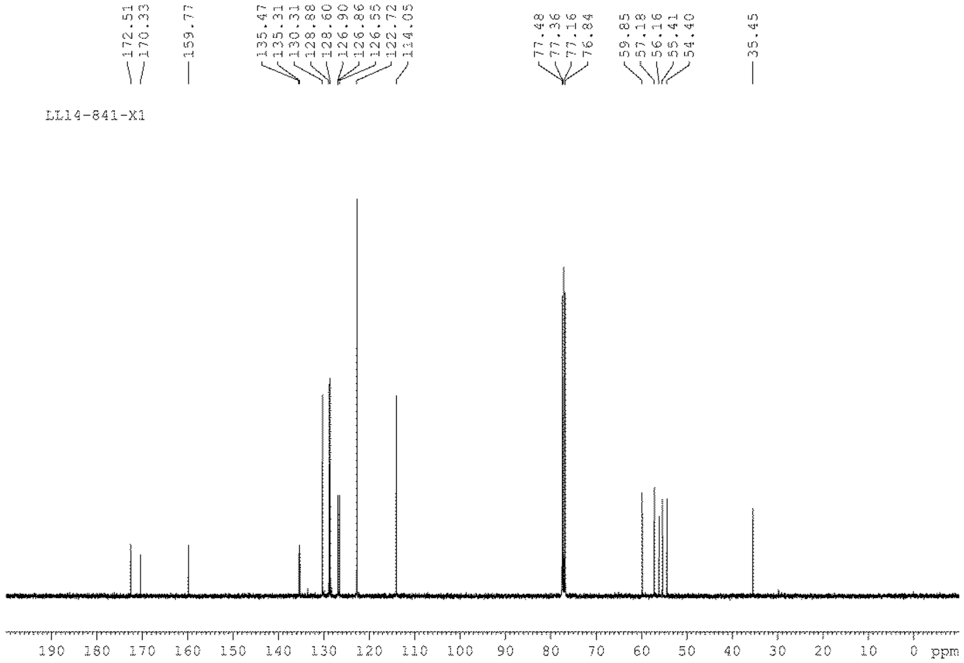


**Supplementary Figure 149**. $^{1}$H and $^{13}$C NMR spectra of product **10**

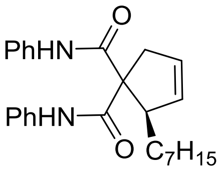


**11**

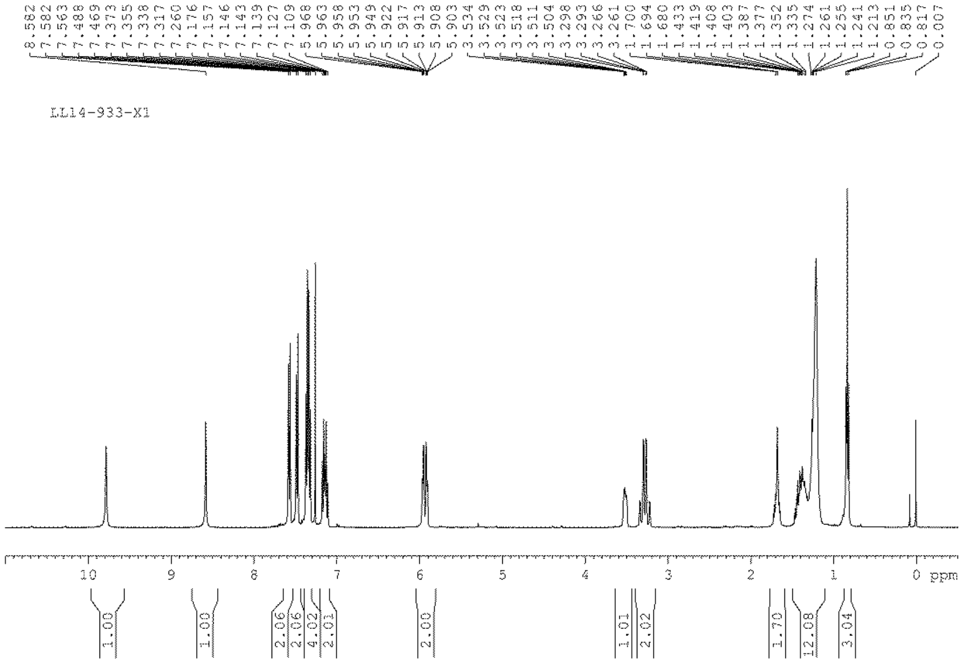


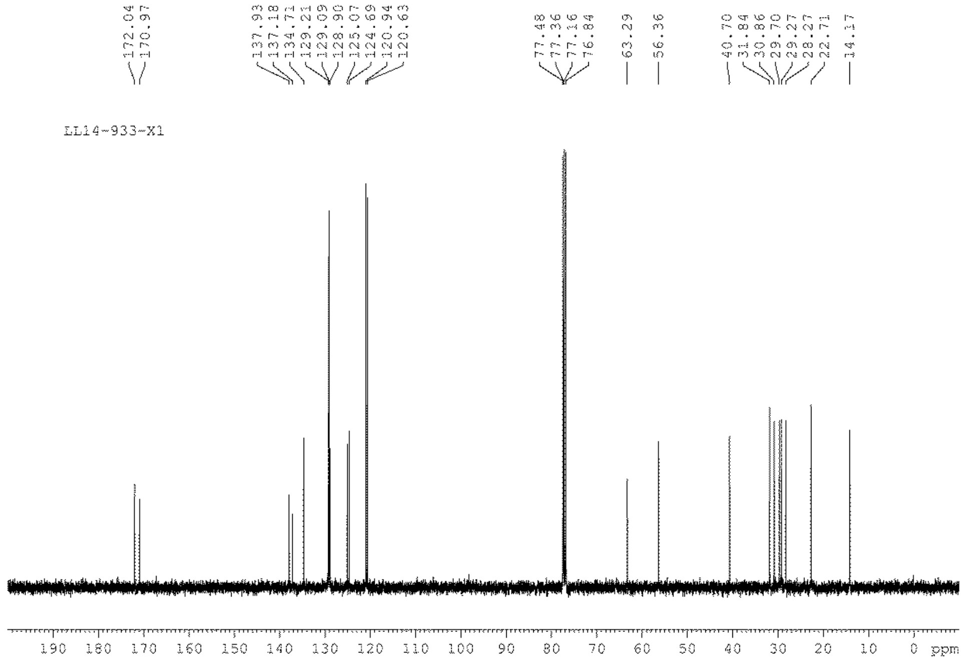


**Supplementary Figure 150**. $^{1}$H and $^{13}$C NMR spectra of product **11**

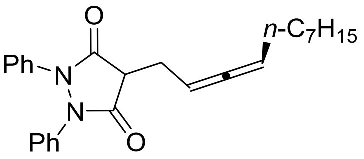


**13**

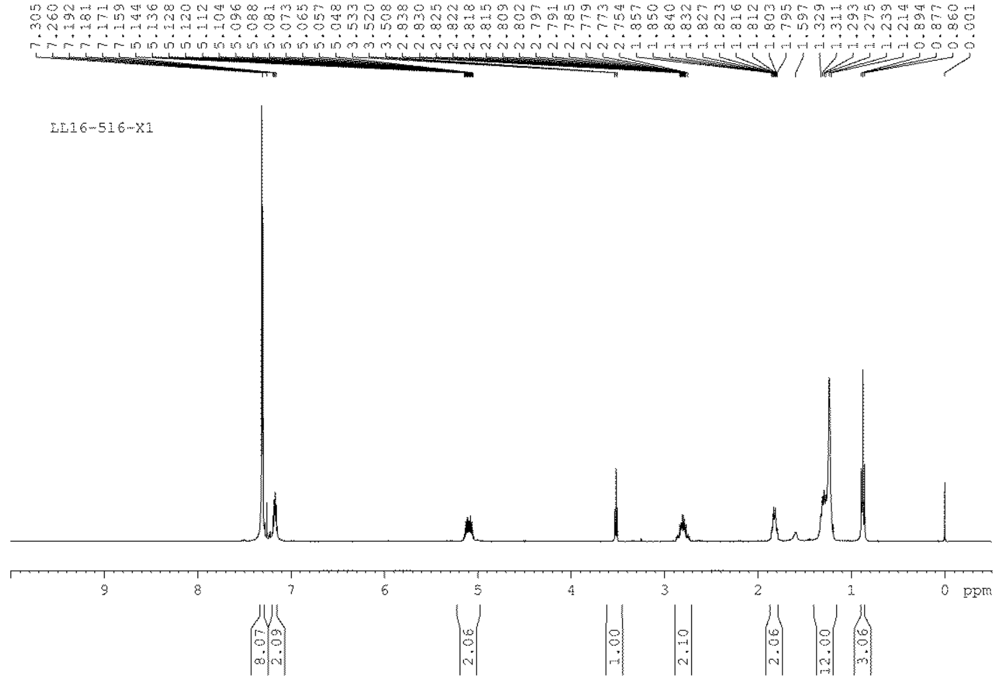


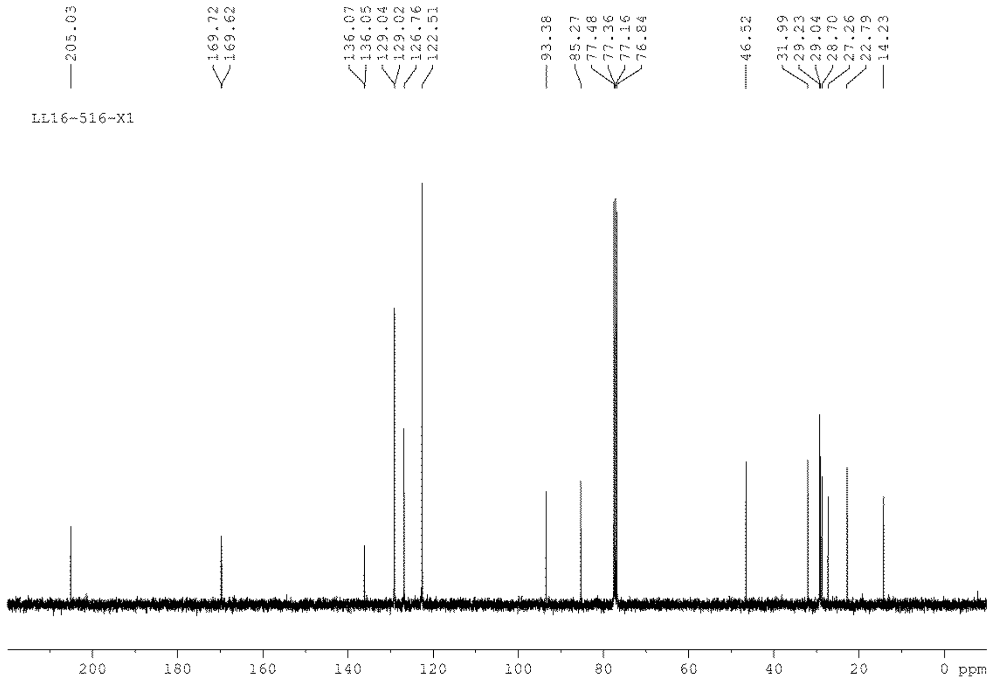


**Supplementary Figure 151**. $^{1}H$ and $^{13}C$ NMR spectra of product **13**

## 4. Supplementary References


1. Kinoshita, H., Ishikawa, T. & Miura, K. Dialkylaluminum hydride-promoted cyclodimerization of silylated 1,3-enynes via skeletal rearrangement. *Org. Lett.* **13**, 6192−6195 (2011).
2. Zhang, Y.-C., Yu, B.-K., Gao, B.-J., Zhang, T.-Z. & Huang, H.-M. Triple-bond insertion triggers highly regioselective 1,4-aminomethylamination of 1,3-enynes with aminals enabled by Pd-catalyzed C–N bond activation. *Org. Lett*. **21**, 535−539 (2019).
3. Huang, X. et al. General $CuBr_2$-catalyzed highly enantioselective approach for optically active allenols from terminal alkynols. *Chem. Commun.* **51**, 6956-6959 (2015).
